# Nasal-spraying *Bacillus* spores as an effective symptomatic treatment for children with acute respiratory syncytial virus infection

**DOI:** 10.1038/s41598-022-16136-z

**Published:** 2022-07-20

**Authors:** Dien Minh Tran, Tu Thanh Tran, Thuy Thi Bich Phung, Huyen Thi Bui, Phuc Thanh Thi Nguyen, Tam Thi Vu, Nga Thi Phuong Ngo, Mai Thi Nguyen, Anh Hoa Nguyen, Anh Thi Van Nguyen

**Affiliations:** 1 Department of Surgical Intensive Care Unit, National Children’s Hospital, No. 18/879 La Thanh, Dong Da, Hanoi, Vietnam; 2 International Center, National Children’s Hospital, No. 18/879 La Thanh, Dong Da, Hanoi, Vietnam; 3 Department of Molecular Biology for Infectious Diseases, National Children’s Hospital, No. 18/879 La Thanh, Dong Da, Hanoi, Vietnam; 4 Key Laboratory of Enzyme and Protein Technology, VNU University of Science, Vietnam National University, Hanoi, 334 Nguyen Trai, Thanh Xuan, Hanoi, Vietnam; 5 Spobiotic Research Center, ANABIO R&D Ltd. Company, No. 22, Lot 7,8 Van Khe Urban, La Khe, Ha Dong, Hanoi, Vietnam; 6 LiveSpo Pharma Ltd. Company, N03T5, Ngoai Giao Doan Urban, Bac Tu Liem, Hanoi, Vietnam

**Keywords:** Clinical microbiology, Randomized controlled trials, Viral infection

## Abstract

Respiratory syncytial virus (RSV) is a leading cause of Acute Respiratory Tract Infections (ARTIs) in young children. However, there is currently no vaccine or treatment available for children. Here, we demonstrated that nasal-spraying probiotics containing 5 billion of *Bacillus* spores (LiveSpo Navax) is an effective symptomatic treatment in a 6-day randomized controlled clinical study for RSV-infected children (*n* = 40–46/group). Navax treatment resulted in 1-day faster recovery-time and 10–50% better efficacy in relieving ARTI symptoms. At day 3, RSV load and level of pro-inflammatory cytokines in nasopharyngeal samples was reduced by 630 folds and 2.7–12.7 folds respectively. This showed 53-fold and 1.8–3.6-fold more effective than those in the control-standard of care-group. In summary, nasal-spraying *Bacillus* spores can rapidly and effectively relieve symptoms of RSV-induced ARTIs while exhibit strong impacts in reducing viral load and inflammation. Our nasal-spraying probiotics may provide a basis for simple-to-use, low-cost, and effective treatment against viral infection in general.

## Introduction

Respiratory syncytial virus (RSV) is the most common virus that causes Acute Respiratory Tract Infections (ARTIs) with high risk of serious bronchiolitis for young babies and infants. RSV is an enveloped negative-sense RNA virus, which belongs to the Paramyxoviridae family^1–3^. The clinical symptoms of RSV infection vary from slight fever, cough, runny nose, wheezing to more severe symptoms such as difficulty breathing, respiratory failure^1–3^. The World Health Organization estimates that 48,000–74,000 children younger than 5 years old die every year due to RSV infection^4–6^. Although inflammatory response to RSV is complicated, high levels of nuclear factor-kappa-B (NF-*κ* B) dependent pro-inflammatory cytokines such as tumor necrosis factor-α (TNF-α), Interleukin-6 (IL-6), and IL-8 in airways of children with bronchiolitis have been reported as potential biomarkers with prognostic value in RSV infection^7–10^. More specifically, it has been reported that IL-6 and IL-8 levels are highly elevated in primary RSV infection^11^. An extreme elevation of IL-6 is related to sudden death in children with RSV infection^8^.

RSV is very contagious due to its multiple routes of infection from person-to-person by getting into the eyes, nose, or mouth while easily spreading through the air via viral droplets^12^. Prevention of RSV infection is important, as repeated infections may occur throughout one’s lifetime^13,14^. Up to now, there is no available vaccine and specific treatment for RSV-infected children. Monoclonal antibody palivizumab therapy and antiviral nucleotide drug ribavirin are either expensive or unsafe for children, and is recommended only for high-risk patients^15,16^. Thus, treatment for pediatric RSV infection remains supportive. These include fever reduction, enhancing nutrition and cleaning nasal airway and surrounding environment to prevent co-infection bacteria and respiratory failure. Antibiotics are only used in the case of acquisition of bacterial co-infection, such as *Streptococcus pneumoniae* and *Haemophilus influenzae*^17–20^.

In recent years, preventive and supportive therapies for respiratory tract infection have been increasingly improved. Probiotics are considered as promising safe candidates for therapeutic support and reduction of antibiotic dependence^21^. It is suggested that probiotics can capture viruses through direct interactions, or produce secondary substances that inhibit virus growth, or stimulate the immune system to capture virus intrusion^22–24^. Recent human clinical trials and animal studies have shown that in addition to the traditional beneficial effects to the digestive tract, several probiotic strains including *Lactobacillus rhamnosus* GG^25–30^, *Bacillus coagulans* GBI-30, 6068^31^, *Lactobacillus gasseri* TMC0356^32^, *L. reuteri* F275^33^, and *Bifidobacterium lactis* Bb-12^25^, also have the ability to reduce the symptoms and prevent respiratory tract infections/re-infections. The major molecular mechanism is their ability to modulate pro-inflammatory cytokines including TNF-α, IL-6, IL-8^22,25,26,31,32^. Notably, *L. rhamnosus* GG probiotic strain has undergone extensive clinical tests. Functional food containing *L. rhamnosus* GG at > 10^8^ CFU/g, for example, have been shown to minimize the risk of upper and lower RTIs in children^27–29^. Another meta-analysis of four randomized controlled trials has found that *L. rhamnosus* GG can reduce the risk of upper RTIs (URTIs), the incidence of acute otitis media, and antibiotic use in children^30^. However, no evidence is yet available to support the use of *L. rhamnosus* GG probiotics in lowering the occurrence and concentration of viral respiratory infections such as RSV, influenza, adenovirus etc. in the nasal tract^22,28–30^.

Although previous human clinical trials have broadened the scope of probiotic applications in the field of respiratory infection, they are limited only in the use of oral administrative probiotics as functional food which are effective only for mild symptoms. These includes nasal congestion, itchy nose, hoarseness, or prevention of re-infection^22,25–32^. Furthermore, because probiotics containing non-spore forming bacterial strain is unstable in liquid-suspension formulation and can only be prepared as a powder or tablet for oral use, it is difficult to promote in-situ effectiveness in inhibiting viruses and bacteria in nasal tract. Thus, the efficacy of oral digested probiotics on the respiratory tract of children has been slow to develop (3–12 months), making such treatment mainly suitable for prevention and not applicable for supportive treatment of ARTIs^22,25–32^. Alternative delivery routes are needed for the use of probiotics in treatment of ARTIs. Here, we proposed that direct spraying of probiotics into the nose can be a fast and effective symptomatic treatment for ARTIs.

In this study, we conducted the first blind, randomized, and controlled clinical trial evaluating efficacy of nasal-spraying administrative probiotics in supporting treatment of children having acute and heavy respiratory symptom due to RSV infection. We formulated a 0.9% NaCl physiological saline suspension containing more than 5 billion/5 mL of highly purified spores of two bacterial strains *B. subtilis* ANA4 and *B. clausii* ANA39 (LiveSpo Navax), which can be easily administrated to patients via nasal spraying. We observed rapid efficacy in RSV-induced symptomatic relief of the nasal-spraying *Bacillus* spore probiotics during 6-day follow-up treatment period in hospital. Furthermore, reduction in viral load and co-infection bacteria as well as modulation of overreacted cytokine release were evident in the group using LiveSpo Navax at day 3 of the treatment. Therefore, we propose that nasal-spraying probiotics is an effective symptomatic treatment for pediatric ARTIs, for which there is currently no available vaccination or pharmacological therapy.

## Results

### Trial design and patient baseline demographic, clinical and sub-clinical characteristics

**Figure 1:**
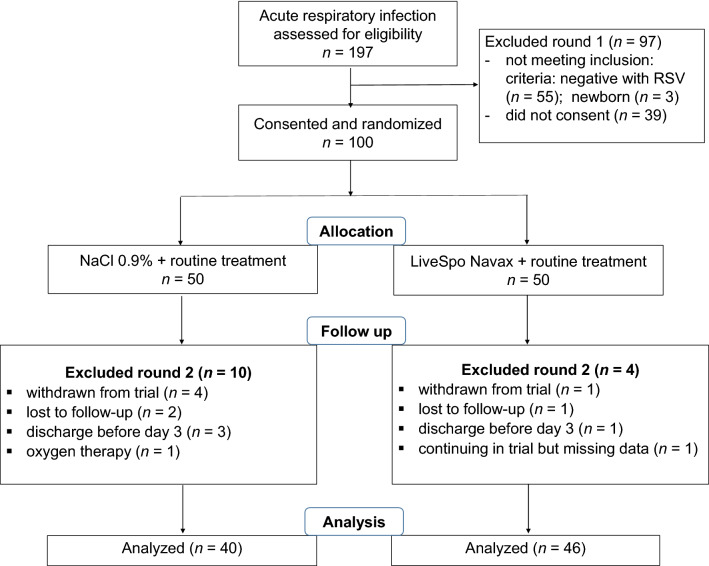
Diagram displaying the flow of participants involved in the study. From the clinical database, participants were screened for eligibility. Eligible participants who provided consent were randomized in either Control or Navax group. Measurements took place between baseline (day 0) and day 6 of treatment period. Participants were recruited from August 2020 to July 2021. Clinical and subclinical data collection and analysis were conducted from September 2020 to August 2021.

A total of 197 participants were screened for eligibility from August 2020 to July 2021. Among them, 100 eligible RSV-infected participants were randomized to the Control standard of care group (50 patients) and to the Navax testing group (50 patients) (Fig. 1—exclusion round 1). During the follow-up of treatment period of maximal 6 days, 10 in the Control group and 4 in the Navax group were excluded, resulting in 40 in the Control group and 46 in the Navax group was included in the analysis by the end of intervention (Fig. 1—exclusion round 2).

As shown in Table 1, RSV-infected participants were from 4 to 60 months in age and there were no statistically significant differences in age or gender distribution between groups (*p* = 0.56). Patients in both groups were hospitalized either on the third or fourth day of their sickness (*p* = 0.85). Before treatment, the participants’ baseline clinical characteristics including runny nose, chest depression, difficulty breathing, dry rales, moist rales, body temperature (°C), pulse oxymetry (SpO_2_) (%), pulse (beats/min), and breath (beats/min) were not statistically different between two groups (all *p* values > 0.05) (Table 1). Similarly, the baseline sub-clinical characteristics i.e., cardiopulmonary X-ray, threshold cycles (C_t_) of real-time PCR for RSV load in nasopharyngeal samples, total white blood cells, serum C-reactive protein (CRP) and bacterial co-infection cases represented no significant difference between the two groups before treatment (all *p* values > 0.05) (Table 2). In the trial, patients in the Control group received the routine treatment (Table 2- routine treatment) and three times per day 0.9% NaCl physiological saline while patients in the Navax group received three times per day 0.9% NaCl physiological saline containing *Bacillus* spores (LiveSpo Navax) in addition to the same standard of care treatment in the International Department, Vietnam National Children's Hospital. The standard treatment regimen is 3–6 days but can be extended further depending on the severity of the patients' respiratory failure. Patients were evaluated for subclinical signs on day 0 (the day of hospital admission) and day 3. Clinical symptoms were also assessed every day until they were discharged from the hospital. During a 6-day follow-up observation, the safety and efficacy were assessed either in a time-dependent manner or at day 3.

**Table 1:** Demographic and clinical characteristics of RSV-infected children before, during, and after treatment.

Characteristic	Control group (N = 40)			Navax group (N = 46)			*p* value		
	Before treatment	After treatment		Before treatment	After treatment		Before treatment	After treatment	
	Day 0	Day 3	Day 6	Day 0	Day 3	Day 6	Day 0	Day 3	Day 6
**Age (months)**									
≥ 3–12. *n* (%)	21 (52.50)			27 (58.70)			0.56		
> 12–60. *n* (%)	19 (47.50)			19 (41.30)					
**Gender**									
Male *n* (%)	20 (50.00)			32 (69.57)			0.07		
Female *n* (%)	20 (50.00)			14 (30.43)					
**Days of sickness before treatment**	3.6			3.7			0.85		
**Typical symptoms due to RSV infection**									
Runny nose *n* (%)	40 (100)	38 (95.00)	7 (17.50)	46 (100)	37 (80.43)	0 (0.00)	0.95	0.06	0.04
Chest depression *n* (%)	11 (27.50)	3 (7.50)	0 (0.00)	13 (28.26)	0 (0.00)	0 (0.00)	0.94	0.16	-^a^
Difficulty breathing *n* (%)	11 (27.50)	4 (10.00)	0 (0.00)	13 (28.26)	0 (0.00)	0 (0.00)	0.94	0.11	- ^a^
Dry rales *n* (%)	37 (92.50)	33 (82.50)	0 (0.00)	46 (100)	30 (65.22)	0 (0.00)	0.16	0.08	-^a^
Moist rales *n* (%)	28 (70.00)	22 (55.00)	1 (2.50)	33 (71.74)	15 (32.61)	1 (2.17)	0.86	0.04	0.92
**Temperature (°C)**									
Normal (≤ 37.5). *n* (%)	36 (90.00)	40 (100)	40 (100)	43 (93.33)	46 (100)	46 (100)	0.56	-^a^	-^a^
Fever (> 37.5). *n* (%)	4 (10.00)	0 (0.00)	0 (0.00)	3 (6.52)	0 (0.00)	0 (0.00)			
**SpO**_**2**_** (%)**									
≥ 95%. *n* (%)	39 (97.50)	40 (100)	40 (100)	44 (95.65)	46 (100)	46 (100)	0.65	-^a^	-^a^
< 95%. *n* (%)	1 (2.50)	0 (0.00)	0 (0.00)	2 (4.35)	0 (0.00)	0 (0.00)			
**Pulse (beats/min)**									
≥ 4–12 months (> 140). n (%)	7 (17.50)	3 (7.50)	0 (0.00)	10 (21.74)	0 (0.00)	0 (0.00)	0.62	0.16	-^a^
> 12 months (> 120). *n* (%)	17 (42.50)	12 (30.00)	0 (0.00)	16 (34.78)	4 (8.70)	0 (0.00)	0.46	0.02	-^a^
**Breath (beats/min)**									
≥ 4–12 months (> 40). n (%)	3 (7.50)	1 (2.50)	0 (0.00)	3 (6.52)	0 (0.00)	0 (0.00)	0.86	0.44	-^a^
> 12 months (> 32). *n* (%)	8 (20.00)	0 (0.00)	0 (0.00)	8 (17.39)	0 (0.00)	0 (0.00)	0.76	-^a^	-^a^

-^a^There is no remained patients to compare the significance.

**Table 2:** Sub-clinical characteristics of RSV-infected children before treatment.

Characteristic	Control group (N = 40)		Navax group (N = 46)		*p* value
	Total. *n* (%)	Min–max	Total. *n* (%)	Min–max	
**Cardiopulmonary X-ray**					
Osler's nodes	21 (52.50)		20 (43.48)		0.40
Hyperinflation	3 (7.50)		8 (17.39)		0.18
Osler's nodes and hyperinflation	11 (27.50)		12 (26.09)		0.88
Mass and hyperinflation	1 (2.50)		2 (4.35)		0.65
Osler’s nodes and mass	0 (0.00)		1 (2.17)		0.55
Normal	4 (10.00)		3 (6.52)		0.56
**RSV positive**					
Rapid test	40 (100)		46 (100)		0.95
Real-time PCR (C_t_)	40 (100)	16.6–29.80	46 (100)	15.47–26.93	0.95
**Hematology and biochemistry**					
Total white blood cells (G/L)		5.11–27.49		2.19–32.60	
< 6.0	3 (7.50)		2 (4.35)		0.54
6.0–10.0 (G/L)	13 (32.50)		24 (52.17)		0.07
> 10.0 (G/L)	24 (60.00)		20 (43.48)		0.13
CRP (mg/L)		0.15–42.26		0.09–53.39	
≤ 6.0	24 (60.00)		35 (76.90)		0.11
> 6.0	16 (40.00)		11 (23.91)		
**Bacterial co-infection**					
*H. influenzae*	10 (25.00)		8 (17.39)		0.39
*S. pneumoniae*	7 (17.50)		14 (30.43)		0.17
*H. influenzae & S. pneumoniae*	0 (0.00)		1 (2.17)		0.55
*M. catarrhalis*	3 (7.50)		5 (10.87)		0.59
*S. aureus*	0 (0.00)		1 (2.17)		0.55
**Treatment therapy**					
Routine treatment	Oral administrative drugs*:* antipyretic paracetamol (Efferalgan); anti-inflammatory corticosteroid methylprednisolon; antibiotics e.g. ampicillin and sulbactam complex (Ama-Power), tobramycin (Medphatobra), or cefotaxim (Goldcefo), based on the results of antibiotic susceptibility test Aerosol therapy: bronchodilator e.g. salbutamol (Ventolin Inhaler) or budesonide (Pulmicort Respules)				
Nasal-spraying treatment	NaCl 0.9%		NaCl 0.9% plus *B. subtilis* and *B. clausii* at 5 billion CFU/5 mL (LiveSpo Navax)		

### Safety and symptomatic-relieving effects of nasal-spraying *Bacillus* spores

**Figure 2:**
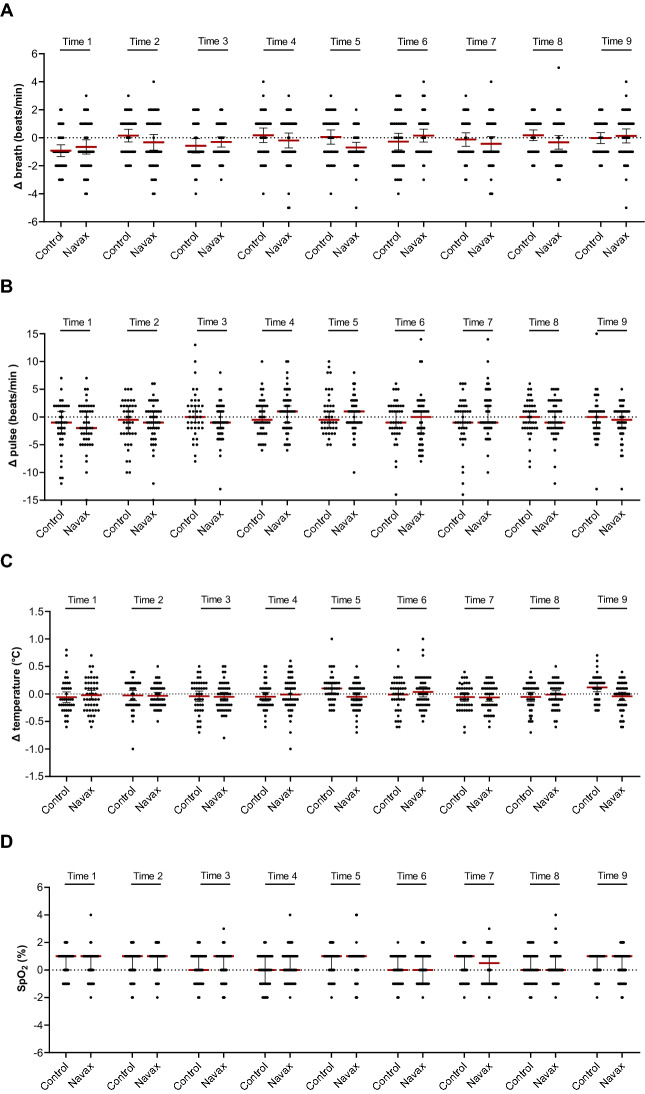
Average values of recorded changes in breath (**A**), pulse (**B**), temperature (**C**) and pulse oxygen (**D**) between before and after nasal-spraying with LiveSpo Navax (in diagonal-strip pattern) and 0.9% NaCl physiological saline (in white pattern), across 9 spraying times over 3 days.

During the entire treatment period, we did not record any case with abnormal changes of breathing, pulse, body temperature, and pulse oxymetry upon spraying *Bacillus* spores. Within the first three days, average values of recorded changes (Δ) in the four clinical indicators before and after each spraying of *Bacillus* spores (Navax group) and 0.9% NaCl physiological saline (Control group) were compared. As shown in Fig. 2, during 9 sprays, Δ breath (beats/min) and Δ pulse (beats/min) values of both groups fluctuated within safe limits of 2 beats/min on average, ranging from − 5 to + 5 beats/min for Δ breath and from − 14 to + 15 beats/min for Δ pulse for individuals. In the meantime, we recorded that Δ temperature (°C) slightly decreased (within 0.1 °C on average, ranging from − 1 to + 0.8 °C for individuals) and Δ SpO_2_ (%) slightly increased (within 1% on average, ranging from − 2 to + 4% for individuals) after nasal spraying in both groups. When sprayed, all participants in both groups did not choke, showed no signs of nasal mucosa irritation, had no symptoms of local bacterial infection, or had any digestive problems such as vomiting, diarrhea. During the observed treatment period, no patient reported abnormality in vital signs. Taken together, the data indicated that nasal-spraying *Bacillus* spores administration is safe and comfortable for pediatric RSV patients.

**Figure 3:**
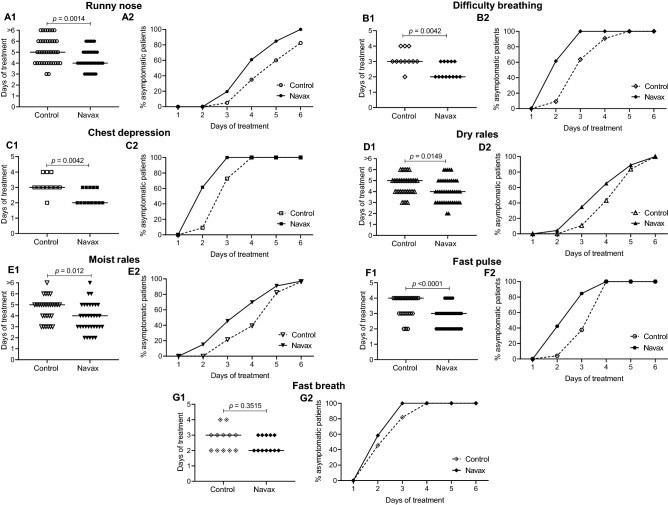
Days of treatment (**A1**–**G1**) and time-dependent percentage (%) of asymptomatics patients (**A2**–**G2**) for observation of seven typical symptoms of ARTIs related to RSV infection in the Control (dashed lines) and Navax (solid lines) groups. More than six days of treatment refers to symptoms that have not improved by day 6. Methods to test distribution was verified by Mann–Whitney test.

To examine the effectiveness of LiveSpo Navax, we evaluated typical clinical features of RSV infection in patients at day 3 and day 6 and observed notable differences between the Navax and the Control groups (Table 1). In comparison to the Control group, the Navax group showed a lower percentage of patients who still had symptoms like moist rales (32.61% in Navax vs. 55.00% in Control; *p* = 0.04), and fast pulse (8.70% in Navax vs. 30.00% in Control; *p* = 0.02) at day 3. At day 6, there was no patient with runny nose in Navax group vs. 17.50% in Control (*p* = 0.04). Because most patients had recovered by the third day of treatment, there was no statistically significant difference in either fever, pulse oxymetry, or fast breath between the two groups. The median days of treatment and changes in percentage (%) of patient’s symptoms free over time in the two groups were then analyzed and shown in Fig. 3A1–G1 and A2–G2, respectively. Remarkably, the Navax group recovered from most of the symptoms, including runny nose (day 4 in Navax vs. day 5 in Control; *p* = 0.0014; Fig. 3A1), difficulty breathing (day 2 in Navax vs. day 3 in Control; *p* = 0.0042; Fig. 3B1), chest depression (day 2 in Navax vs. day 3 in Control; *p* = 0.0042; Fig. 3C1), dry and moist rales (day 4 in Navax vs. day 5 in Control; *p* = 0.0149 and *p* = 0.012, respectively; Fig. 3D1–E1), fast pulse (day 3 in Navax vs. day 4 in Control; *p* < 0.0001; Fig. 3F1). Although the Navax group's fast breathing symptom was recovered 1-day earlier than that of the Control group, the difference is not statistically significant (*p* = 0.3515) (Fig. 3G1). We also discovered that in the Navax group, the Days of Treatment when 50% patients are no longer symptomatic (DT_50_) for all observed seven symptoms were shorter than in the Control group. In details, pairwise comparison between the Navax group vs. the Control group demonstrated that DT_50_ in Navax group was shorten for multiple clinical indicators: (i) runny nose by 1.2-fold (3.74 in Navax vs. 4.60 in Control; Fig. 3A2); (ii) for difficulty breathing by 1.3-fold (2.17 in Navax vs. 2.75 in Control; Fig. 3B2); (iii) for chest depression by 1.2-fold (2.17 in Navax vs. 2.64 in Control; Fig. 3C2); (iv) for dry rales by 1.2-fold (3.50 in Navax vs. 4.17 in Control; Fig. 3D2); (v) for moist rales by 1.3-fold (3.19 in Navax vs. 4.25 in Control; Fig. 3E2); (vi) for fast pulse by 1.5-fold (2.18 in Navax vs. 3.20 in Control; Fig. 3F2), (vii) and for fast breath symptoms by 1.1-fold (1.85 in Navax vs. 2.13 in Control; Fig. 3G2). This data indicated that Navax helped to increase the efficiency of symptomatic treatment by 10–50% with chest depression being the most improved. Taken all together, we concluded that spraying *Bacillus* spores alleviates normal RSV-infection symptoms 1-day early and 10–50% more effectively.

### Lowering RSV load and co-infection bacterial concentration by nasal-spraying *Bacillus* spores

**Figure 4:**
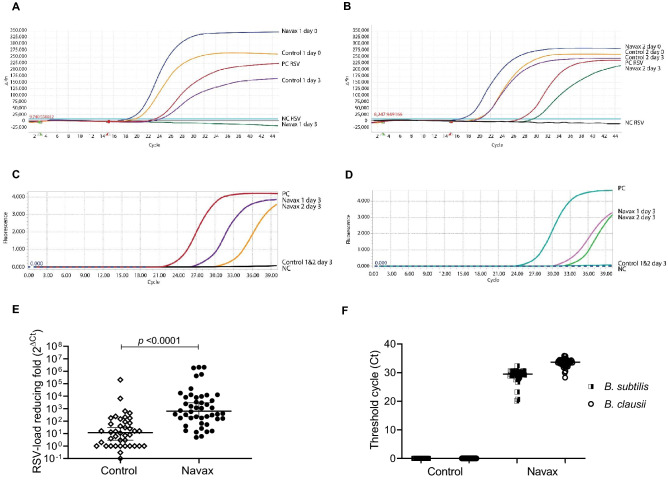
Real-time PCR TaqMan probes amplification curves (**A**, **B**) specifically for RSV taken from two representative nasopharyngeal samples of Control (Control 1_code N74, Control 2_code N66) and Navax groups (Navax 1_code N58, Navax 2_code N43) at day 0 and 3 of treatment. PC, NC are positive and negative controls of RSV; real-time PCR SYBR Green amplification curves specifically for *B. subtilis* (**C**) and *B. clausii* (**D**) taken from two representative nasopharyngeal samples of Control (Control 1, 2) and Navax (Navax 1, 2) groups at day 3 of treatment. PC, NC are positive and negative controls of *B. subtilis* and *B. clausii*; Reducing-fold levels (2^△Ct^) of RSV load in nasopharyngeal samples of Control and Navax groups at day 3 compared to day 0 (**E**). The Mann–Whitney test was used to calculate the median difference in viral load reduction in the two groups; Threshold cycle (C_t_) of fluorescent signals for *B. subtilis* and *B. clausii* measured in nasopharyngeal samples of Control and Navax groups at day 3 (**F**).

Next, to determine how *Bacillus* spores act to relieve symptoms, we conducted real-time PCR TaqMan probes to measure RSV loads in nasopharyngeal samples. The changes in RSV loads and co-infection bacteria concentrations were semi-quantified using 2^△Ct^ values where △C_t_ is the difference between threshold cycle (C_t_) at day 3 and C_t_ at day 0. Day 3 was chosen since it was noted that the benefit of LiveSpo Navax treatment in improving symptoms in RSV-infected patients was highest at this time point. As shown in Fig. 4A–B, two representative amplification curves taken from two representative nasopharyngeal samples of each group demonstrated that RSV load in both groups was much lower (or not detectable) at day 3 compared to before treatment. Amplification curves with similar results were seen in other samples of both groups (Figs. S1–S2). Moreover, the 2^△Ct^ values showed that RSV viral load in the Navax group was reduced by more than 630-fold, compared to 12-fold in the Control group (Fig. 4E). Thus, nasal-spraying *Bacillus* spores has improved efficacy of RSV load clearance by 53 folds. To independently validate that the control 0.9% NaCl physiological saline vs. LiveSpo Navax were used correctly in the two experimental groups, we examined the presence of *B. subtilis* and *B. clausii* in nasopharyngeal samples at day 3 by real-time PCR SYBR Green detection. We found that while there was no detectable signal in the Control group, fluorescent signals corresponding to amplifications of *B. subtilis* and *B. clausii* genomic DNA were readily detected in the Navax group (representative data- Fig. 4C–D and Fig. S1-2). In fact, amplification thresholds for *B. subtilis* and *B. clausii* in the Navax group were at C_t_ values (median, lower–upper confidence limit) of 29.6 (28.9–30.1) and 33.6 (33.1–33.9), respectively, and non-detectable in the Control group (Fig. 4F). The data confirmed the correct administration of LiveSpo Navax and the control product in our trial.

 Given that bacterial co-infection in nasal tract is common in patients with RSV infection and is the cause of complication, we developed a multiplex real-time PCR assay to evaluate bacterial co-infection. As shown in Table 3, ten individuals in each group who were tested positive with *S. pneumoniae* and *H. influenzae* with C_t_ values of < 30 at day 0 were further tested at day 3. Concentrations below this threshold cycle do not pose a high risk of bacterial co-infection in the respiratory tracts and do not necessitate the use of antibiotics in routine hospital care. Surprisingly, we observed that 9 out of 10 patients in Navax group infected with *S. pneumoniae* (C_t_ range: 25–29) or/and *H. influenzae* (C_t_ range: 26–29) at day 0 became negative (C_t_ > 40) at day 3, meaning that the reducing folds (2^△Ct^) in these cases were more than 10^3^–10^4^. The remained one case had unchanged *H. influenzae* concentration. By contrast, there were only 3 out of 10 patients in the Control group infected with either *S. pneumoniae* or *H. influenzae* showed a negative result at day 3. Meanwhile, 3 cases had 3.5–42-fold decrease; 1 case was unchanged; and 2 cases showed 4.0–6.0-fold increase. Comparing the number of negative vs. positive results obtained after 3 days of treatment between Navax and Control, the difference was statistically significant (OR_Navax/Control_ = 21; *p* = 0.0157).

**Table 3:** Fold-changes in co-infection bacteria’s concentrations in nasal tract of Navax and Control group after 3 days of treatment.

Patient Code	Bacterial species	Day 0 (Ct)	Day 3 (Ct)	Reducing fold (2^△Ct^)	Patient code	Bacterial species	Day 0 (Ct)	Day 3 (Ct)	Reducing fold (2^△Ct^)
**Control group**					**Navax group**				
C1	*H influenzae*	28.5	26.0	+ 6.0^a^	N1	*S. pneumoniae*	29.0	Negative^b^	> 10^3^
C2	*H influenzae*	24.0	29.4	42	N2	*S. pneumoniae*	25.2	Negative^b^	> 10^4^
C3	*H influenzae*	26.0	30.0	16	N3	*S. pneumoniae*	27.0	Negative^b^	> 10^3^
C4	*S. pneumoniae*	25.0	23.0	+ 4.0^a^	N4	*S. pneumoniae*	25.2	Negative^b^	> 10^4^
C5	*S. pneumoniae*	25.0	Negative^b^	> 10^4^	N5	*S. pneumoniae*	26.0	Negative^b^	> 10^4^
C6	*H influenzae*	25.9	Negative^b^	> 10^4^	N6	*S. pneumoniae*	27.0	Negative^b^	> 10^3^
C7	*H influenzae*	23.0	Negative^b^	> 10^5^	N7	*S. pneumoniae*	26.0	Negative^b^	> 10^4^
C8	*S. pneumoniae*	27.0	27.0	Unchanged	N8	*S. pneumoniae*	27.0	Negative^b^	> 10^3^
						*H. influenzae*	29.0	Negative^b^	> 10^3^
C9	*S. pneumoniae*	28.0	26.0	+ 4.0^a^	N9	*H. influenzae*	26.2	Negative^b^	> 10^4^
C10	*S. pneumoniae*	28.0	29.8	3.5	N10	*H. influenzae*	29.0	28.7	Unchanged

+ ^a^: Increasing fold but not reducing fold.

Negative^b^: SYBR Green fluorescent signal is not detectable or equivalent to C_t_ > 40.

### Immunomodulatory activities of nasal-spraying *Bacillus* spores

**Figure 5:**
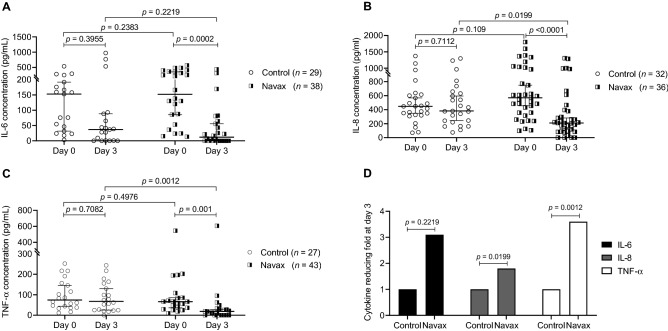
Pro-inflammatory cytokines levels (pg/mL) in nasopharyngeal samples of Control and Navax groups at day 3 compared to day 0. The Wilcoxon test was used to calculate the median differences in (i) IL-6 (**A**), IL-8 (**B**), TNF-α (**C**) levels at day 0 and day 3 in each group. The Mann–Whitney test was used to compared cytokine concentrations (**A**–**C**) and cytokine reducing folds (**D**) between the two groups. Only samples with measurable cytokine concentrations at day 0 were included in the statistical analysis. 95% CI for median in each group and the median difference between the two groups were shown in (**A**–**C**). The significance level of all analyzes was set at the *p* < 0.05.

Next, to investigate the relevance of immunomodulatory activities of Navax, we evaluated changes in levels of common pro-inflammatory cytokines including IL-6, IL-8 and TNF-α after 3 days of treatment. At day 0, the levels of IL-6, IL-8 and TNF-α in both groups were high and represented with no statistical difference (*p* = 0.2383, *p* = 0.109, *p* = 0.4976, respectively). At day 3 of LiveSpo Navax treatment, we observed that IL-6, IL-8 and TNF-α levels were remarkably reduced by 12.7-fold (*p* = 0. 0002), 2.7-fold (*p* < 0.0001), and 3.5-fold (*p* = 0.001), respectively (day 3 vs. day 0: IL-6: 12.0 pg/mL vs.152.3 pg/mL, IL-8: 210.2 pg/mL vs. 569.3 pg/mL, and TNF-α: 18.91 pg/mL vs. 66.13 pg/mL) (Fig. 5A–C). On the other hand, while the trend in reduction of IL-6, IL-8 and TNF-α levels were also observed in the Control group, the differences were not statistically significant i.e., 4.2-fold (*p* = 0.3955), 1.2-fold (*p* = 0.7112), and 1.1-fold (*p* = 0.7082), respectively (Fig. 5A–C). Thus, IL-6, IL-8, and TNF-α were lowered by 3.1-fold (*p* = 0.2219), 1.8-fold (*p* = 0. 0199), and 3.6-fold (*p* = 0.0012), respectively, when comparing the levels of those cytokines at day 3 between the Navax and the Control groups (Fig. 5D). The data strongly indicated that nasal-spraying *Bacillus* spores effectively suppress cytokine overreacted production in immune response to RSV infection.

### Associations between improvements in clinical symptoms, reduction in RSV load, and reductions in cytokines levels

To uncover the associations between days of treatment for the typical symptoms of ARTIs, reduction in RSV load, and reductions in cytokines levels, we performed Spearman's rank correlation coefficients (rho) analysis (Fig. S3). Regarding the symptoms, there was a monotonic correlation between the relief from chest depression and difficulty breathing (rho =  + 1.00; *p* = 0.001), but a weak correlation between dry rales and runny nose (rho =  + 0.34; *p* = 0.019) in the Navax group. In the Control group, there was a moderate association between dry rales relief and runny nose relief (rho =  + 0.44; *p* = 0.007), but difficulty breathing relief was more strongly connected with dry rales relief (rho =  + 0.79; *p* = 0.010) than with chest depression relief (rho =  + 0.55; *p* = 0.155). Relief from symptoms in patients of Navax group was proportional with reductions in RSV load (rho =  + 0.30 in breath difficulty and chest depression), IL-6 (rho =  + 0.32 in moist rales; rho =  + 0.22 in runny nose), IL-8 levels (rho =  + 0.23 in dry rales; rho =  + 0.17 in runny nose), or in TNF-α levels (rho =  + 0.25 in dry rales; rho =  + 0.24 in fast pulse). However, these correlations were not statistically significant, possibly due to the size of the experimental groups. On the other hand, patients in the Navax group exhibited a stronger association between RSV load and IL-8 level reduction (rho =  + 0.49; *p* = 0.003) than those in the Control group (rho =  + 0.19; *p* = 0.36). Furthermore, in the Navax group, the IL-8 reduction was found to be linked with the IL-6 reduction (rho =  + 0.48; *p* = 0.02), whereas this proportional connection (rho =  + 0.38; *p* = 0.121) was lower and not statistically significant in the Control group. Overall, there were notable correlations between improvements in several major clinical symptoms, reduction in RSV load, and cytokines levels, suggesting a potential mechanism of action for nasal-spraying *Bacillus* spores in lowering viral load and modulating immune response.

## Discussion

Safe *Bacillus* species like *B. subtilis*, *B. clausii,* and *B. coagulans* can retain their efficacy for a long time because they can form heat-stable spores under harsh conditions such as starvation^24,34–37^. *Bacillus* spore probiotics can be made as a liquid suspension with extremely pure and concentrated spores for direct spraying into the nasal tract. In this clinical trial with a moderate number of 86 patients (40 in Control vs. 46 in Navax), the data showed that LiveSpo Navax was safe for all participants. Correct administration of LiveSpo Navax provided 1-day earlier and 10–50% more effective symptomatic-relief including runny nose (*p* = 0.0014), chest depression (*p* = 0.0042), difficulty breathing (*p* = 0.0042), dry rales (*p* = 0.0149), moist rales (*p* = 0.012), fast pulse (*p* < 0.0001), and possibly fast breath (*p* = 0.3515). All patients in the Navax group were fully recovered during 6-day follow up, while several patients in the Control group still had runny nose (8/40 cases, 26.67%) and dry rales (3/40 cases, 10%) at day 6. These findings formed a foundation for future clinical trials with a larger sample size to further evaluate the effects of nasal-spraying *Bacillus* spores in symptomatic treatment for children with RSV and other viral infection.

The ability of LiveSpo Navax in shortening one day treatment while improving 10–50% efficacy is beneficial not only to the children’s health but also alleviate parents' anxiety and caring time while lowering the cost of therapy and hospitalization. Our data indicated a faster and superior efficacy in supportive treatment of ARTIs using nasal-spray *Bacillus* spore probiotics when compared to previous results using oral administrative ones. In the clinical study in 80 healthy children aged 6–8 years to assess the effects of oral *Bacillus coagulans* GBI-30, 6086 probiotics against upper respiratory tract infections (URTIs) after up to three months usage, there is a lower incidence of URTI symptoms such as nasal congestion, bloody nasal mucus, itchy nose, and hoarseness, as well as a shorter duration of URTI-related symptoms such as hoarseness, headache, red eyes, and fatigue^31^. In another study, Slivnik et al. 2020 have conducted a clinical trial in children aged 2–6 years, which looked at the effects of *B. subtilis* DE111 probiotics on gastrointestinal tract infections and URTIs. Even after long observation period (up to 102 days), despite the gastrointestinal efficacy, there was no change in the incidence of respiratory infection (41.3% probiotic vs. 36.2% placebo, *p* = 0.60)^38^.

Results from real time PCR assays revealed that nasal-spraying *Bacillus* spores reduced RSV viral load with a remarkable efficacy of over 53-fold more effective than standard of care treatment. This data also provided a mechanistic insight underlying the benefit of *Bacillus* spore probiotics in relieving typical symptoms due to RSV infection. These findings are in agreement with prior studies that looked at the impact of nasal-dropping *Bacillus* spores on viral infection in mice. Song et al. (2012) have found that *B. subtilis* PY79 spores can non-specifically adsorb H5N1 influenza virus at an estimated 8 virions per one spore, and that the complex virus-spore suspension can function as a nasal mucosal vaccine with 80% protection of mice against H5N1 viral challenge infection at 5LD_50_ dosage^39^. Hong et al. (2019) have also shown that nasal drops of *B. subtilis* spores have lowered 4–5 folds RSV levels in mice by improving the antiviral function of alveolar macrophages^40^.

Secondary infections caused by *S. pneumoniae, H. influenzae, S. aureus,* and *M. catarrhalis* have been linked to RSV infection in children. Co-infection with these pathogens worsens the respiratory failure, lengthens the treatment period, and raises the cost of antibiotic treatment^41^. Antibiotic resistance represents a rising problem in patients infected with *S. pneumoniae* and *H. influenzae*^5,42^. In our study, bacterial co-infection cases accounted for 50–60% of RSV-infected patients, which is similar to recent reports^41,43–45^. The primary data from semi-quantitative real-time PCR of co-infection bacteria in both groups (*n* = 10/group) showed that 9/10 probiotic-treated patients in the Navax group became negative at day 3 of treatment, while unclear reduction in bacterial concentration was observed in 7/10 patients in the Control group. The data suggests that *Bacillus* spores sprayed directly into the nose can block co-infection bacteria's growth far more efficiently than physiological saline. To the best of our knowledge, this is the first study to demonstrate the efficacy of probiotics in reduction of bacterial concentrations in nasal tract.

We hypothesized that sprayed *Bacillus* spores can compete with RSV in interacting with nasal epithelium, resulting in a less systemic inflammatory response syndrome. Mechanistically, our data suggested that *Bacillus* spore spraying could diminish "cytokine storm" in the nasal cavity of RSV-infected patients by regulating the cell mediated immune system. Given the limited material of the nasopharyngeal samples, the most relevant cytokines IL-6, IL-8 and TNF-α in respiratory airway, which were reported as potential biomarkers of severity and prognosis for RSV infection, were chosen in our study^7,8,10^. We found that the IL-8 level detected in nasopharyngeal samples at day 0 was the highest, followed by IL-6 level and the lowest was TNF-α level. The correlation of these three cytokines levels in nasal fluid of RSV-infected children is similar to a recent study by Garcia et al.^46^. After 3 days of treatment, levels of IL-6, IL-8, and TNF-α in nasopharyngeal samples of patients in Navax group were about 2.5-fold more effective than those in the Control group. The IL-6 level (12.0 pg/mL) measured in nasopharyngeal samples of Navax group at day 3 was close to those (14.9–16.6 pg/mL) measured in intravenous blood of healthy children reported recently^47,48^. T-helper responses are critical in stimulating cytotoxic T lymphocyte (CTL) proliferation, as well as the production of cytokines^39^. IL-6, along with IL-1, IL-2, IL-8 and TNF-α, play important role in modulating the host immune response during viral infection^49^. In infants with acute RSV infection, inflammation in the upper and lower airways is dominated by an intense neutrophilia, with overreacted release of pro-inflammatory cytokines such as IL-6, IL-8, and TNF-α produced in response to RSV^11^. A number of in-vitro and animal studies have shown that certain probiotic strains can protect against virus infections by triggering or modulating cytokine responses in respiratory epithelial cells or immune cells^22,26^. Therefore, our data support the notion that while cell mediated immunity does not specially prevent viral infection, it can aid the recovery of infection-related-consequences.

Notably, there is statistically significant correlation between reduction of RSV load and decreased levels of IL-8, IL-6 and TNF-α in Navax group. One possible mode of action for *Bacillus* spores is the ability of ligands on their outer coat layer to adsorb RSV virions while simultaneously competing with the viruses for interaction with nasal epithelium. This results in reducing activation of the innate immune system in the nasal-associated lymphoid tissue, hence modulating the hyper-induction of pro-inflammatory cytokines. Due to ethical concerns, limited nasopharyngeal samples were collected only once for each time point, which is sufficient only for measuring the loads of RSV, co-infection bacteria, *Bacillus* spores by real-time RT/PCR, and concentrations of the three cytokines by conventional ELISA method. Therefore, other pro-inflammatory cytokines with potential prognostic value in RSV infection, such as IL-3 and IL-12 (Th1-type cytokines), IL-13 and IL-33 (Th2-type cytokines)^10^, were not included in the evaluation. The procedure thus limited the measurement of other immuno-stimulatory parameters reflecting activation of macrophages, maturation of dendritic cells (DC), recruitment of natural killer (NK) cells, and other cytokines relating to Th1/Th2 balance. In the future, by applying Luminex XMAP technology for measuring multiple cytokines in a single test, changes in key cytokines relating to multiple pathways that regulate immune cell proliferation and differentiation following RSV infection will provide us with more insights on mechanisms of action for nasal-spraying *Bacillus* spores against RSV infection.

Since the mechanism of interaction between *Bacillus* spores with nasal mucosal immune system, virus and bacteria relies on non-specific interaction, our findings suggest that nasal-spraying *Bacillus* spores could be useful not only for supportive treatment of RSV infection, but also for other rapidly emerging RTIs virus like influenza virus or coronavirus. People who have been vaccinated via the intramuscular route are at risk of ARTIs due to either low in-situ antibody levels in the nasal tract or appearance of new immunity-escape mutations. Nasal-spraying *Bacillus* spores can provide an additional barrier of protection to help increase the effectiveness of preventing viral infection in general. This novel probiotic treatment has a distinct advantage of being simple to use, low-cost, and effective against viral infection, making it ideal for developing countries with limited medical resources. Future clinical trials of nasal-spraying *Bacillus* spores with these viruses, if successful, could pave the way for the prevention and supportive treatment of viral infectious respiratory diseases in children for which there is current no vaccination, vaccination with low protection level, or specific drug therapy.

## Conclusion

This study is the first randomized and blind clinical trial in RSV-infected children to demonstrate safety and beneficial effects of a nasal-spraying liquid suspension of *Bacillus* spore probiotics in relieving clinical respiratory symptoms of RSV infection, thereby shortening the treatment period 1 day and improving symptomatic treatment efficacy by 10–50%. The sprayed *Bacillus* spores significantly inhibited the multiplications of RSV (630 folds) and co-infection bacteria *S. pneumoniae* and *H. influenzae* (10^3^–10^4^ folds), thereby reducing the overreacted immune response of epithelium cells to lower IL-6, IL-8, TNF-α pro-inflammatory cytokine levels (2.7–12.7 folds) in nasal tract.

## Methods

### Materials

Nasal-spraying probiotics LiveSpo Navax (LiveSpo Pharma, Hanoi, Vietnam) was formulated as a 0.9% NaCl physiological saline suspension containing *Bacillus subtilis* ANA4 (accession no. MT123906.1 in NCBI) and *B. clausii* ANA39 (accession no. MT275656.1 in NCBI) spores at a total concentration of > 5 × 10^9^ CFU/ 5 mL. LiveSpo Navax was manufactured as a Class-A medical device product (Product declaration approval 210001337/PCBA-HN) under manufacturing standards approved by Hanoi Health Department, Ministry of Health, Vietnam (Certificate No YT117-19) and ISO 13485:2016. Summarized data of (i) microbial and biochemical characterization (Table S1), (ii) antibiotics susceptibility (Table S2), (iii) 16S rRNA sequencing analysis (Figs. S4–S6), and (iv) sequence analysis of antibiotic resistance and toxic genes in *B. subtilis* ANA4 and *B. clausii* ANA39 whole genomes (Table S3–S5) were shown in Supplemental data, indicating that the two strains are safe in-vitro tests, have high spore formulation efficiency (> 90%), heat-stability (> 65 °C), and survive in both aerobic/anerobic conditions. These advantage properties are suitable for low-cost production of spore suspension in physiological saline NaCl 0.9%, stable quality during storage at room temperature, and being alive to exhibit their effects in nasal tract. Acute and sub-acute toxicity studies in mice and rabbit for LiveSpo Navax conducted in Vietnam National Drug Quality Control Institute have indicated that the product is non-toxic. LiveSpo Navax (intervention product) and 0.9% NaCl physiological saline (control product) were indistinguishable regarding taste and smell. The color and turbidity of LiveSpo Navax suspension is unrecognizable to nurses and patient's parents due to opaque plastic spray bottle. Nasal-spraying 0.9% NaCl physiological saline (control product) was prepared by extracting 5 mL from 0.9% NaCl intravenous infusion 500 mL PP bottle (B. Braun, Germany), and then transferring it into the opaque plastic spray bottle that is used for LiveSpo Navax. The control and intervention products were coded number 1 and 2, respectively, and this coding information was blind to most investigators and nurses (except the PI, the data analyst, and the chief nurse who is responsible for product preparation and coding) and to all parents of children.

### Ethical issues

This study received ethics approval by the Ethics Committee in Medical Research of the Vietnam National Children’s Hospital under Decision No. 1266 / BVNTW-VNCSKTE on 28 August 2020. The study was conducted in accordance with the ethical principles in accordance with the Helsinki statement and the ICH GCP guidelines, in accordance with Vietnam Ministry of Health's current ethical regulations and standards on research on subject’s human. All parents of pediatric patients participating in the study were provided with information about the study and agreed to sign the informed consent form to participate in the study. Participants are free to withdraw from participation in the study at any time upon request. The study was registered with ClinicalTrials.gov of US. National Library of Medicine (Identifier No: NCT05164692) on 21/12/2021.

### Study design and patient collection

This was a blind, randomized, controlled trial, with the Control group used 0.9% NaCl physiological saline and an experimental group (named the “Navax” group) used the probiotics LiveSpo Navax. The study lasted for 12 months, from August 2020 to August 2021, and included pediatric patients (both male and female sex) having acute respiratory infection symptoms and were diagnosed with positive RSV infection at International Center, Vietnam National Children’s Hospital. Sample size was calculated based on a hypothesis is that LiveSpo Navax alleviates RSV-infection symptoms about 25% more effectively, as indicated by 90% of patients in the Navax group are symptom free at day 3–6 of intervention, compared to 65% of patients in the Control group. Estimated required sample size for each group was 43 at the end of intervention (α = 0.05; power level = 0.8). Thus, sample size at the beginning of intervention was decided to be 50 per each group to reduce the risk of patient’s drop out during follow-up treatment. Totally 100 eligible RSV-infected participants were randomly assigned by lottery to the Control and the Navax groups. In detail, the chief nurse randomly took paper sheets coding either number 1 or 2 from a box and assigned the coding number to each participant right after his/her parents signed the informed consent form. The numbers 1 and 2 were assigned to the Control and the Navax groups, respectively, and this information was also blind to all parents of children, nurses, and investigators, except the PI and the data analyst. The flow charge of study was shown in Fig. 1.

Inclusion criteria were:

1. Children (male/female) aged from 4 to 60 months.
2. Admitted hospital due to lower respiratory infection.
3. RSV positive by rapid test.
4. Parents of the pediatric patient agreed to participate in the study, explained and signed the research informed consent form.

Exclusion criteria included:

1. Newborn babies
2. Have history of drug allergy
3. Need oxygen therapy
4. Discharged before day 3
5. Lost to follow-up
6. Withdrawn from trial
7. Continuing in trial but missing data
8. Meeting the criteria for psychiatric disorders other than depression and/or anxiety.

### Questionnaires, treatment procedures, and clinical observation

The patient’s parents were required to provide the following information of their children: full name, sex, age, obstetric history, vaccination history, antibiotic use history, underlying diseases. The patient was given a coded spray in the form of a blind sample to ensure the objectivity of the study. Nurses were instructed and trained to use sprayers with dosages of 3 presses (each press is about 50 µl 0.9% NaCl physiological saline (with/without 2.5 × 10^8^ *Bacillus* spores)) per each nasal cavity/ time × 3 times/ day directly into the nasal cavity continuously for maximal follow-up 6 days of treatment. The nasal spray products were applied in parallel with routine treatment drugs at hospital such as antipyretics, anti-inflammatory corticosteroids, and aerosol therapy as described in Table 2. Antibiotic susceptibility tests and adequate antibiotic medication were assigned for all patients who tested positive for bacterial co-infection. During treatment, patients were monitored daily for typical clinical symptoms of RSV-induced respiratory tract infections, including runny nose, chest depression, difficulty breathing, dry rales, moist rales, body temperature (°C), pulse oxymetry (SpO_2_) (%), pulse (beats/min), and breath (beats/min) until discharged. The patients’ health conditions were observed by doctors and nurses, and their pieces of information were filled in medical records.

### Routine diagnostics at hospital

Screening of RSV-infected cases from nasopharyngeal samples at day 0 was firstly conducted by using “BD Veritor System for rapid detection of RSV” kit (Becton Dickinson, NJ, US). Detection of respiratory infection bacteria from nasopharyngeal samples was conducted by microbial culture assays on specific mediums. CRP concentrations and white blood cell counts were measured to access the level of infection. Chest radiography was appointed for visualization of lung hyperinflation, atelectasis... Due to ethical concerns, all these tests were conducted only at day 0 following routine procedures at Vietnam National Children’s Hospital.

### Real-time PCR for detection of microorganism in nasopharyngeal samples

Semi-quantitative assays for measuring changes in RSV load and co-infection bacterial concentrations in nasal tract was conducted by the real-time PCR using CFX 96Dx instrument (Bio-Rad Laboratories, CA, USA), which had been standardized under ISO 15189:2012 criteria and routinely used in Vietnam National Children’s Hospital. Nasopharyngeal samples were prepared by rotating a solid plastic handle with breakpoint swab PurFlock Ultra (Puritan, ME, Canada) into nostril straight back (not upwards), one circular path 2 times and keep in place for 5 s, then soak the cotton swab in 1 mL of 0.9% NaCl physiological saline. DNA/RNA from 200 µl specimens (repeated twice) was extracted by MagNA Pure LC Total Nucleic Acid Isolation Kit and MagNA Pure LC 2.0 Instrument (Roche Diagnostics, IA, US) automatic sample extraction system according to the manufacturer's instructions, and 100 µl of the purified DNA/RNA was aliquoted into three PCR tubes (approximately 30 µl/tube) for storage at − 80 °C. Each tube of − 80 °C purified DNA/RNA was thawed only once to take 5 µl for use as the template for each real-time PCR/RT-PCR to detect RSV, *B. subtilis,* and *B. clausii*.

For RSV detection at day 0 and day 3, the following primers (5’→ 3’) and probes (5’→ 3’) were used as described previously^50^: RSV-Fw: TTG GAT CTG CAA TCG CCA; RSV-Rv: CTT TTG ATC TTG TTC ACT TCT CCT TCT at 400 nM each primer; RSV- probe: /56FAM/ TGG CAC TGC/ZEN/TGT ATC TAA GGT CCT GCA CT/3IABkFQ/ at 80 nM; β-actin-Fw: 5’-ATG TCC ACG TCA CAC TTC A; Rnase-Fw: AGA TTT GGA CCT GCG AGC G; Rnase-Rv: GAG CGG CTG TCT CCA CAA GT at 400 nM each primer; Rnase-probe: FAM-TTC TGA CCT GAA GGC TCT GCG CG-BHQ1 at 20 nM. For multiplex real-time PCR detection of co-infection bacteria at day 0 and day 3, the Allplex Respiratory Panel 4 kit (SeeGene, Seoul, Korea) was used to detect simultaneously 07 pathogenic bacterial species, including *Bordetella parapertussis* (BPP), *Bordetella pertussis* (BP), *Chlamydophila pneumoniae* (CP), *Haemophilus influenzae* (HI), *Legionella pneumophila* (LP), *Mycoplasma pneumoniae* (MP), *Streptococcus pneumoniae* (SP). All reactions used an initial denaturation at 95 °C for 10 min, followed by 45 amplification and detection cycles at 50 °C for 2 min, 95 °C for 10 min, amplification for 40 cycles at 95 °C for 15 s, 60 °C for 1 min. The read-out standardization for the analysis of RSV and co-infection bacteria needed to be adjusted to a C_t_ of < 40 to confirm whether they are a true positive or not. The testing procedures was carried out according to ISO 17025:2017 standard and applied routinely at the Department of Analytical Biology of hospitals, National Children's Hospital.

Detection of *B. subtilis* and *B. clausii* ANA39 in nasopharyngeal samples was also conducted at day 0 and day 3 by real-time PCR SYBR Green using primers specifically to *B. subtilis* (Subtilis-F: 5’- ACC ATT GCG GTA GGT GCG -3’; Subtilis-R: 5’- GCG TTT GTC CAA GTC GGG -3’)^51^ and to *B. clausii* (Clausii-F: 5’- AAT TTT TAC CGC CCT CAA G -3’, Clausii-R: 5’- ACT TTT GGA ACA TGC CGA AC -3’) at 400 nM^52^, and using LightCycler 96 instrument (Roche Diagnostics, Mannheim, Germany). Real-time PCR SYBR Green was performed using either LightCycler 96 instrument (Roche Diagnostics, Mannheim, Germany) at following condition: 95 °C for 10 min, amplification for 40 cycles at 95 °C for 15 s, 60 °C for 20 s, 72 °C for 30 s. The read-out standardization for the analysis of *B. subtilis* and *B. clausii* needed to be adjusted to a C_t_ of < 37 to confirm whether they are a true positive or not. The protocol had been standardized under ISO 17025:2017 standard and routinely in the Key Laboratory of Enzyme and Protein Technology, VNU University of Science.

### ELISA assays for cytokine levels

Pro-inflammatory cytokines levels (pg/mL) including interleukin (IL-6, IL-8) and TNF-α in nasopharyngeal samples at day 0 and 3 were quantified using an enzyme-linked immunosorbent assay kit (ELISA) according to the manufacturer’s instructions. IL-6 and TNF-α were quantified from 100 µL samples by the Human IL-6 DuoSet ELISA and the Human TNF-α ELISA kit, respectively (R&D Systems, MN, US). IL-8 was quantified by 50 µL samples by IL-8 Human ELISA kit (Invitrogen/Thermo Fisher Scientific, MA, US). Samples were measured by the SpectraMax Plus384 Microplate Reader system. The results were analyzed by using SoftMax Pro 6.3 Software (Molecular Devices, CA, US). The number of measurable samples for IL-6, IL-8 and TNF-α levels was lower than number of collected patients, both in the Control and the Navax groups was due to (i) lack of sample volume left over from failed and repeated performance in ELISA and real-time PCR assays that occurred at random in each sample; (ii) measured values at day 0 were found below the detection range.

### Data collection and statistical analysis

Individual medical records were collected, and the patient's information was then gathered and systematized in a data set. The efficacy of LiveSpo Navax was evaluated and compared to 0.9% NaCl physiological saline based on following clinical and sub-clinical criteria obtained in the Navax and the Control groups: (i) the symptomatic-relieving day; (ii) the reduction levels (2^△Ct^) of RSV load and co-infection bacteria concentrations. △C_t_ for target genes was calculated as C_t_ (threshold cycle) at day 3—C_t_ at day 0 while C_t_ of internal control were adjusted to be equal among all samples. Negative result or non-detectable signal was assigned to C_t_ of 40; (iii) the reduction levels of IL-6, IL-8, and TNF-α cytokines.

Tabular analysis was performed on dichotomous variables using the Fisher’s exact test when the expected value of any cell was below five (Table 3). Distributions of the data were verified using both the normality test and QQ plot. Variables were compared using either the Wilcoxon test or Mann–Whitney test due to the unnormal distribution of data. The Wilcoxon two-tail test was used on paired samples (Fig. 5A–C for IL6, IL8, and TNF- concentrations at day 3 versus day 0 in each group). The Mann–Whitney test was used on independent samples (Figs. 3, 4E for RSV-load reducing folds at day 3 between the two groups; Fig. 5A–C for IL6, IL8, and TNF- concentrations at either day 0 or day 3 between the two groups; Fig. 5D for IL6, IL8, and TNF- reducing folds at day 3 between the two groups). The correlations among the variables were assessed by Spearman's correlation analysis. Statistical and graphical analysis were performed on GraphPad Prism v8.4.3 software (GraphPad Software, CA, USA). The significance level of all analyzes was set at the *p* < 0.05.

## Supplementary Information

Supplementary Information 1.

Supplementary Information 2.

Supplementary Information 3.

Supplementary Information 4.

## Data Availability

The datasets generated during this current study is available at https://anabio.com.vn/sample/
