# Supplementary material for: Nasal-spraying Bacillus spores as an effective symptomatic treatment for children with acute respiratory syncytial virus infection
Source: Sci Rep. 2022 Jul 20;12:12402. doi: 10.1038/s41598-022-16136-z (PMC9297280; doi:10.1038/s41598-022-16136-z)
Supplement: Supplementary file 1 — Supplementary Information 1. [file 41598_2022_16136_MOESM1_ESM.pdf]

## Supplemental Fig. S1

### Real time PCR curves of RSV, *B. subtilis*, and *B. clausii* of nasopharyngeal samples in Control group

| No | Patient code | A. RSV | B. *B. subtilis* & *B. clausii* |
|---|---|---|---|
| | | | Red —Positive Control (PC) of *B. subtilis*<br>Blue —PC of *B. clausii*<br>Black —Negative control (NC) of *B. subtilis*, NC of *B. clausii*<br>Other colors: samples in Control group with negative signal or $C_t \geq 37$ |
| 1 | N02_D0 | 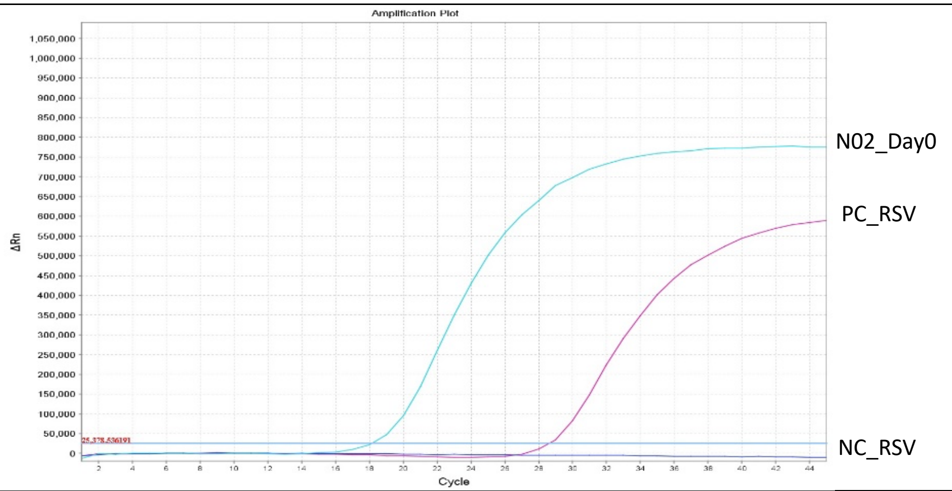 | |
| | N02_D3 | 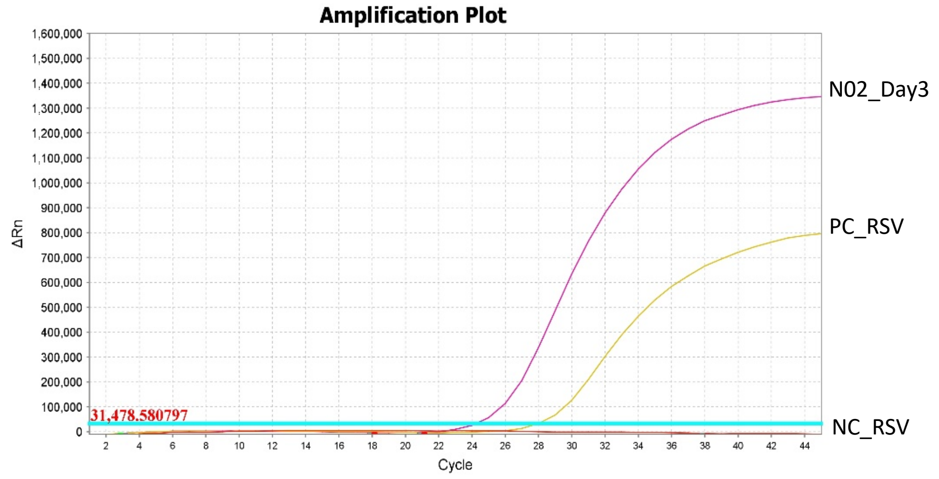 | 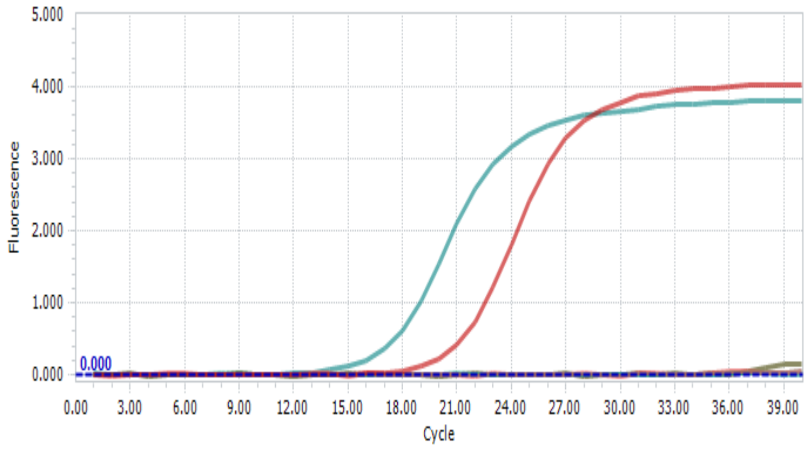 |

<table>
<tr>
<td>2</td>
<td>N04_D0</td>
<td>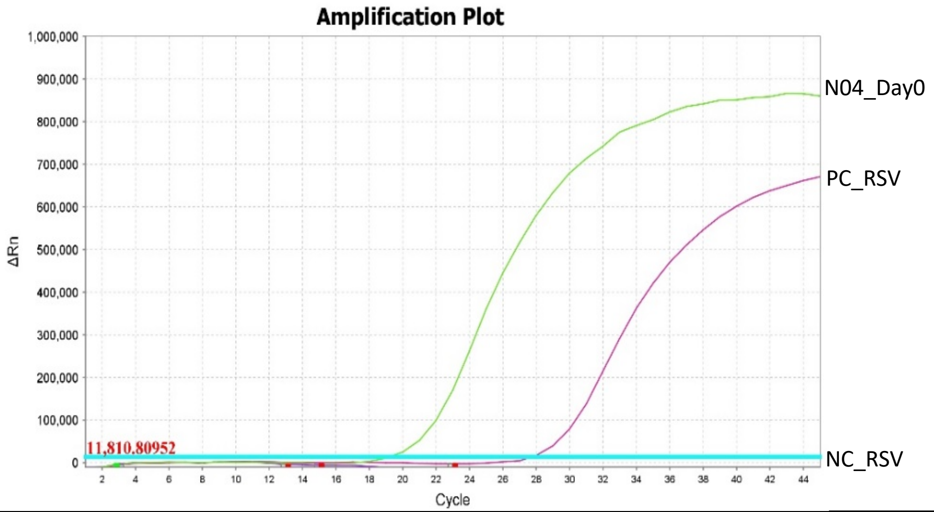
</td>
<td></td>
</tr>
<tr>
<td></td>
<td>N04 _D3</td>
<td>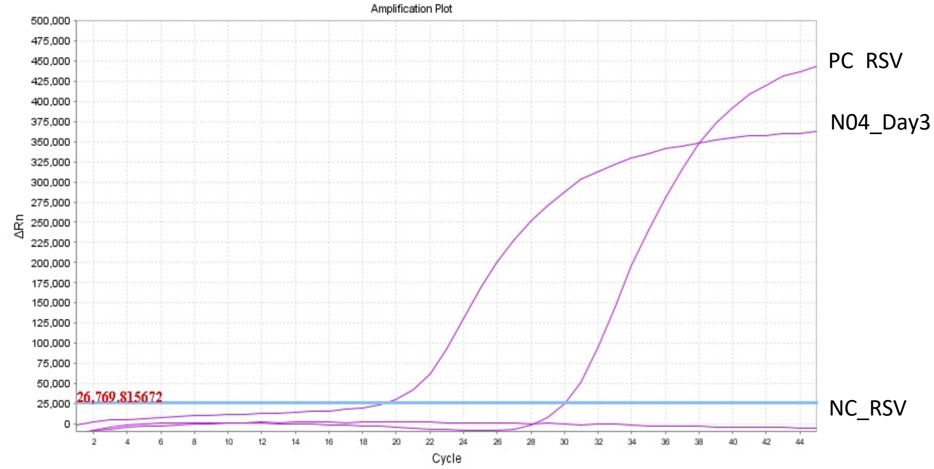
</td>
<td>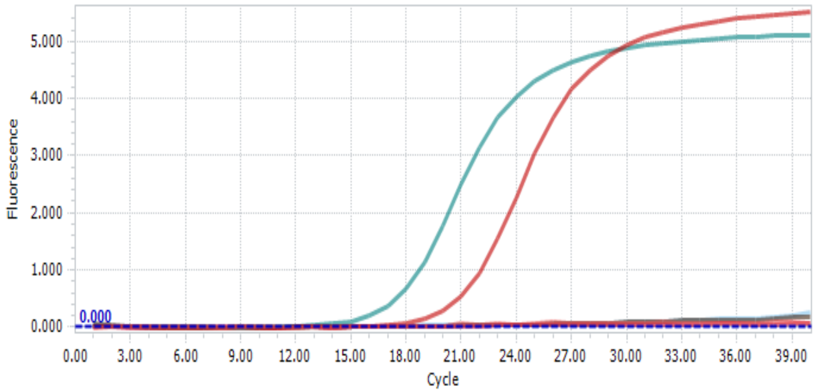
</td>
</tr>
</table>

<table>
<tr><td>3</td><td>N05_D0</td><td>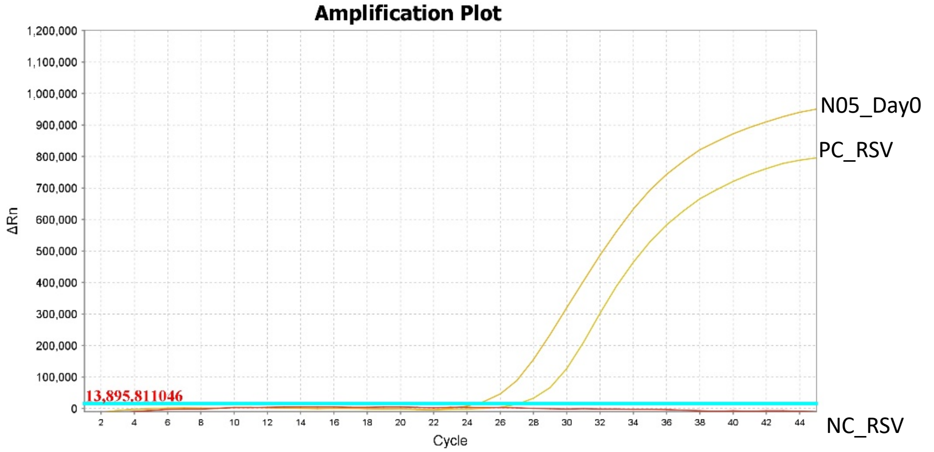
</td><td></td></tr>
<tr><td></td><td>N05_D3</td><td>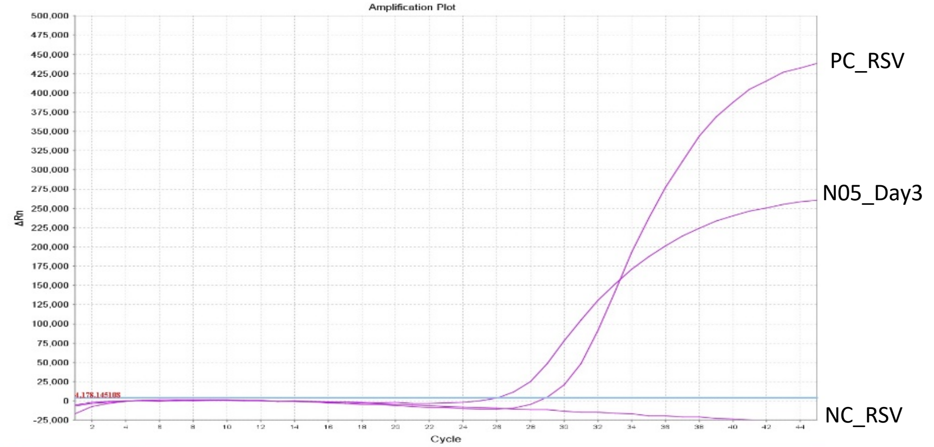
</td><td>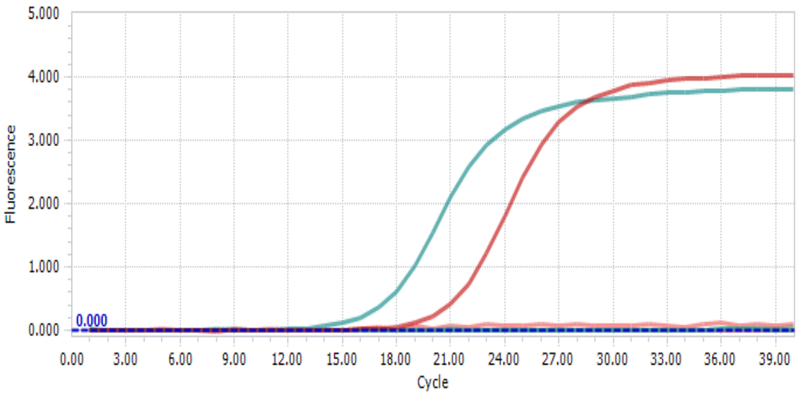
</td></tr>
</table>

<table>
<tr>
<td rowspan="2">4</td>
<td>N08_D0</td>
<td>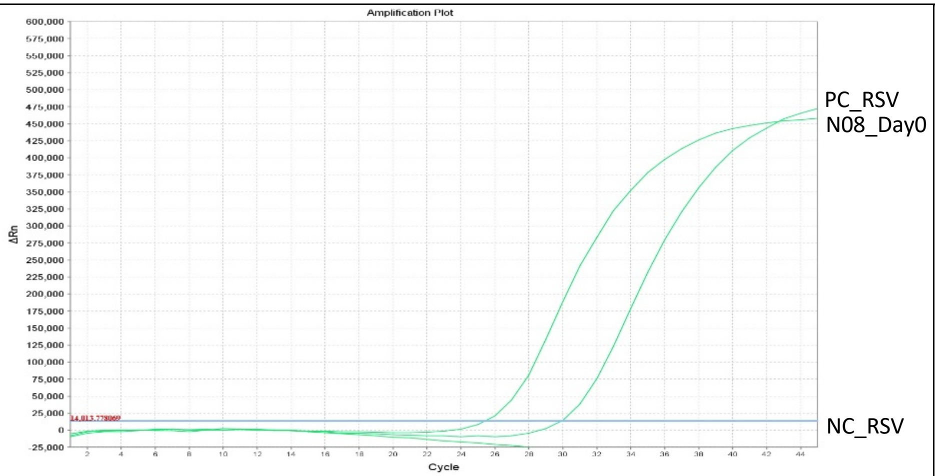
</td>
<td></td>
</tr>
<tr>
<td>N08_D3</td>
<td>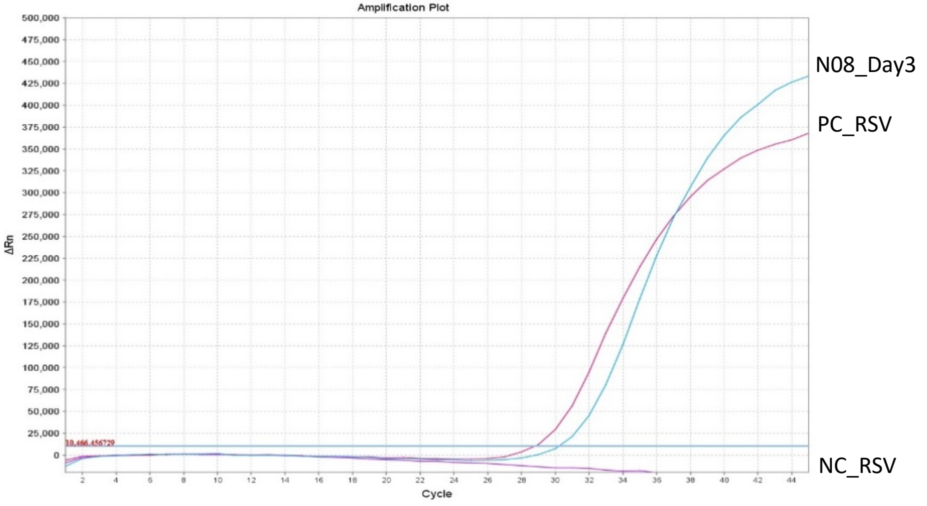
</td>
<td>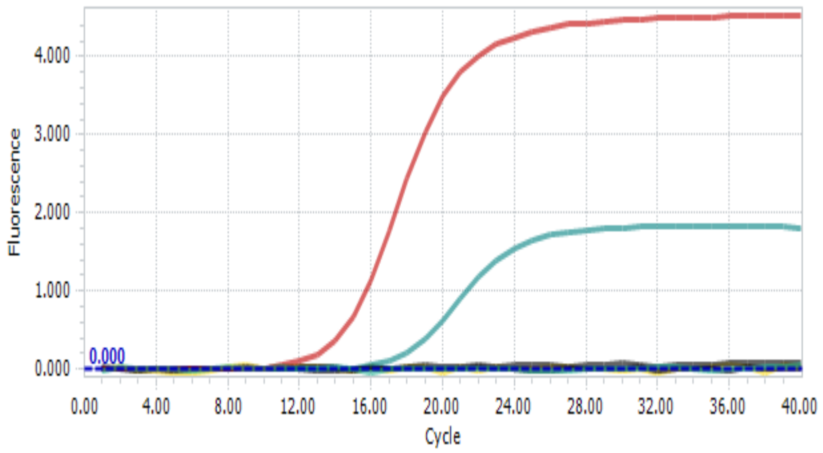
</td>
</tr>
</table>

<table>
<tr>
<td>5</td>
<td>N14_D0</td>
<td>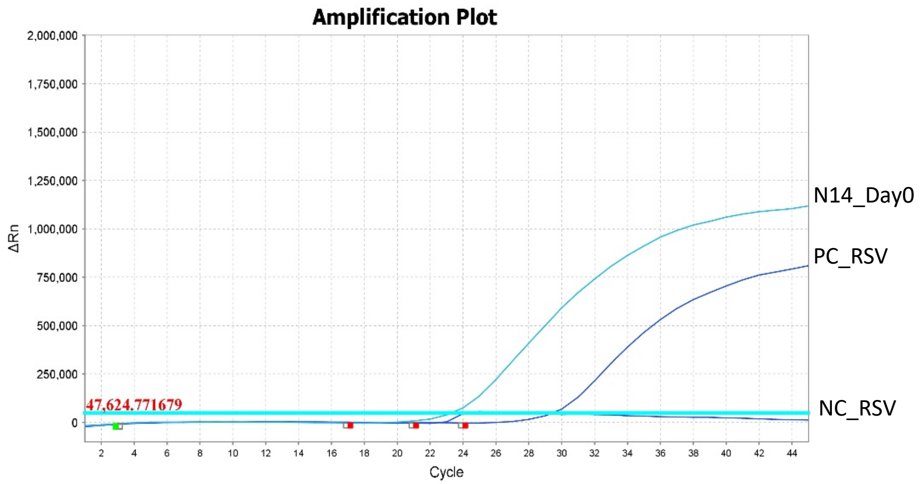
</td>
<td></td>
</tr>
<tr>
<td></td>
<td>N14_D3</td>
<td>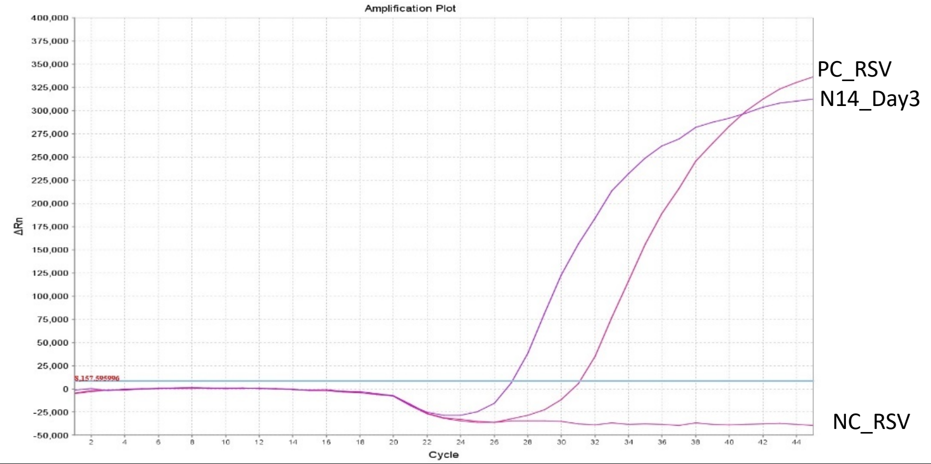
</td>
<td>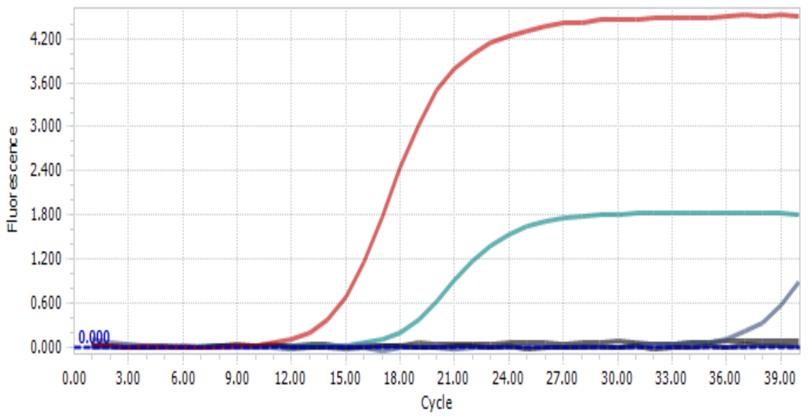
</td>
</tr>
</table>

<table>
<tr><td rowspan="2">6</td><td>N20_D0</td><td>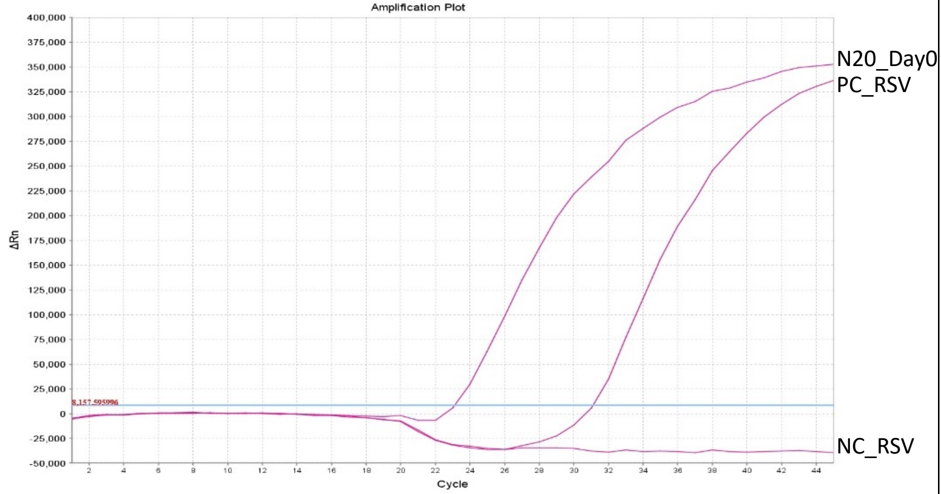
</td><td></td></tr>
<tr><td>N20_D3</td><td>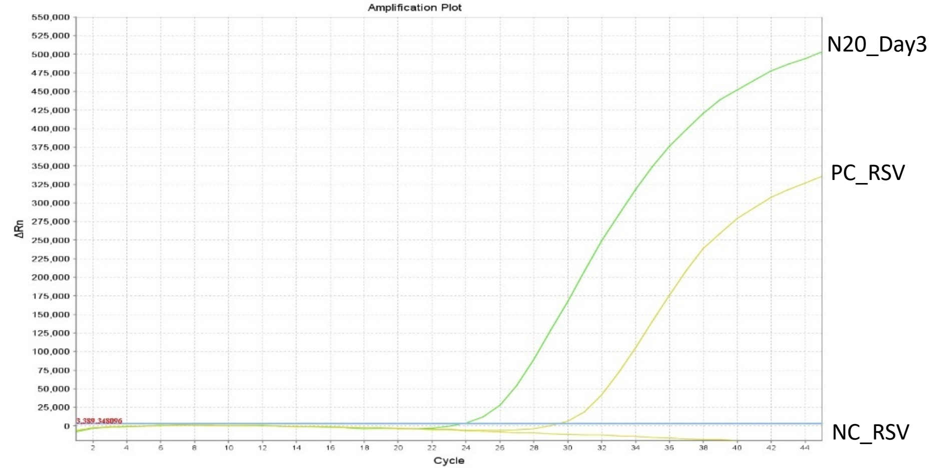
</td><td>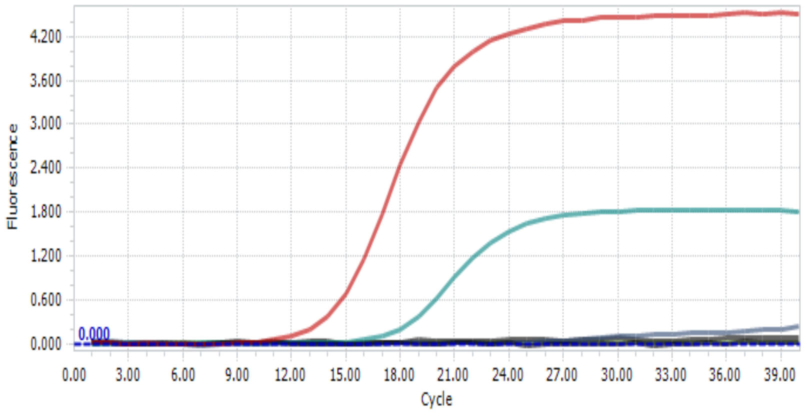
</td></tr>
</table>

<table>
<tr><td rowspan="2">7</td><td>N22_D0</td><td>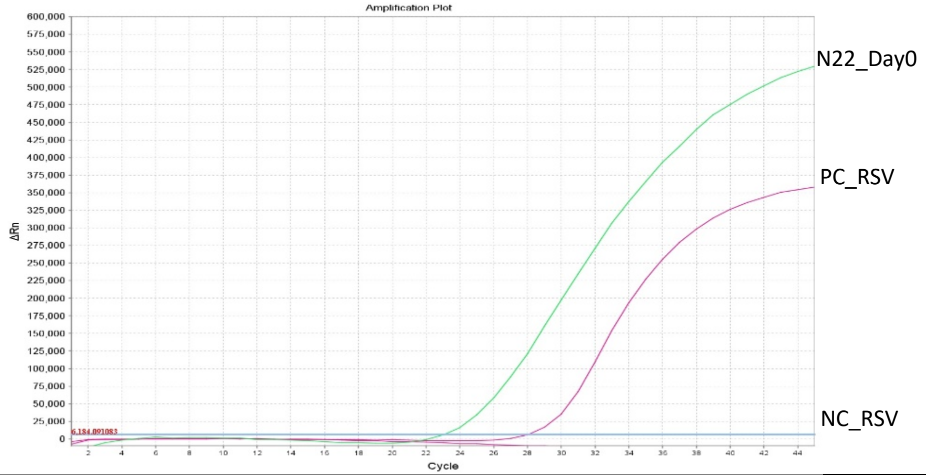
</td><td></td></tr>
<tr><td>N22_D3</td><td>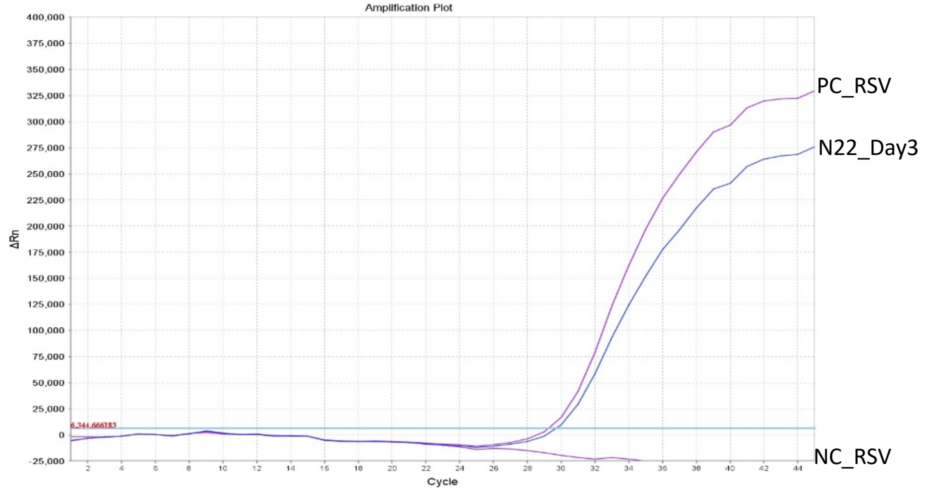
</td><td>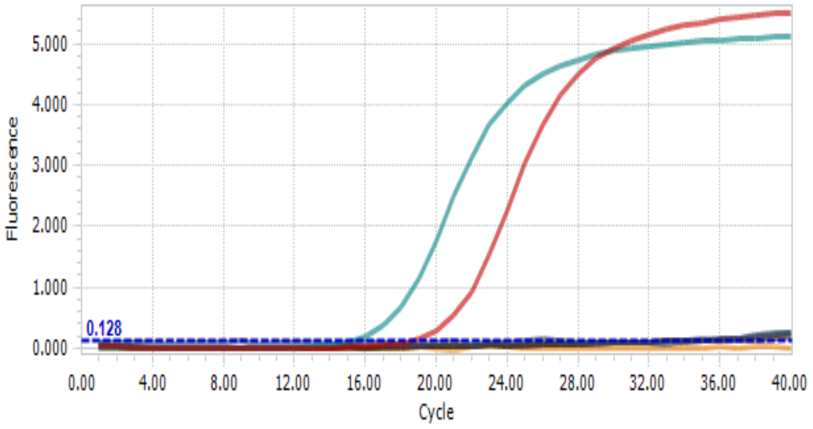
</td></tr>
</table>

<table>
<tr><td rowspan="2">8</td><td>N23_D0</td><td>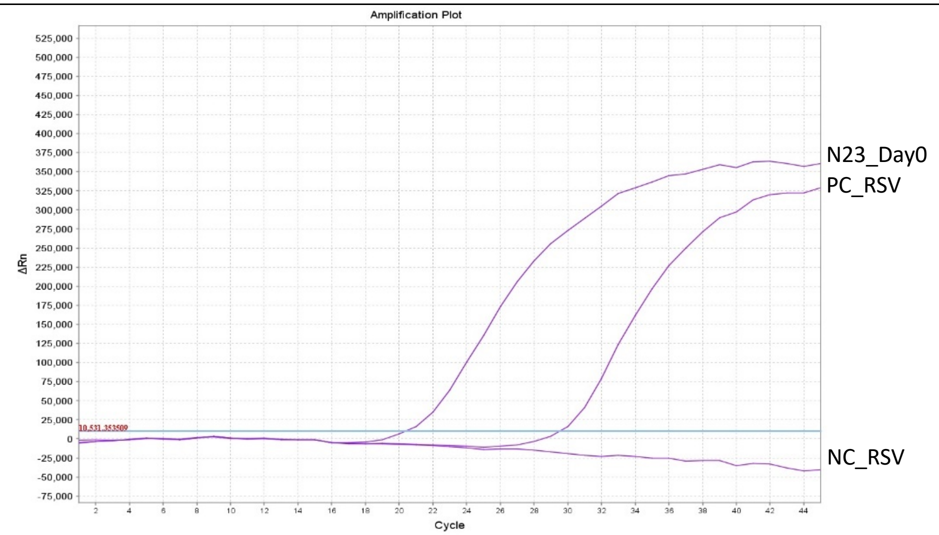
</td><td></td></tr>
<tr><td>N23 _D3</td><td>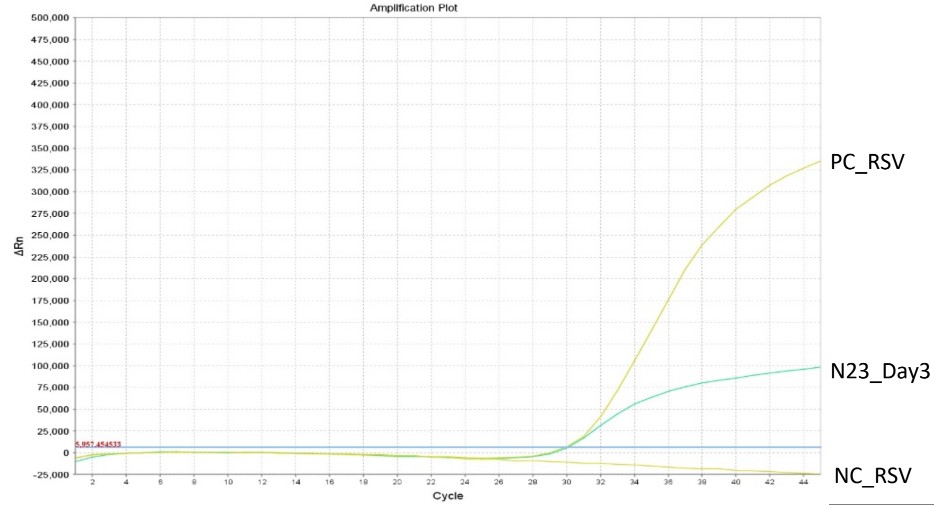
</td><td>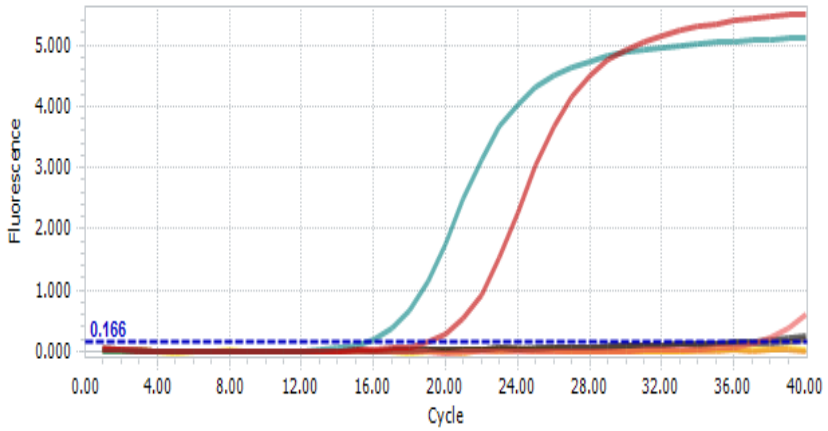
</td></tr>
</table>

| | | | |
|---|---|---|---|
| 9 | N24_D0 | 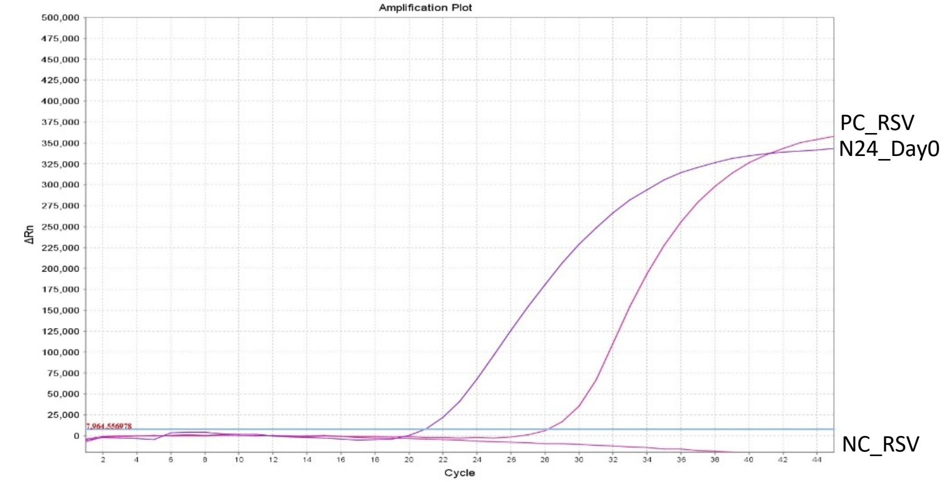 | |
| | N24_D3 | 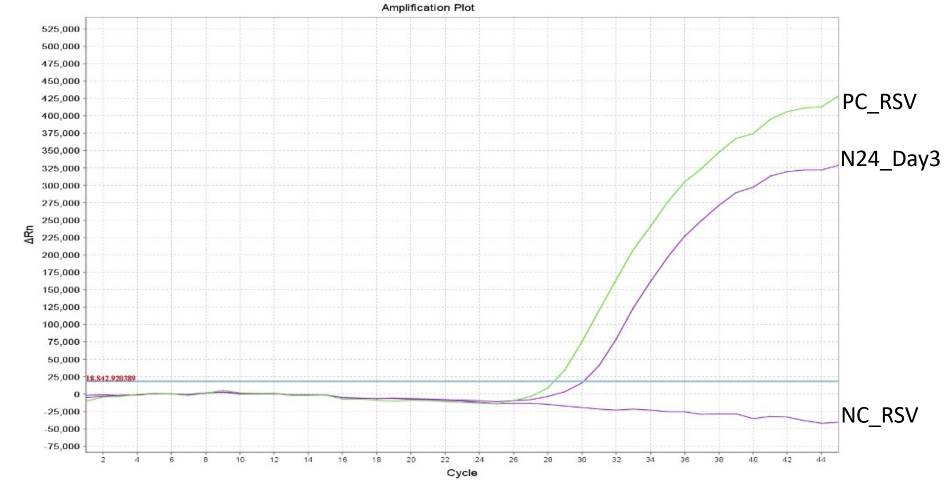 | 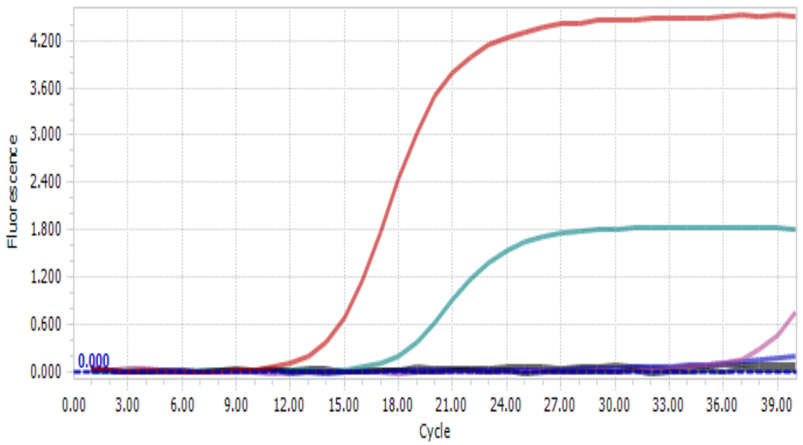 |

<table>
<tr><td rowspan="2">10</td><td>N27_D0</td><td>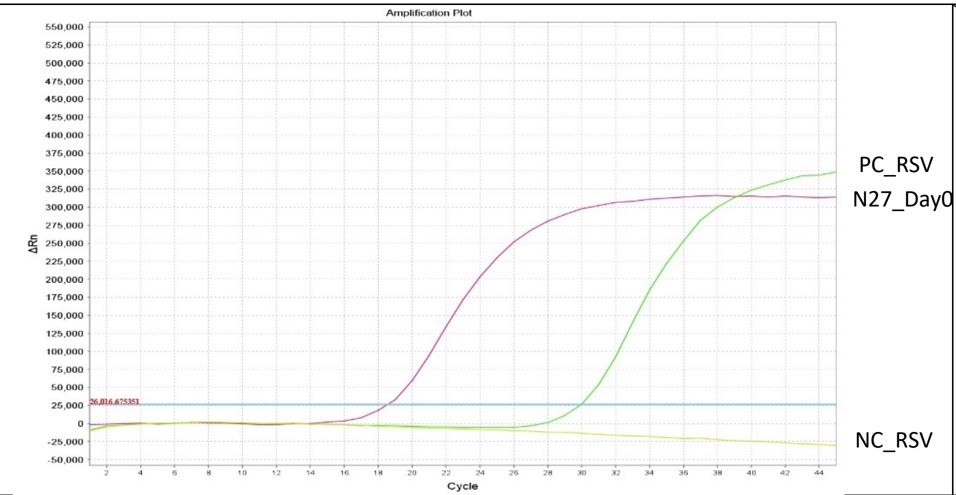
</td><td></td></tr>
<tr><td>N27_D3</td><td>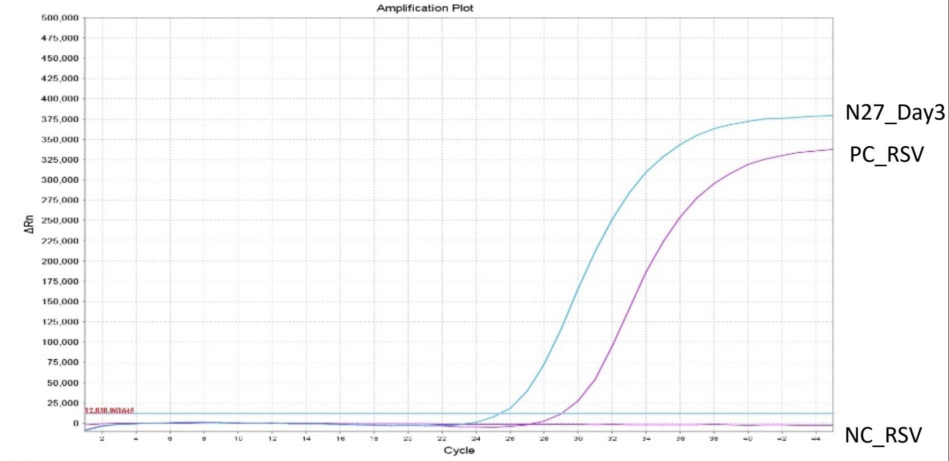
</td><td>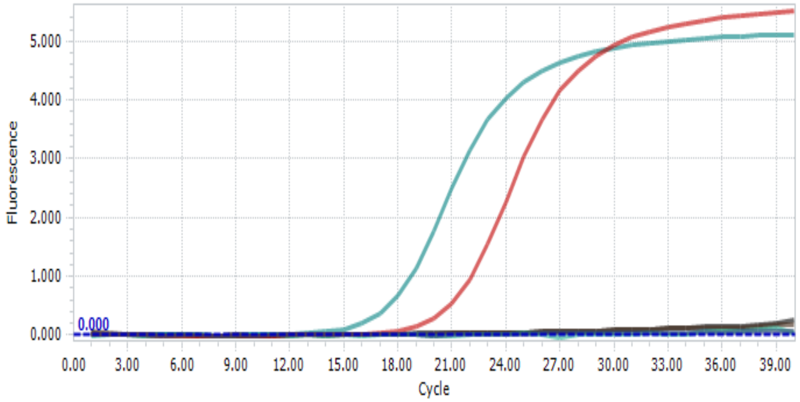
</td></tr>
</table>

<table>
<tr><td rowspan="2">11</td><td>N28 _D0</td><td>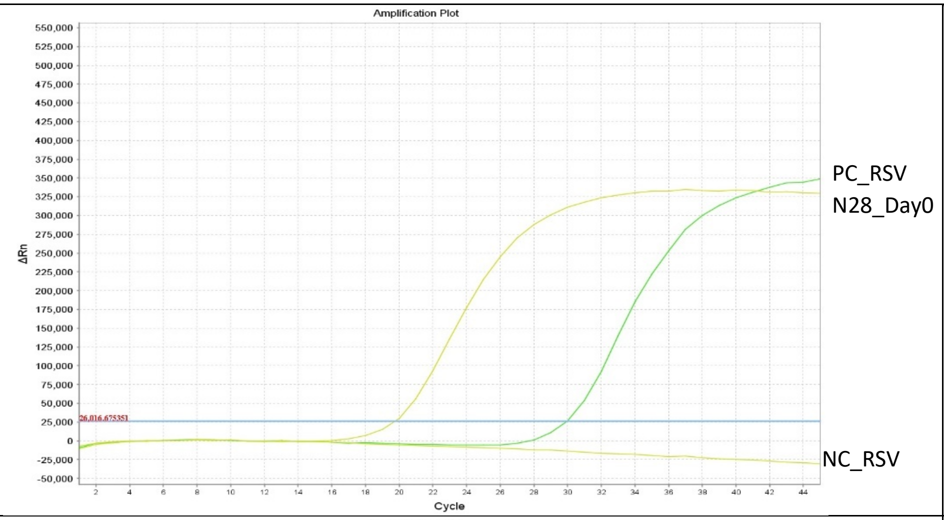
</td><td></td></tr>
<tr><td>N28_D3</td><td>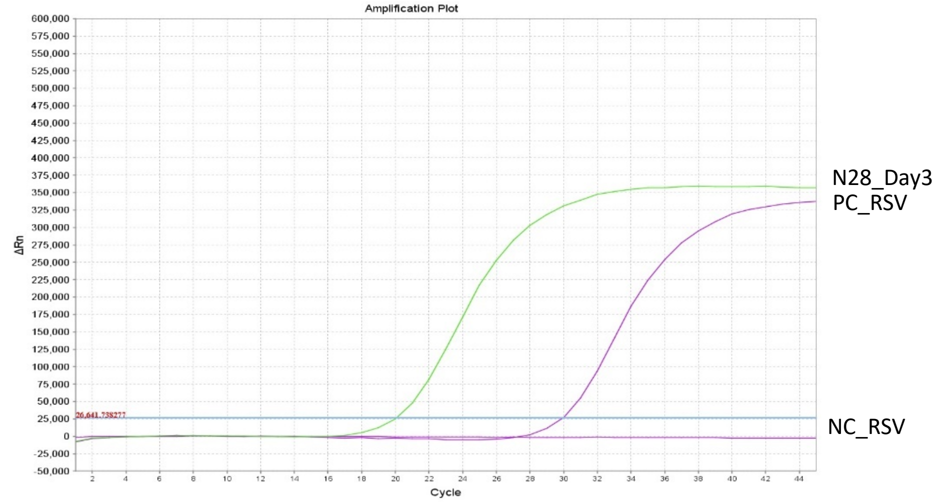
</td><td>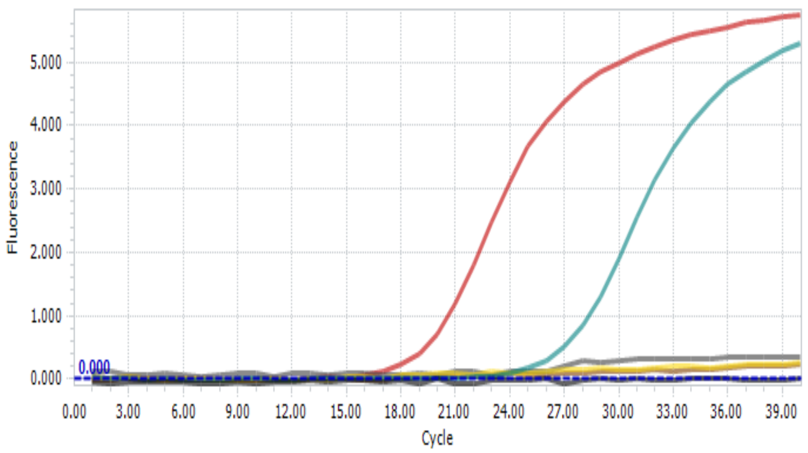
</td></tr>
</table>

<table>
<tr>
<td rowspan="2">12</td>
<td>N30_D0</td>
<td>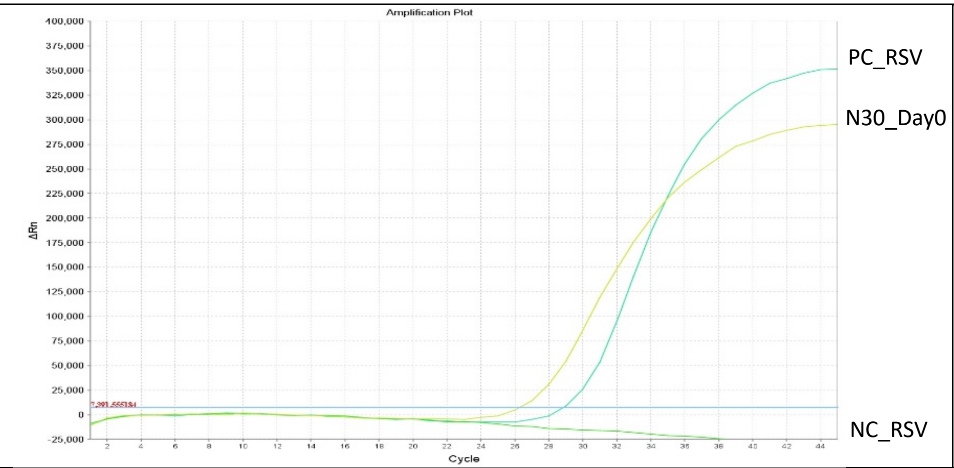
</td>
<td></td>
</tr>
<tr>
<td>N30_D3</td>
<td>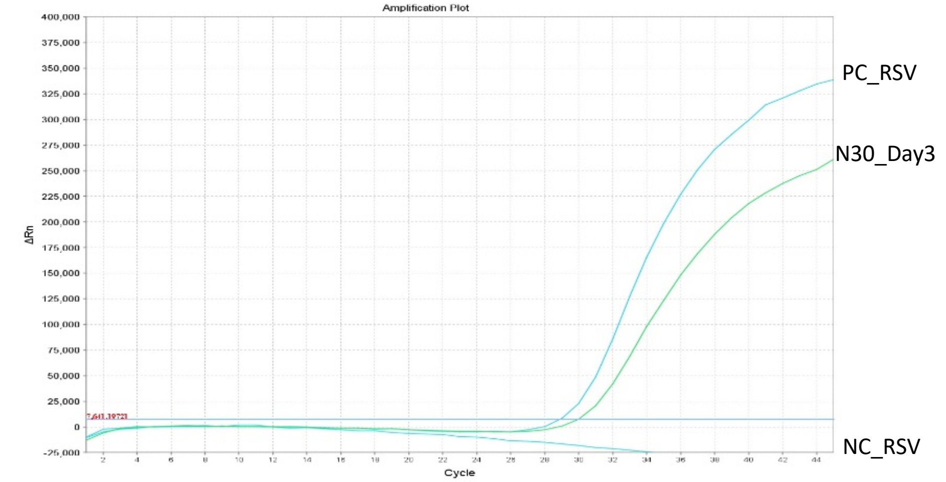
</td>
<td>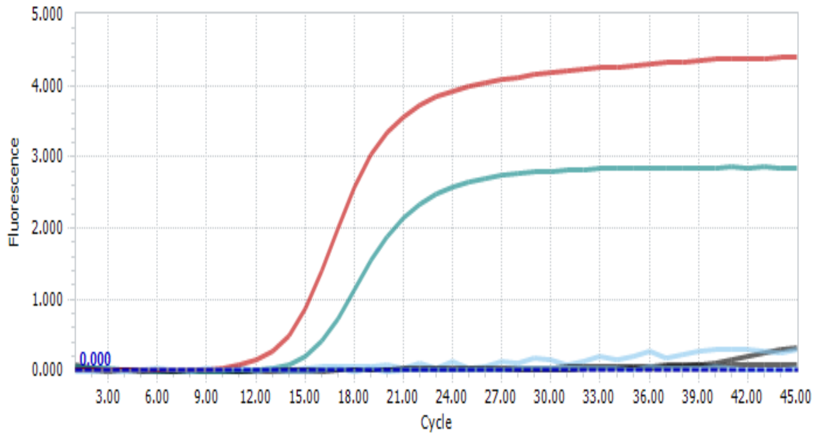
</td>
</tr>
</table>

| | | | |
|---|---|---|---|
| 13 | N35_D0 | 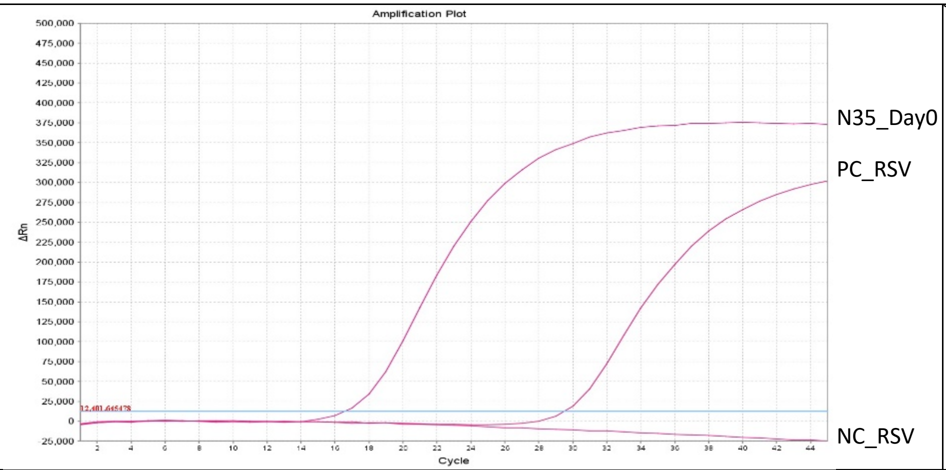 | |
| | N35_D3 | 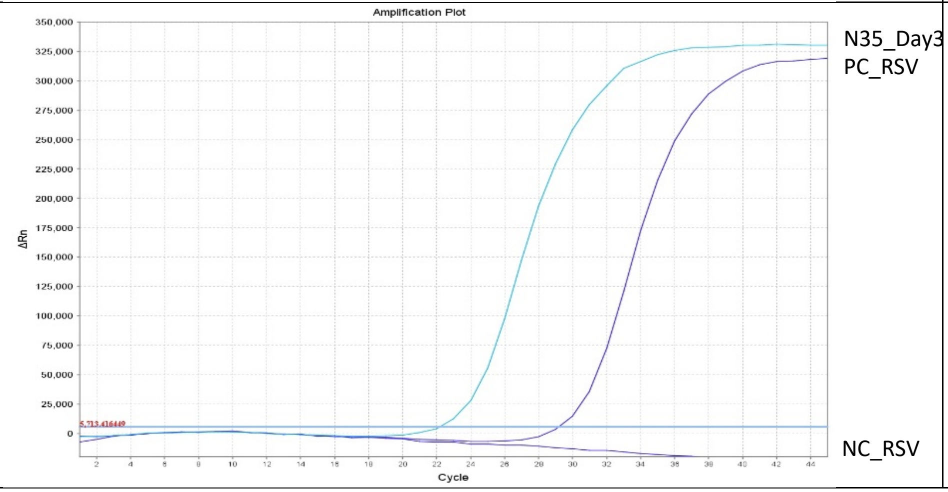 | 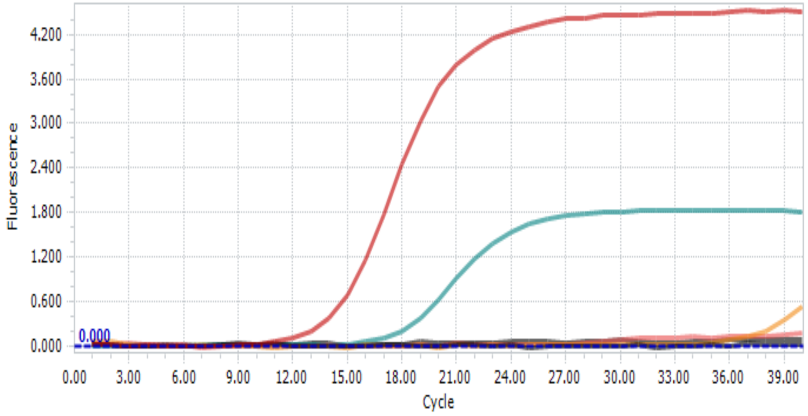 |

| | | | |
|---|---|---|---|
| 14 | N36 _D0 | 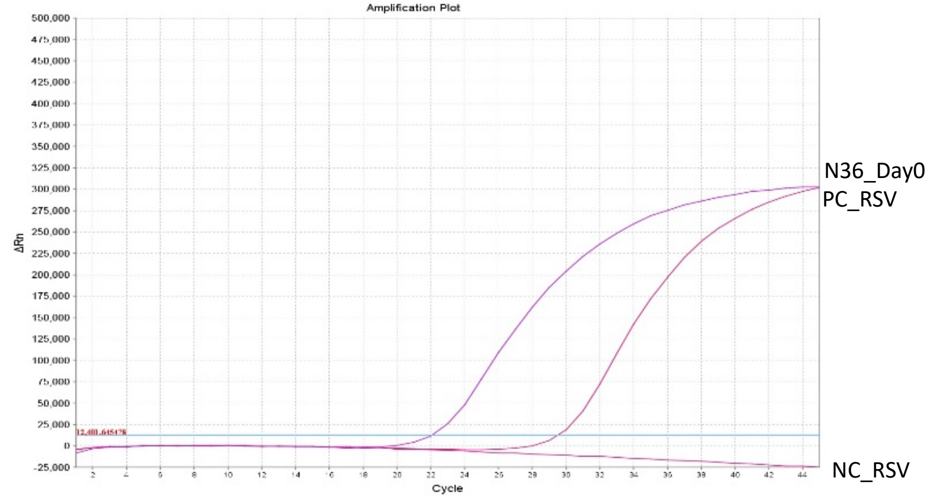 | |
| | N36_D3 | 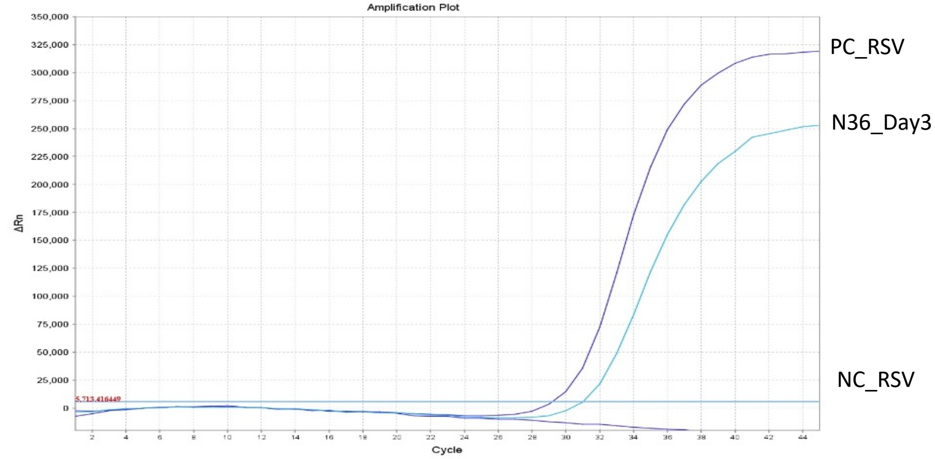 | 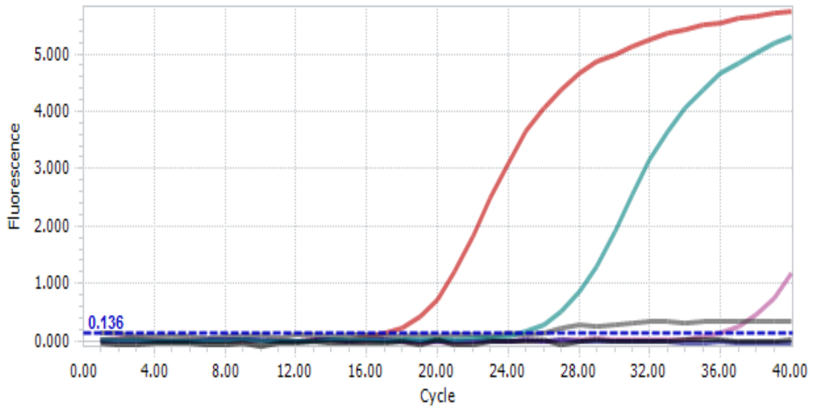 |

<table>
<tr><td rowspan="2">15</td><td>N42 _D0</td><td>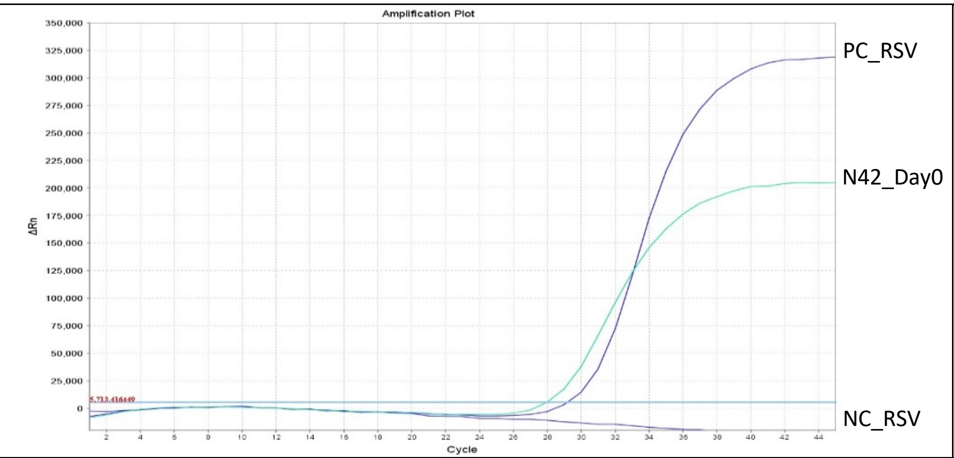
</td><td></td></tr>
<tr><td>N42_D3</td><td>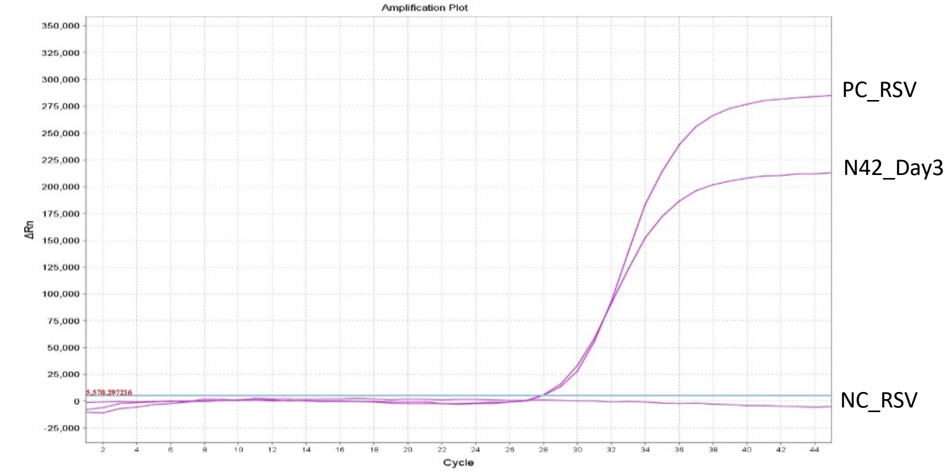
</td><td>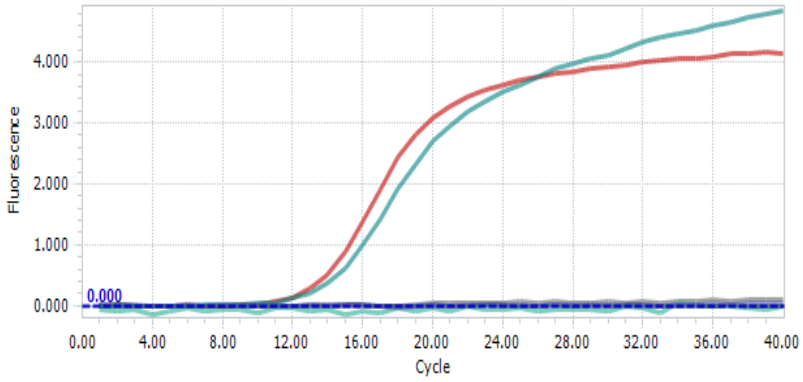
</td></tr>
</table>

<table>
<tr><td rowspan="2">16</td><td>N44 _D0</td><td>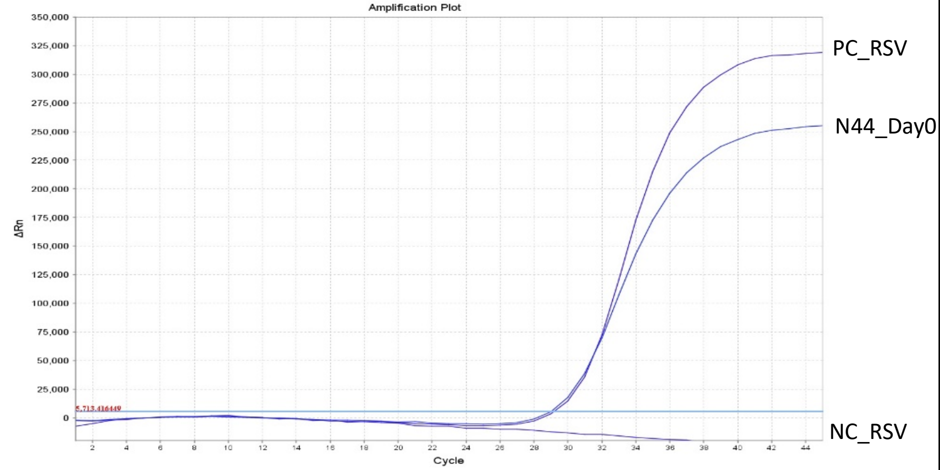</td><td></td></tr>
<tr><td>N44_D3</td><td>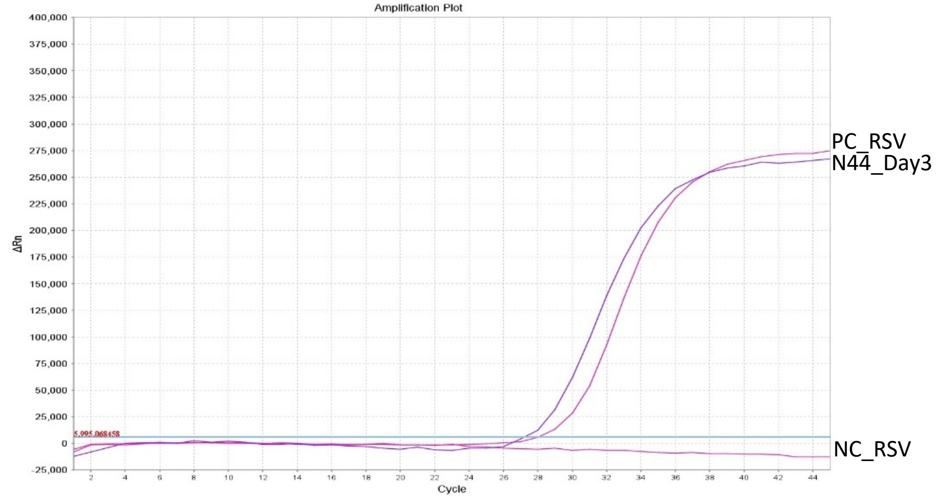</td><td>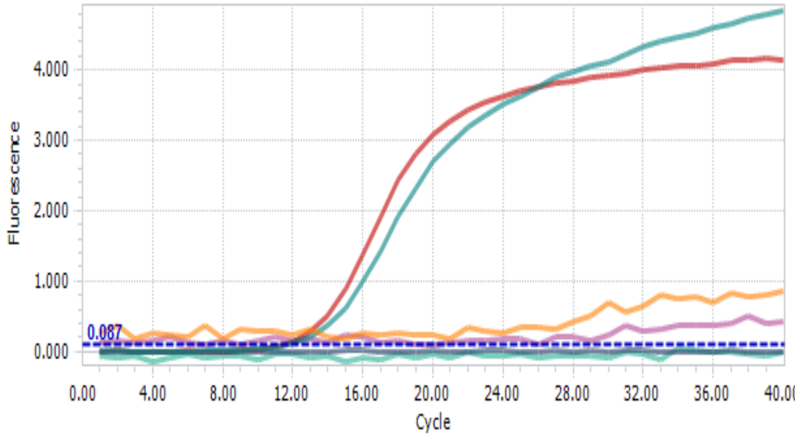</td></tr>
</table>

| | | | |
|---|---|---|---|
| 17 | N46 _D0 | 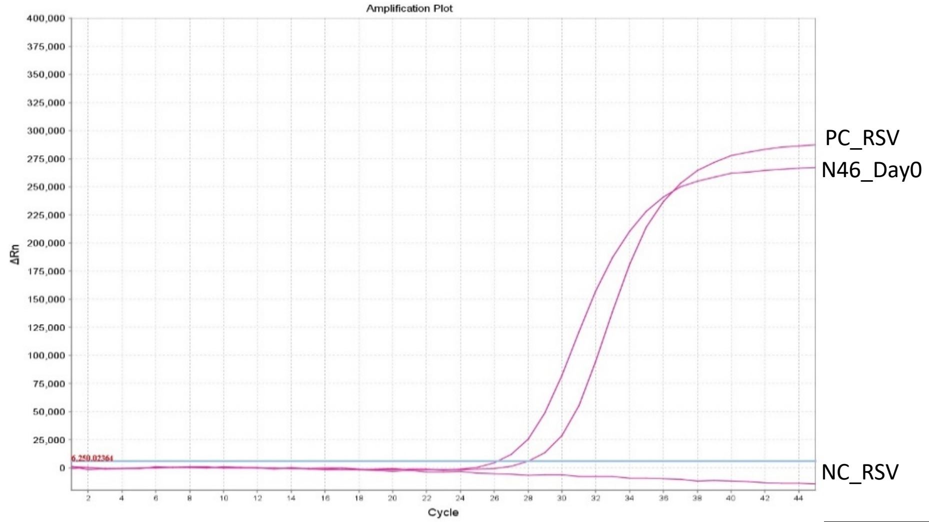 | |
| | N46_D3 | 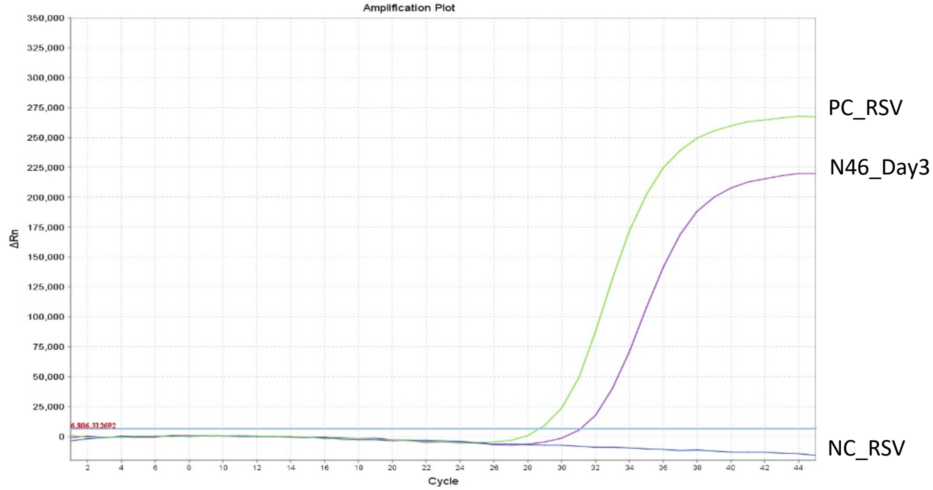 | 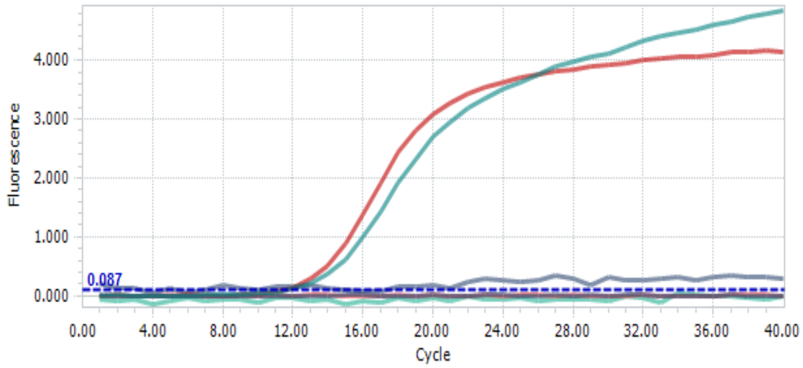 |

| | | | |
|---|---|---|---|
| 18 | N49 _D0 | 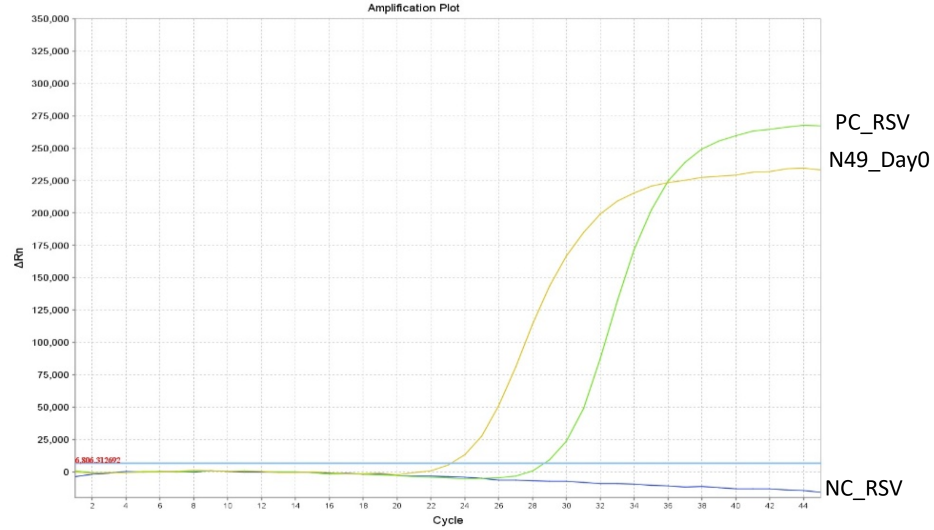 | |
| | N49_D3 | 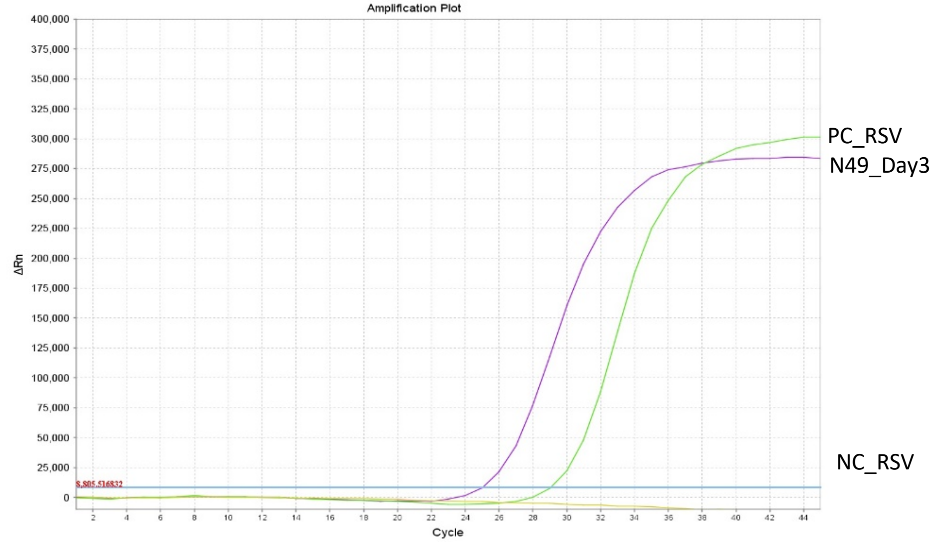 | 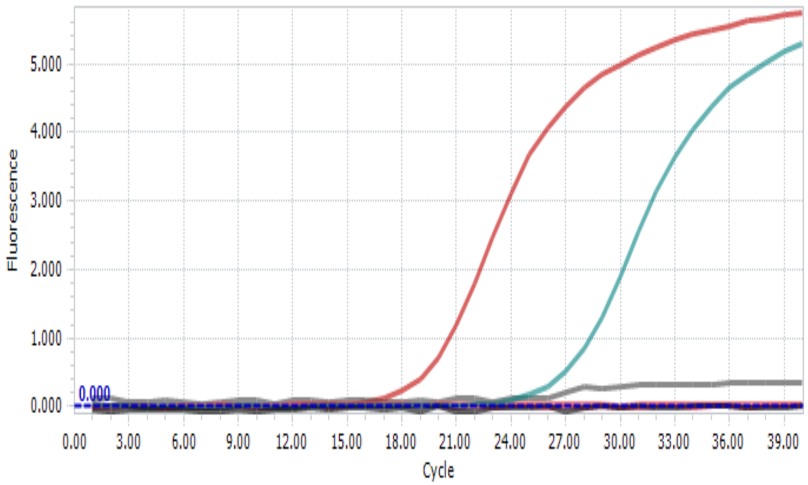 |

<table>
<tr><td rowspan="2">19</td><td>N50_D0</td><td>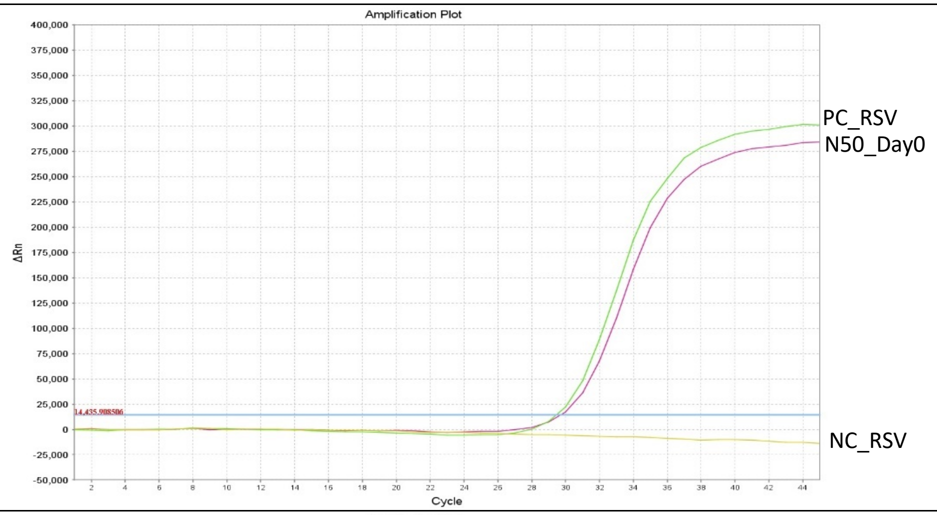
</td><td></td></tr>
<tr><td>N50_D3</td><td>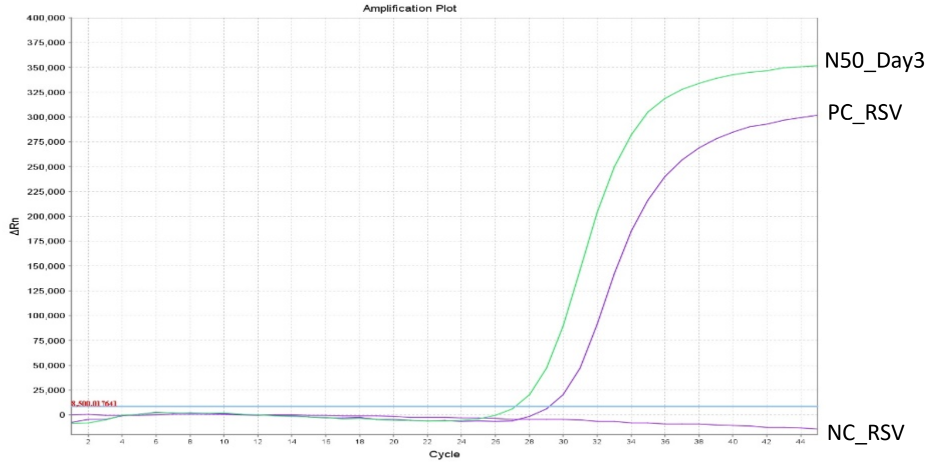
</td><td>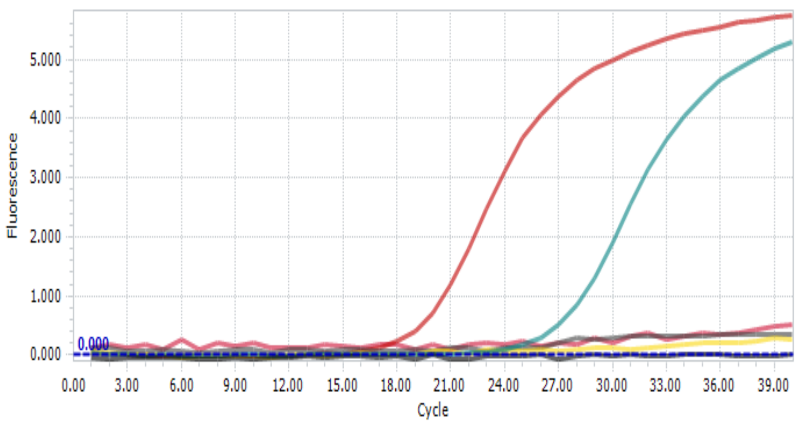
</td></tr>
</table>

<table>
<tr><td rowspan="2">20</td><td>N53 _D0</td><td>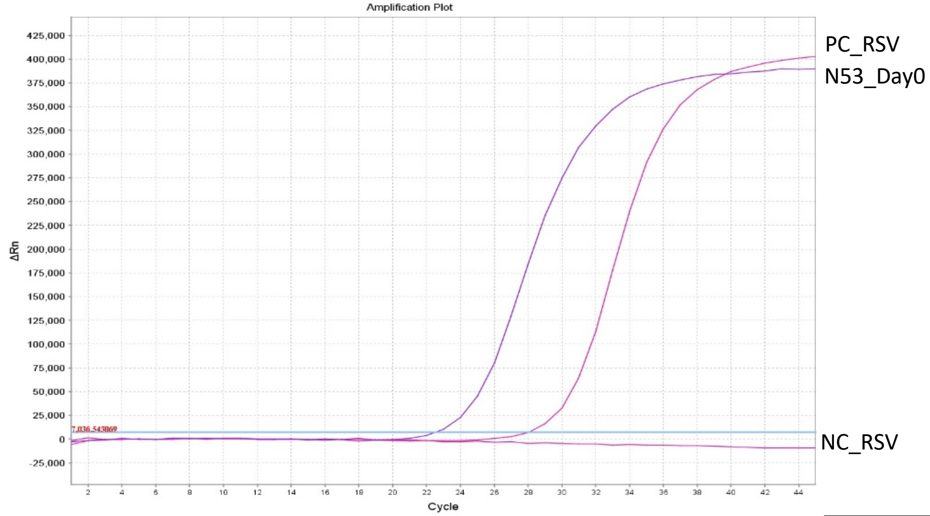</td><td></td></tr>
<tr><td>N53_D3</td><td>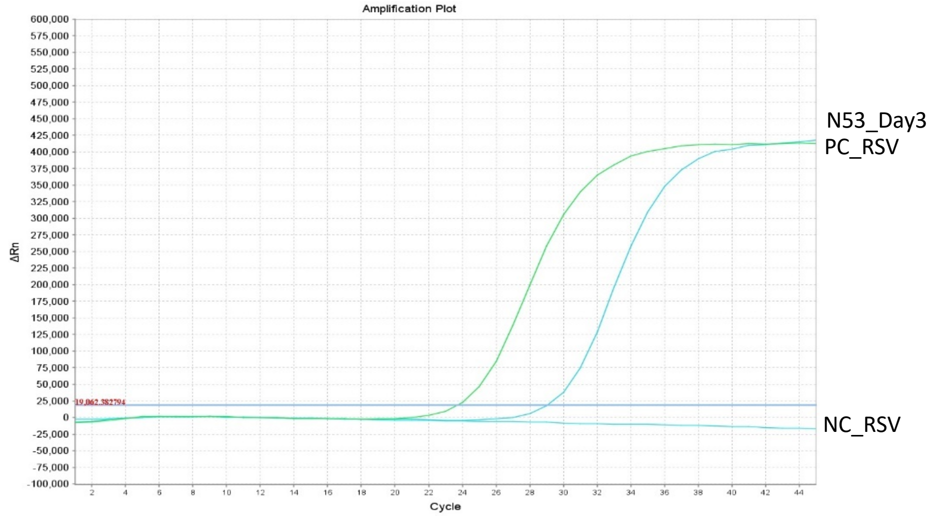</td><td>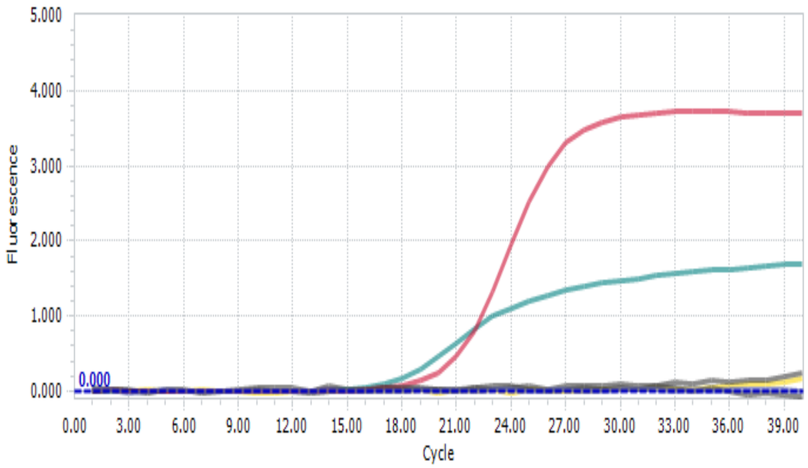</td></tr>
</table>

<table>
<tr>
<td rowspan="2">21</td>
<td>N55 _D0</td>
<td>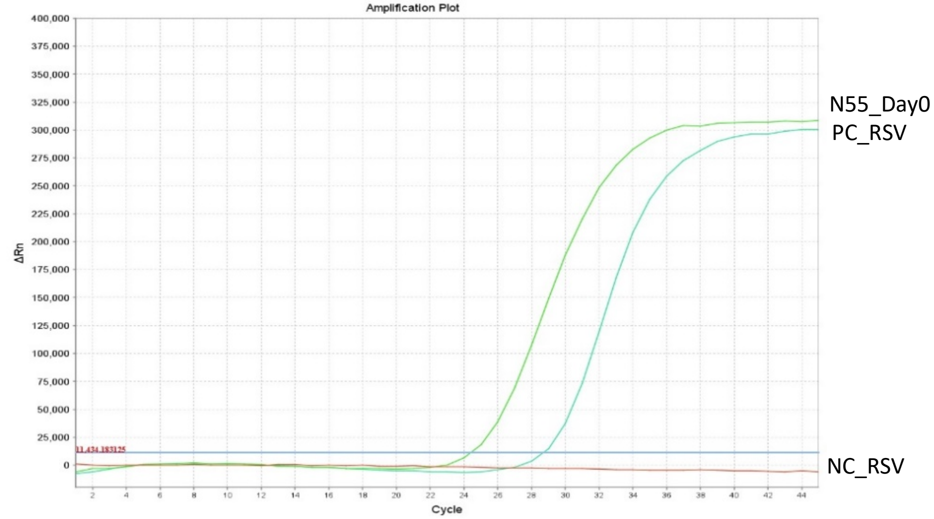
</td>
<td></td>
</tr>
<tr>
<td>N55_D3</td>
<td>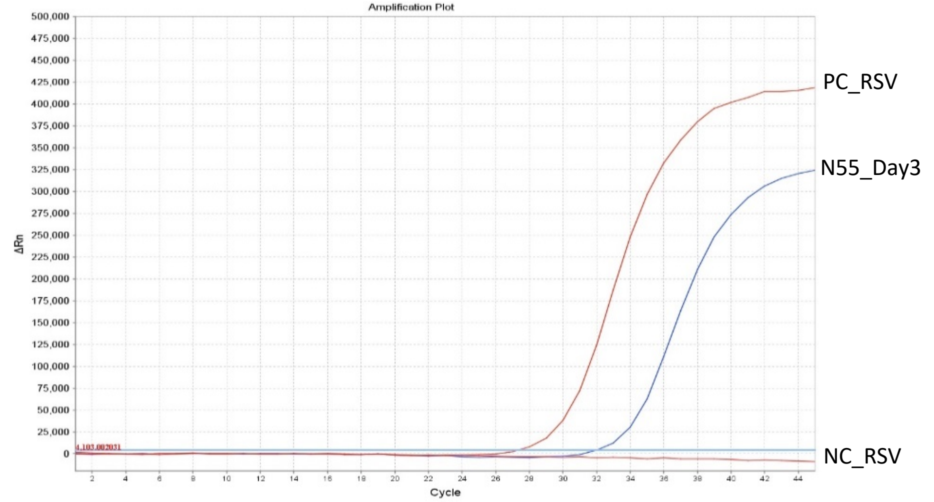
</td>
<td>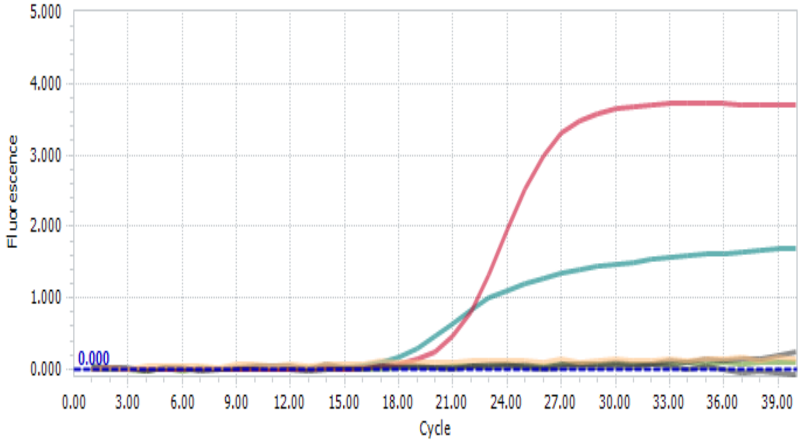
</td>
</tr>
</table>

<table>
<tr><td rowspan="2">22</td><td>N59_D0</td><td>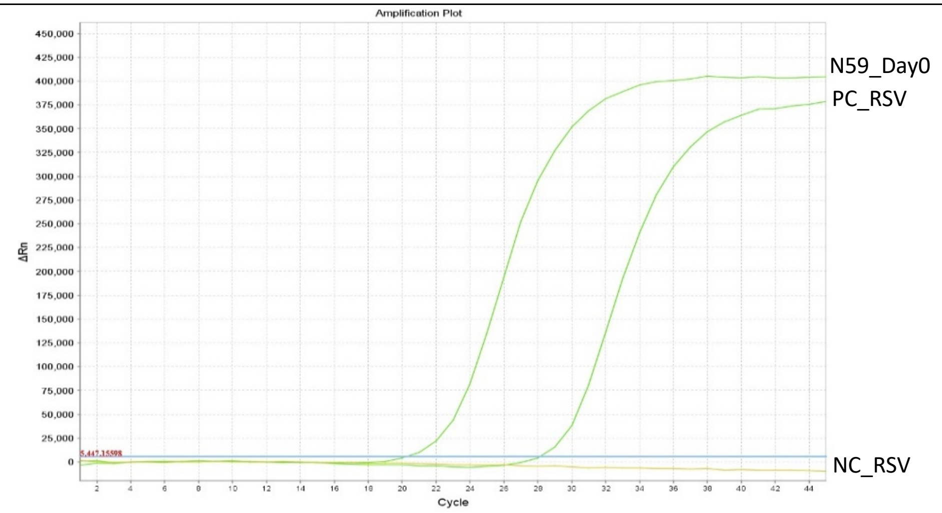
</td><td></td></tr>
<tr><td>N59_D3</td><td>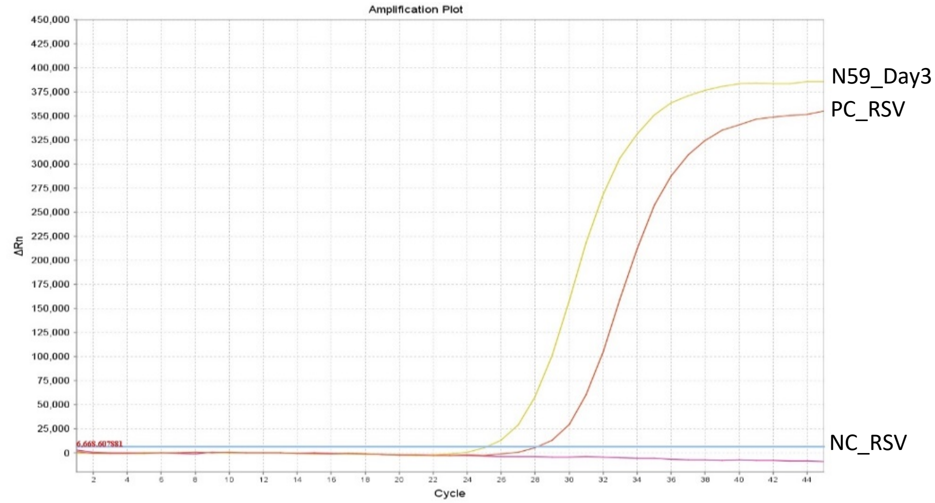
</td><td>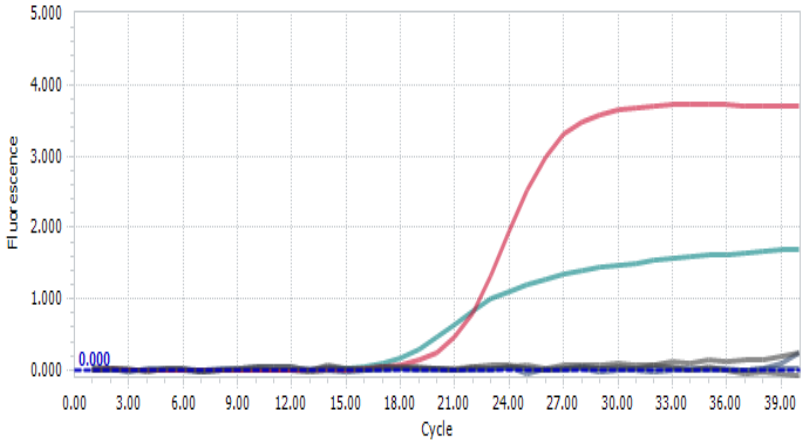
</td></tr>
</table>

| | | | |
|---|---|---|---|
| 23 | N60_D0 | 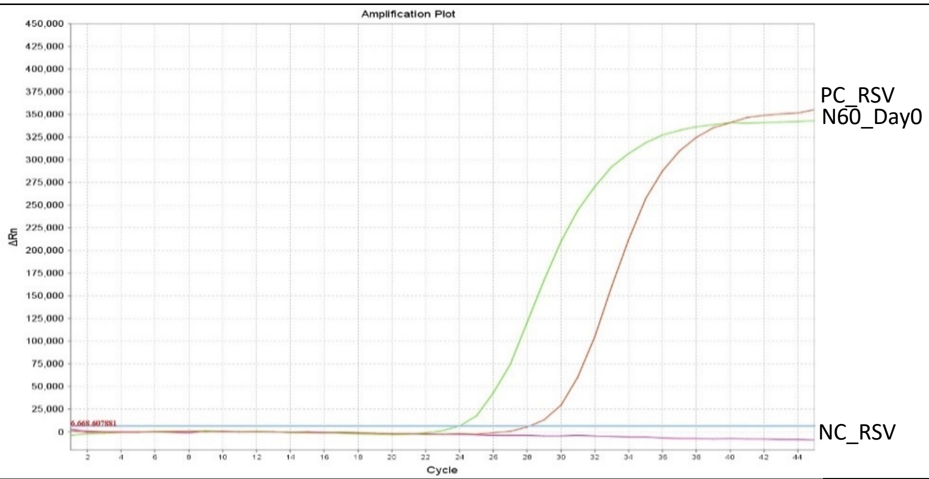 | |
| | N60_D3 | 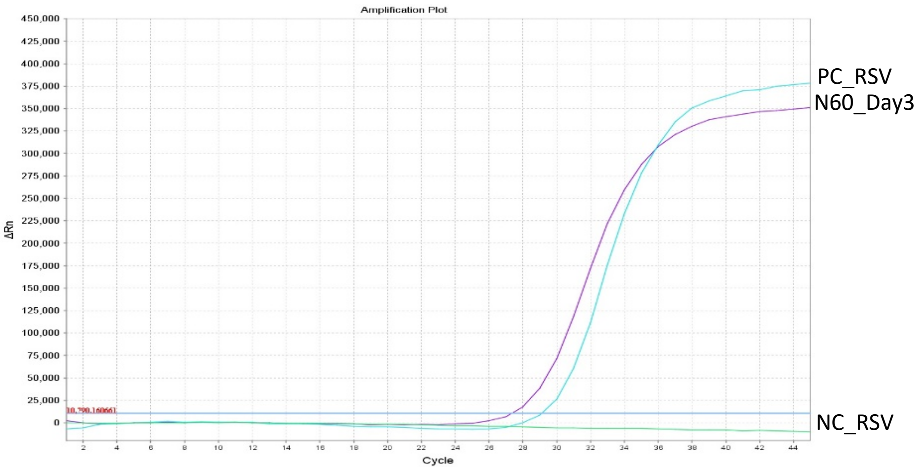 | 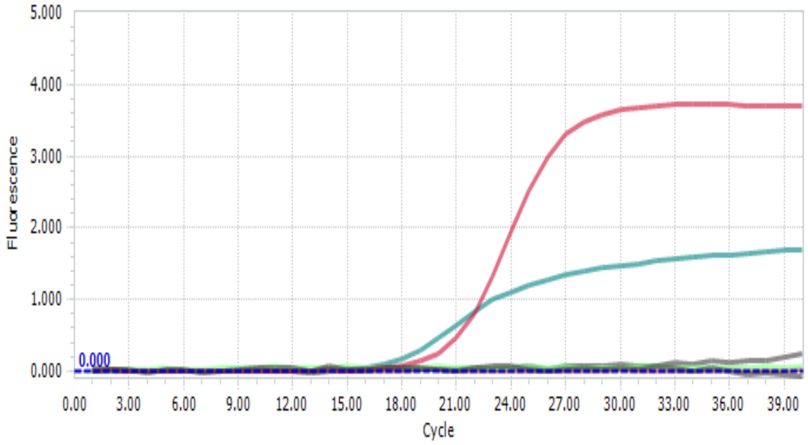 |

<table>
<tr><td>24</td><td>N65_D0</td><td>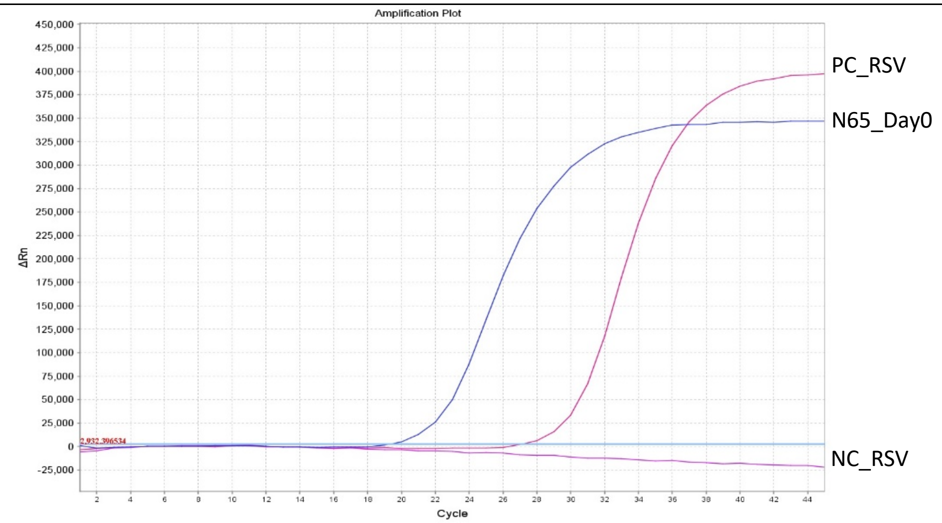</td><td></td></tr>
<tr><td></td><td>N65_D3</td><td>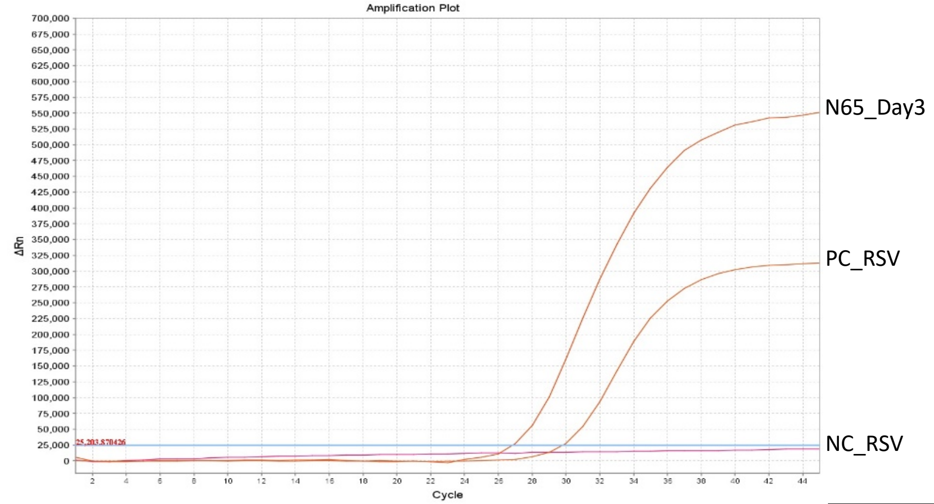</td><td>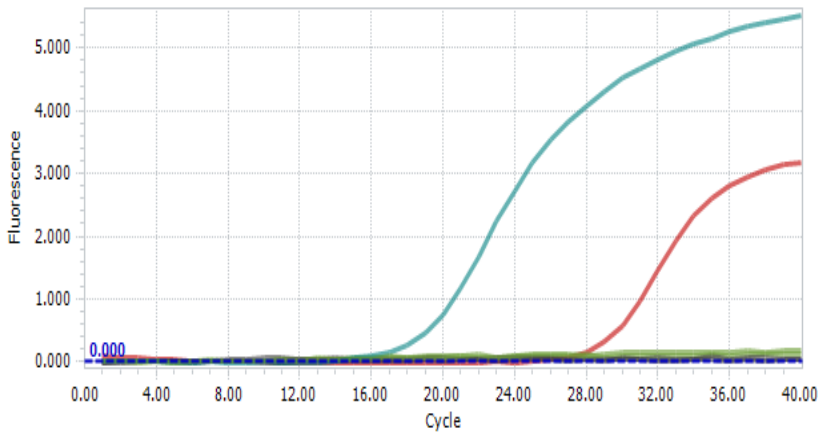</td></tr>
</table>

<table>
<tr><td rowspan="2">25</td><td>N66_D0</td><td>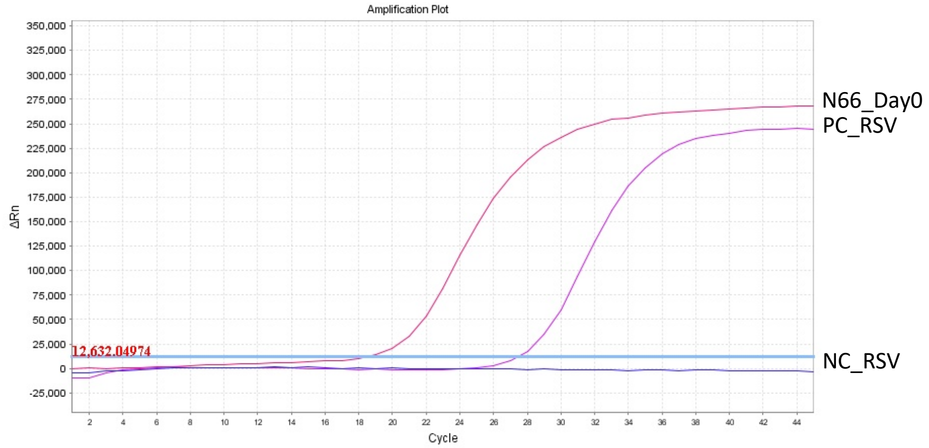
</td><td></td></tr>
<tr><td>N66_D3</td><td>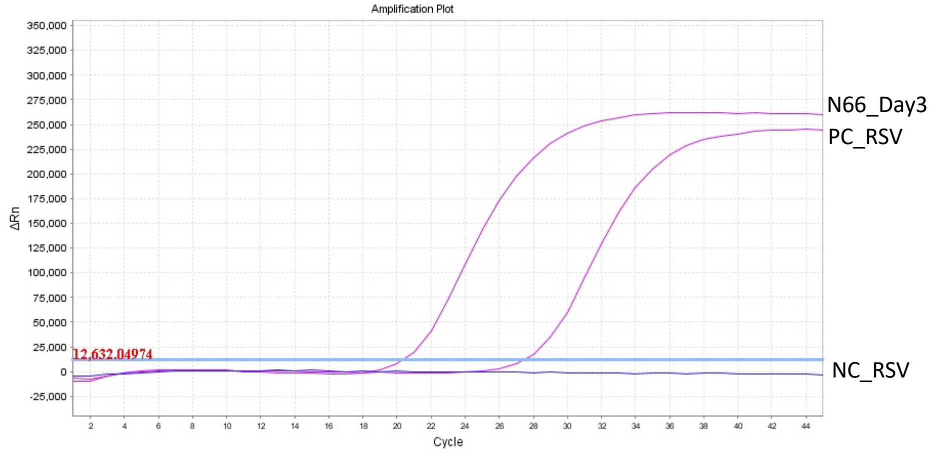
</td><td>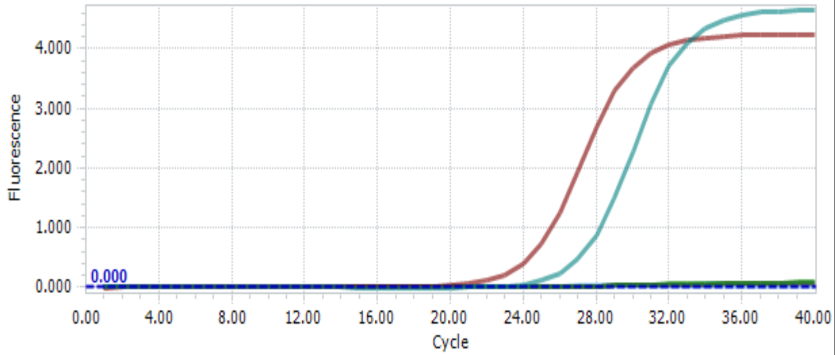
</td></tr>
</table>

| | | | |
|---|---|---|---|
| 26 | N70_D0 | 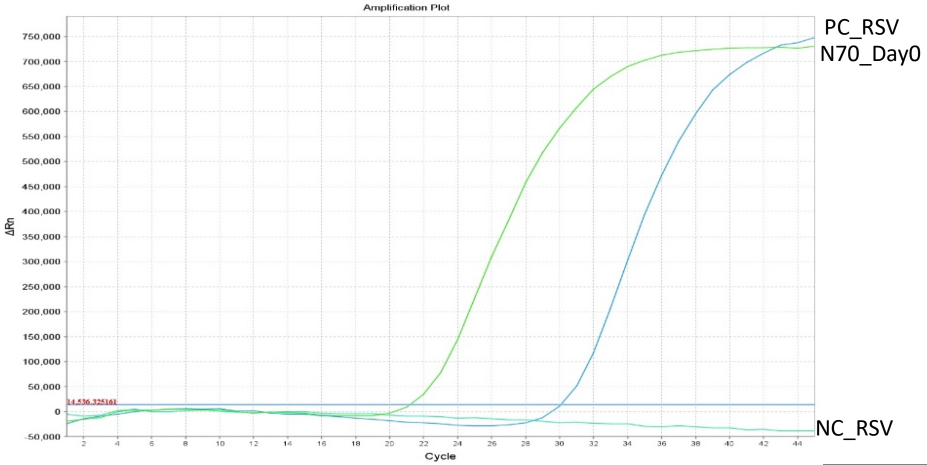 | |
| | N70_D3 | 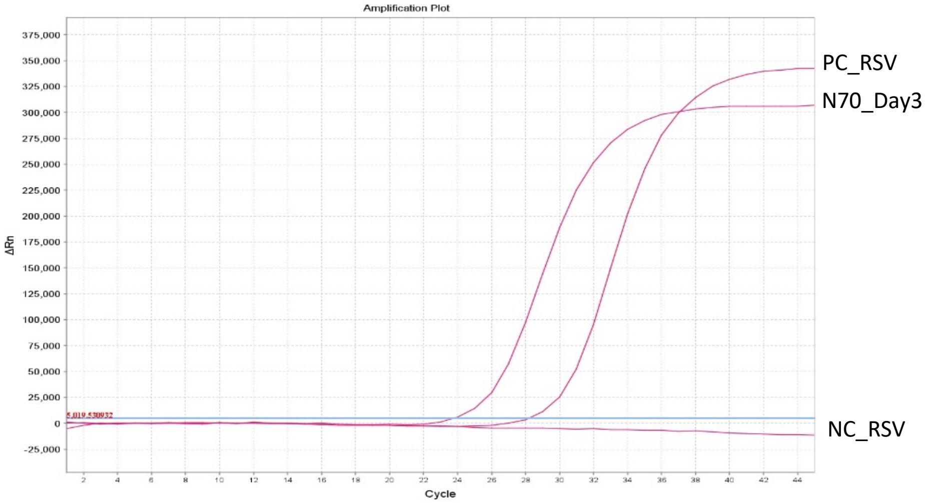 | 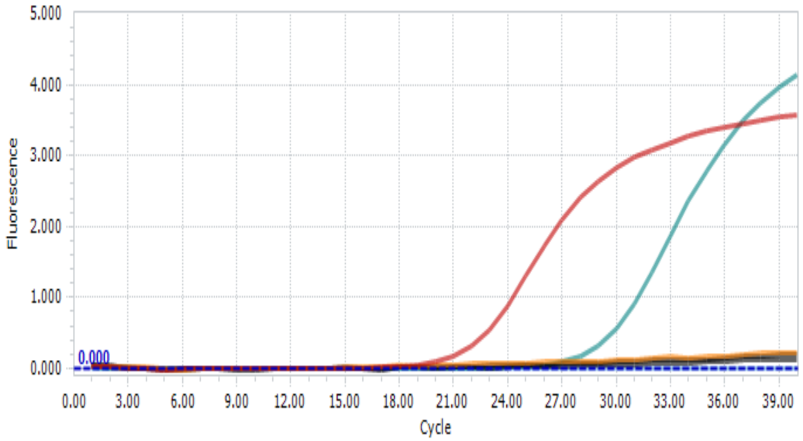 |

| | | | |
|---|---|---|---|
| 27 | N71_D0 | 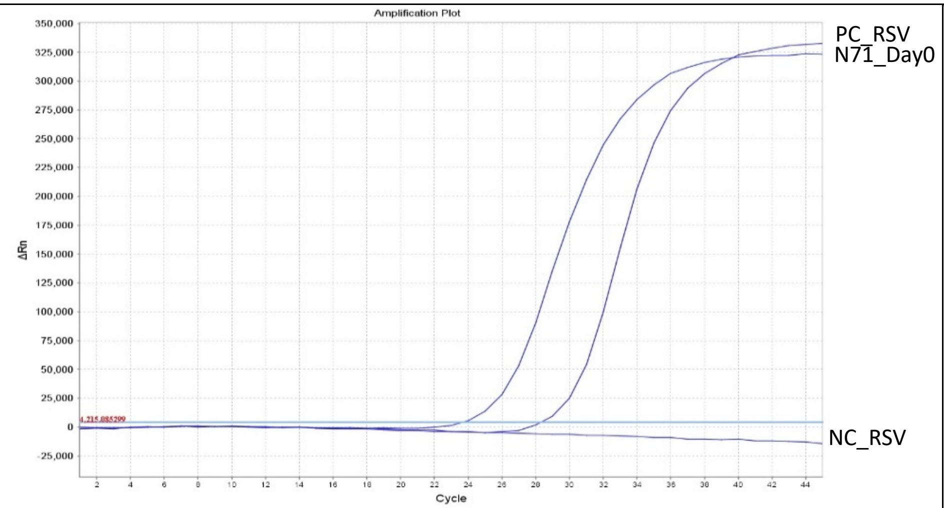  | |
| | N71_D3 | 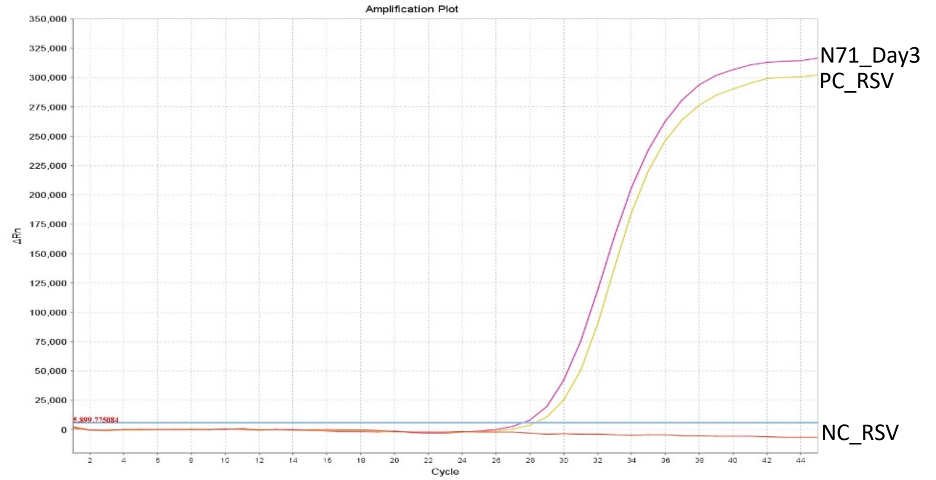  | 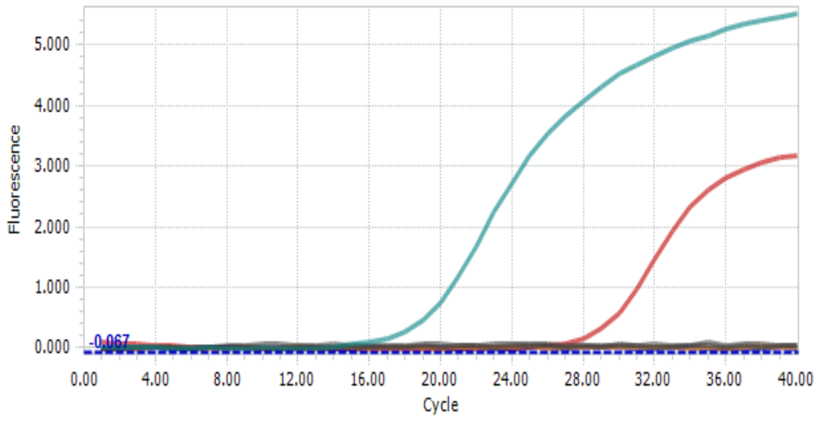  |

| | | | |
|---|---|---|---|
| 28 | N74_D0 | 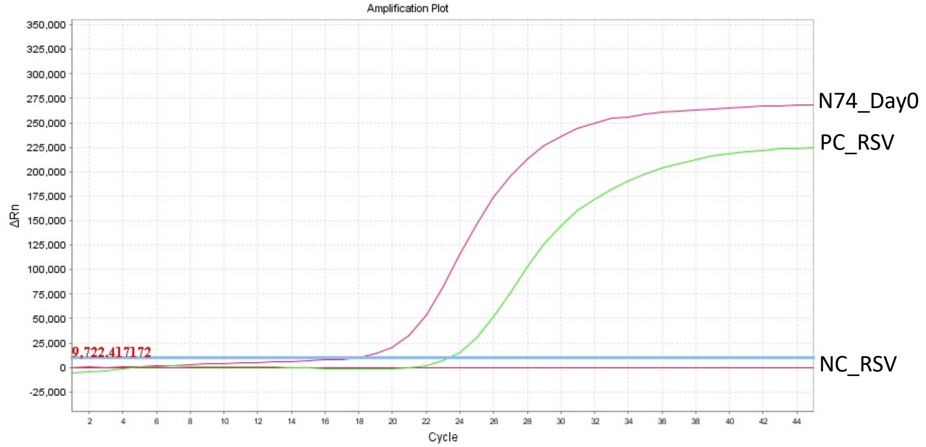 | |
| | N74_D3 | 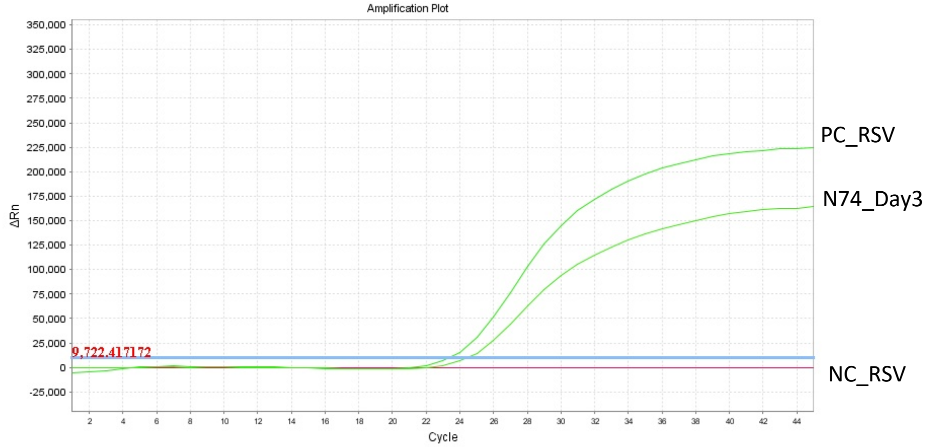 | 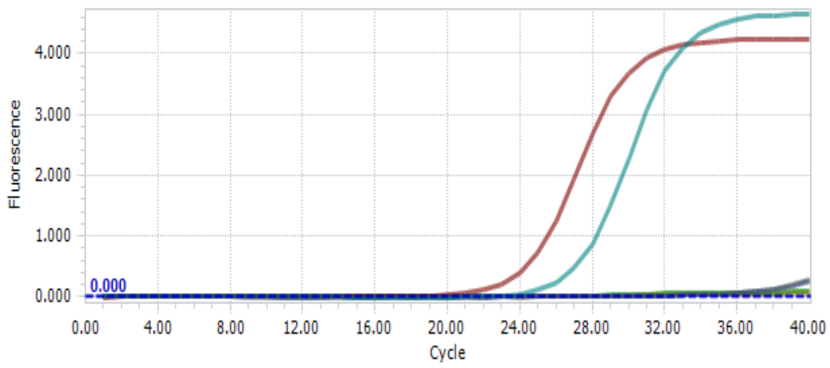 |

<table>
<tr><td>29</td><td>N75_D0</td><td>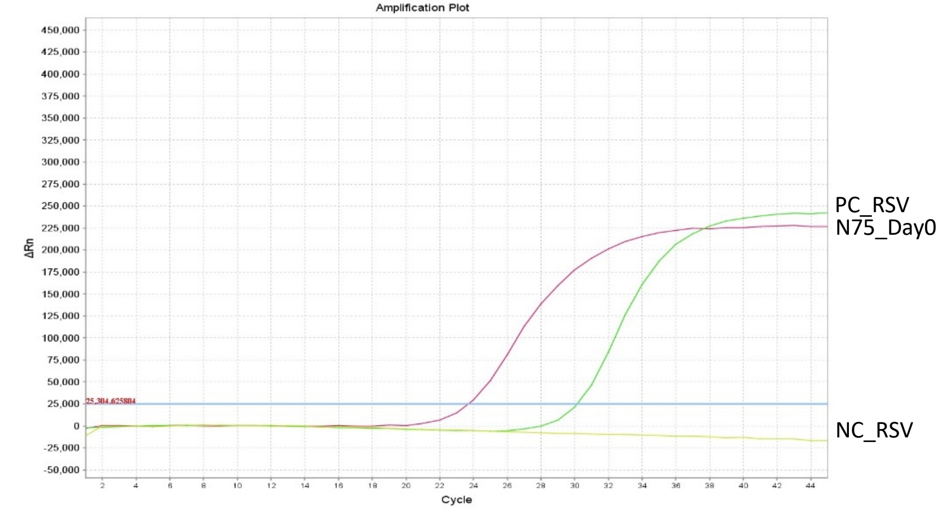
</td><td></td></tr>
<tr><td></td><td>N75_D3</td><td>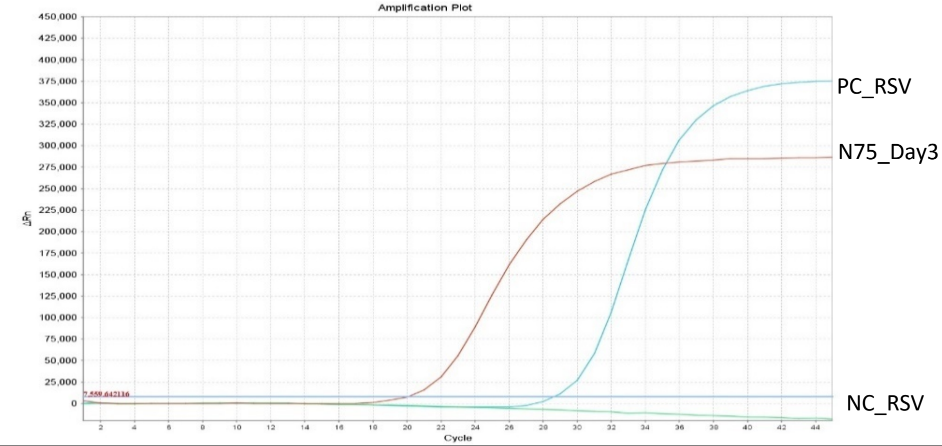
</td><td>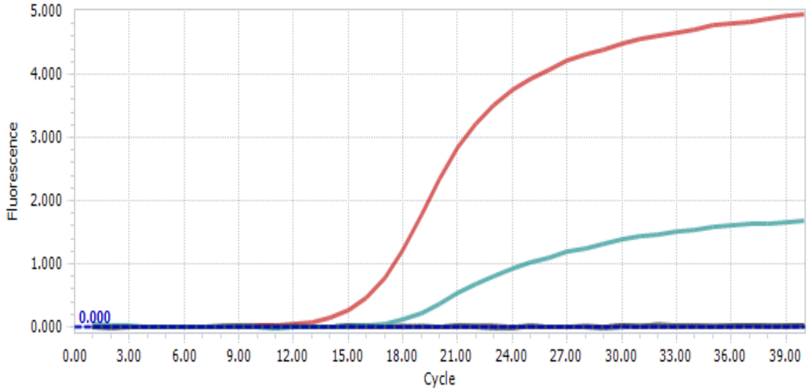
</td></tr>
</table>

| | | | |
|---|---|---|---|
| 30 | N76_D0 | 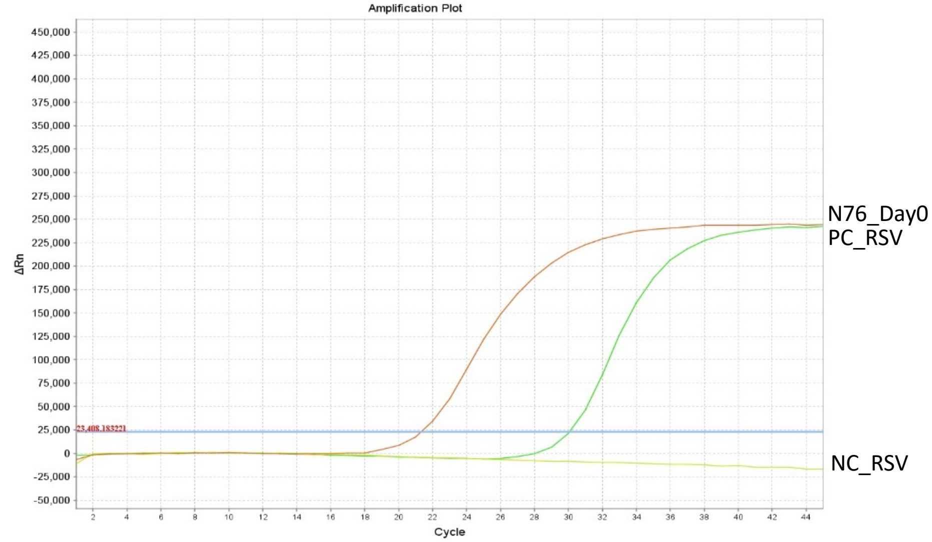 | |
| | N76_D3 | 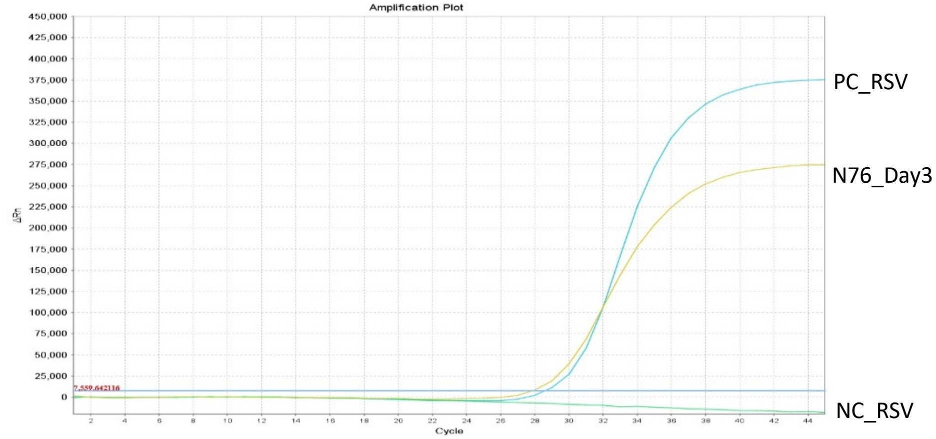 | 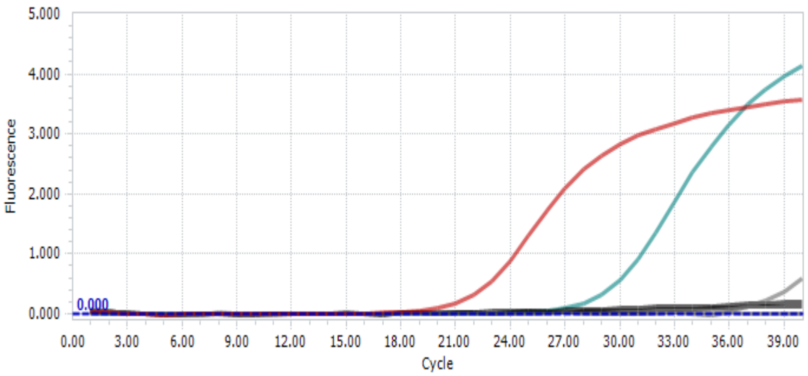 |

| | | | |
|---|---|---|---|
| 31 | N81_D0 | 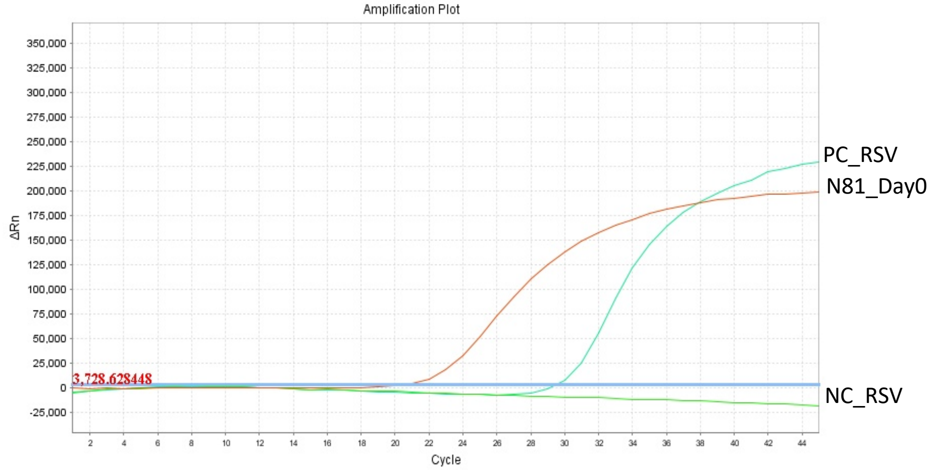 | |
| | N81 _D3 | 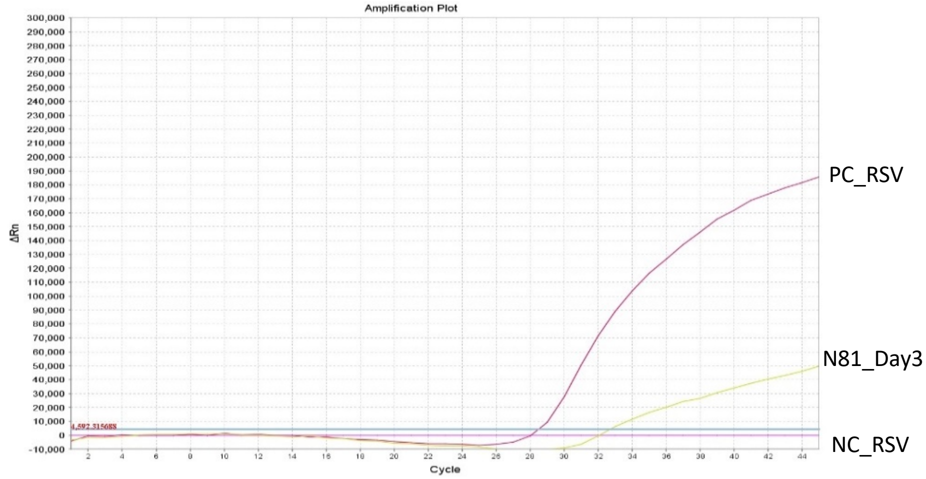 | 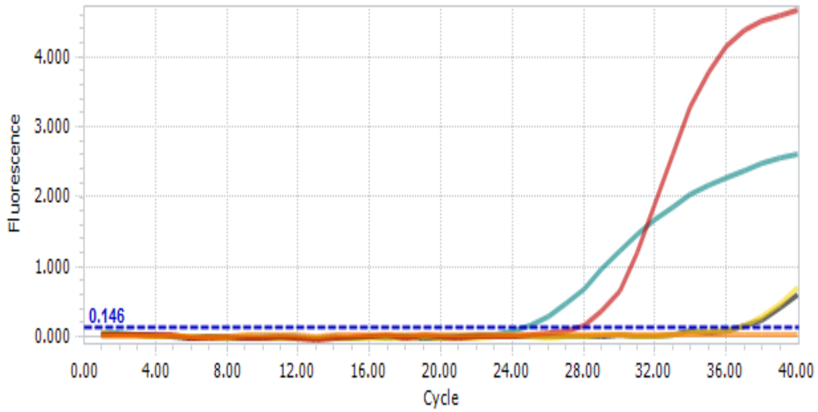 |

<table>
<tr>
<td rowspan="2">32</td>
<td>N82_D0</td>
<td>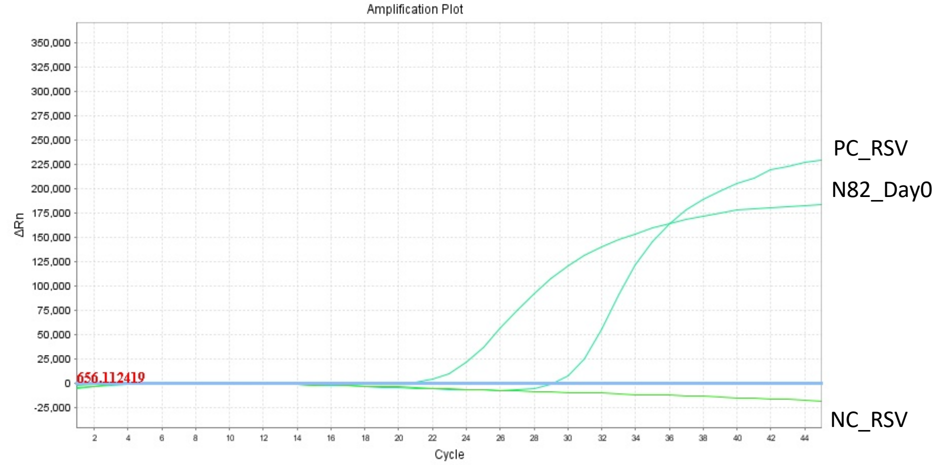
</td>
<td></td>
</tr>
<tr>
<td>N82_D3</td>
<td>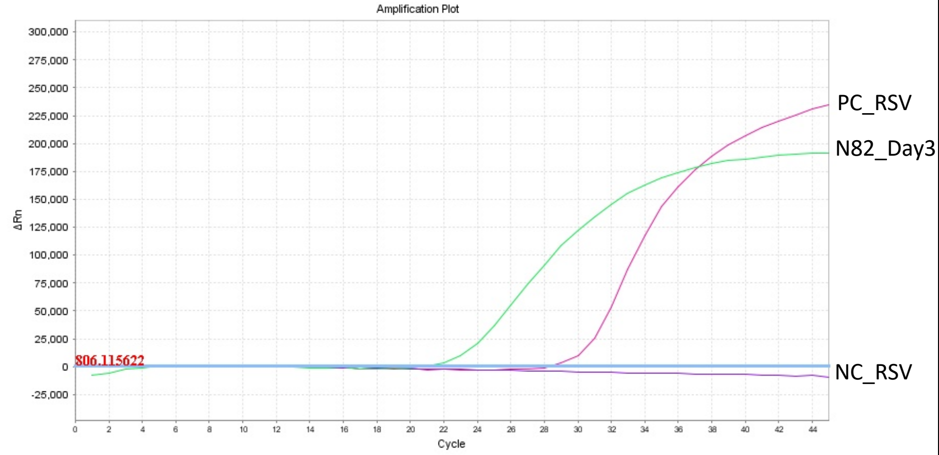
</td>
<td>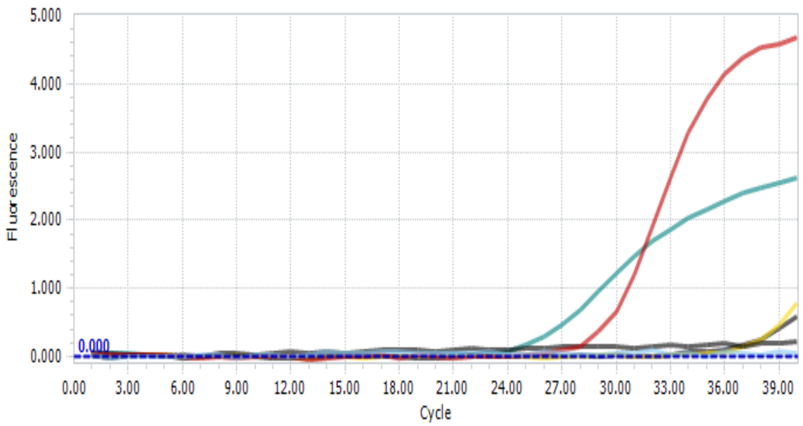
</td>
</tr>
</table>

| | | | |
|---|---|---|---|
| 33 | N87_D0 | 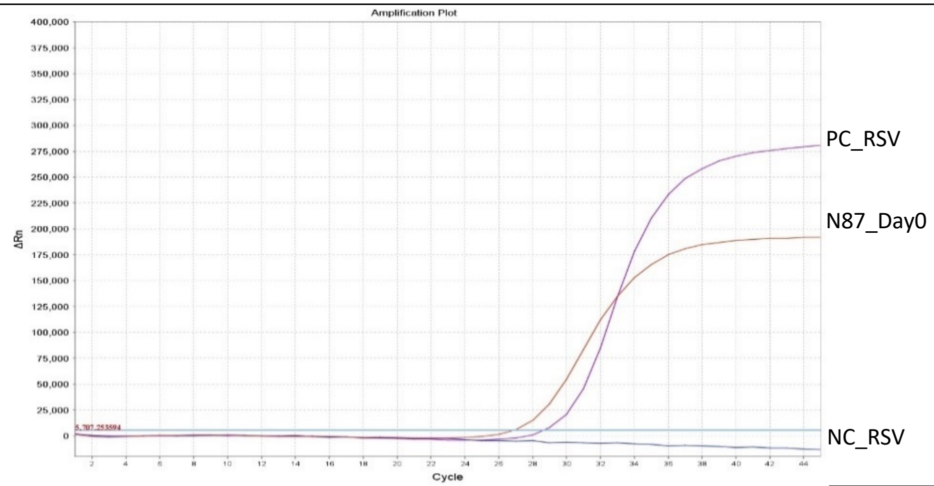  | |
| | N87_D3 | 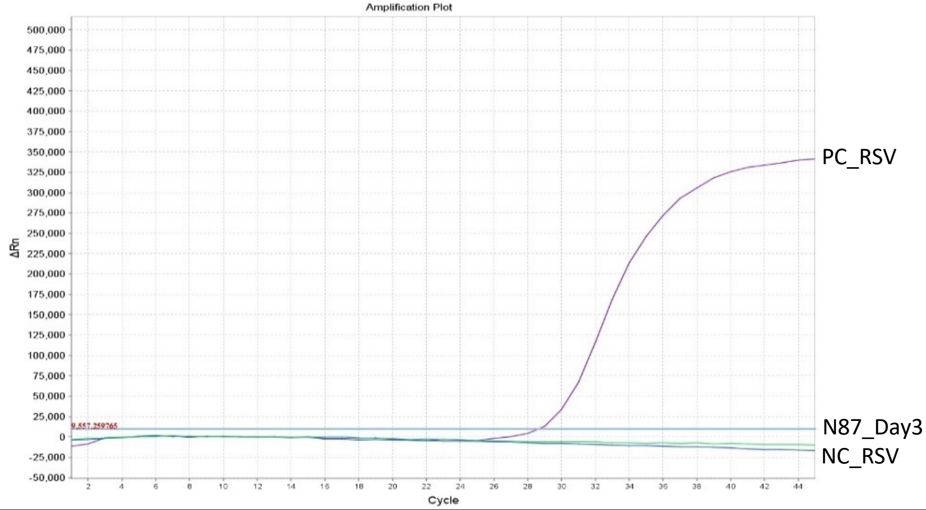  | 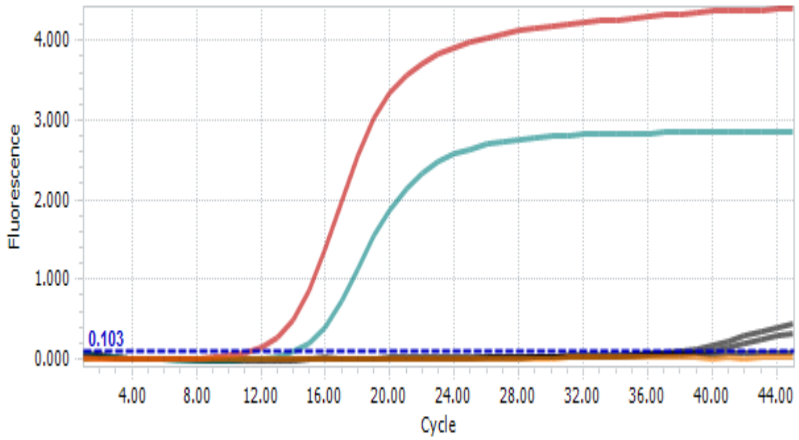  |

| | | | |
|---|---|---|---|
| 34 | N89_D0 | 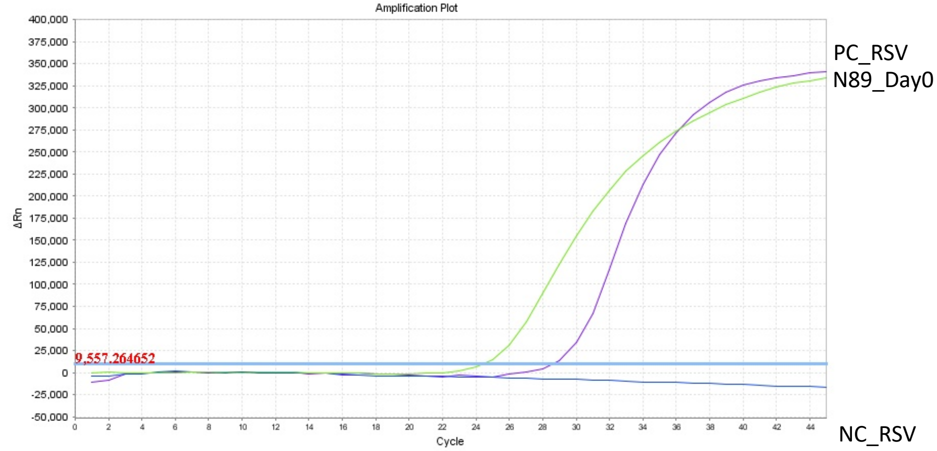 | |
| | N89_D3 | 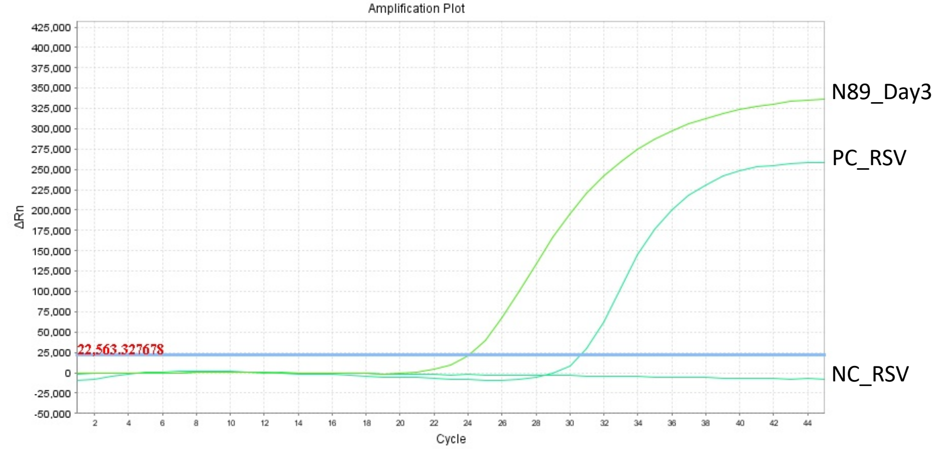 | 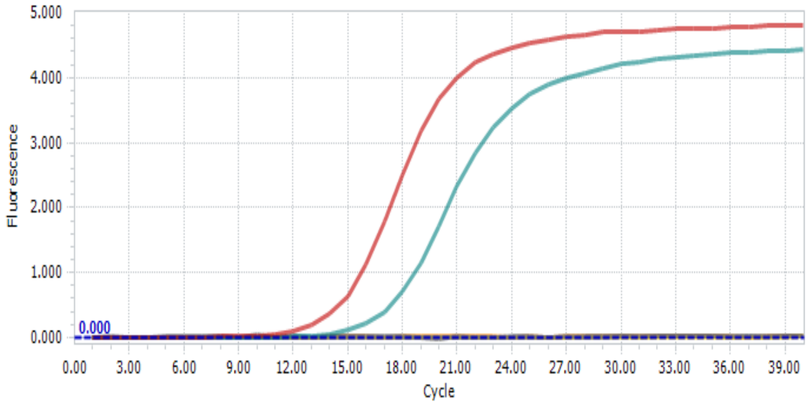 |

<table>
<tr><td>35</td><td>N90_D0</td><td>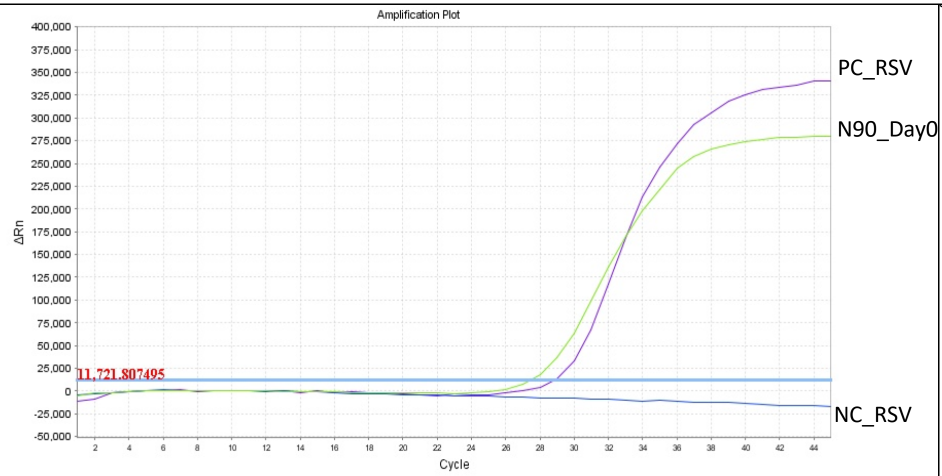
</td><td></td></tr>
<tr><td></td><td>N90 _D3</td><td>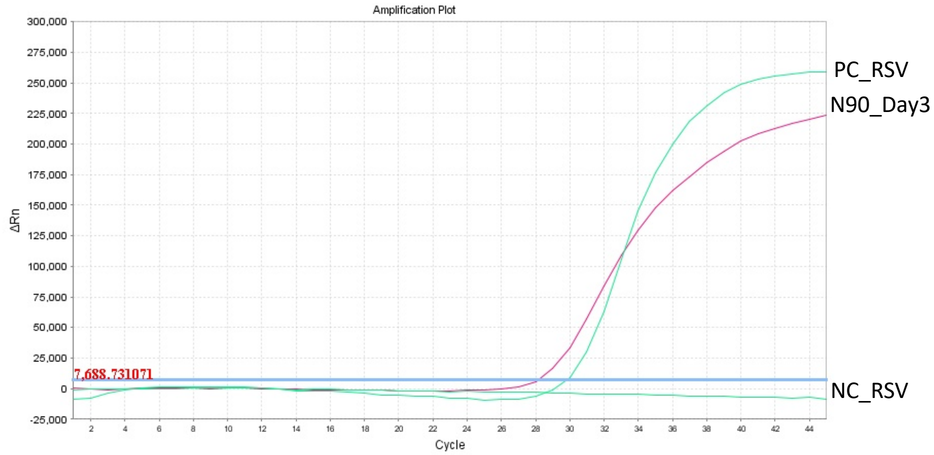
</td><td>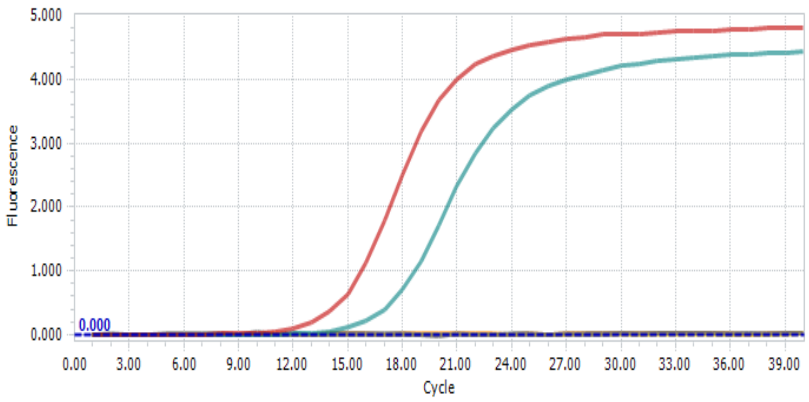
</td></tr>
</table>

<table>
<tr><td>36</td><td>N94_D0</td><td>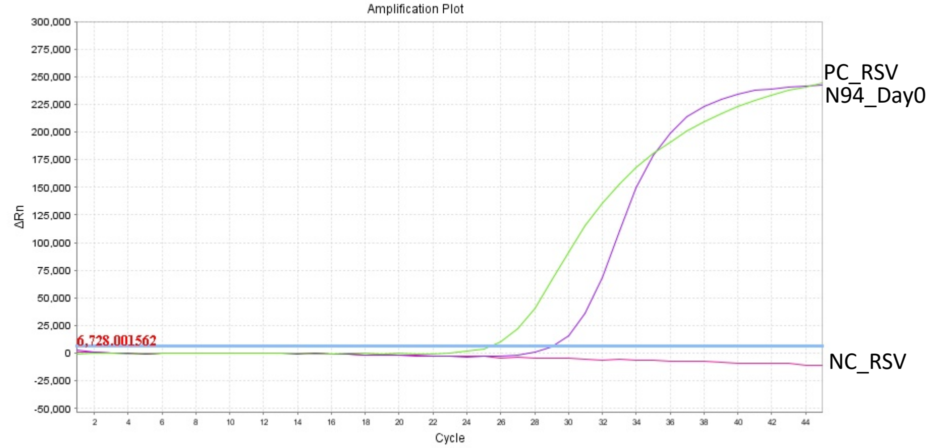
</td><td></td></tr>
<tr><td></td><td>N94_D3</td><td>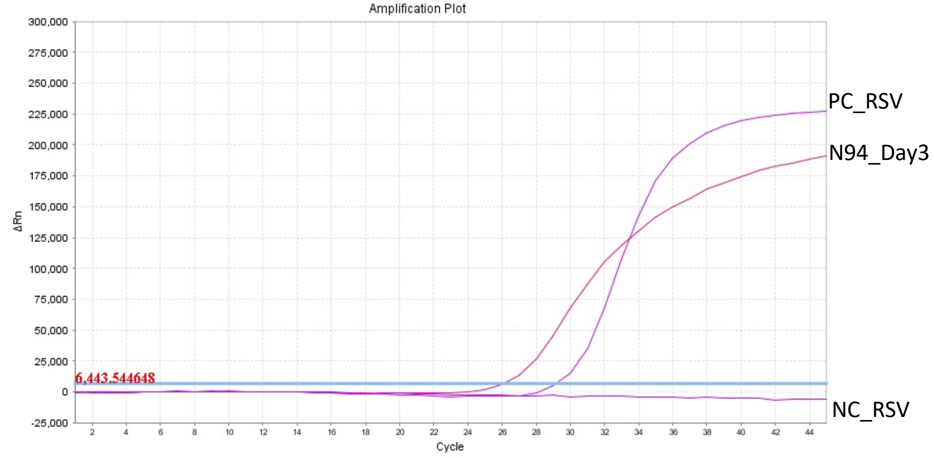
</td><td>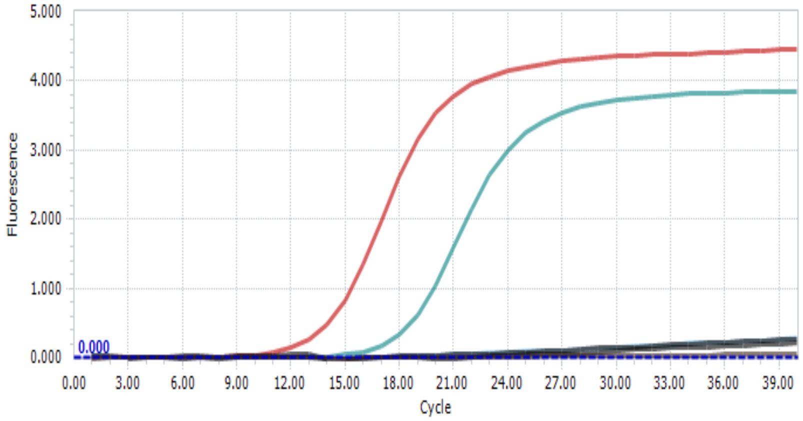
</td></tr>
</table>

| | | | |
|---|---|---|---|
| 37 | N96 _D0 | 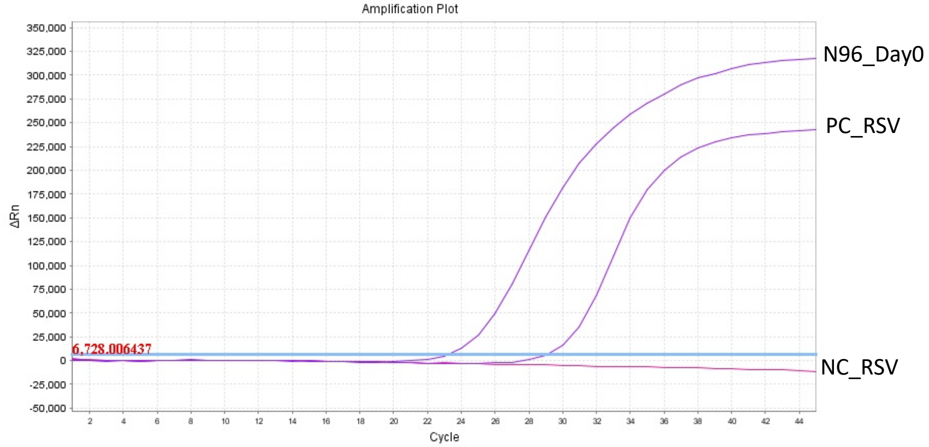 | |
| | N96_D3 | 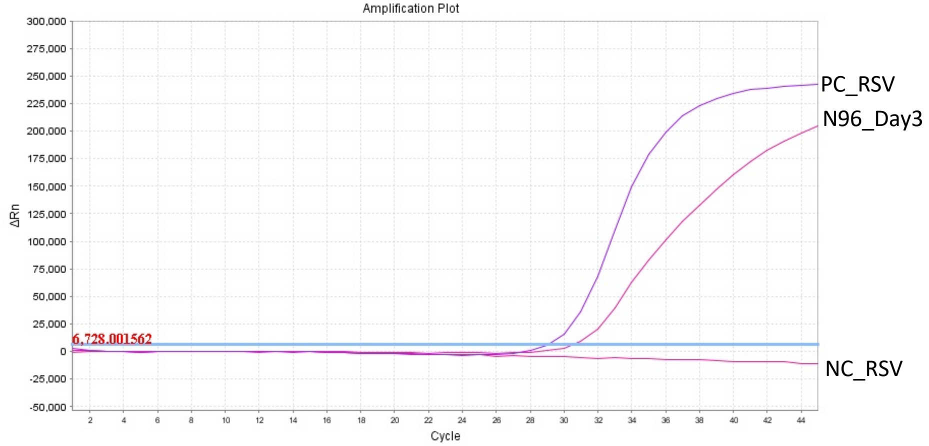 | 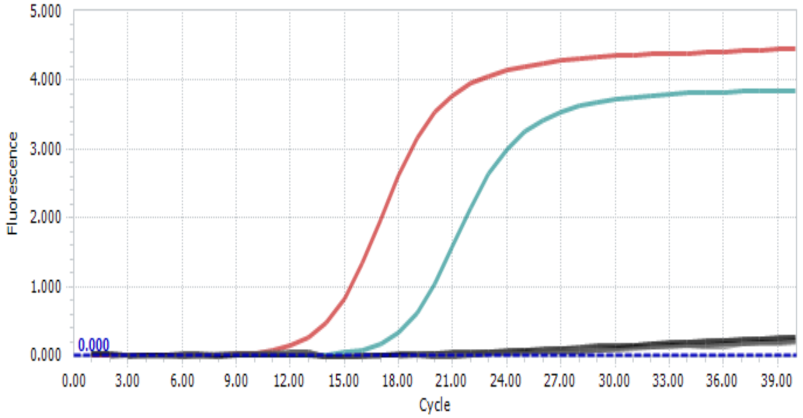 |

<table>
<tr><td rowspan="2">38</td><td>N97 _D0</td><td>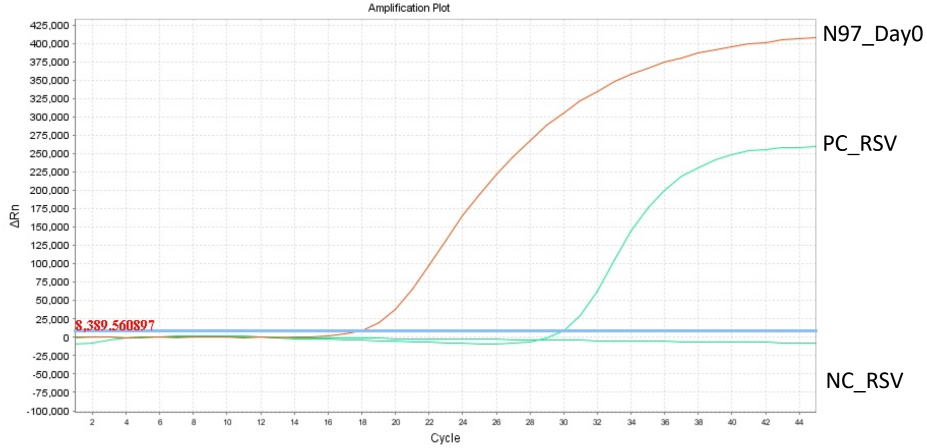
</td><td></td></tr>
<tr><td>N97_D3</td><td>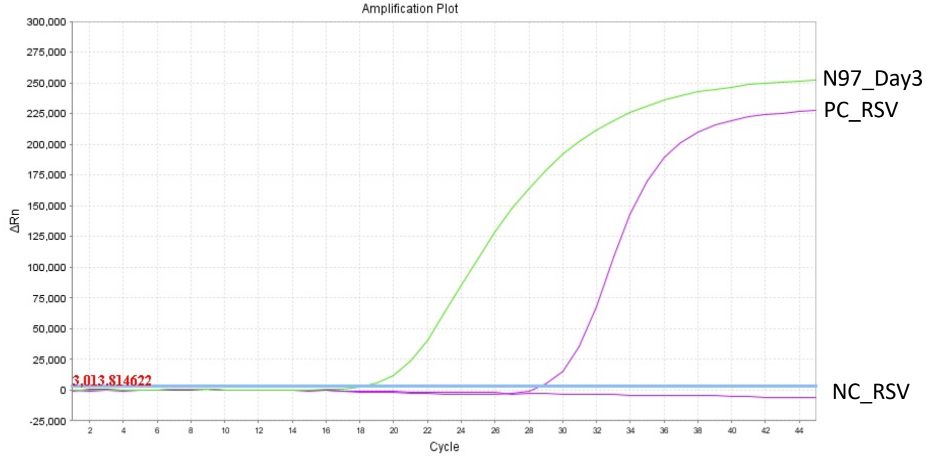
</td><td>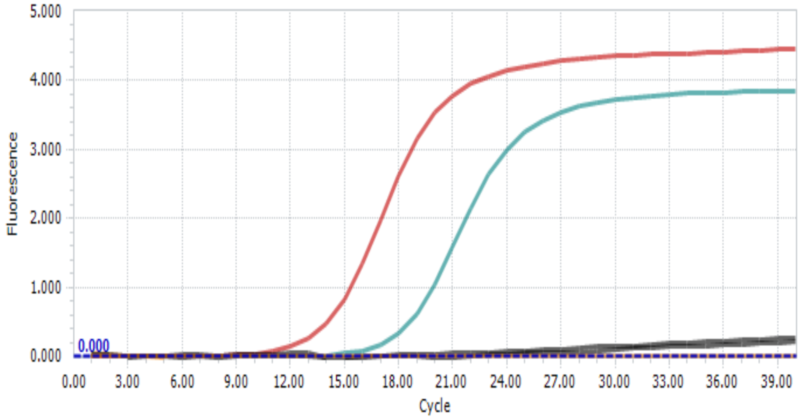
</td></tr>
</table>

<table>
<tr><td rowspan="2">39</td><td>N98 _D0</td><td>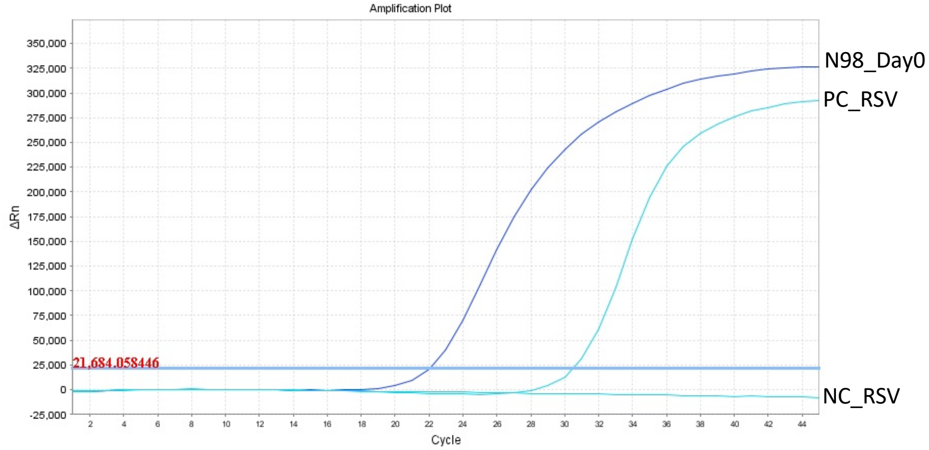
</td><td></td></tr>
<tr><td>N98 _D3</td><td>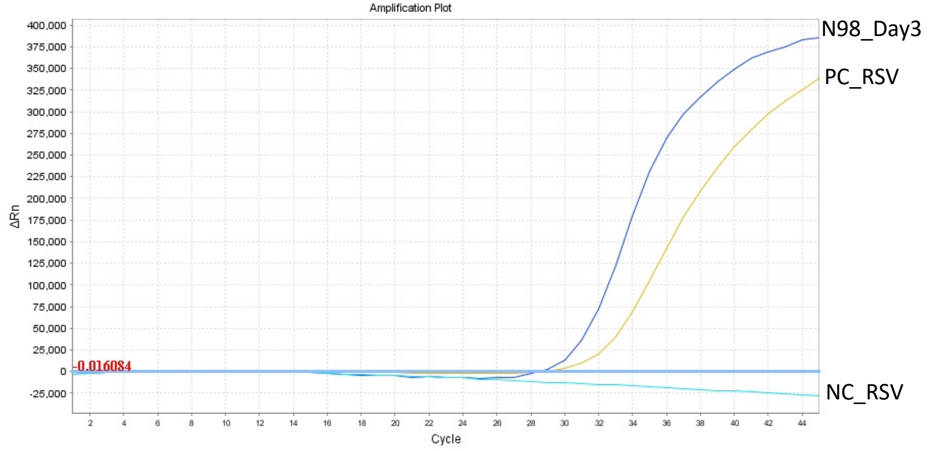
</td><td>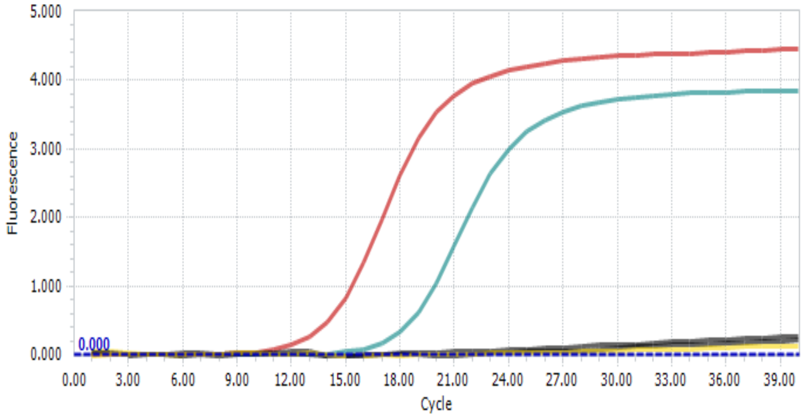
</td></tr>
</table>

<table>
<tr><td rowspan="2">40</td><td>N100_D0</td><td>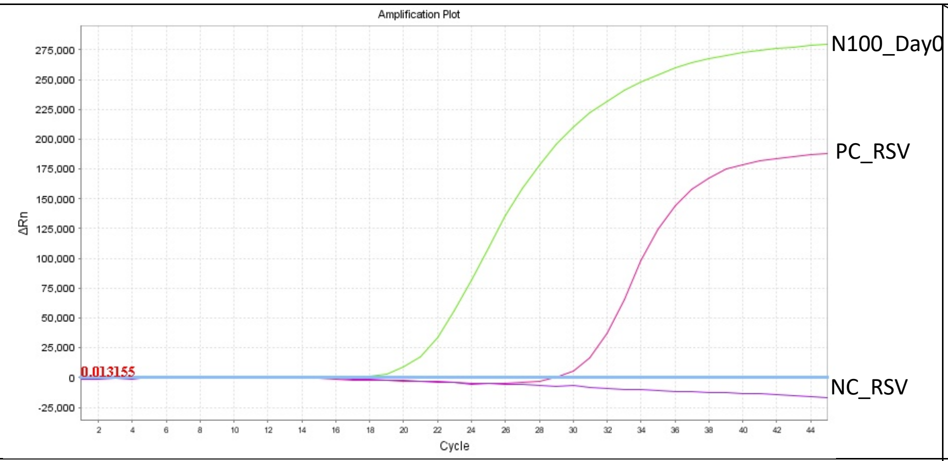
</td><td></td></tr>
<tr><td>N100_D3</td><td>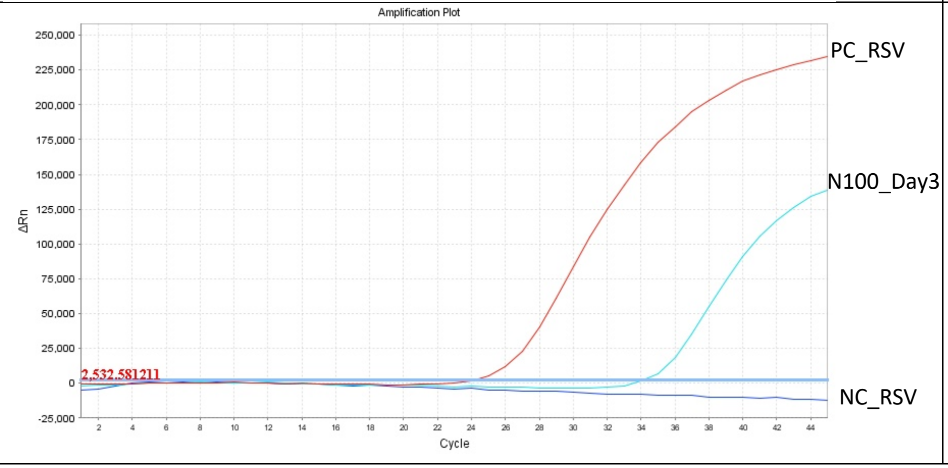
</td><td>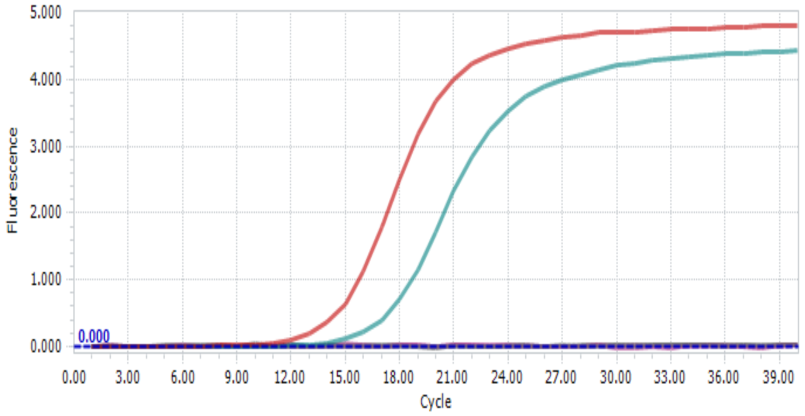
</td></tr>
</table>

**Fig. S1.** A. Real-time PCR TaqMan probes amplification curves specifically for RSV taken from nasopharyngeal samples of Control group at day 0 and 3 of treatment. PC, NC are positive and negative controls of RSV; B. SYBR Green real-time PCR amplification curves specifically for *B. subtilis* and *B. clausii* was either non-detectable or with $C_t \geq 37$ in nasopharyngeal samples of Control groups at day 3 of treatment. PC are positive controls of *B. subtilis* (red line) and *B. clausii* (blue line), NC (black) are negative controls of *B. subtilis* and *B. clausii*. By lottery, the patient's number is coded at random. The sequence of the image's appearance in the figure corresponds to the order of the patient's hospitalization.
