# Supplementary material for: Nasal-spraying Bacillus spores as an effective symptomatic treatment for children with acute respiratory syncytial virus infection
Source: Sci Rep. 2022 Jul 20;12:12402. doi: 10.1038/s41598-022-16136-z (PMC9297280; doi:10.1038/s41598-022-16136-z)
Supplement: Supplementary file 2 — Supplementary Information 2. [file 41598_2022_16136_MOESM2_ESM.pdf]

Supplemental Fig. S2

Real time PCR curves of RSV, *B. subtilis,* and *B. clausii* of nasopharyngeal samples in Navax group

No	Patient code	A. RSV	B. *B. subtilis & B. clausii*<br>Red —Positive Control (PC) of *B. subtilis*<br>Blue —PC of *B. clausii*<br>Black —Negative control (NC) of *B. subtilis*, NC of *B. clausii*<br>Other colors: samples with positive signals (S: *B. subtilis*; C: *B. clausii)*
1	N01_D0	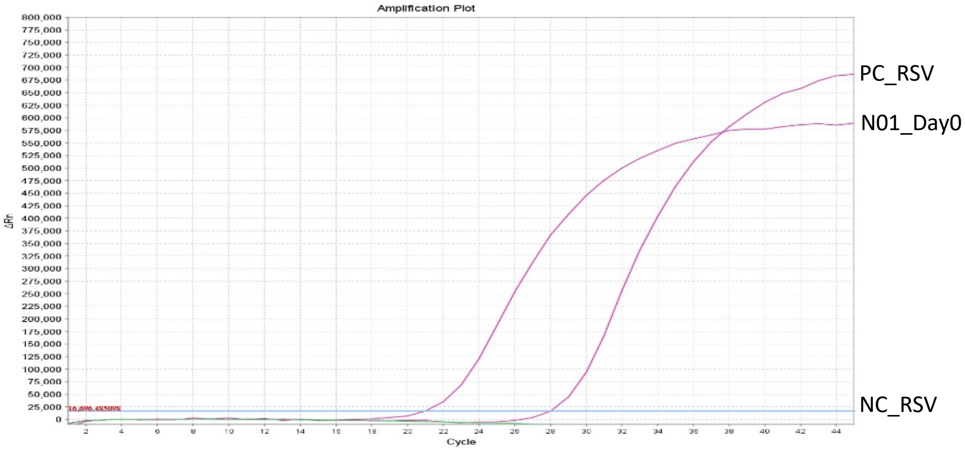	
	N01_D3	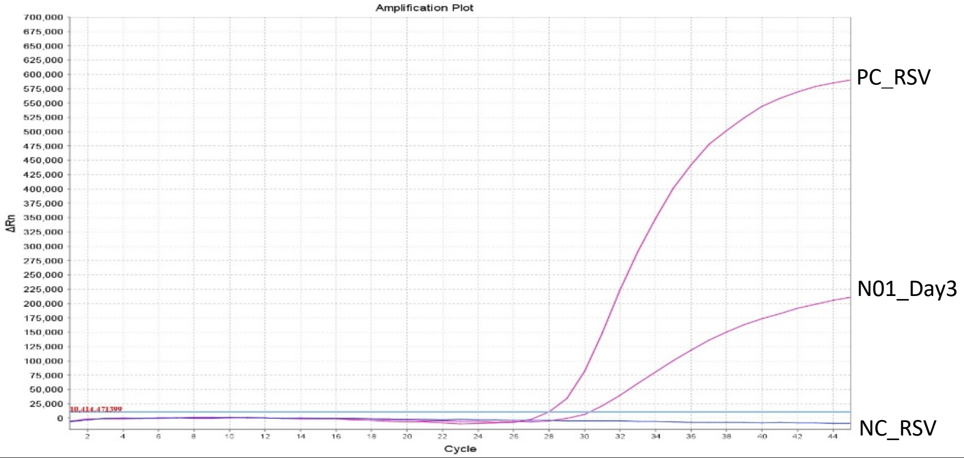	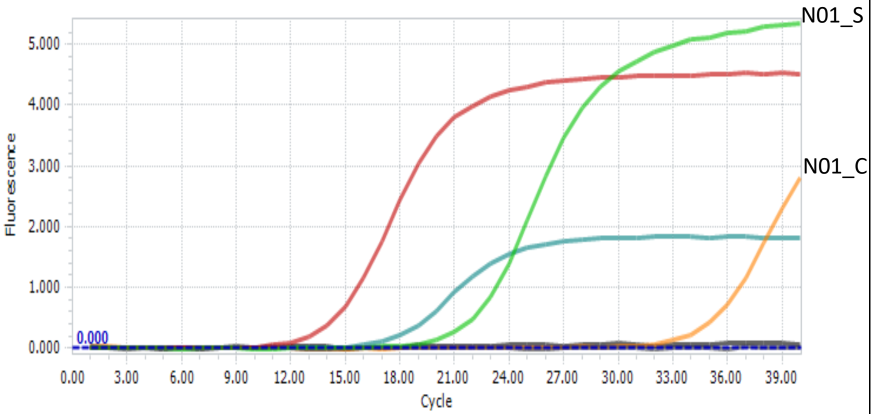

<table>
<tr><td>2</td><td>N03_D0</td><td>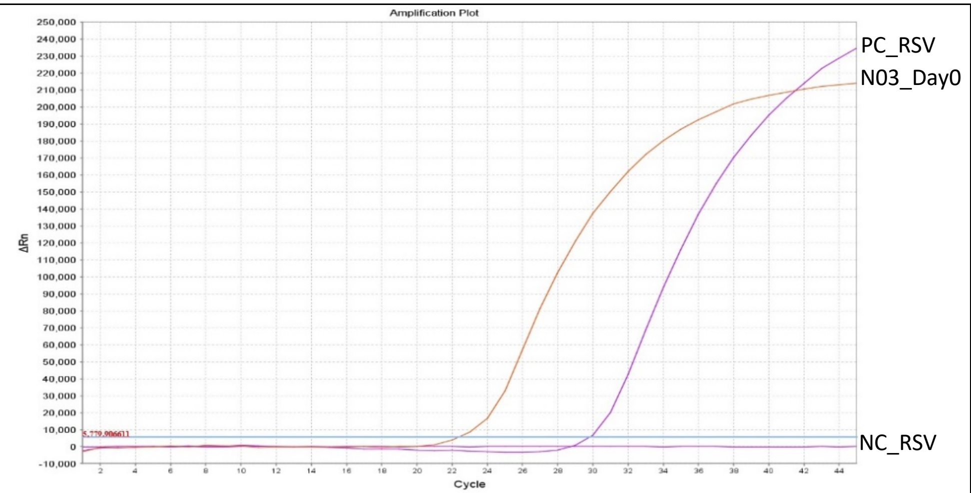
</td><td></td></tr>
<tr><td></td><td>N03_D3</td><td>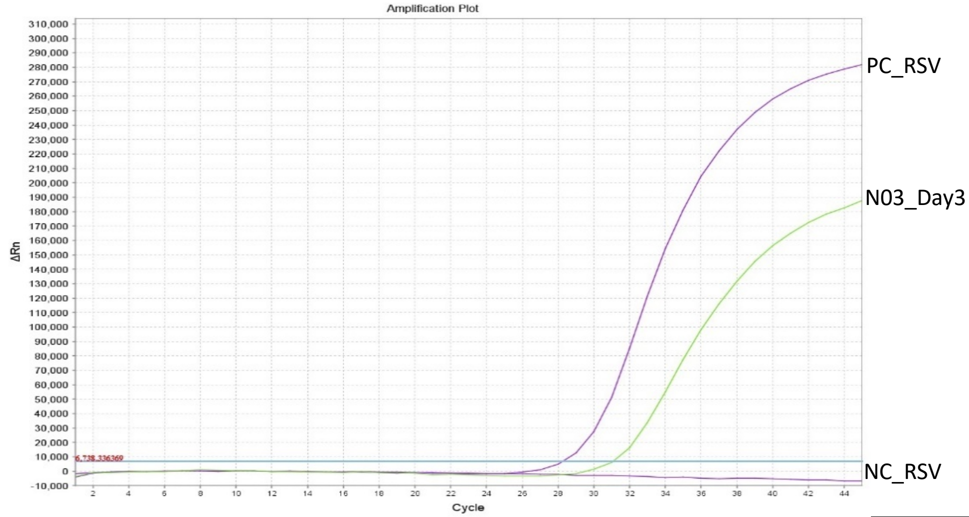
</td><td>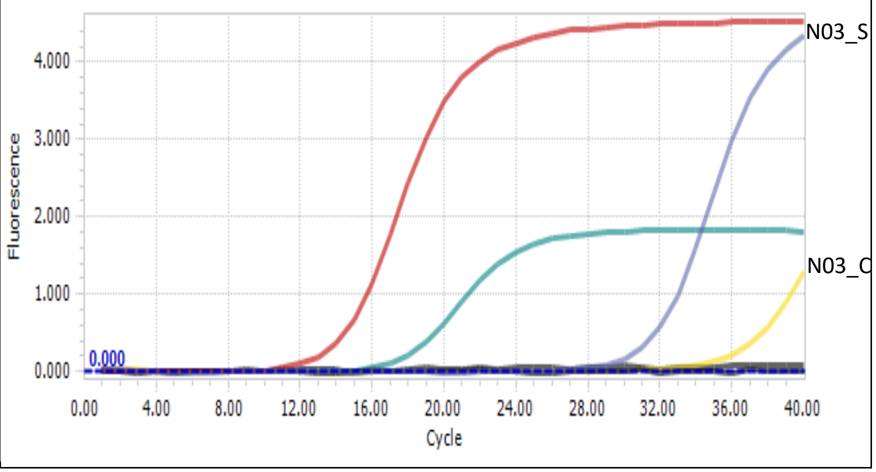
</td></tr>
</table>

<table>
<tr><td rowspan="2">3</td><td>N06_D0</td><td>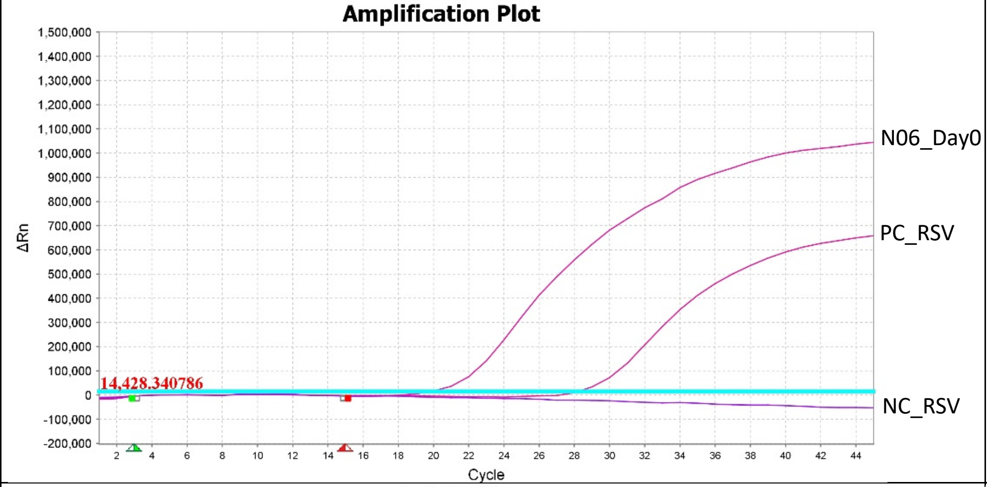
</td><td></td></tr>
<tr><td>N06_D3</td><td>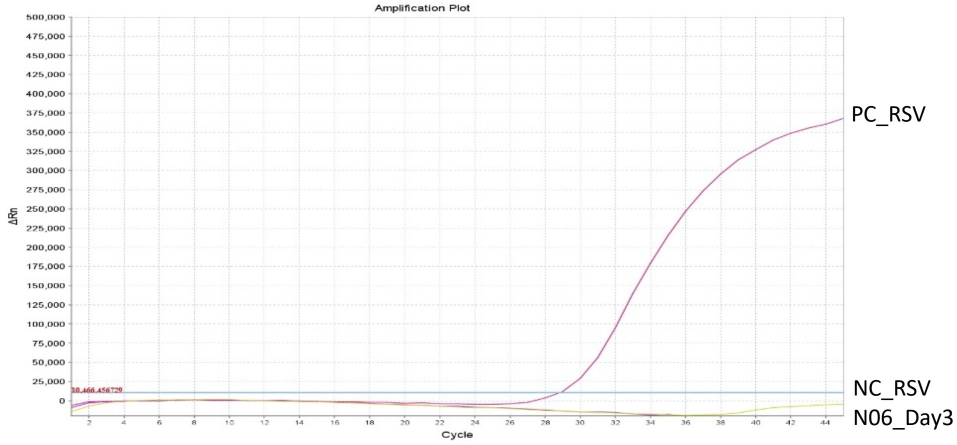
</td><td>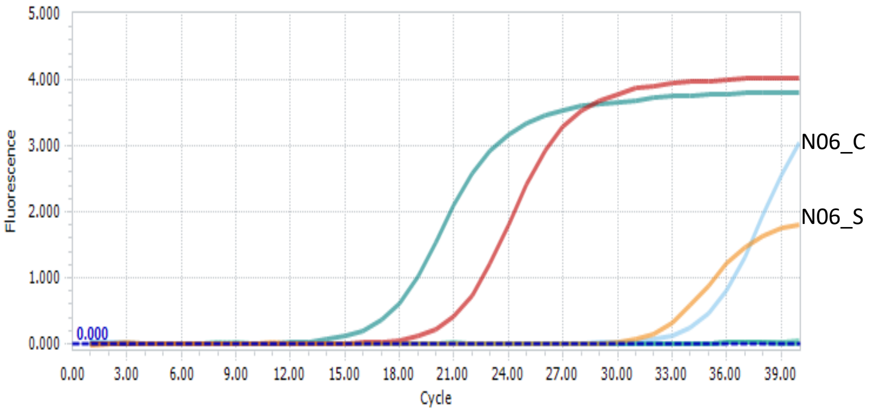
</td></tr>
</table>

<table>
<tr><td rowspan="2">4</td><td>N07_D0</td><td>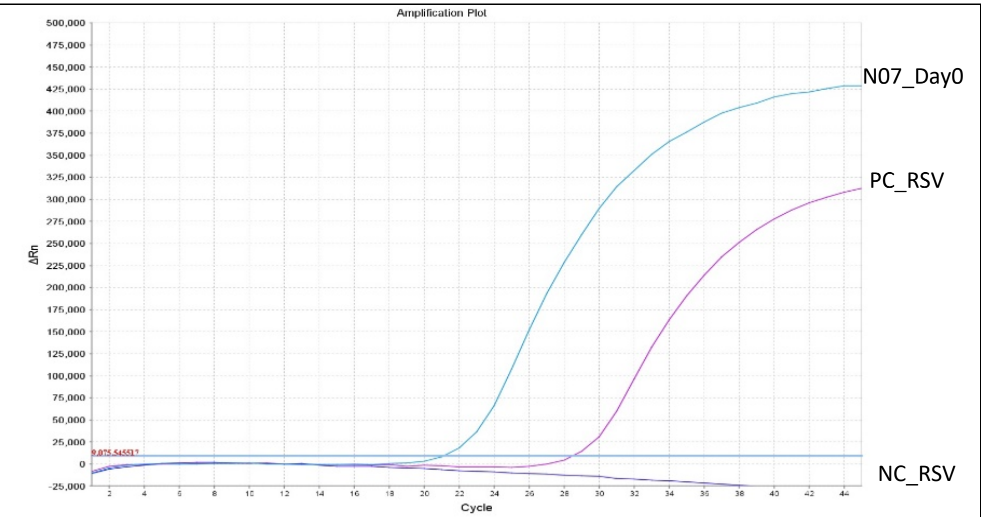
</td><td></td></tr>
<tr><td>N07_D3</td><td>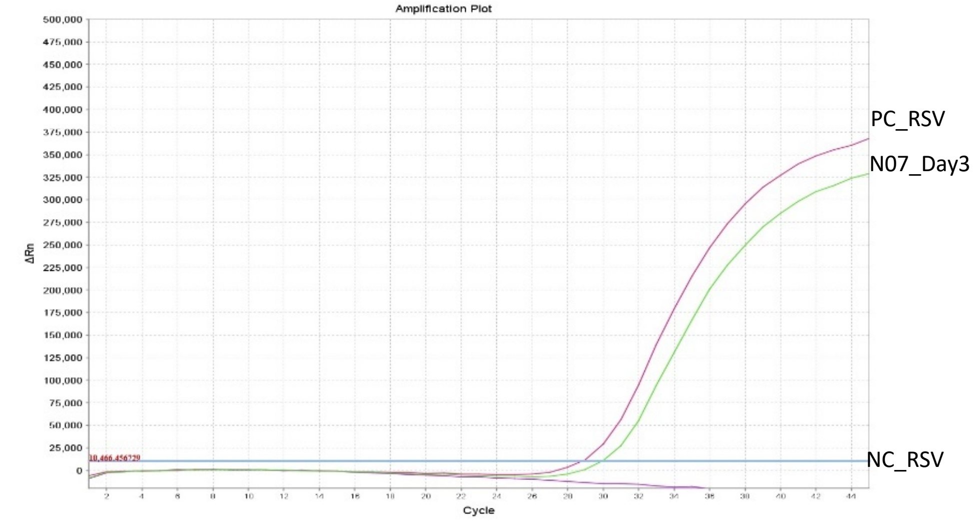
</td><td>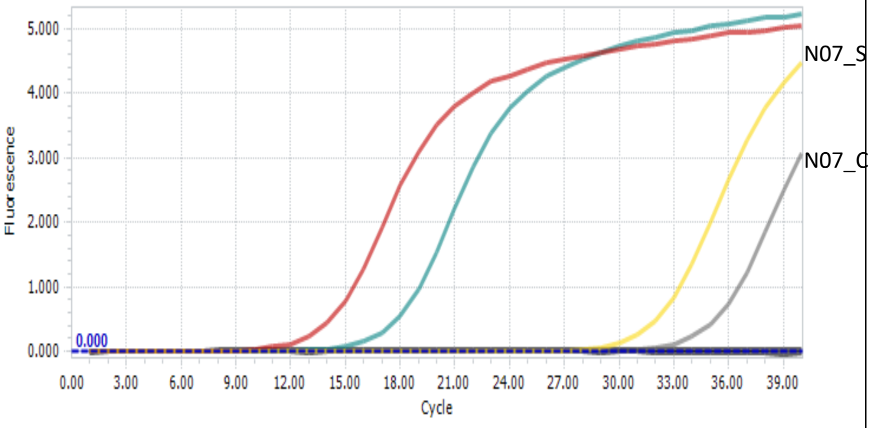
</td></tr>
</table>

5	N09_D0	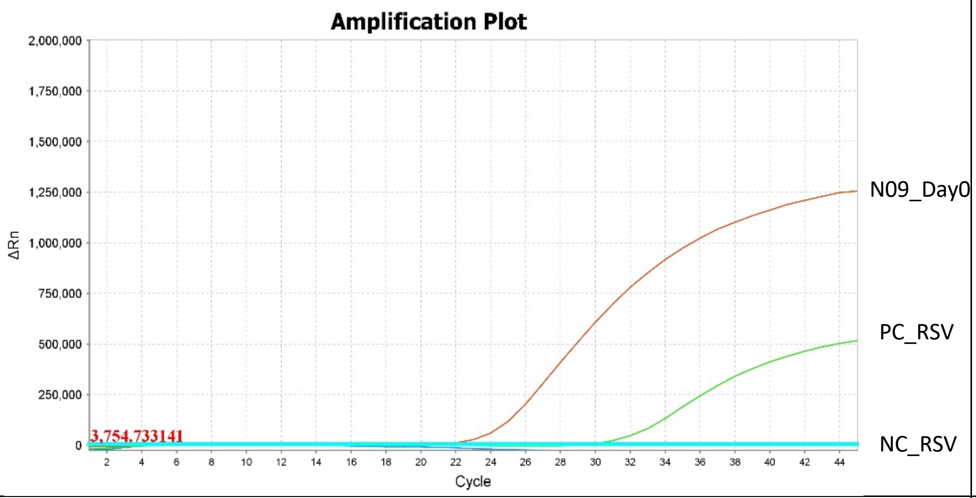	
	N09_D3	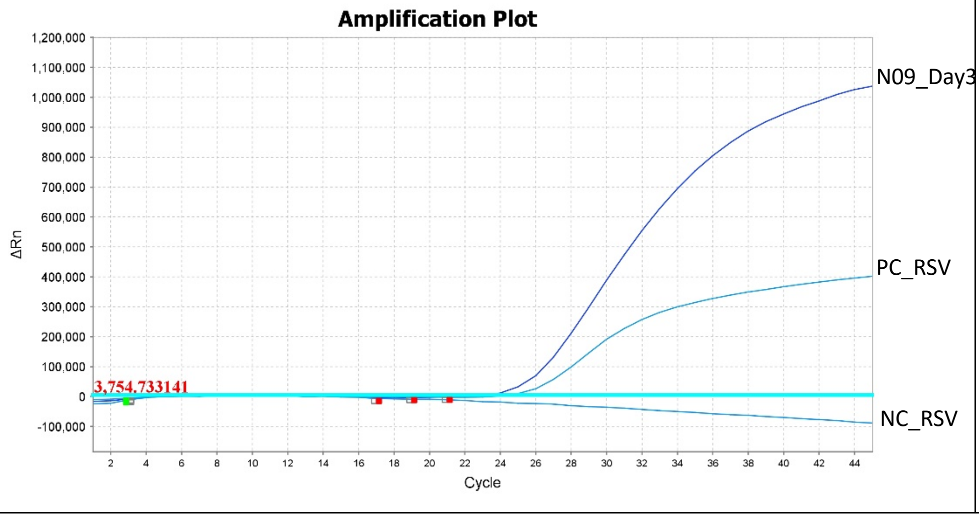	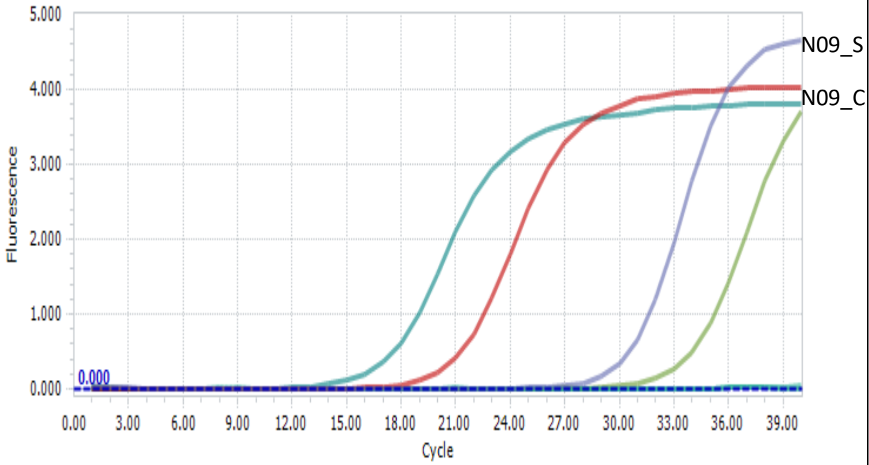

<table>
<tr><td rowspan="2">6</td><td>N12_D0</td><td>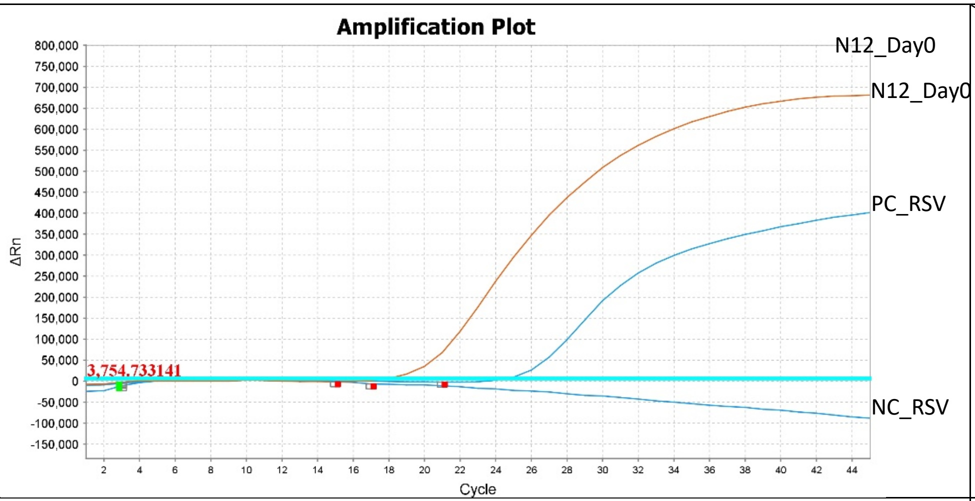
</td><td></td></tr>
<tr><td>N12_D3</td><td>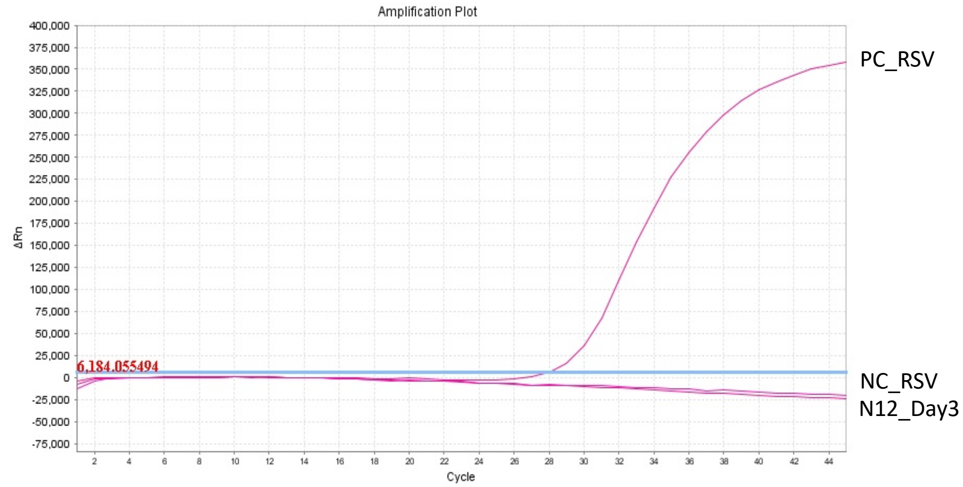
</td><td>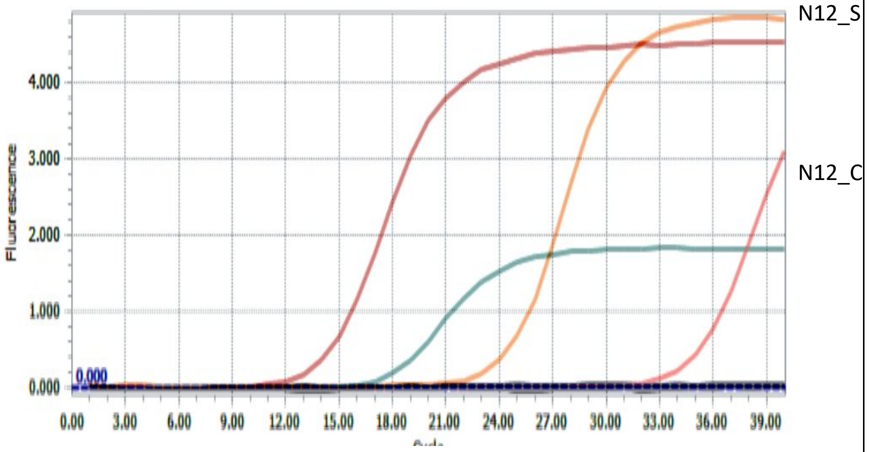
</td></tr>
</table>

<table>
<tr><td>7</td><td>N13_D0</td><td>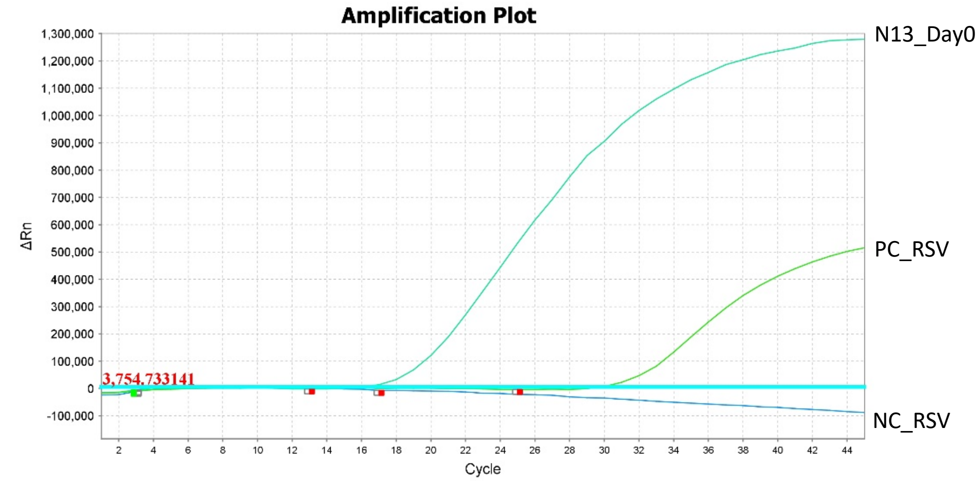
</td><td></td></tr>
<tr><td></td><td>N13_D3</td><td>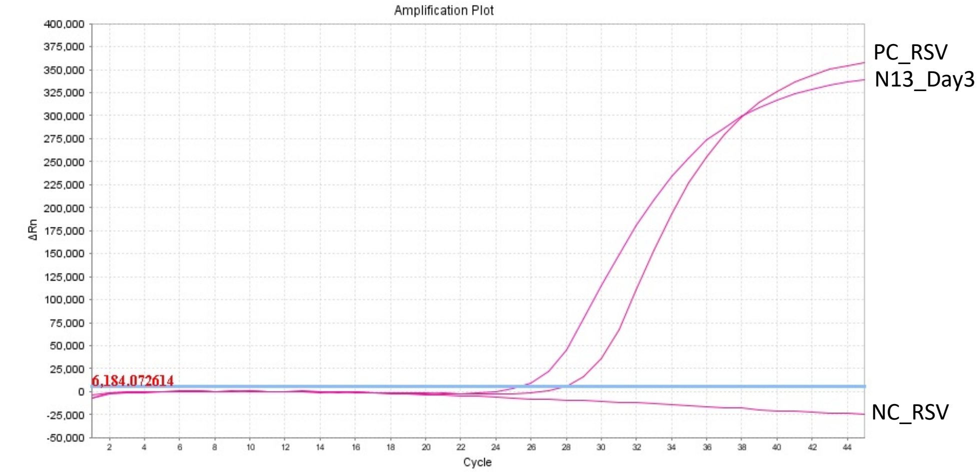
</td><td>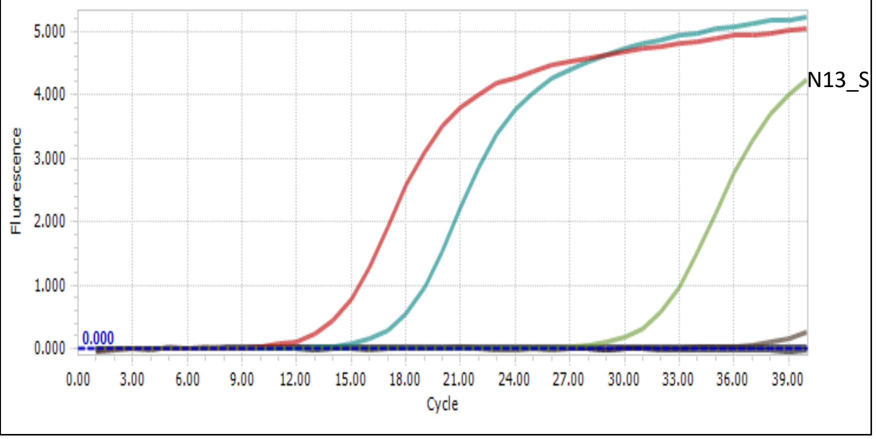
</td></tr>
</table>

<table>
<tr><td rowspan="2">8</td><td>N15_D0</td><td>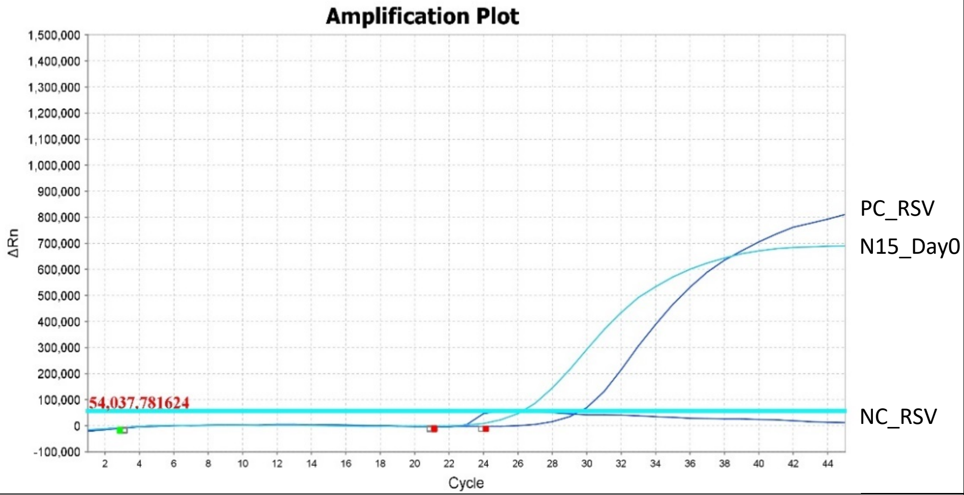
</td><td></td></tr>
<tr><td>N15_D3</td><td>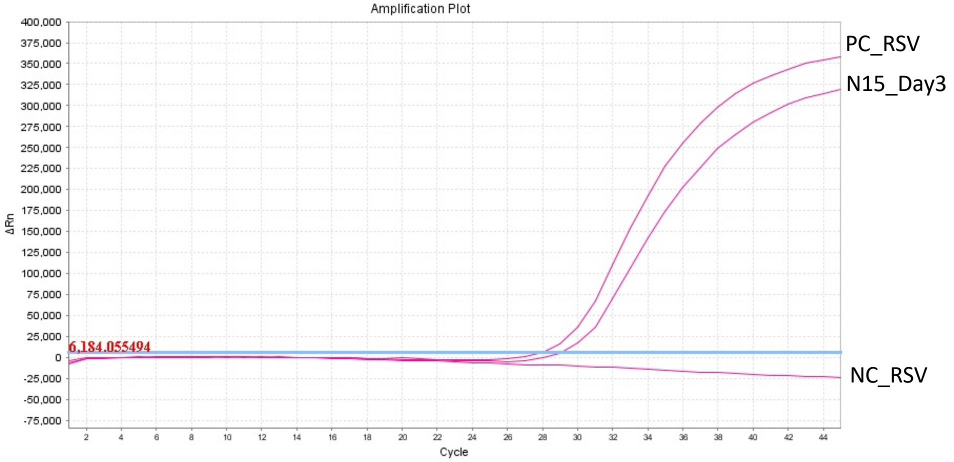
</td><td>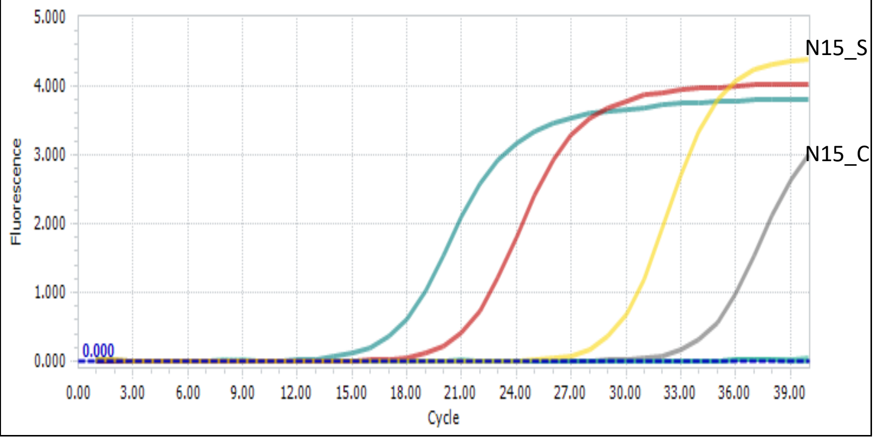
</td></tr>
</table>

<table>
<tr><td>9</td><td>N16_D0</td><td>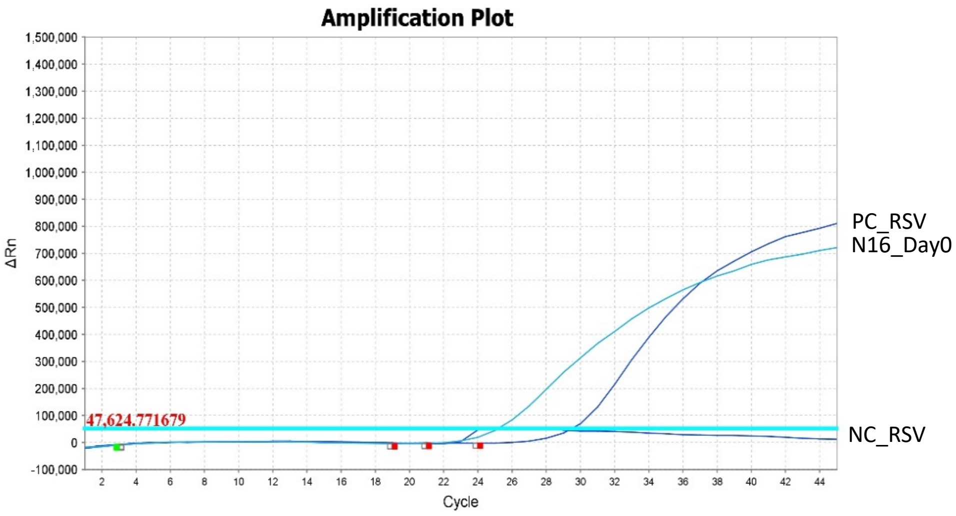
</td><td></td></tr>
<tr><td></td><td>N16_D3</td><td>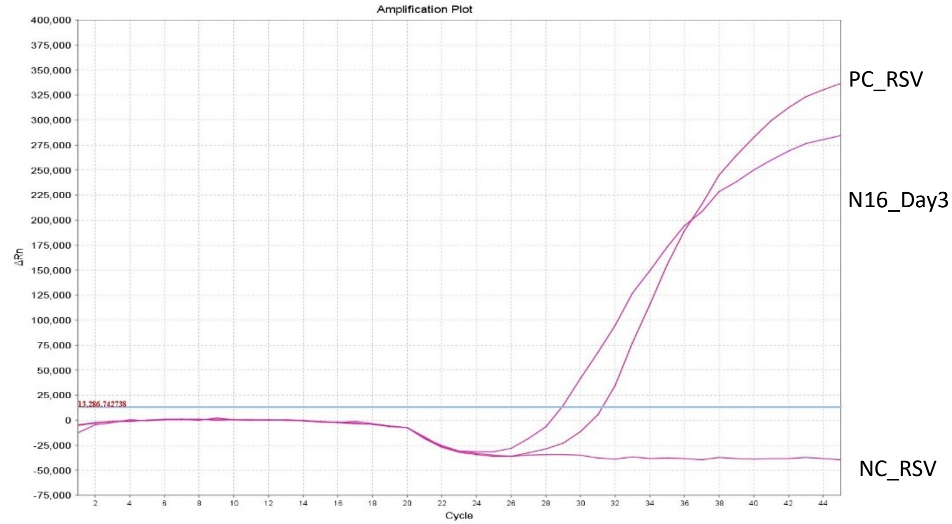
</td><td>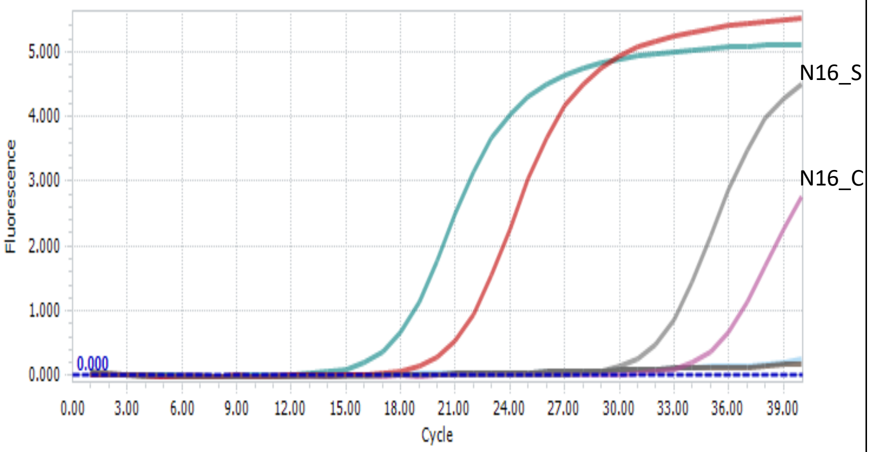
</td></tr>
</table>

<table>
<tr><td>10</td><td>N17_D0</td><td>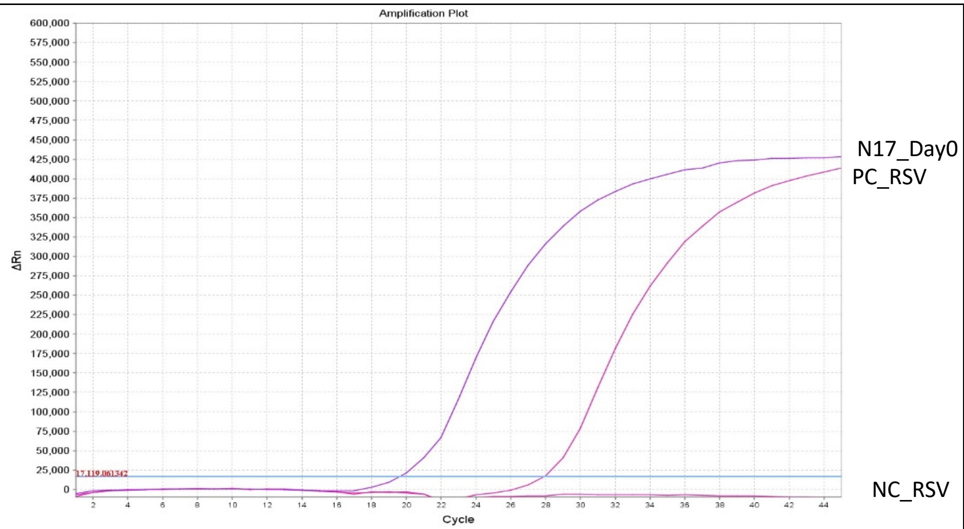
</td><td></td></tr>
<tr><td></td><td>N17_D3</td><td>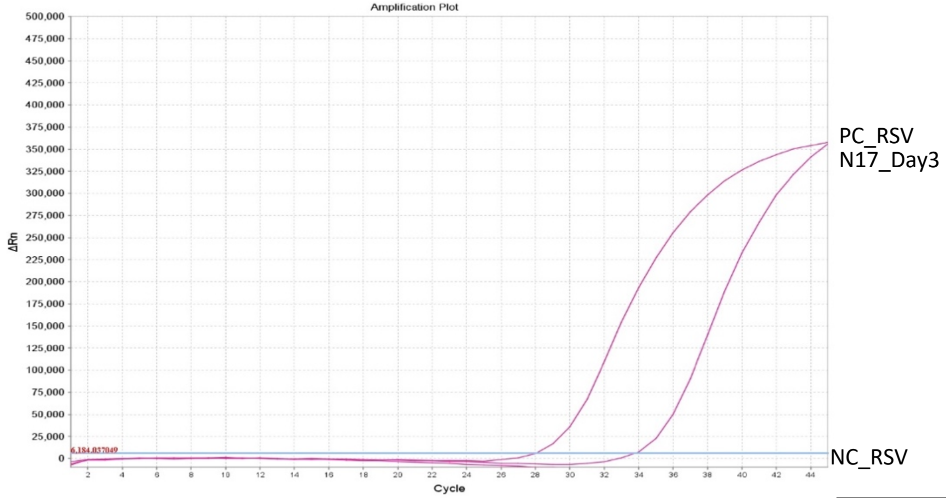
</td><td>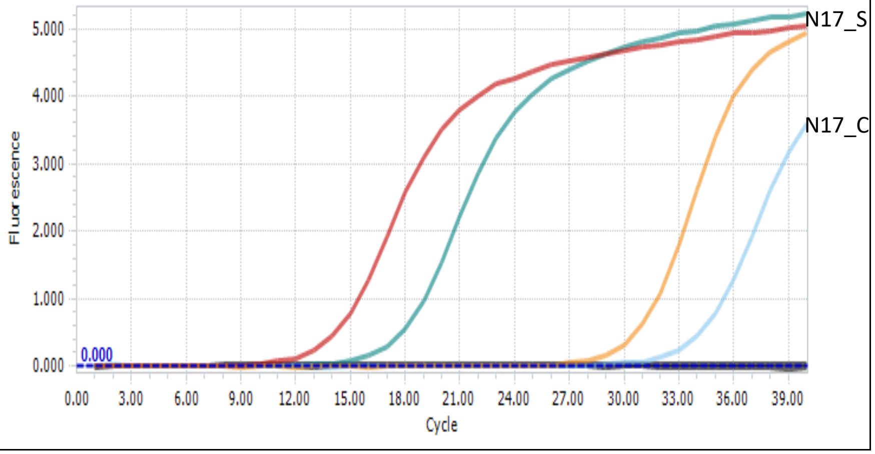
</td></tr>
</table>

11	N19_D0	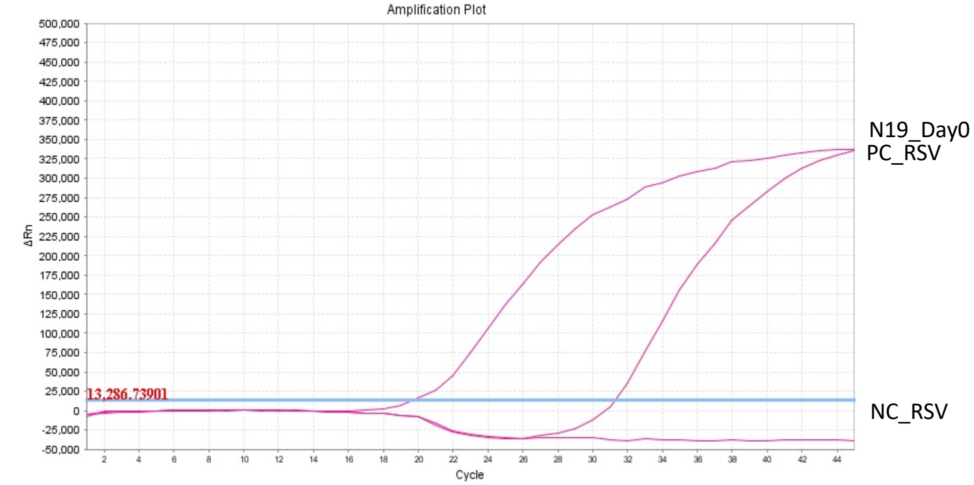	
	N19_D3	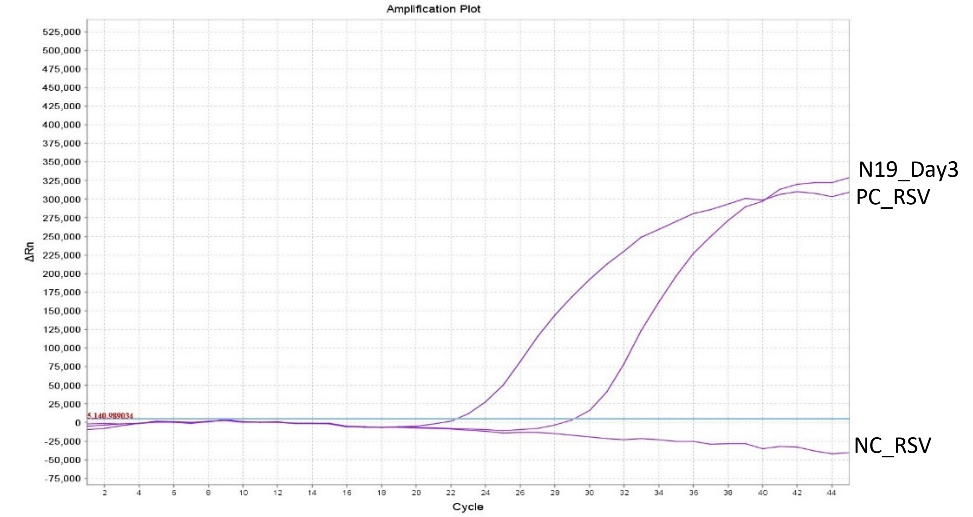	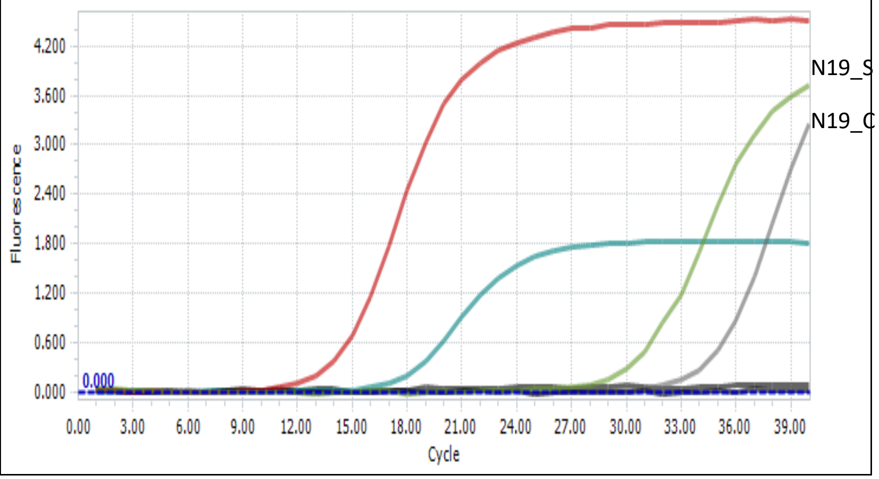

12	N21_D0	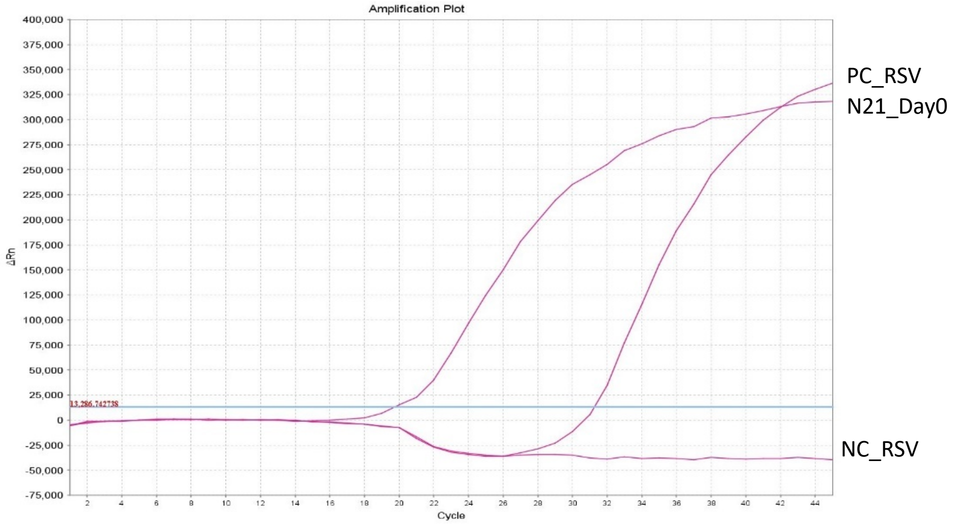	
	N21_D3	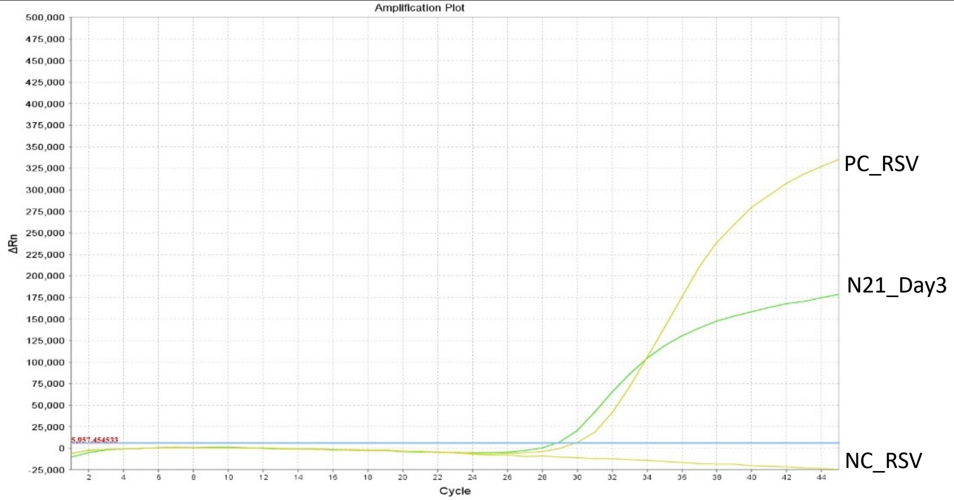	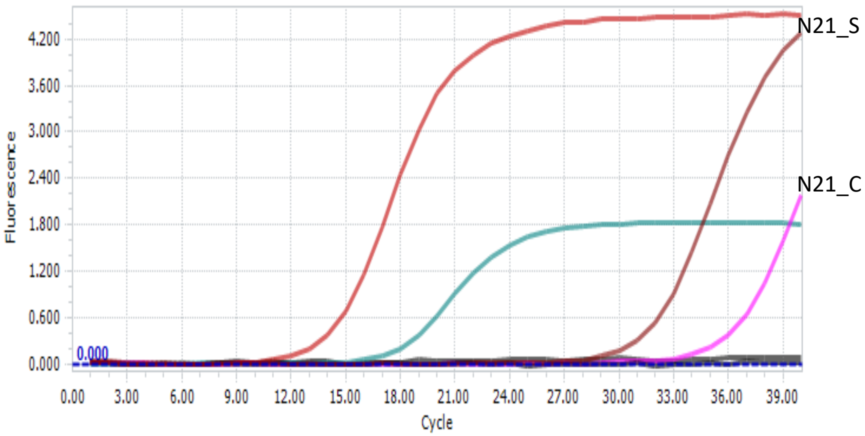

<table>
<tr>
<td>13</td>
<td>N25_D0</td>
<td>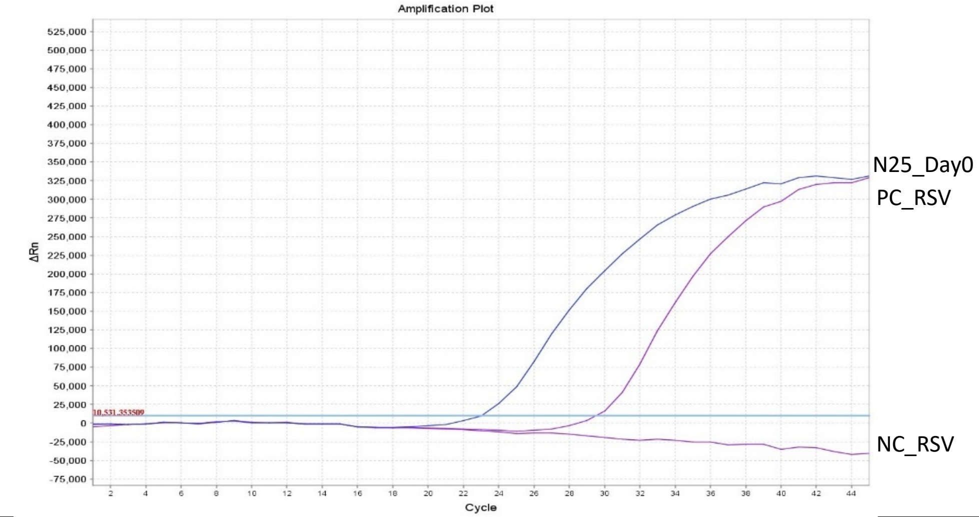
</td>
<td></td>
</tr>
<tr>
<td></td>
<td>N25_D3</td>
<td>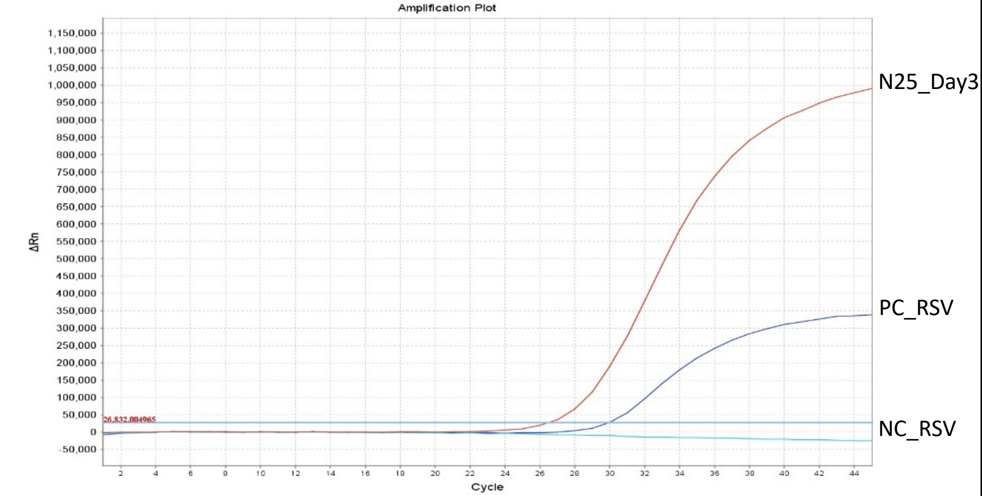
</td>
<td>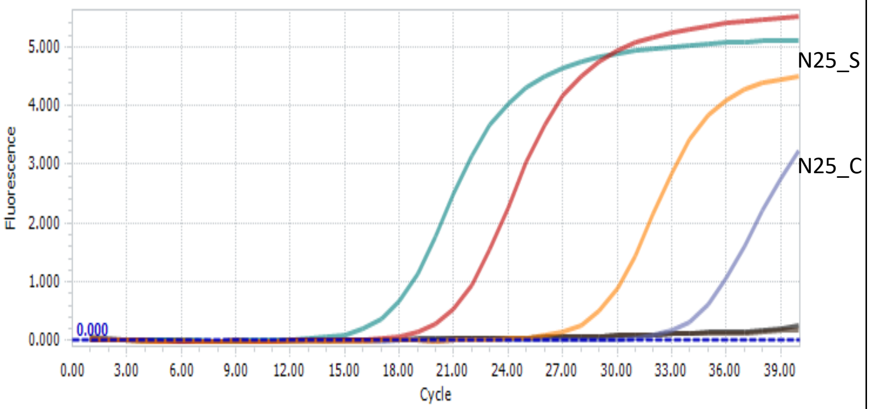
</td>
</tr>
</table>

14	N26_D0	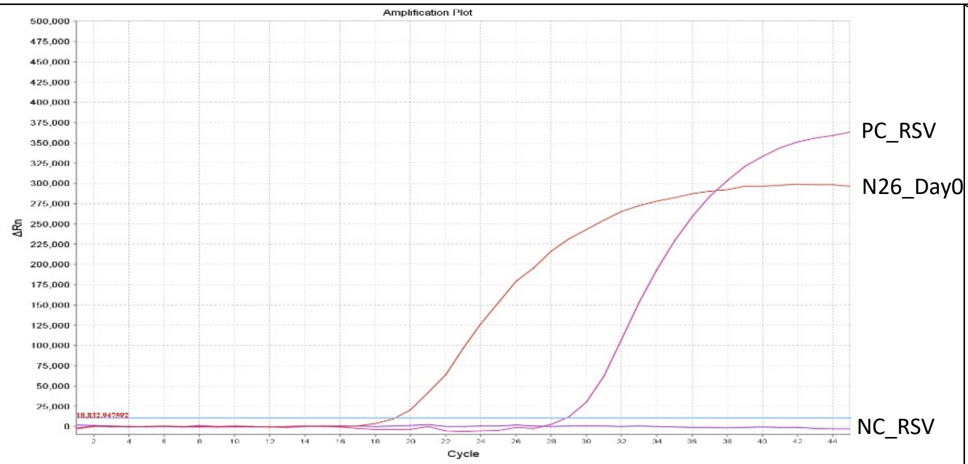	
	N26_D3	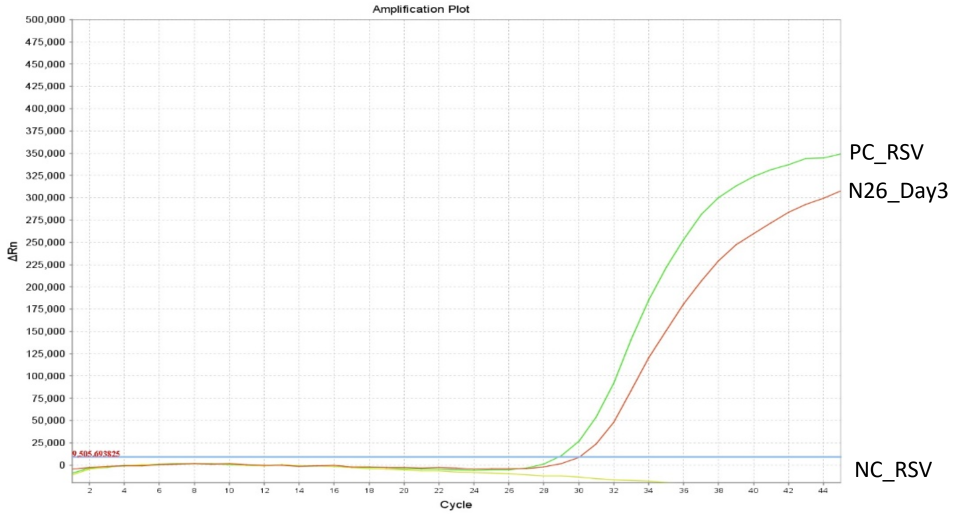	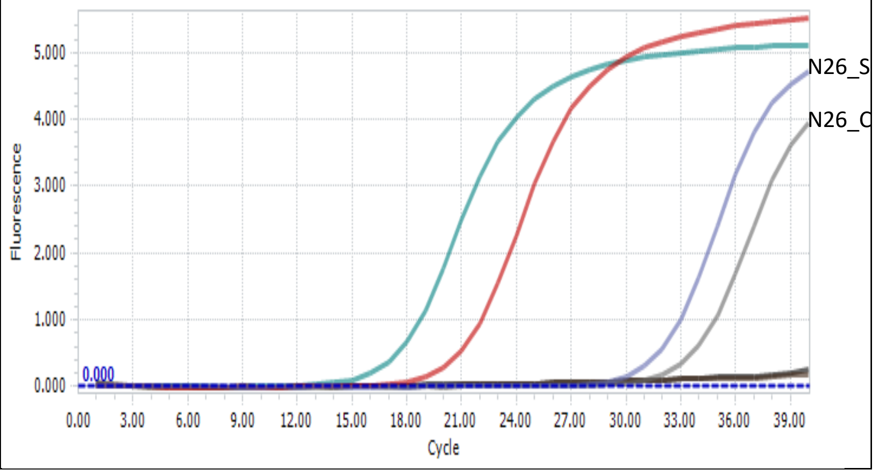

<table>
<tr><td>15</td><td>N31_D0</td><td>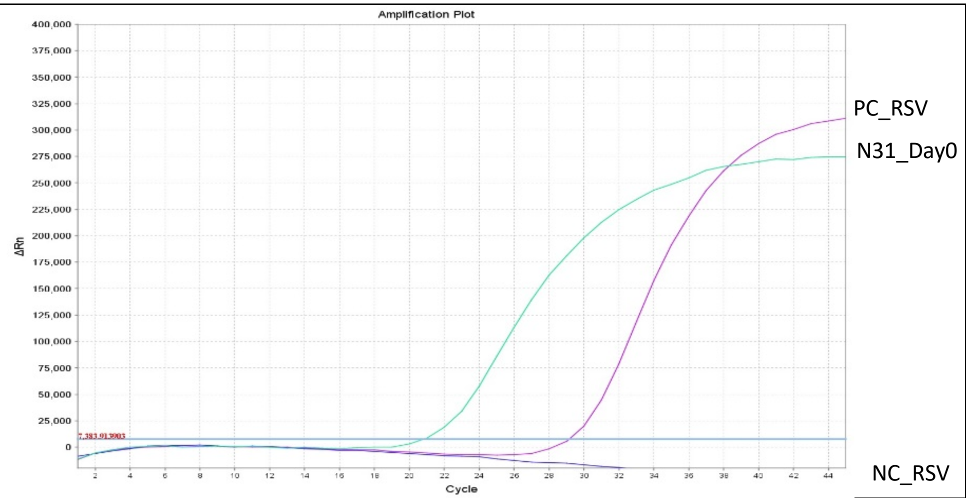
</td><td></td></tr>
<tr><td></td><td>N31_D3</td><td>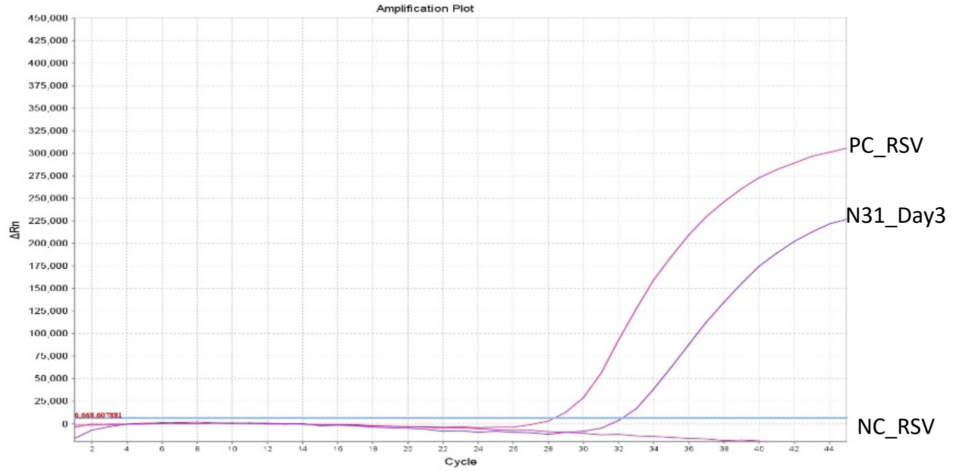
</td><td>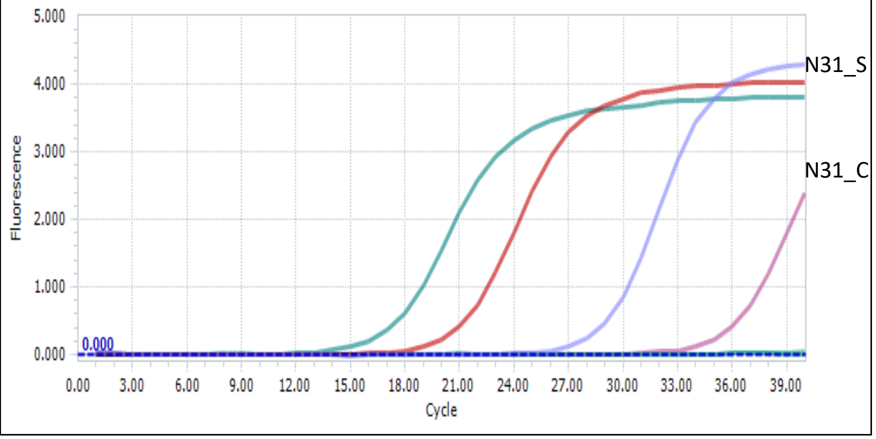
</td></tr>
</table>

<table>
<tr><td>16</td><td>N32_D0</td><td>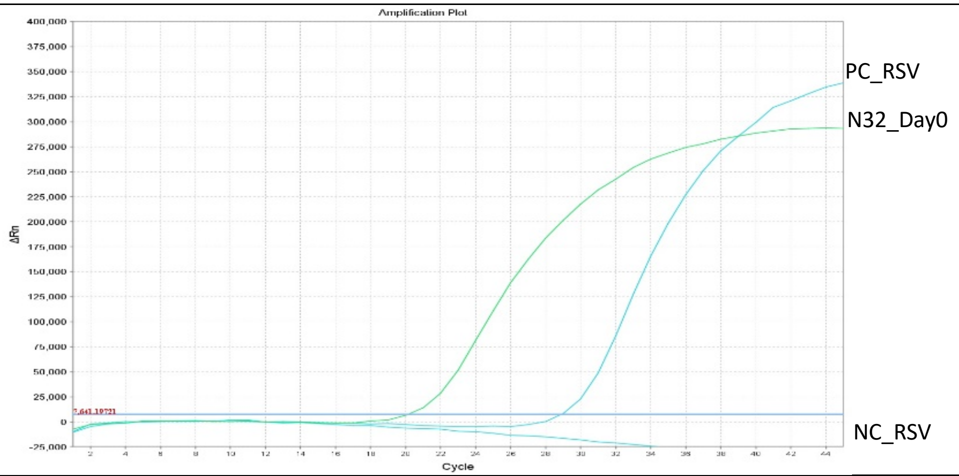
</td><td></td></tr>
<tr><td></td><td>N32_D3</td><td>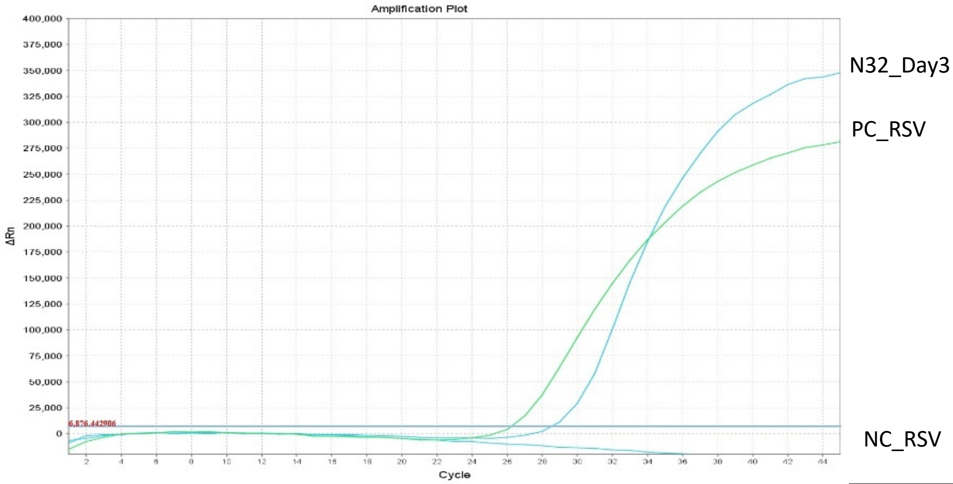
</td><td>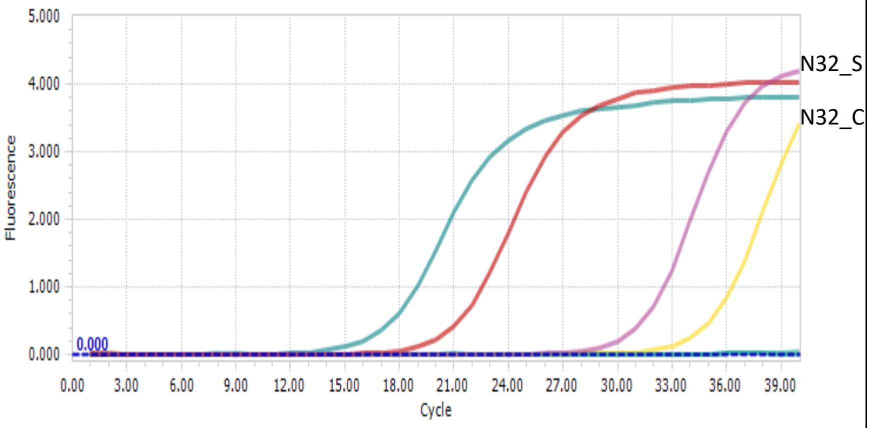
</td></tr>
</table>

17	N34_D0	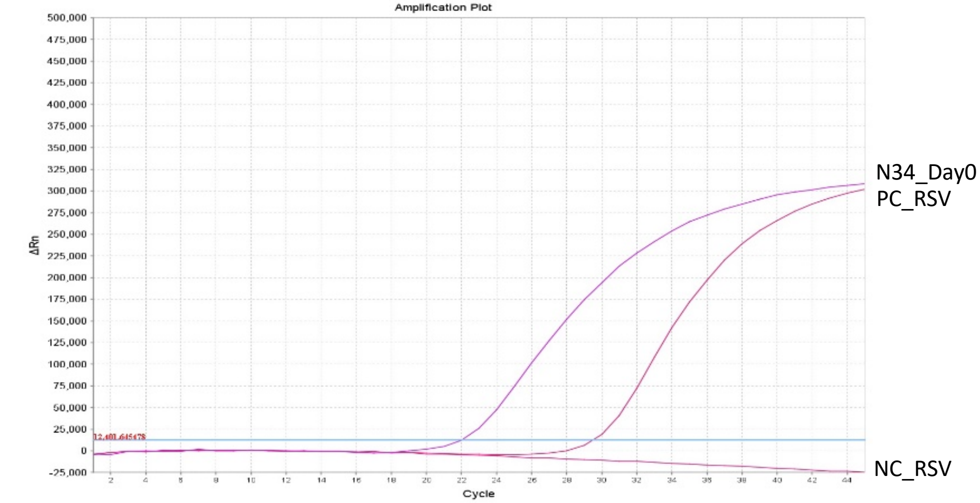	
	N34_D3	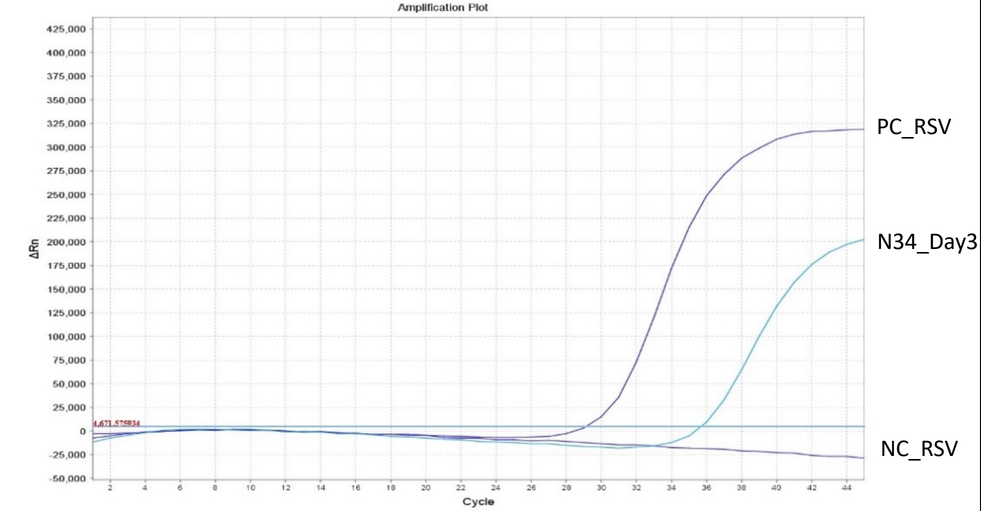	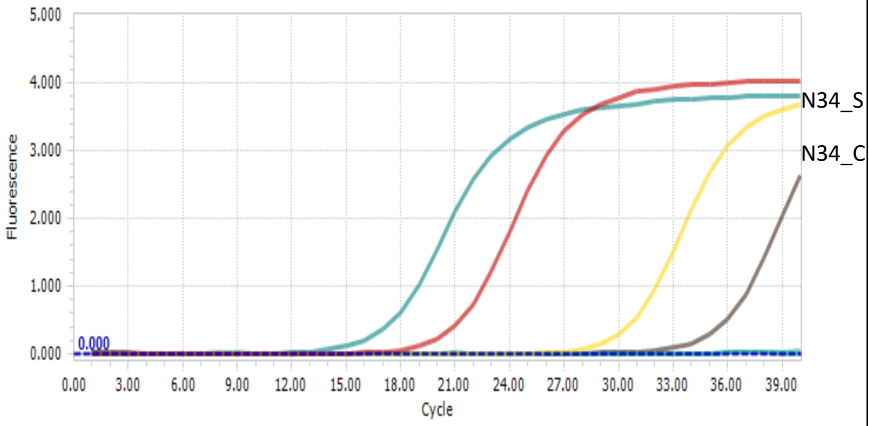

<table>
<tr><td rowspan="2">18</td><td>N37_D0</td><td>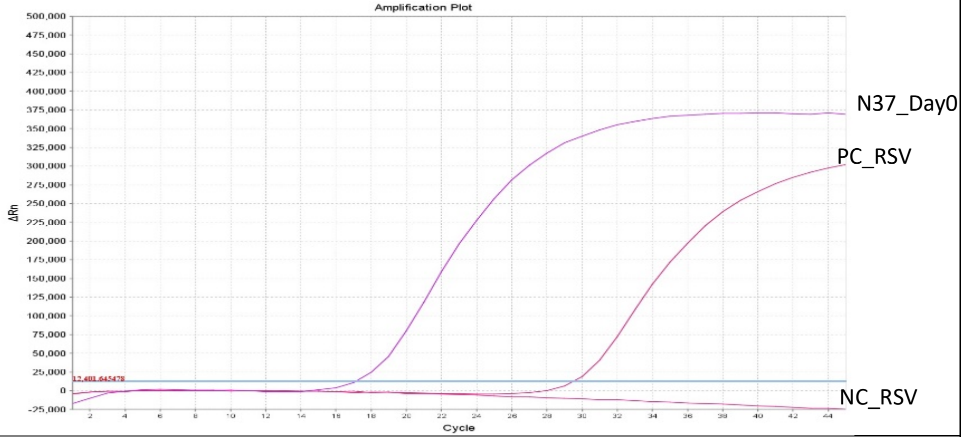
</td><td></td></tr>
<tr><td>N37_D3</td><td>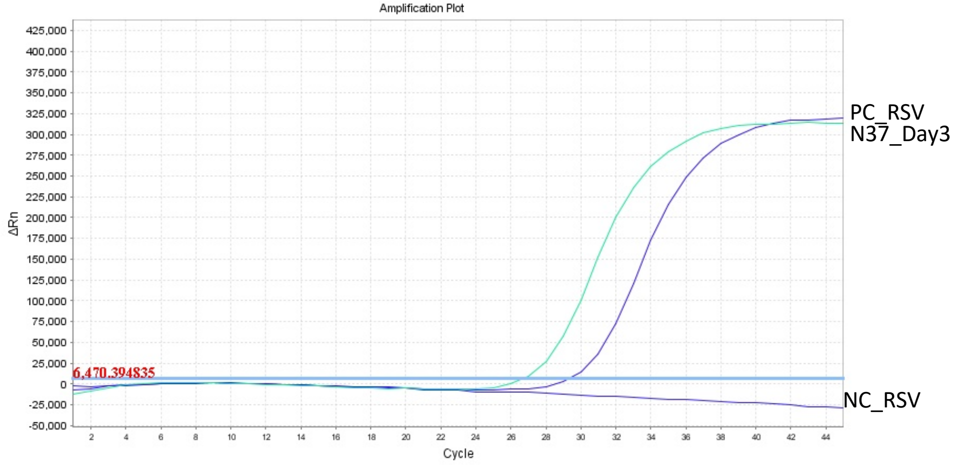
</td><td>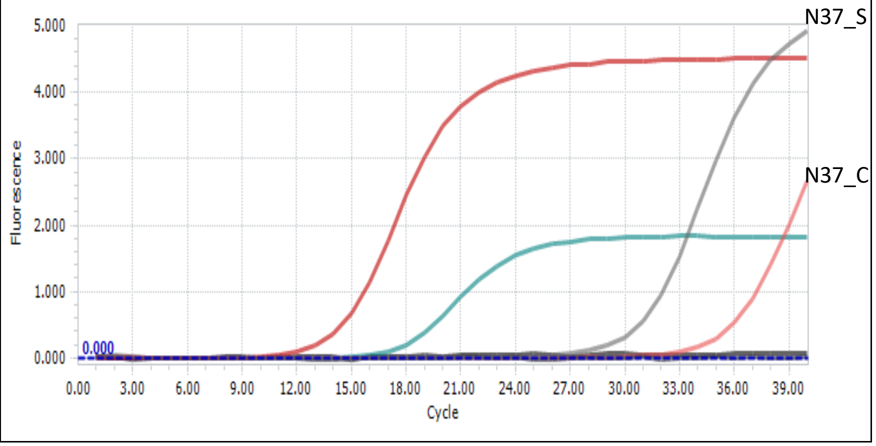
</td></tr>
</table>

19	N39_D3	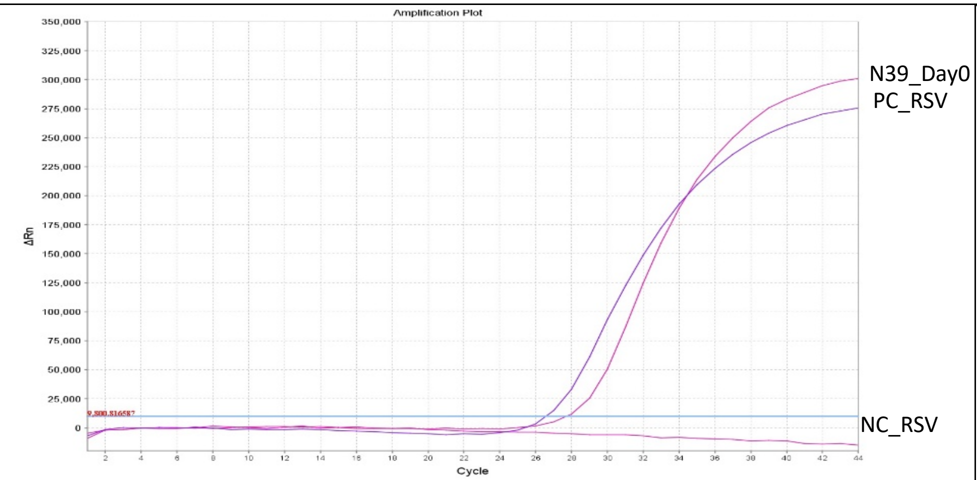	
	N39_D3	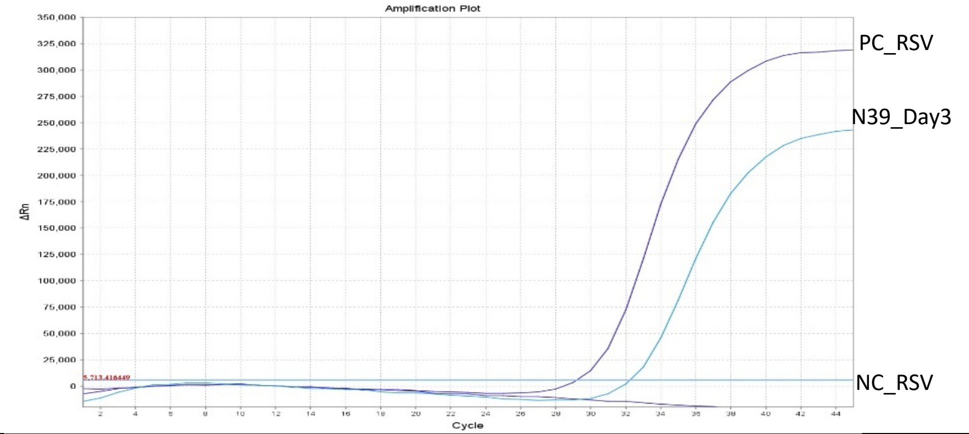	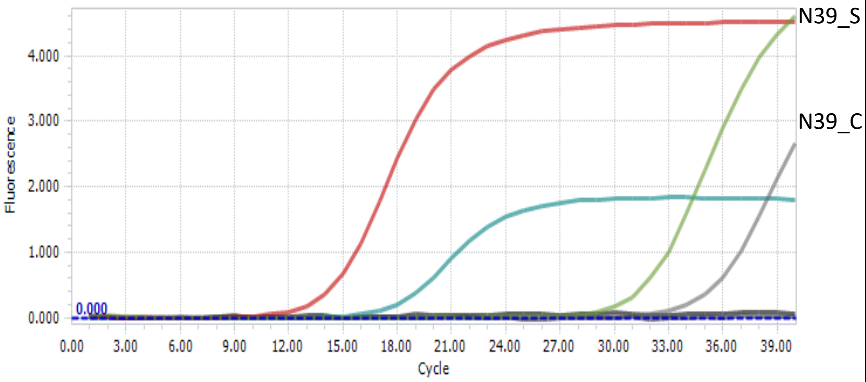

20	N40_D0	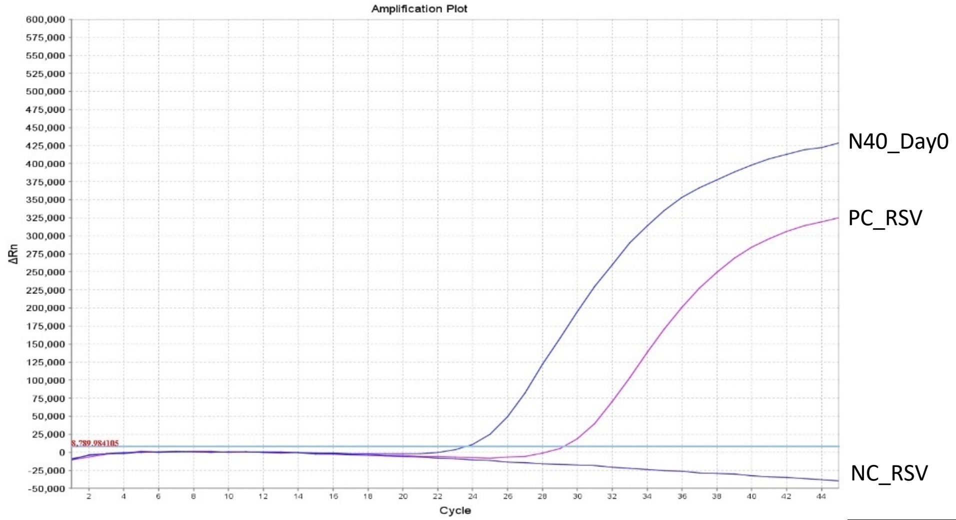	
	N40_D3	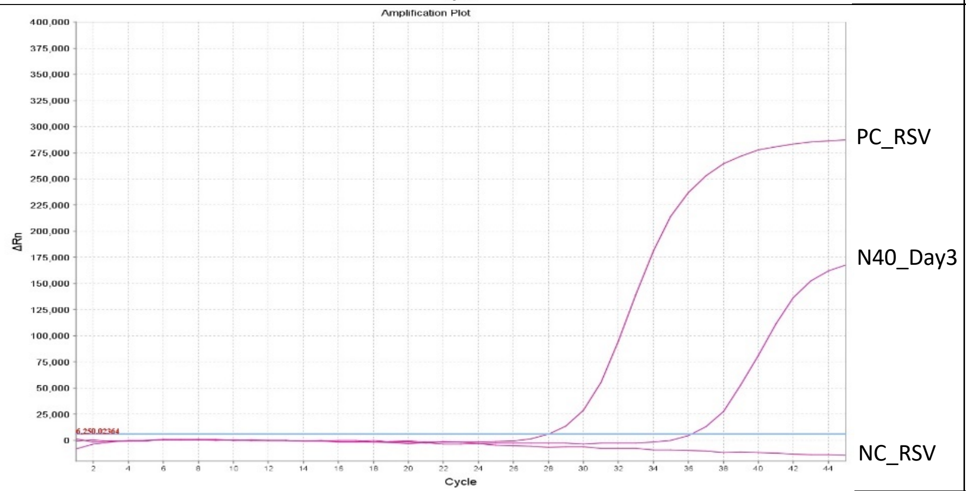	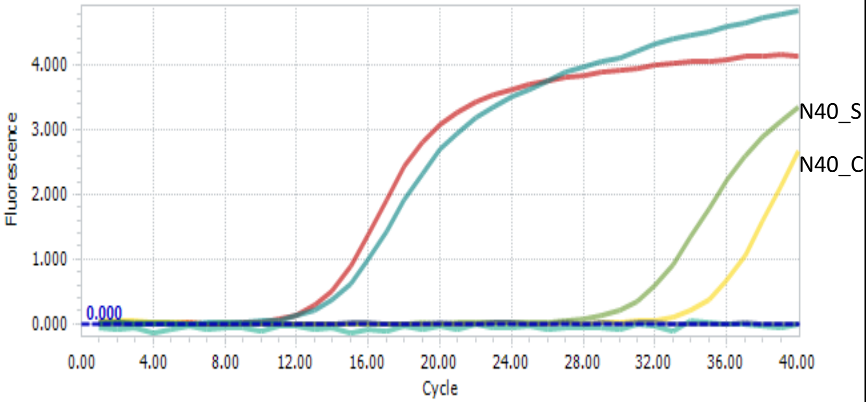

21	N41_D0	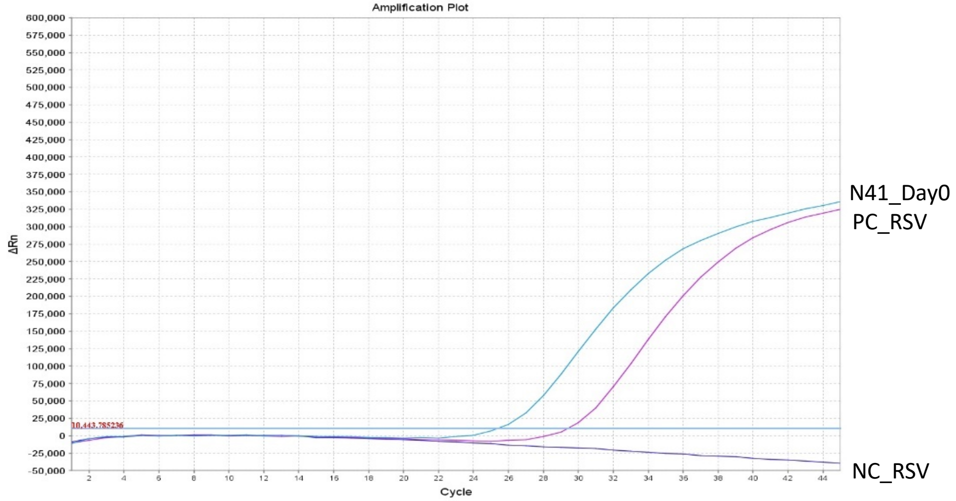	
	N41_D3	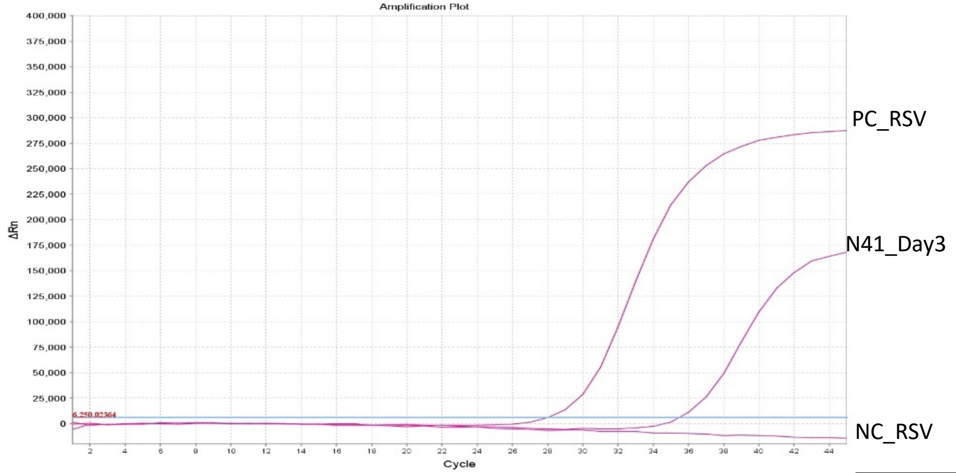	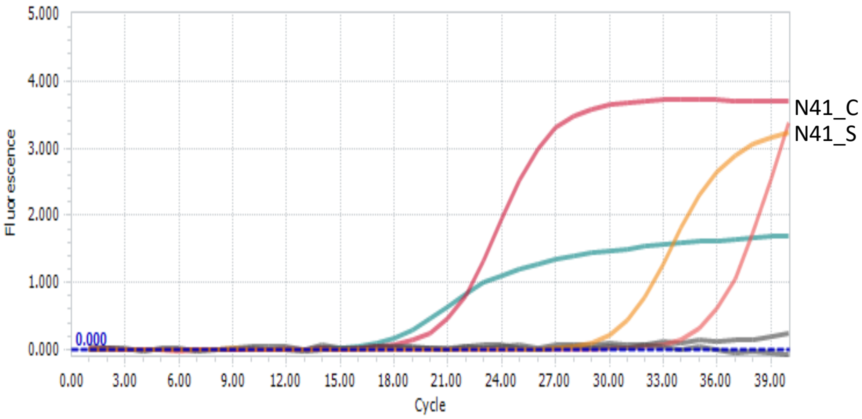

22	N43_D0	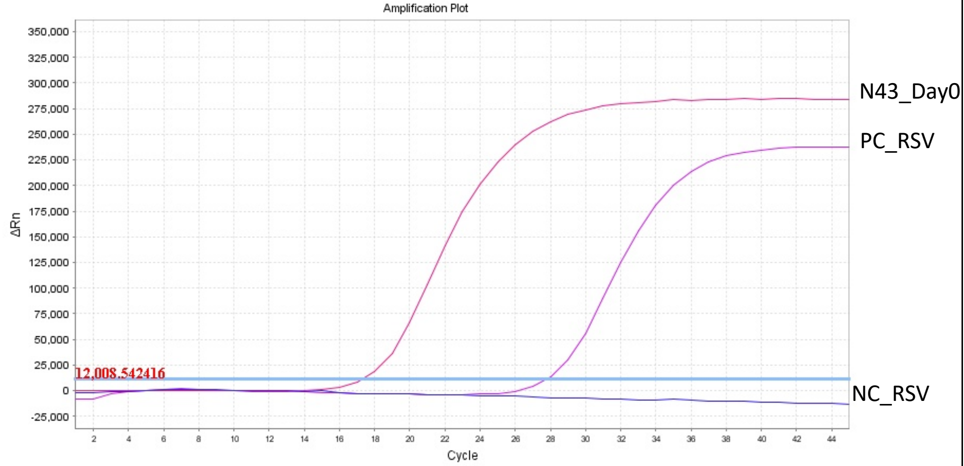	
	N43_D3	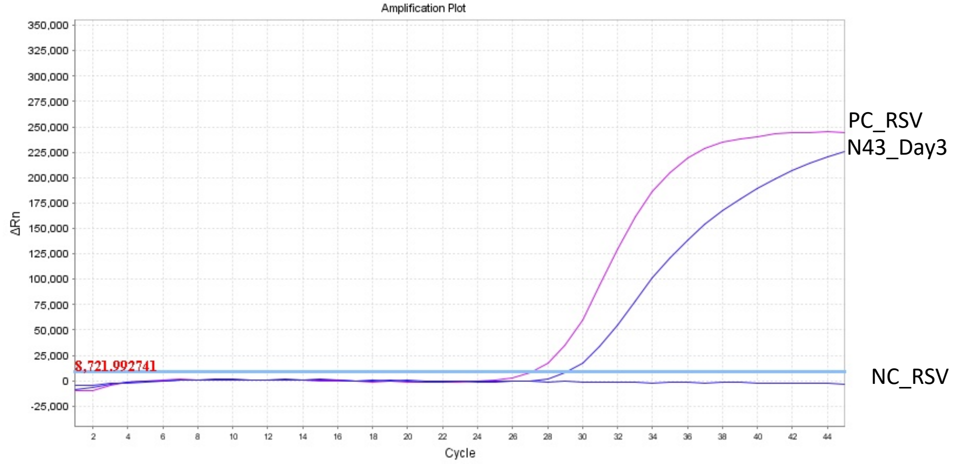	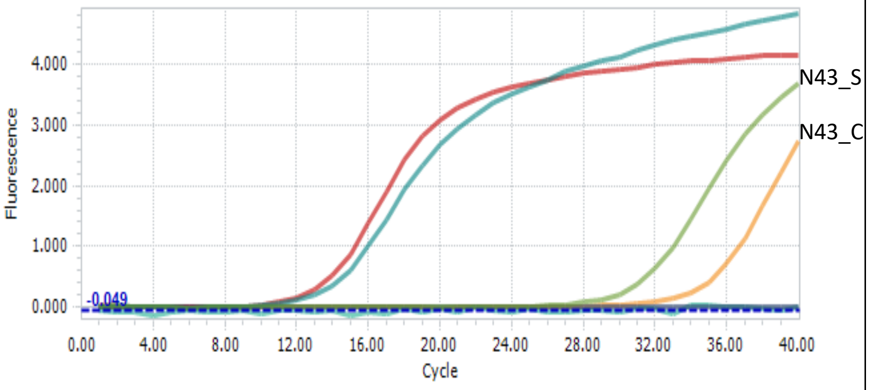

<table>
<tr><td>23</td><td>N45
_D0</td><td>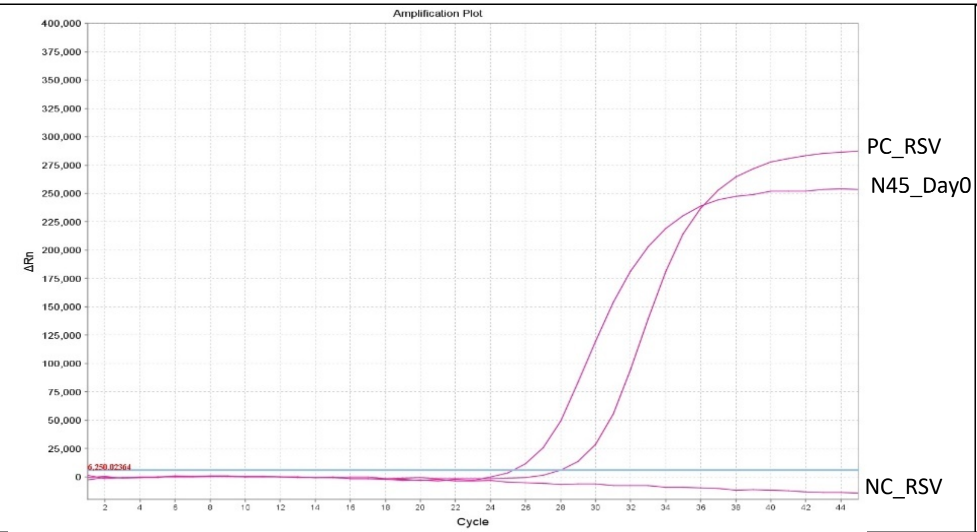
</td><td></td></tr>
<tr><td></td><td>N45_D3</td><td>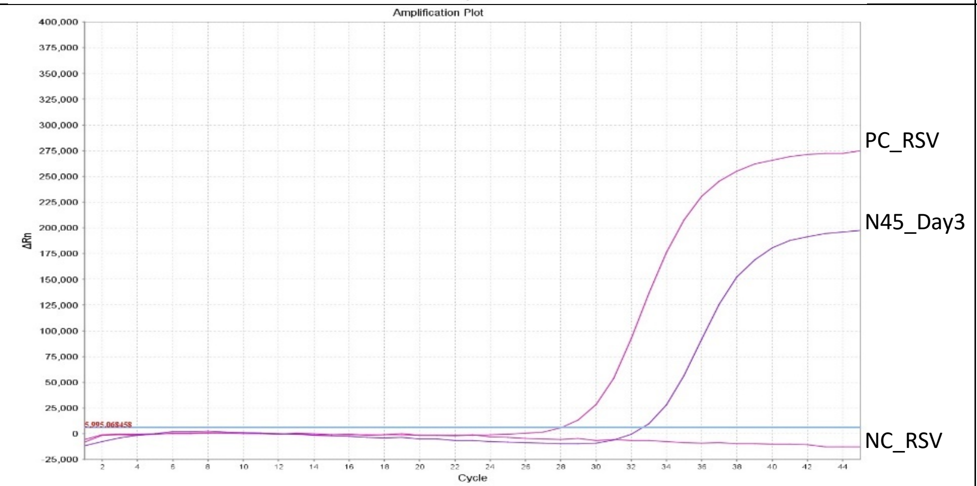
</td><td>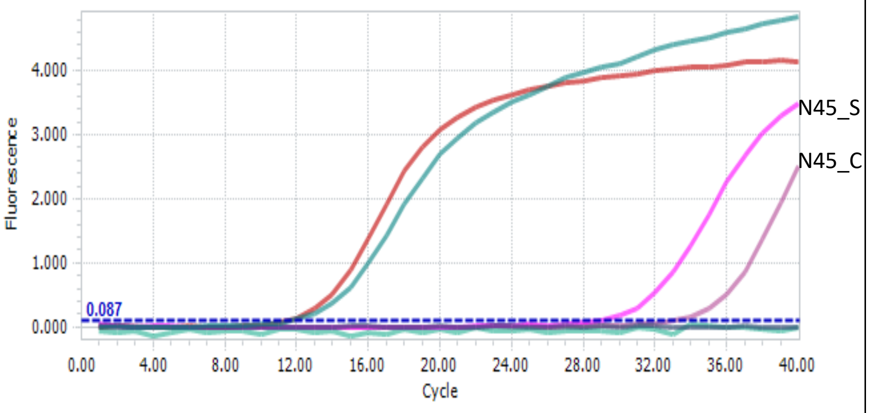
</td></tr>
</table>

<table>
<tr><td>24</td><td>N47
_D0</td><td>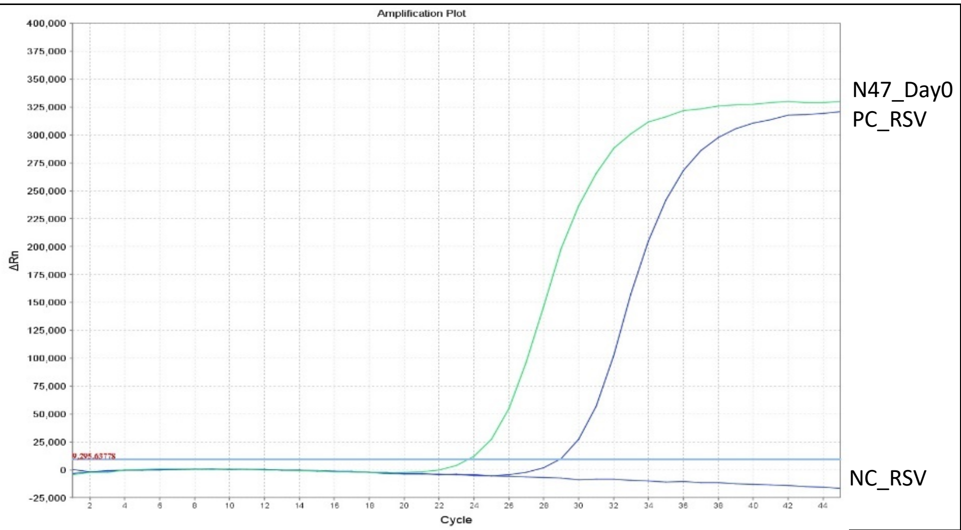</td><td></td></tr>
<tr><td></td><td>N47_D3</td><td>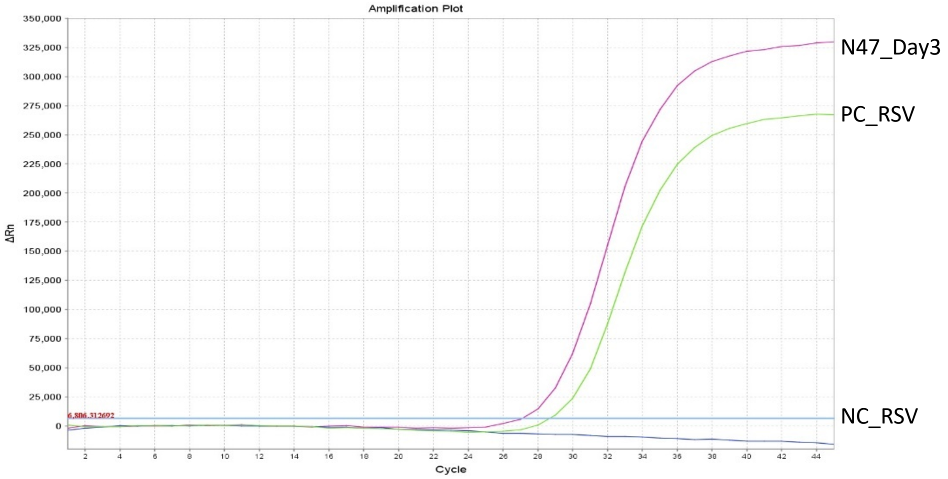</td><td>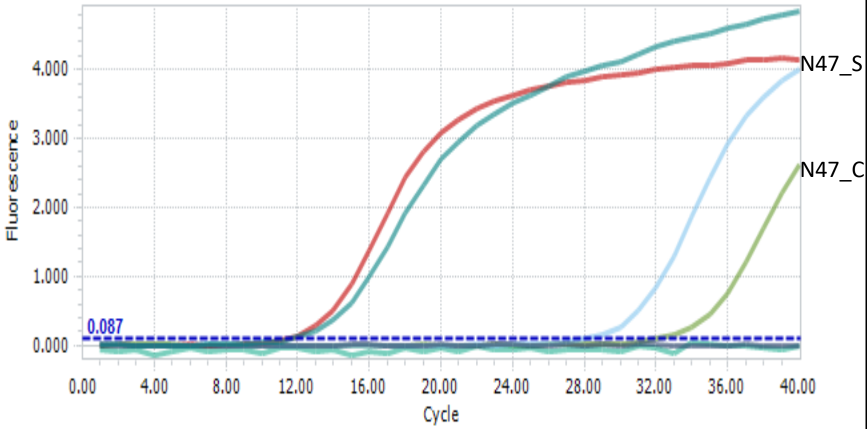</td></tr>
</table>

<table>
<tr><td rowspan="2">25</td><td>N48 _D0</td><td>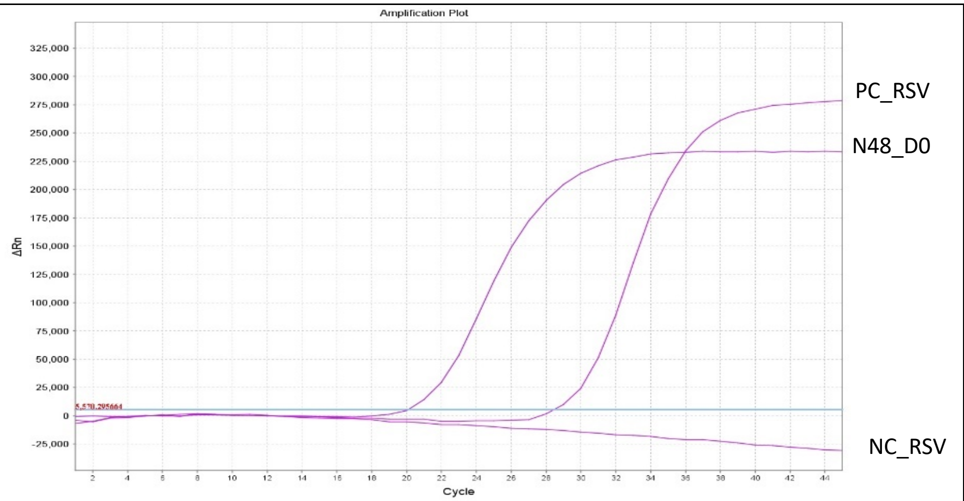
</td><td></td></tr>
<tr><td>N48_D3</td><td>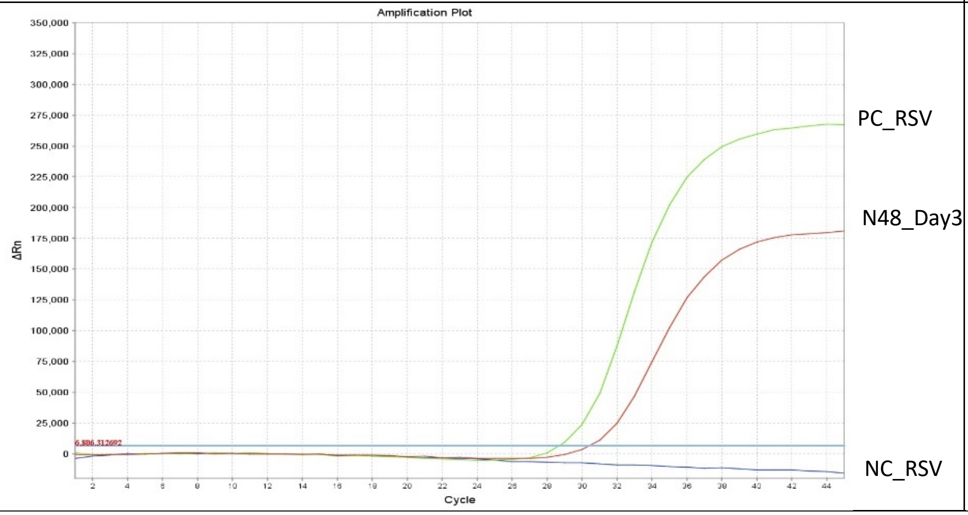
</td><td>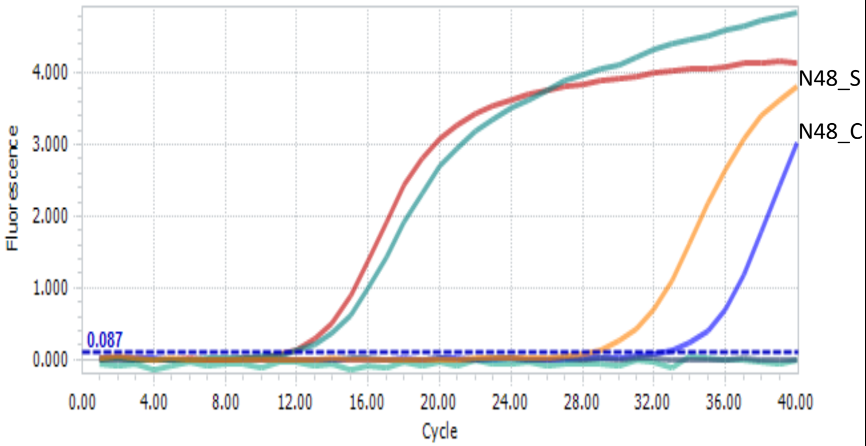
</td></tr>
</table>

<table>
<tr><td rowspan="2">26</td><td>N51_D0</td><td>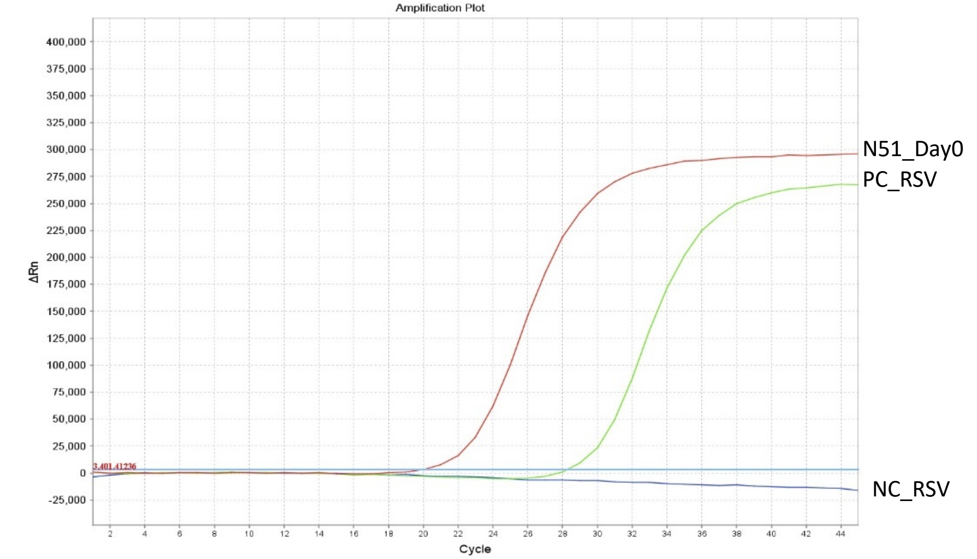</td><td></td></tr>
<tr><td>N51_D3</td><td>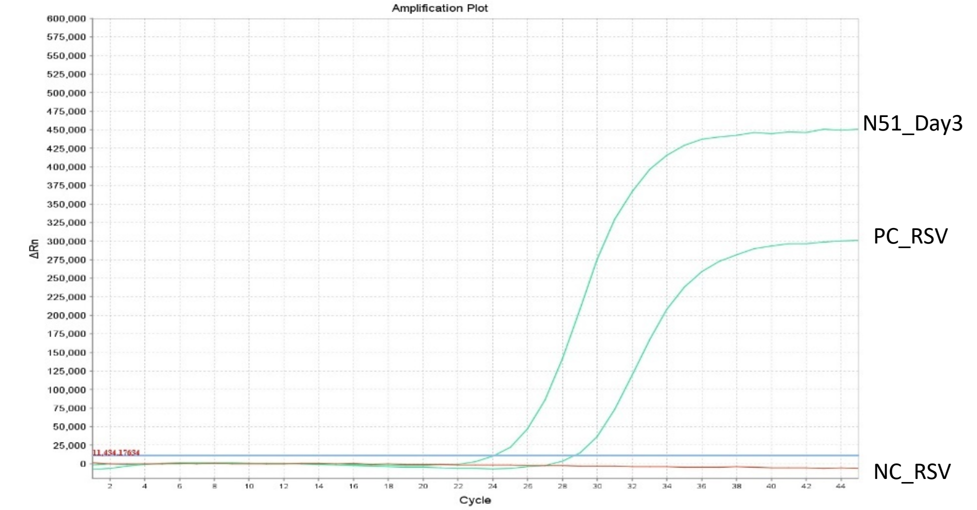</td><td>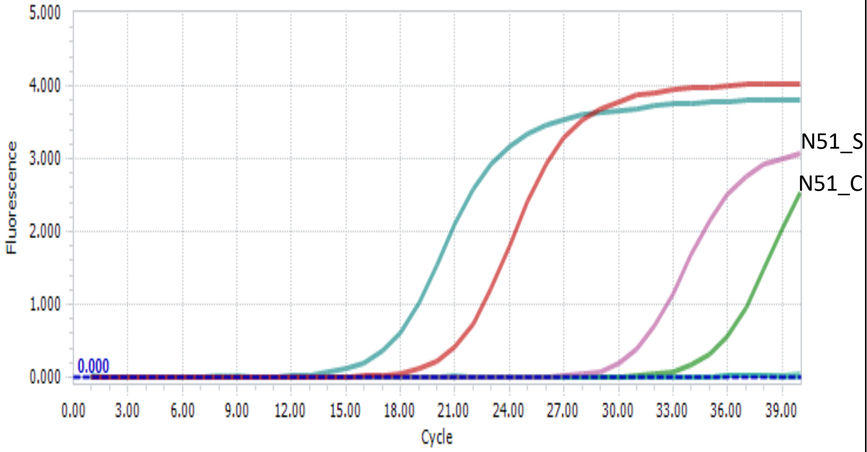</td></tr>
</table>

<table>
<tr><td rowspan="2">27</td><td>N52_D0</td><td>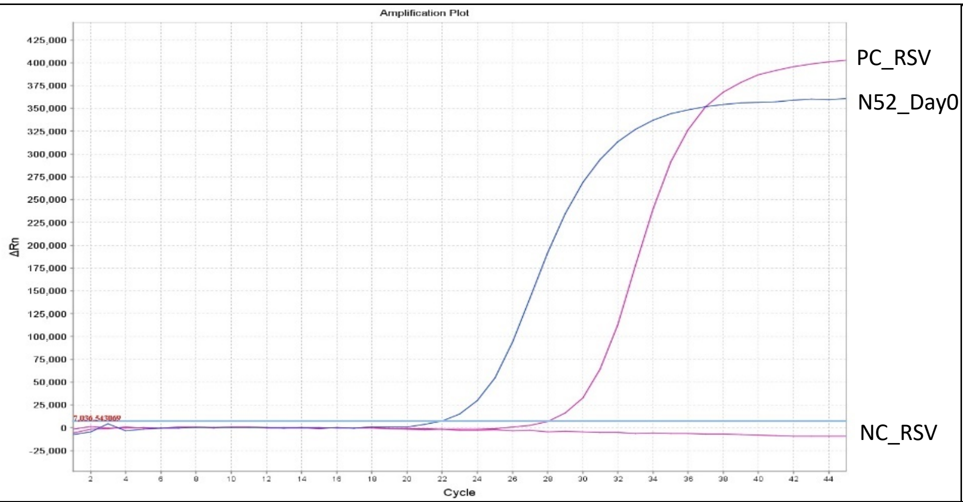
</td><td></td></tr>
<tr><td>N52_D3</td><td>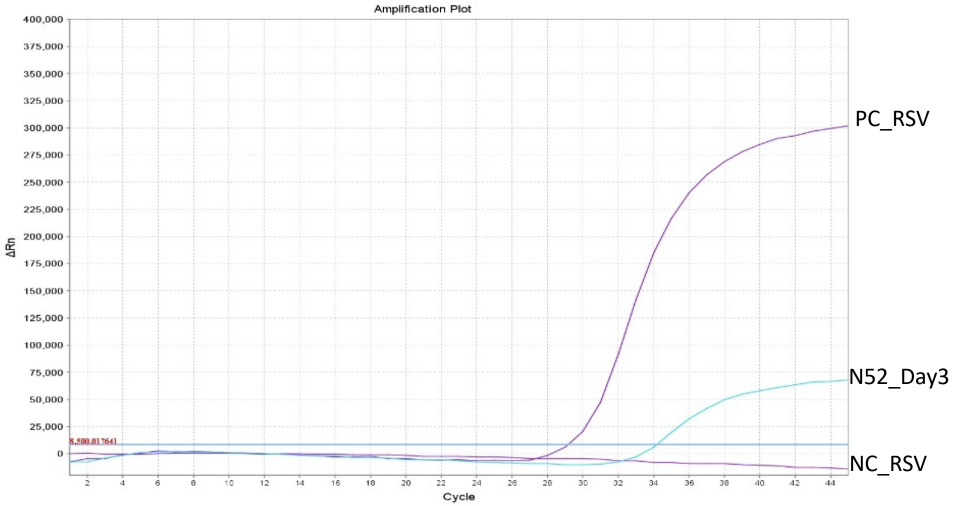
</td><td>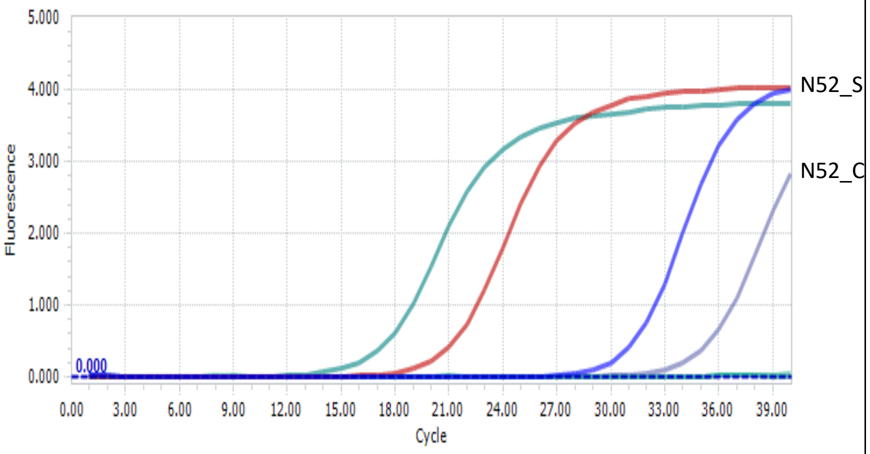
</td></tr>
</table>

28	N54_D0	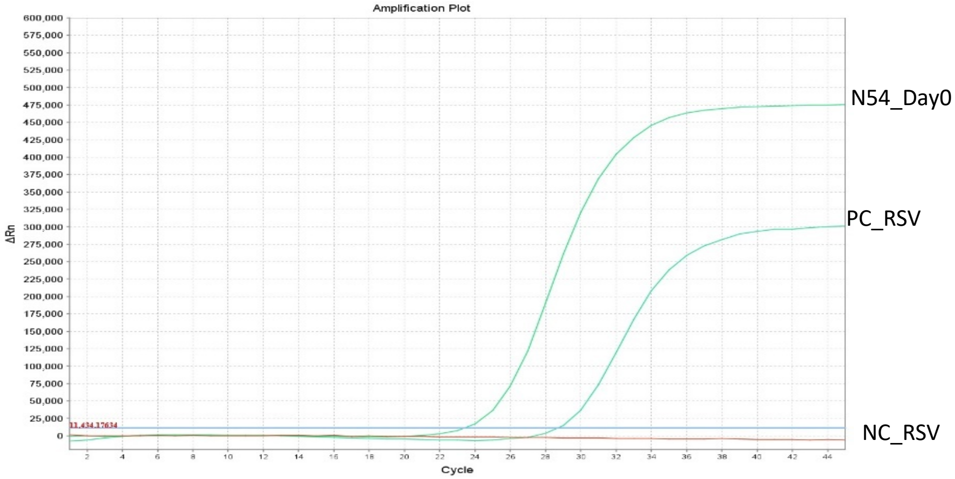	
	N54_D3	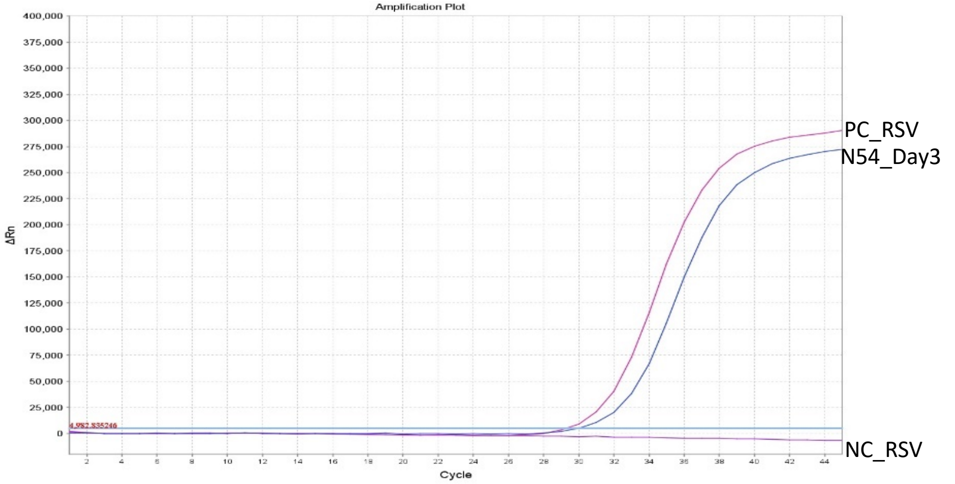	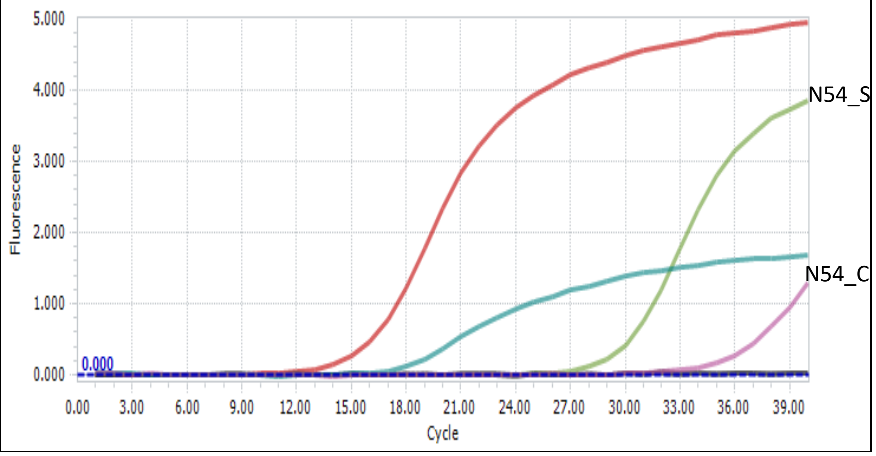

29	N56_D0	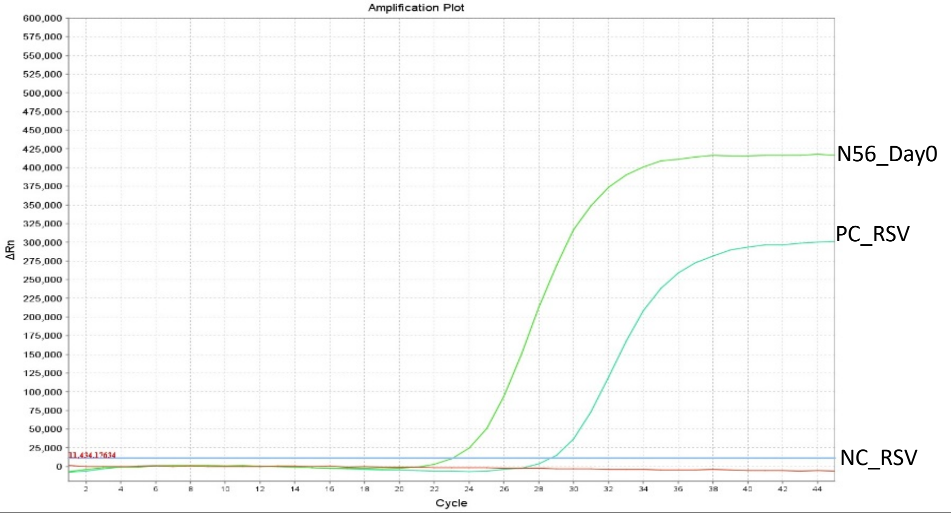	
	N56_D3	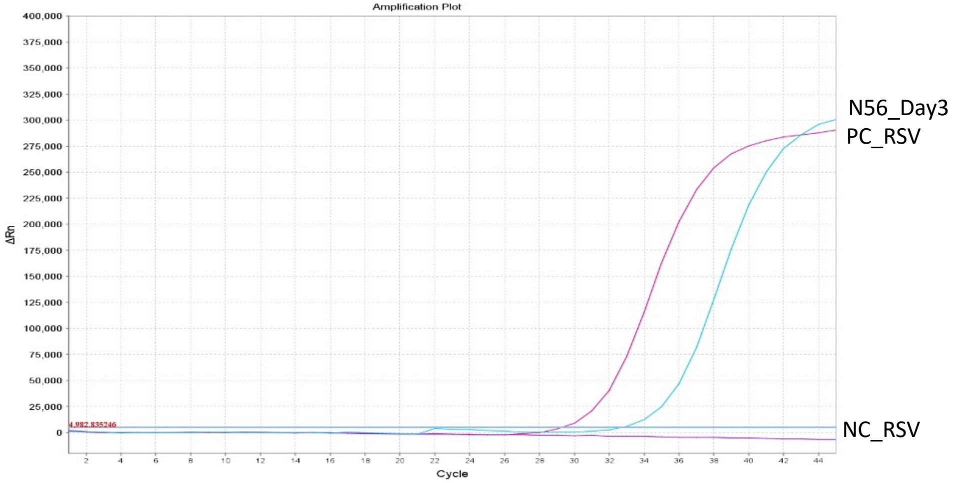	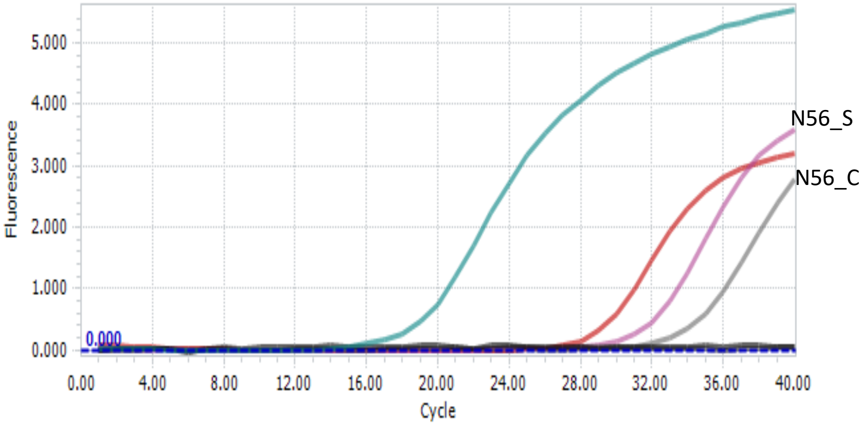

<table>
<tr><td>30</td><td>N57_D0</td><td>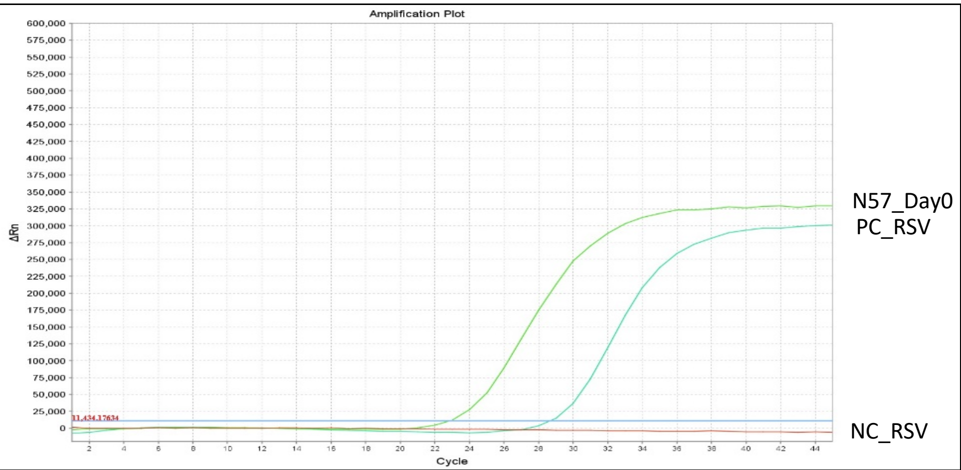
</td><td></td></tr>
<tr><td></td><td>N57_D3</td><td>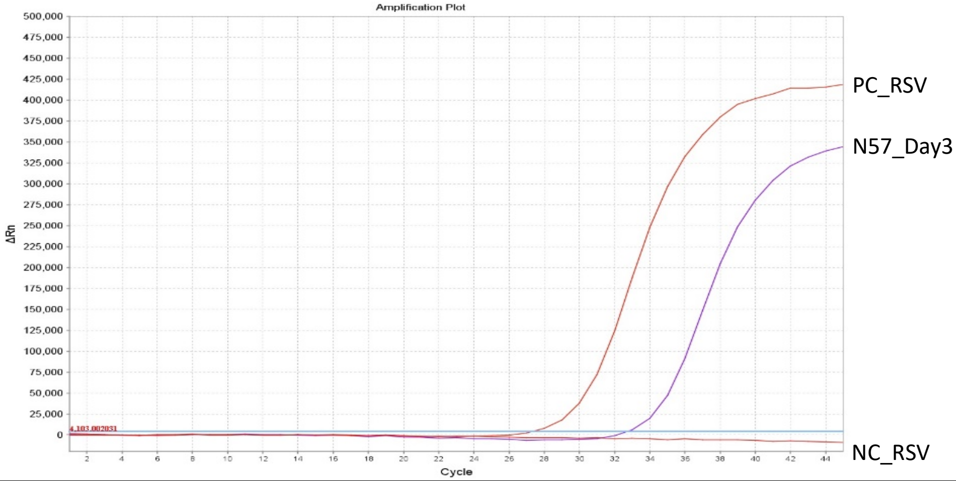
</td><td>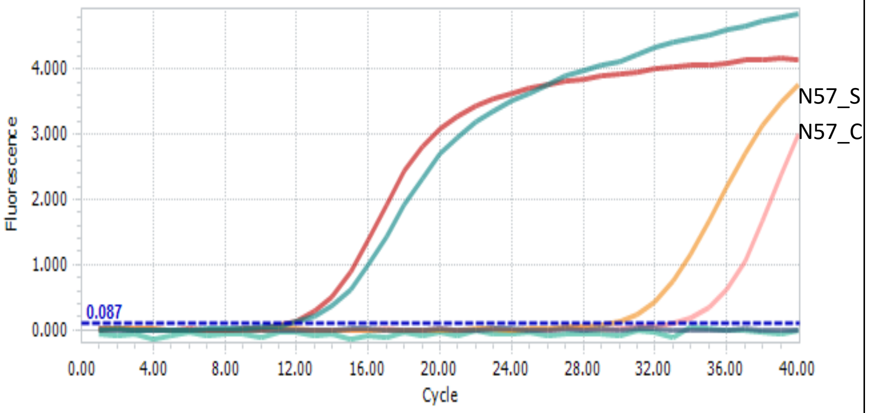
</td></tr>
</table>

<table>
<tr><td rowspan="2">31</td><td>N58_D0</td><td>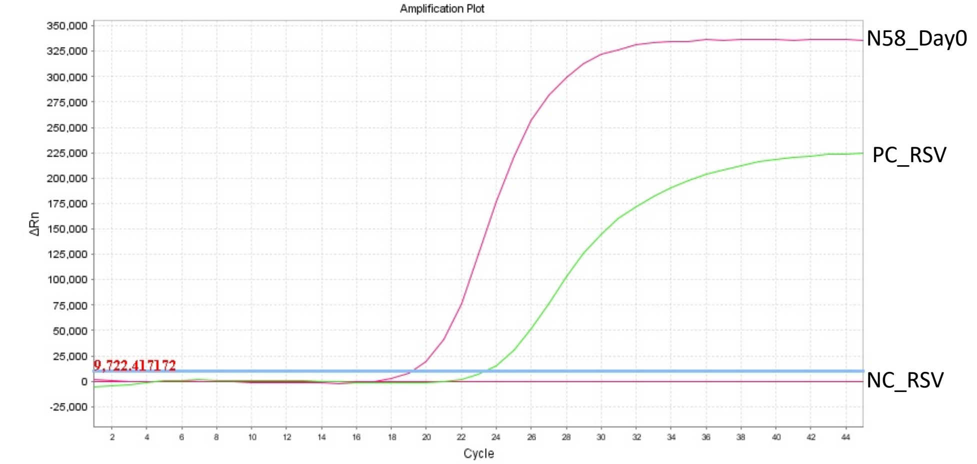
</td><td></td></tr>
<tr><td>N58_D3</td><td>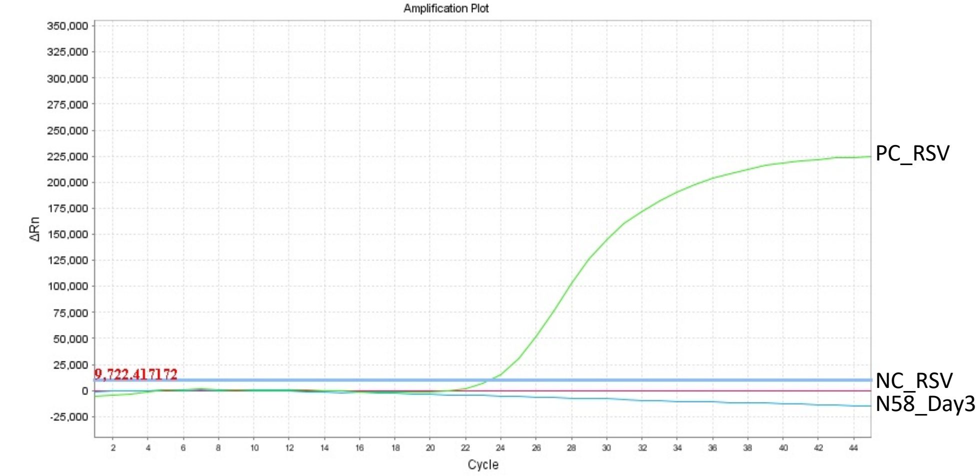
</td><td>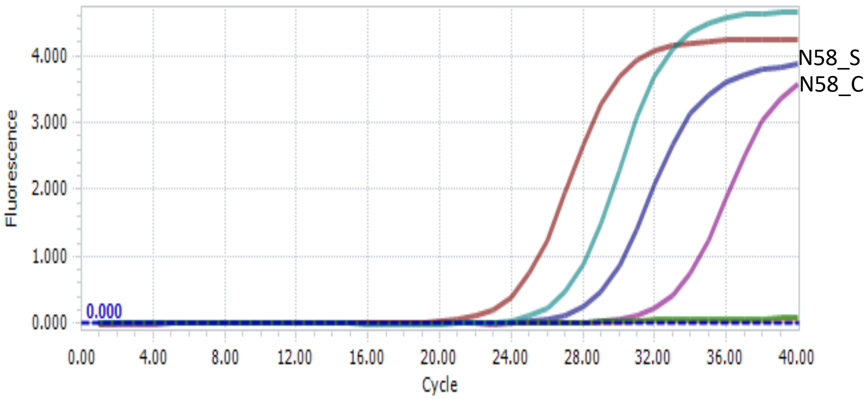
</td></tr>
</table>

32	N61_D0	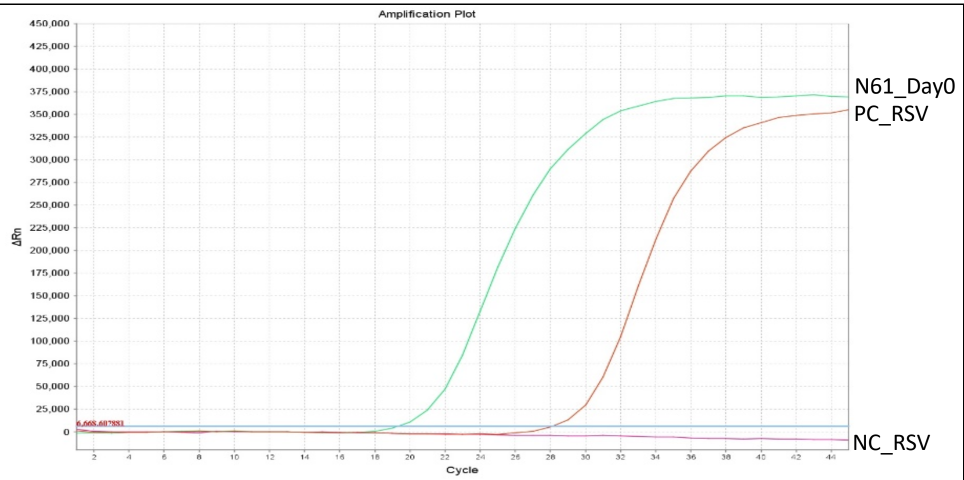	
	N61_D3	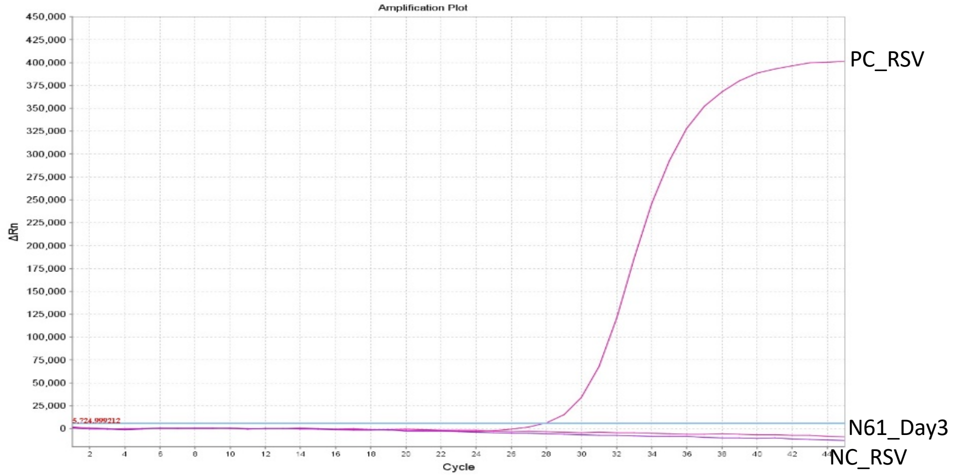	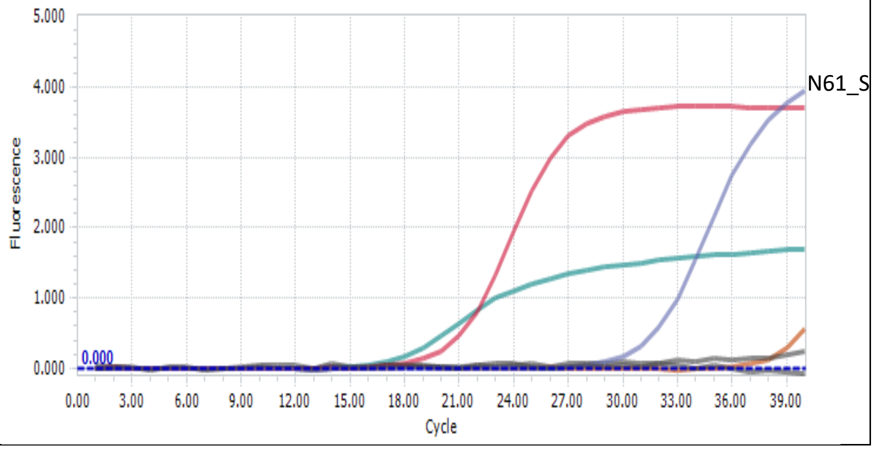

<table>
<tr><td rowspan="2">33</td><td>N62_D0</td><td>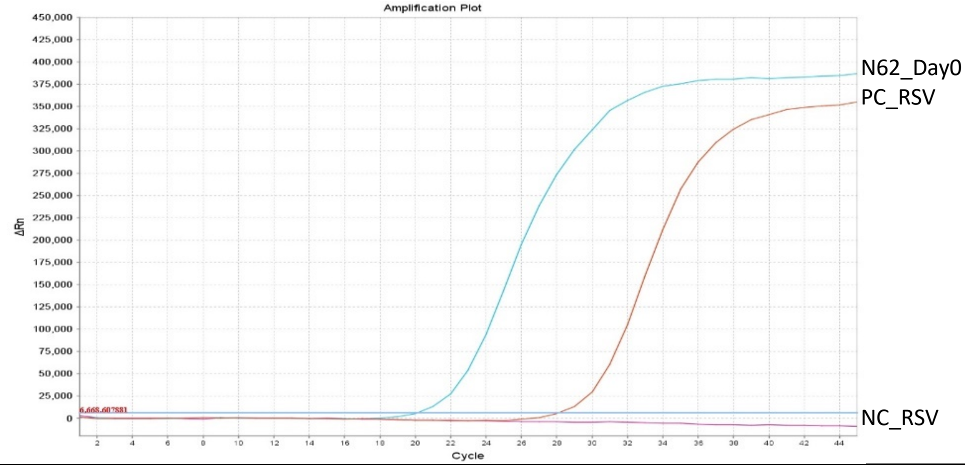</td><td></td></tr>
<tr><td>N62_D3</td><td>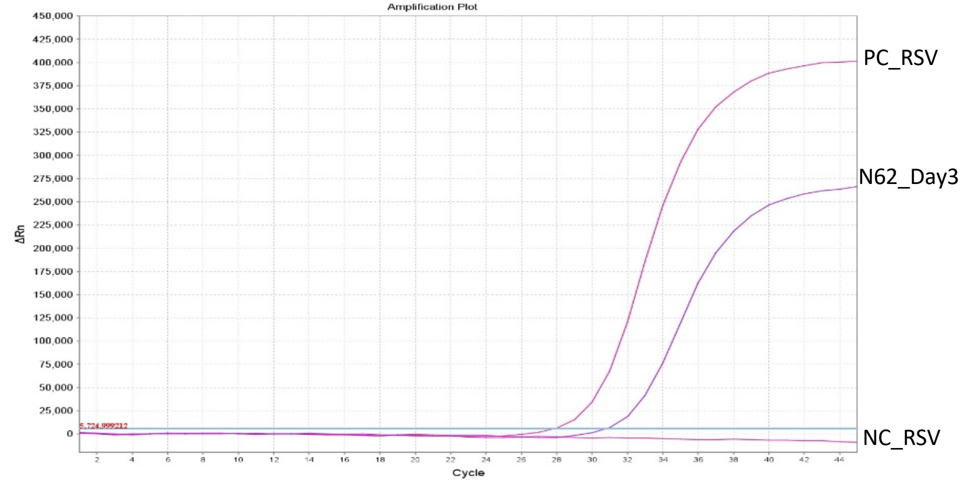</td><td>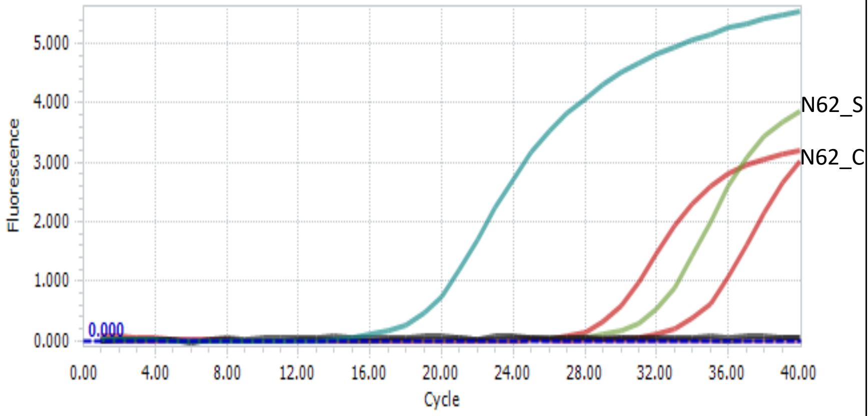</td></tr>
</table>

34	N63_D0	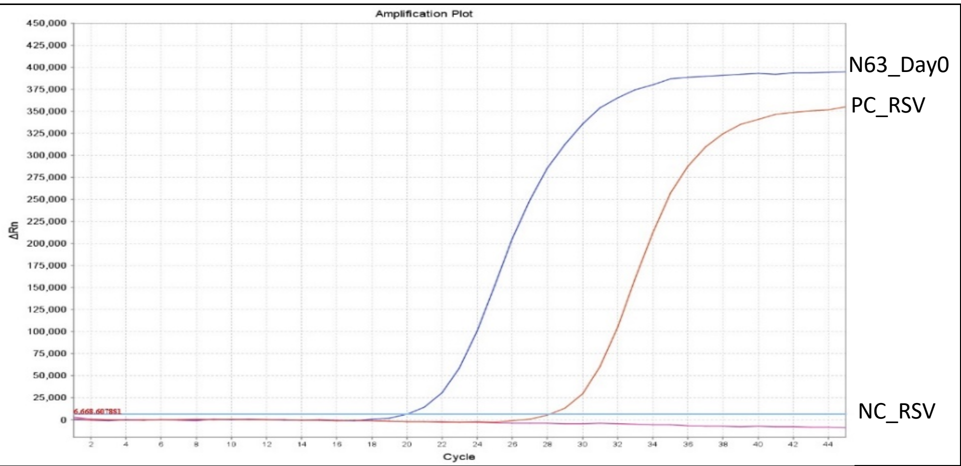	
	N63_D3	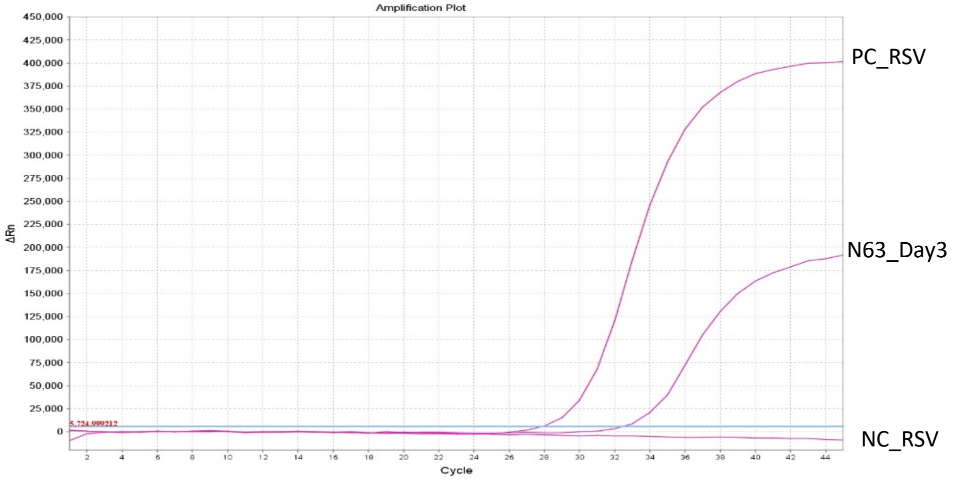	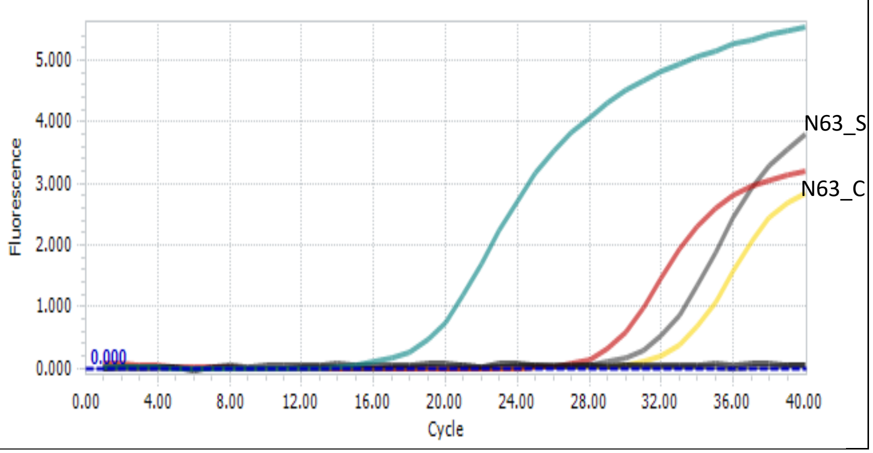

<table>
<tr><td>35</td><td>N64_D0</td><td>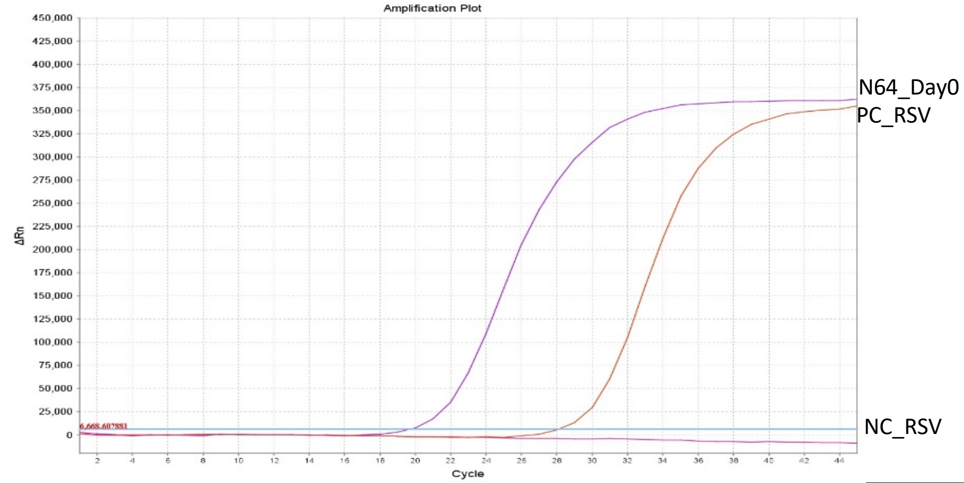</td><td></td></tr>
<tr><td></td><td>N64_D3</td><td>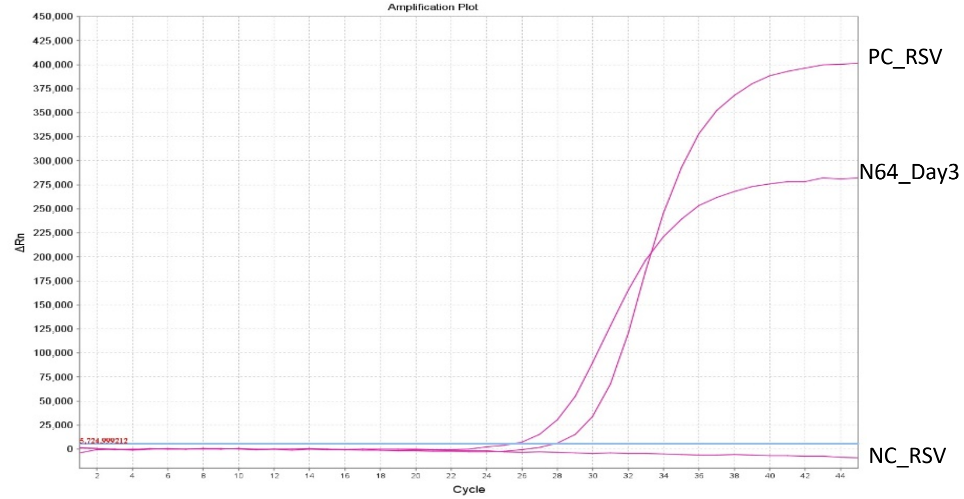</td><td>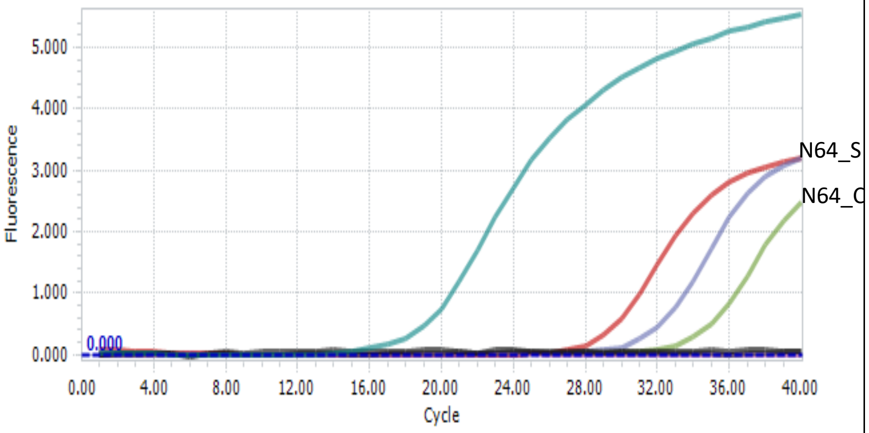</td></tr>
</table>

36	N67_D0	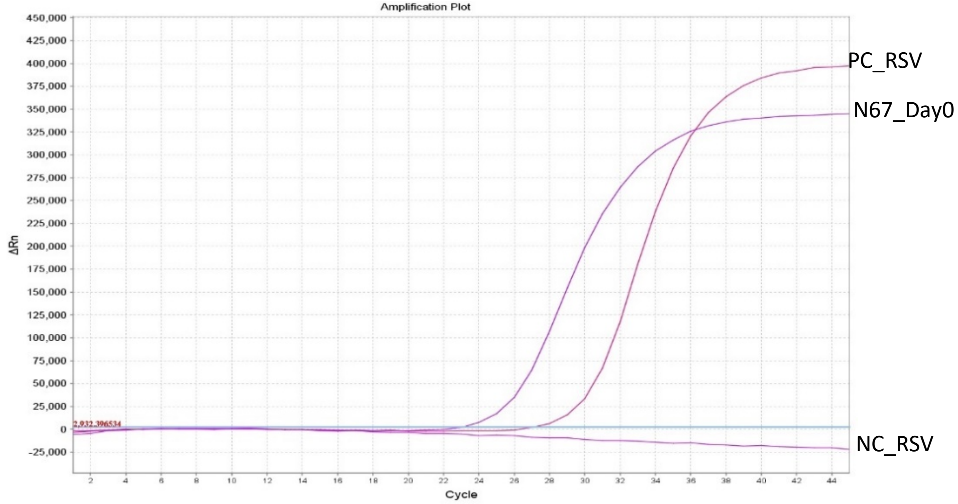	
	N67_D3	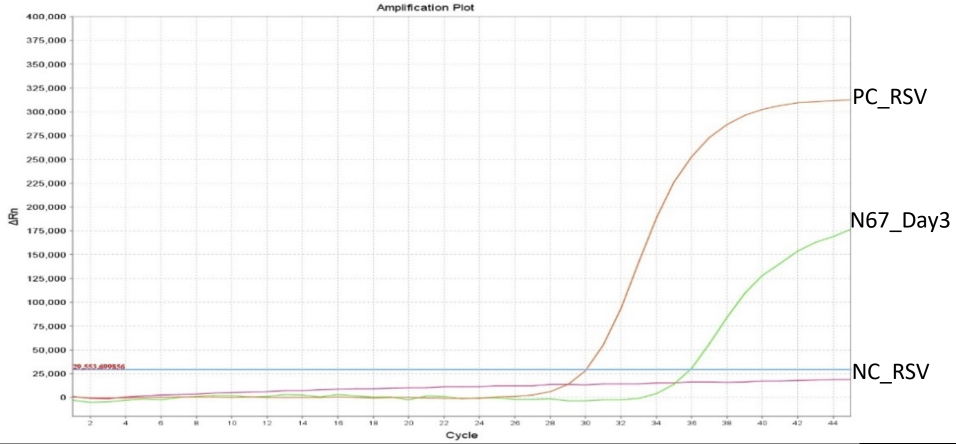	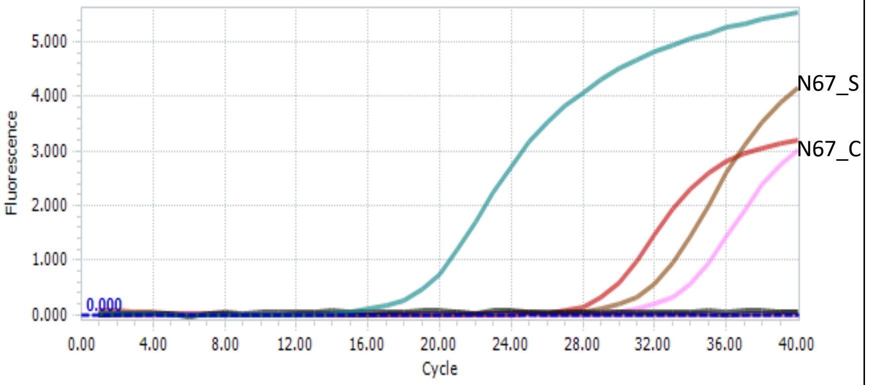

<table>
<tr><td rowspan="2">37</td><td>N68_D0</td><td>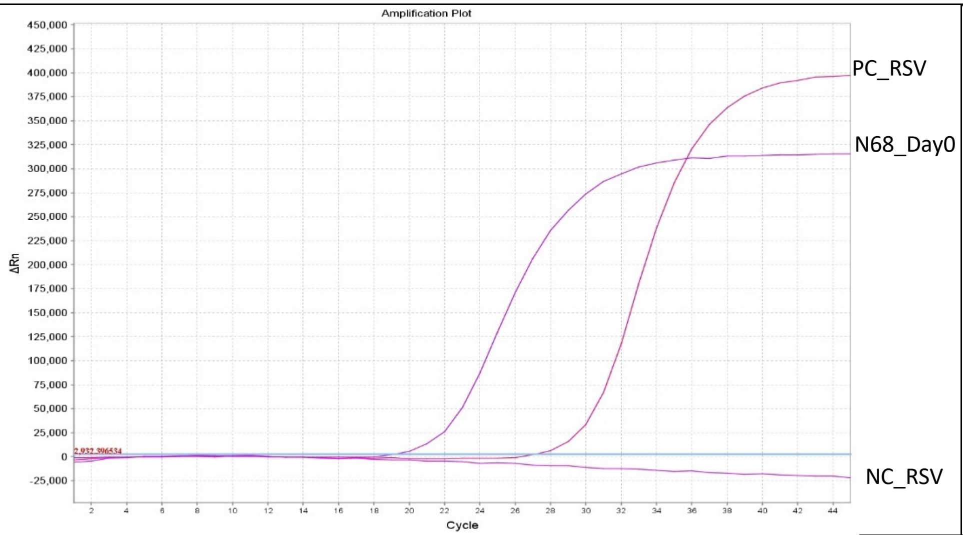
</td><td></td></tr>
<tr><td>N68_D3</td><td>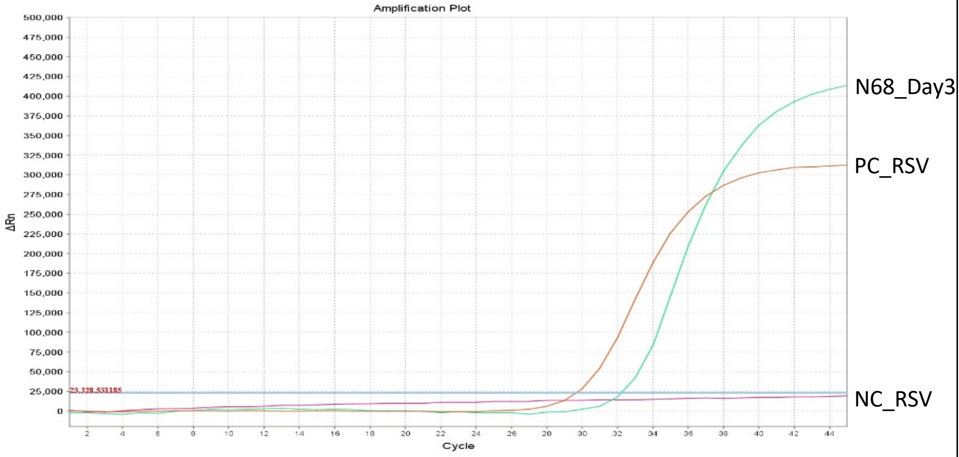
</td><td>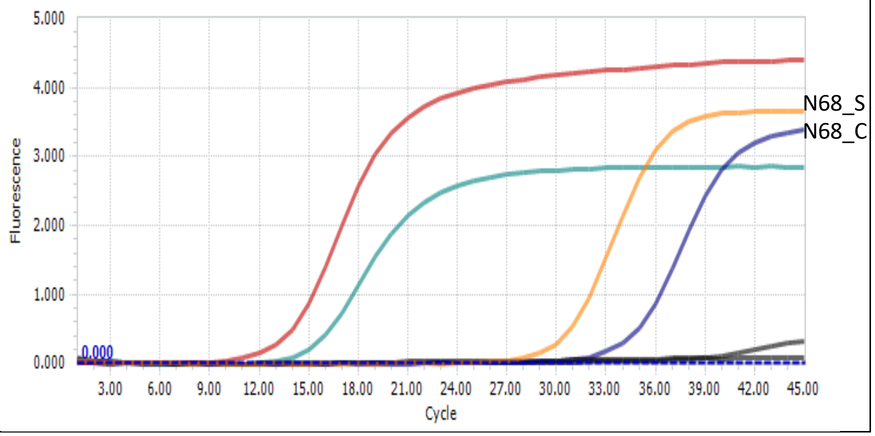
</td></tr>
</table>

<table>
<tr><td rowspan="2">38</td><td>N69_D0</td><td>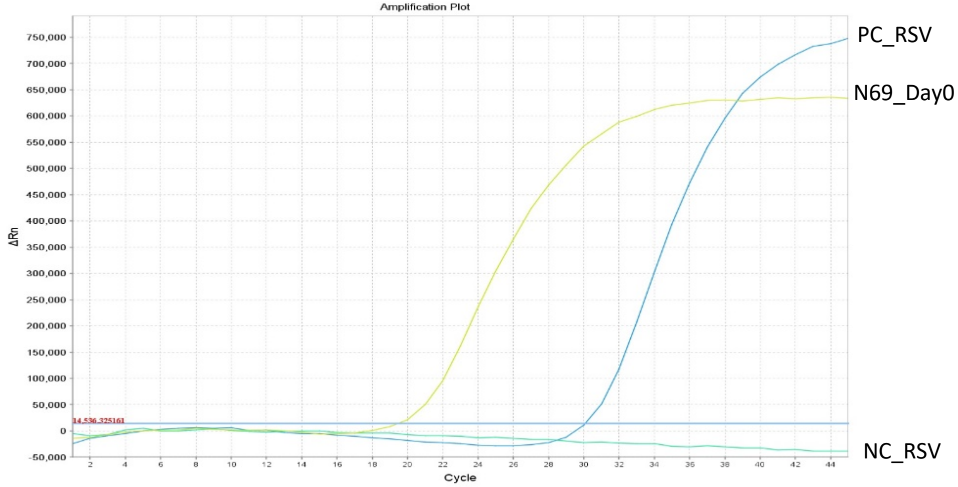
</td><td></td></tr>
<tr><td>N69_D3</td><td>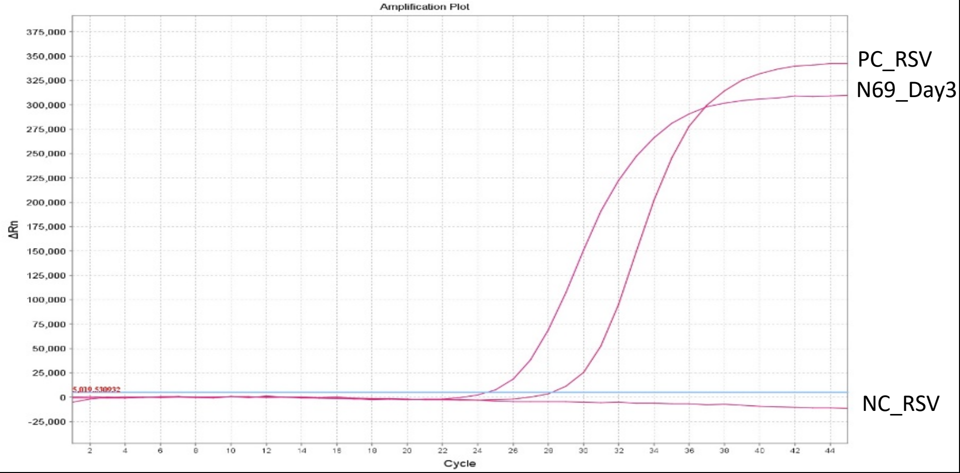
</td><td>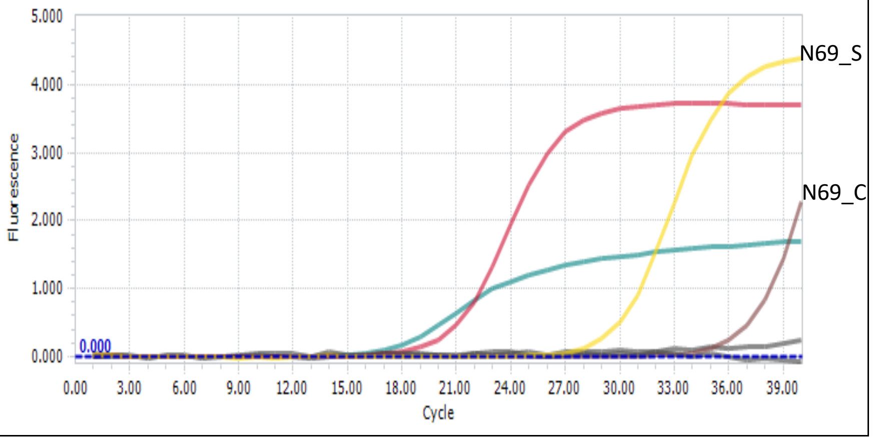
</td></tr>
</table>

<table>
<tr><td rowspan="2">39</td><td>N77_D0</td><td>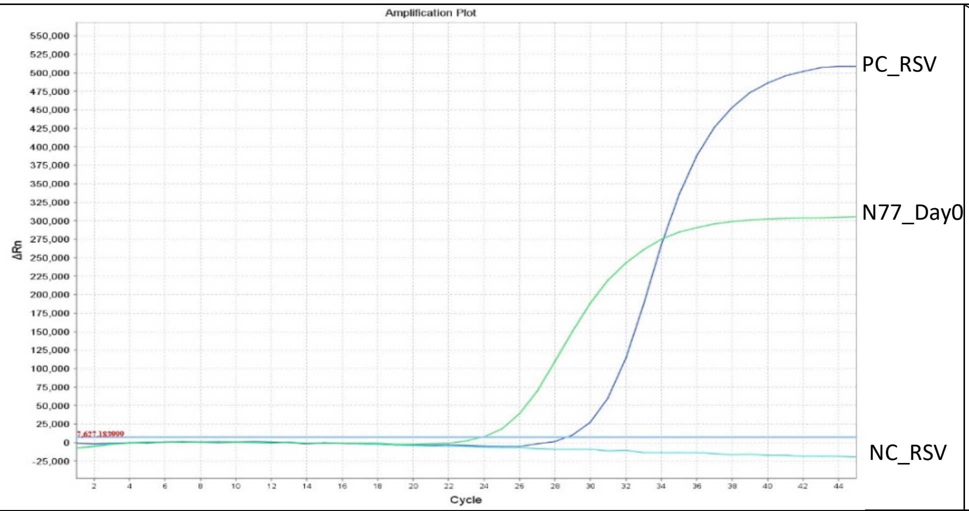
</td><td></td></tr>
<tr><td>N77_D3</td><td>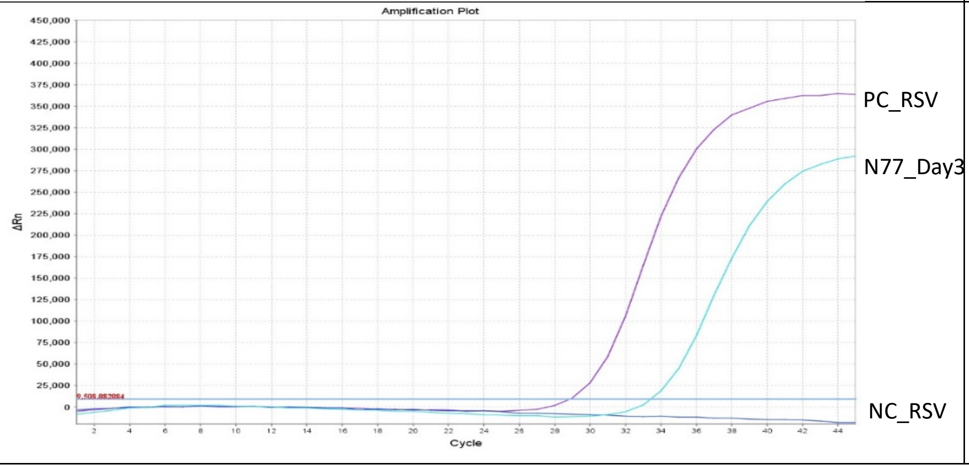
</td><td>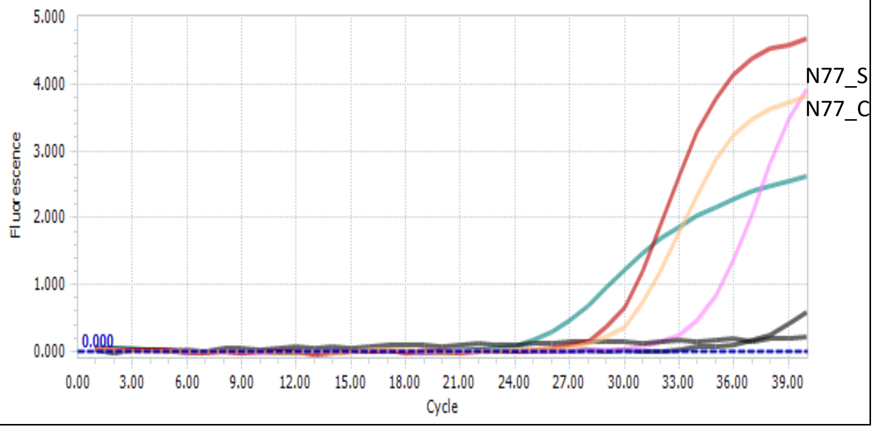
</td></tr>
</table>

<table>
<tr><td>40</td><td>N78_D0</td><td>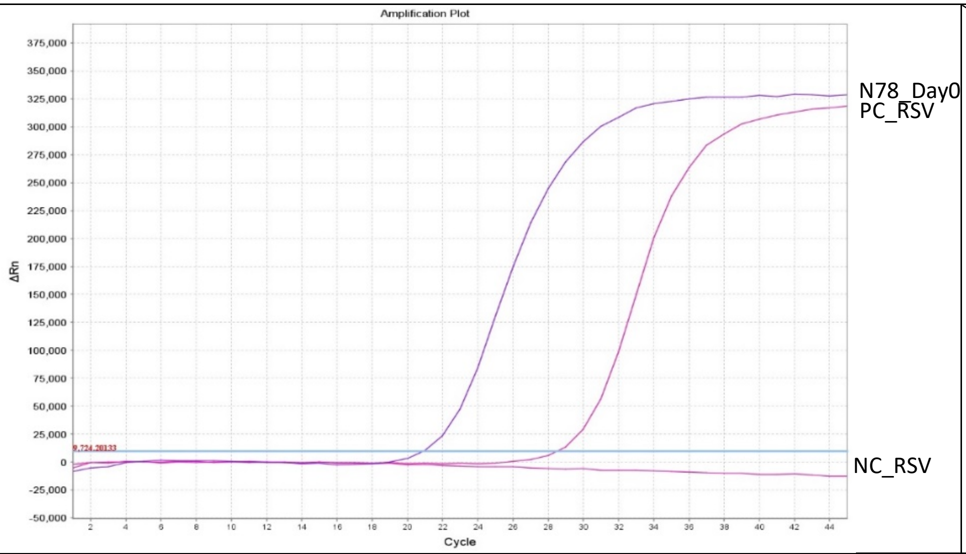
</td><td></td></tr>
<tr><td></td><td>N78_D3</td><td>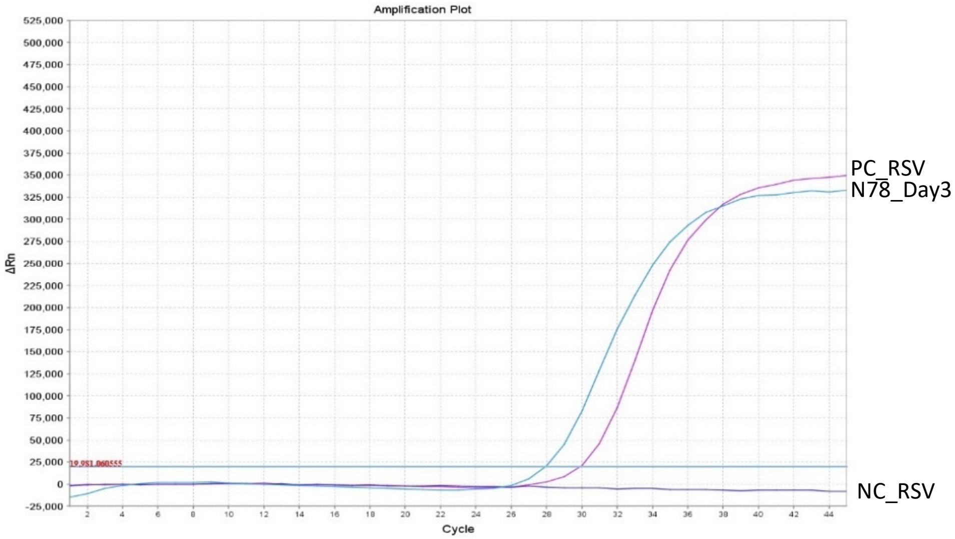
</td><td>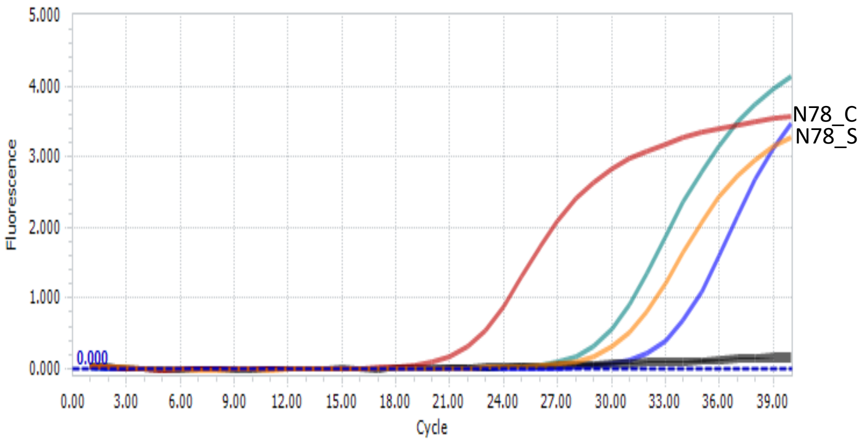
</td></tr>
</table>

41	N79_D0	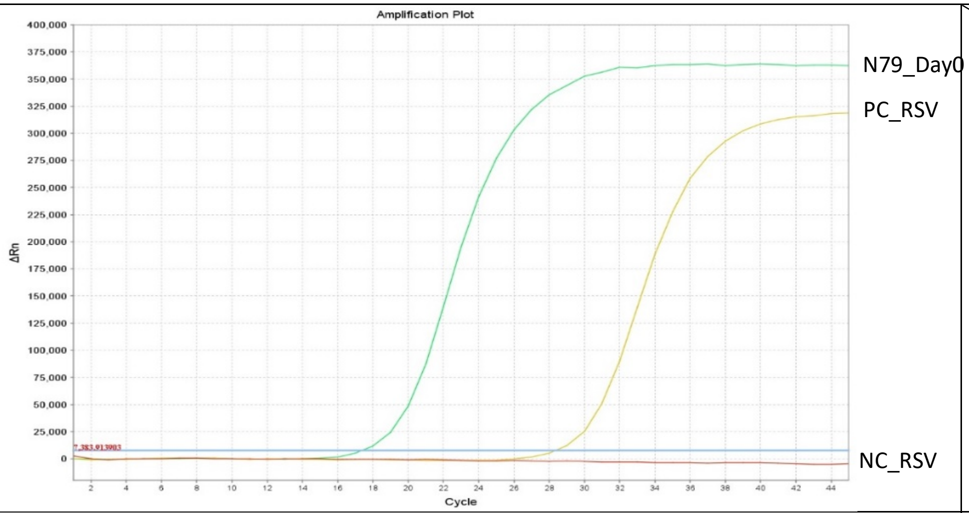	
	N79_D3	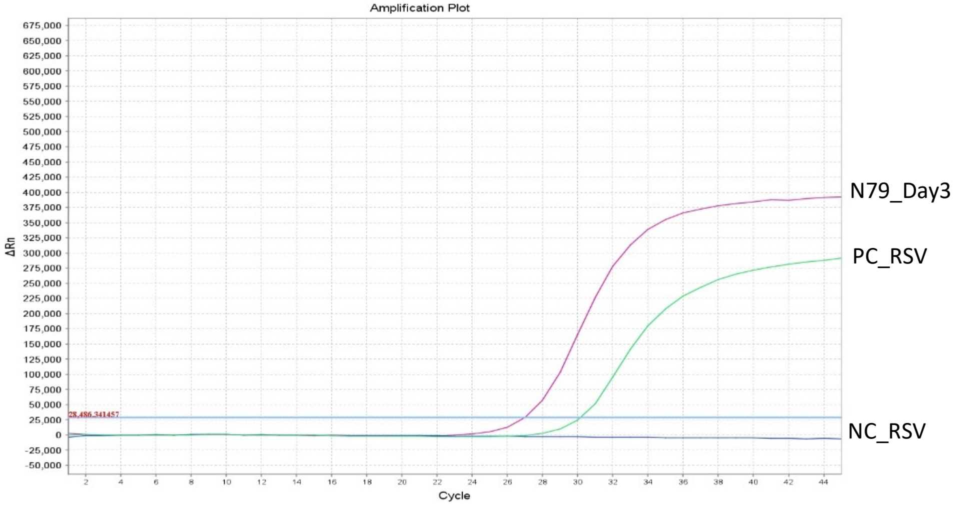	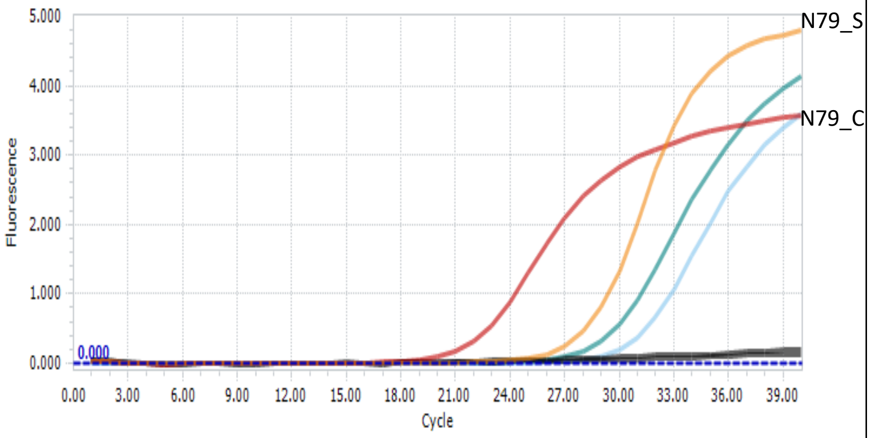

<table>
<tr><td rowspan="2">42</td><td>N80_D0</td><td>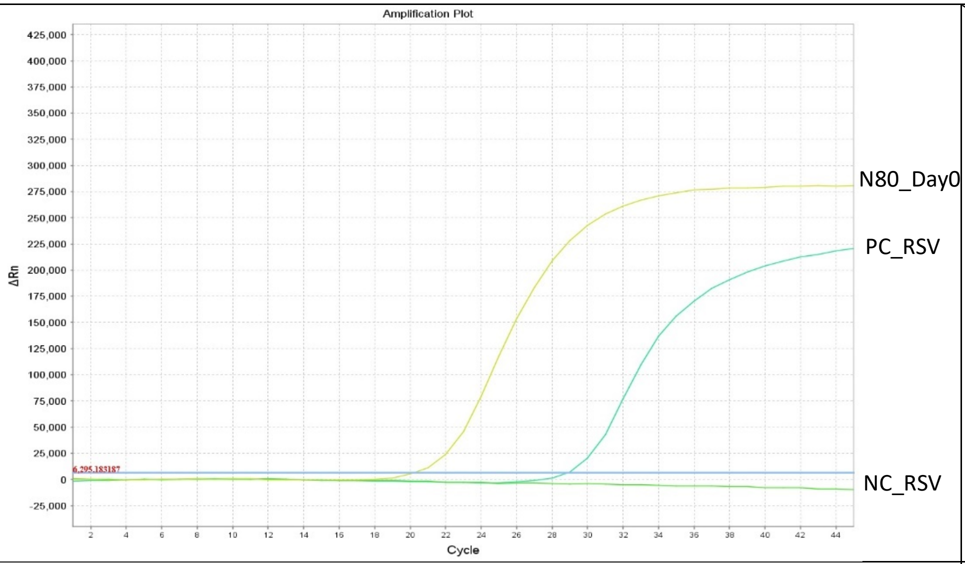
</td><td></td></tr>
<tr><td>N80_D3</td><td>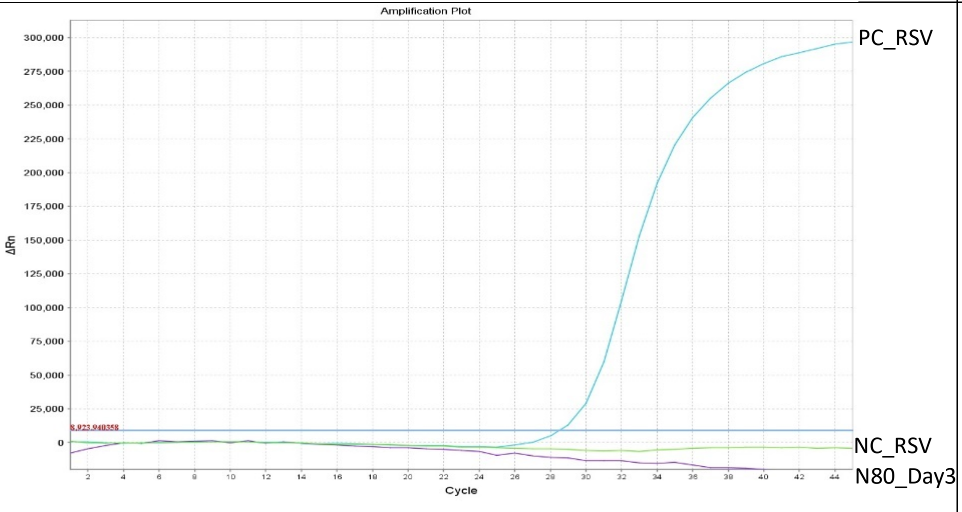
</td><td>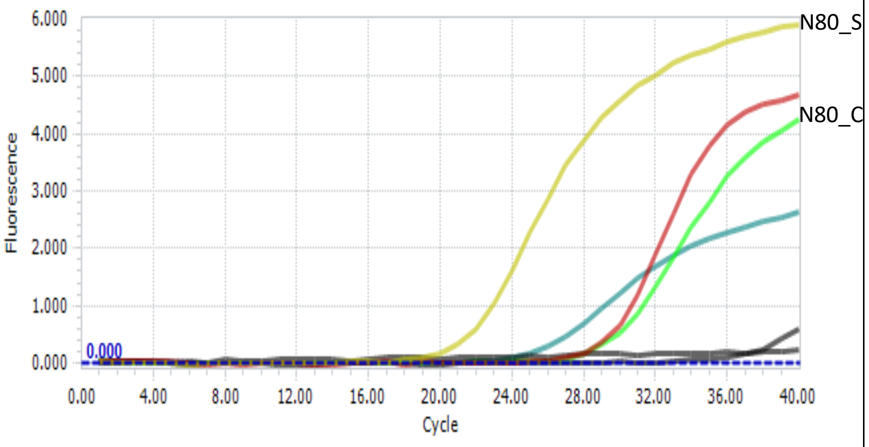
</td></tr>
</table>

<table>
<tr><td>43</td><td>N83_D0</td><td>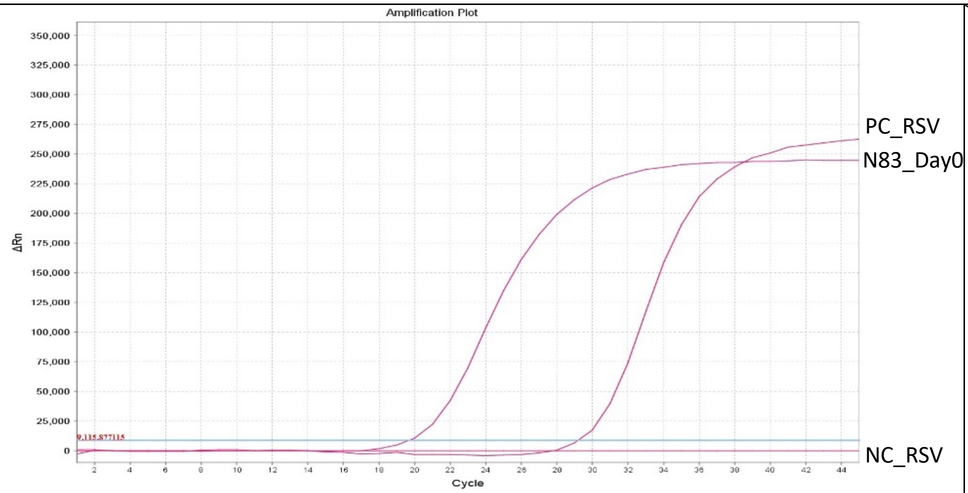
</td><td></td></tr>
<tr><td></td><td>N83_D3</td><td>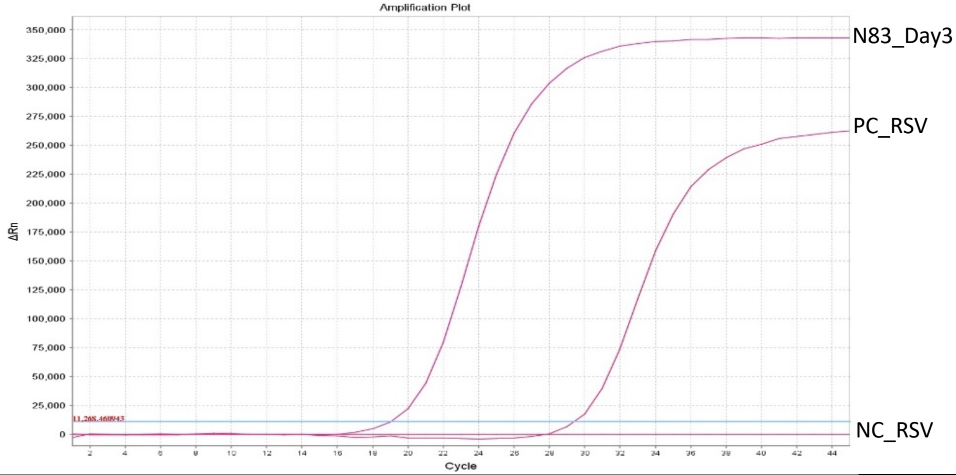
</td><td>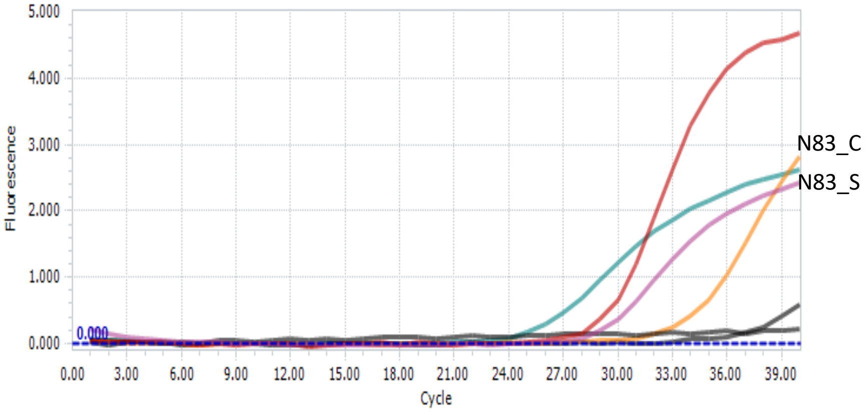
</td></tr>
</table>

44	N86_D0	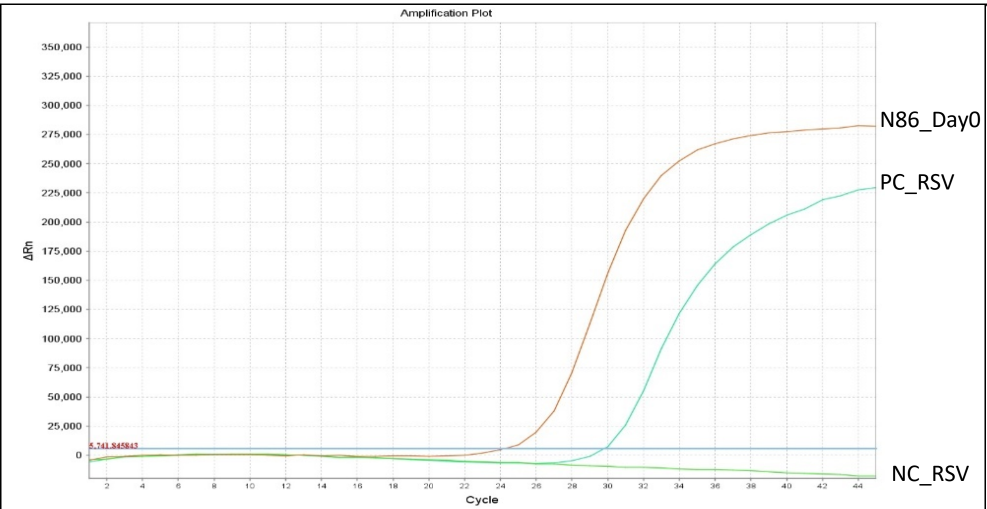	
	N86_D3	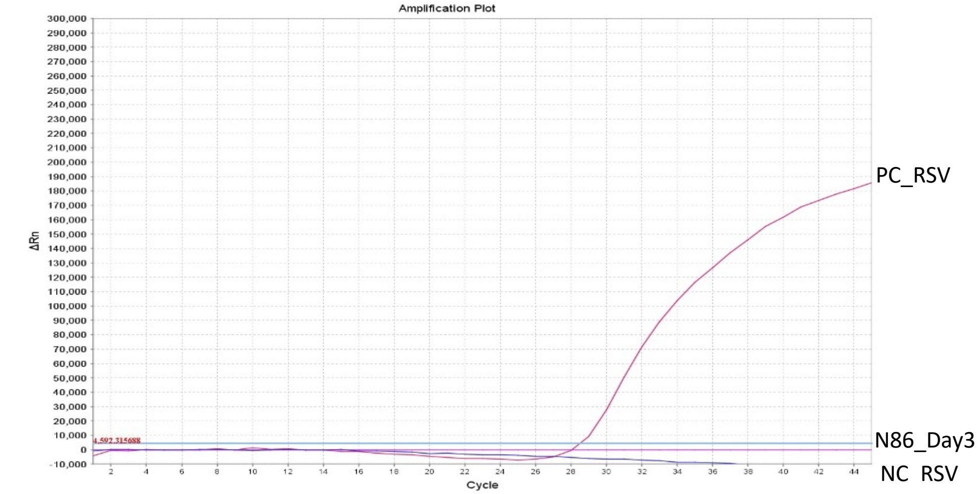	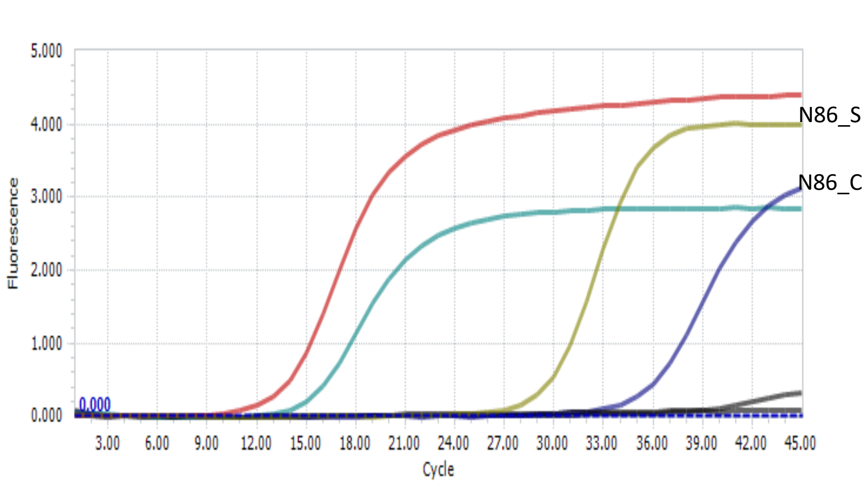

45	N92_D0	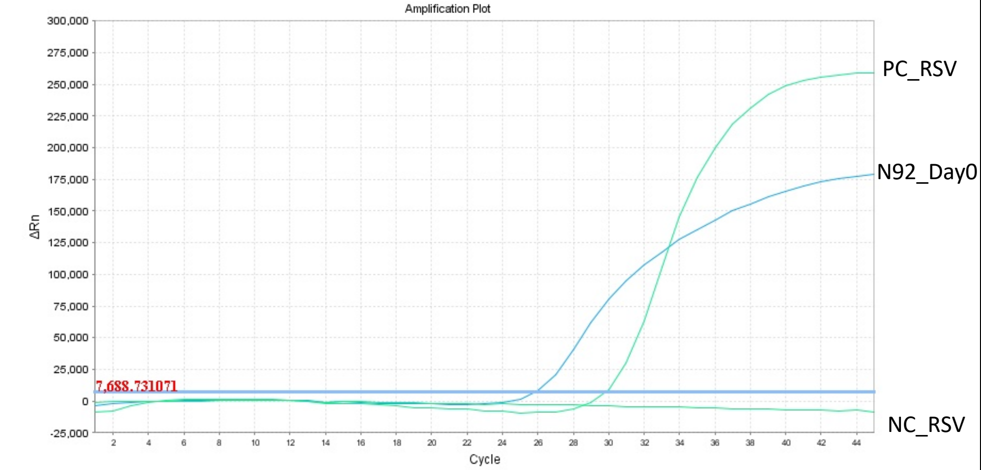	
	N92_D3	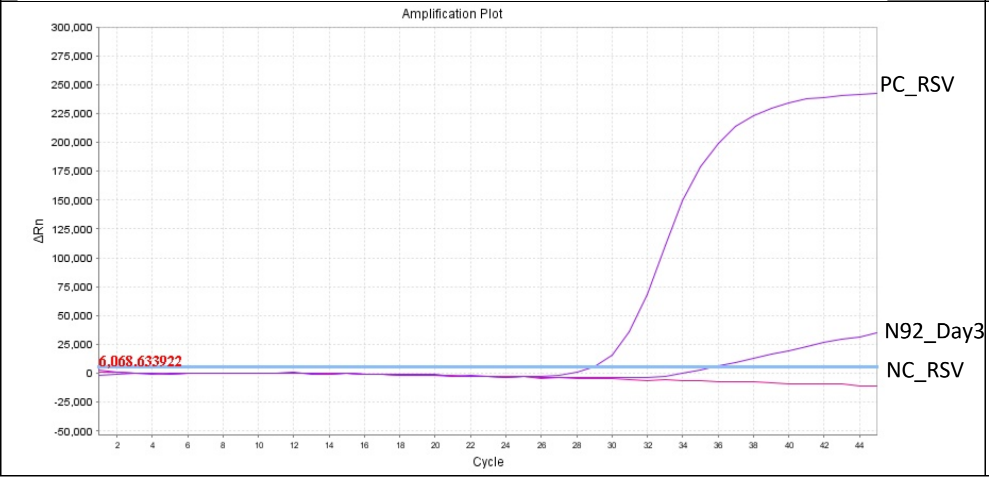	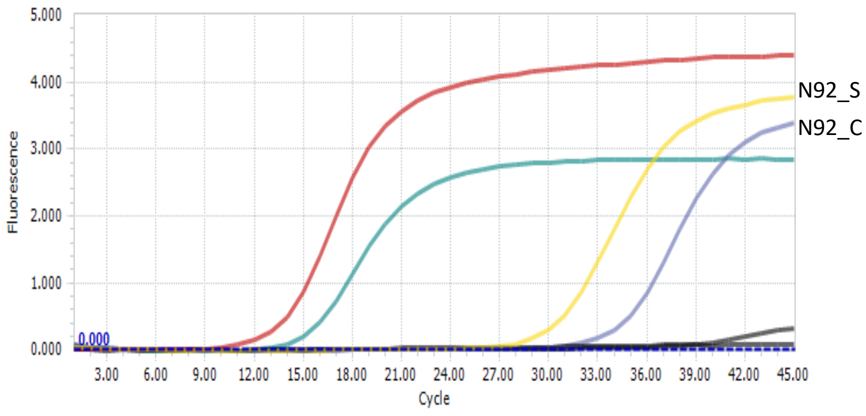

<table>
<tr><td>46</td><td>N95_D0</td><td>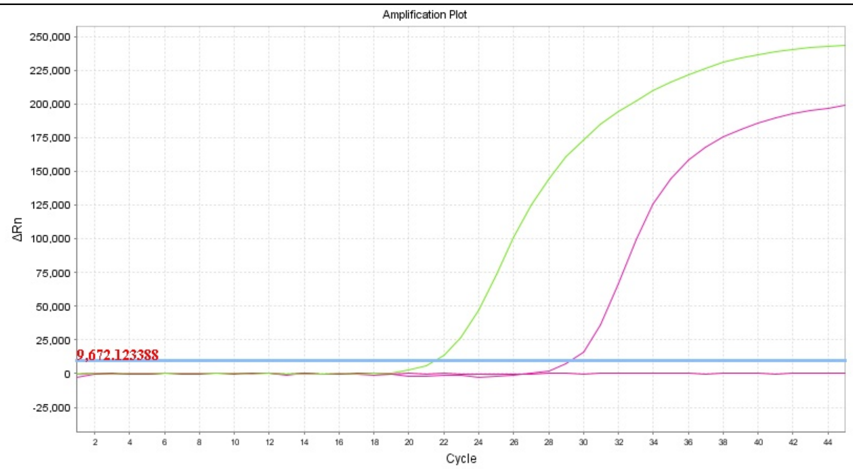</td><td></td></tr>
<tr><td></td><td>N95_D3</td><td>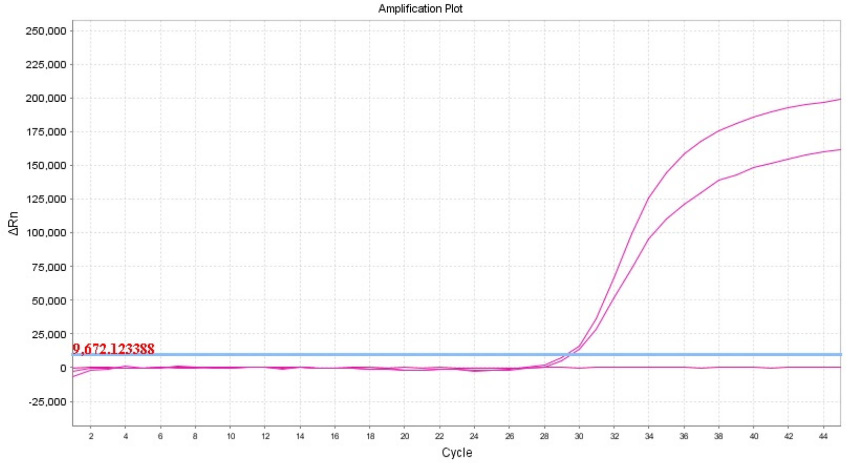</td><td>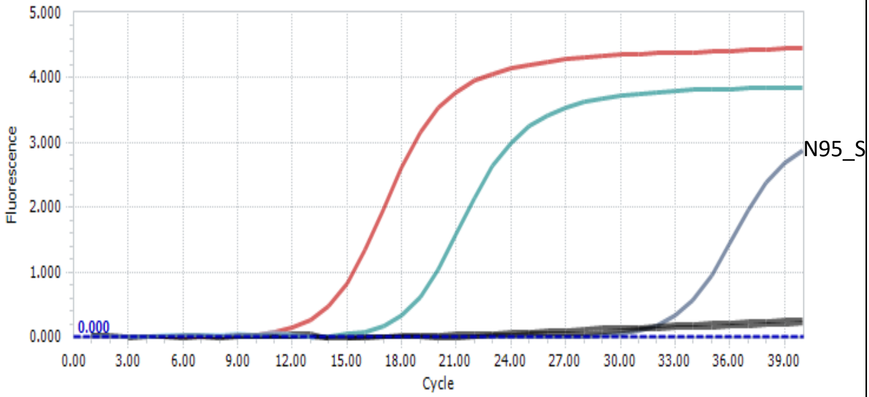</td></tr>
</table>

Fig. S2. A. Real-time PCR TaqMan probes amplification curves specifically for RSV taken from nasopharyngeal samples of Navax group at day 0 and 3 of treatment. PC, NC are positive and negative controls of RSV; B. SYBR Green real-time PCR amplification curves specifically for *B. subtilis* and *B. clausii* taken from nasopharyngeal samples of Navax group at day 3 of treatment. PC are positive controls of *B. subtilis* (red line) and *B. clausii* (blue line), NC (black) are negative controls of *B. subtilis* and *B. clausii*. By lottery, the patient's number is coded at random. The sequence of the image's appearance in the figure corresponds to the order of the patient's hospitalization.
