# Supplementary material for: Nasal-spraying Bacillus spores as an effective symptomatic treatment for children with acute respiratory syncytial virus infection
Source: Sci Rep. 2022 Jul 20;12:12402. doi: 10.1038/s41598-022-16136-z (PMC9297280; doi:10.1038/s41598-022-16136-z)
Supplement: Supplementary file 3 — Supplementary Information 3. [file 41598_2022_16136_MOESM3_ESM.pdf]

Supplemental Figures S3-S6

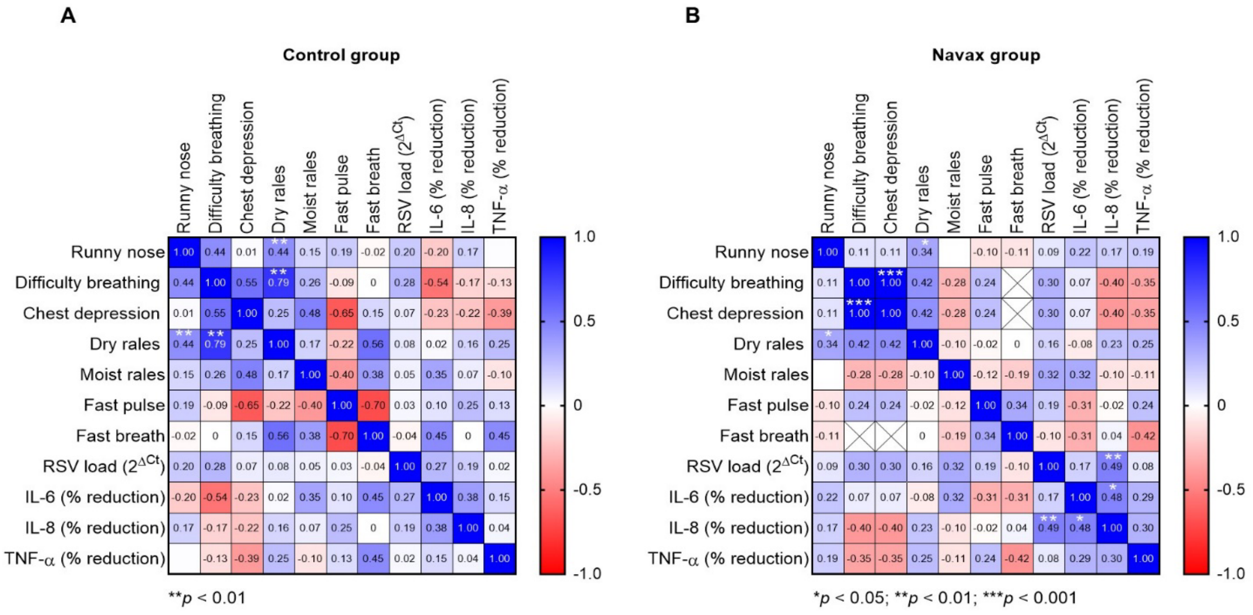

Fig. S3. Associations between symptom improvements and reductions in RSV load and in cytokine levels (IL-6, IL-8, TNF-α) in Control (A) and Navax (B) groups were assessed by Spearman's correlation analysis. Colors and numbers represent correlation factor values (blue: r>0 shows proportional correlation, red: r<0 shows inverse correlation). Some results are left blank either because only one row contains data for both variables or because one of the variables has the same value in all rows.

A.

Descriptions | Graphic Summary | Alignments | Taxonomy

Sequences producing significant alignments

Download | New Select columns | Show 10

select all *10 sequences selected*

GenBank | Graphics | Distance tree of results | New MSA Viewer

Description	Scientific Name	Max Score	Total Score	Query Cover	E value	Per. Ident	Acc. Len	Accession
Bacillus sp. SPB7 16S ribosomal RNA gene, partial sequence	Bacillus rugosus	2338	2338	100%	0.0	100.00%	1548	MT554518.1
Bacillus subtilis strain JCL16 chromosome, complete genome	Bacillus subtilis	2338	23378	100%	0.0	100.00%	4101682	CP054177.1
Bacillus subtilis strain ZIM3 16S ribosomal RNA gene, partial sequence	Bacillus subtilis	2338	2338	100%	0.0	100.00%	1544	MT539995.1
Bacillus subtilis strain KA9 16S ribosomal RNA gene, partial sequence	Bacillus subtilis	2338	2338	100%	0.0	100.00%	1550	MT491101.1
Bacillus subtilis subsp. subtilis str. 168 chromosome, complete genome	Bacillus subtilis subsp. subtilis str. 168	2338	23362	100%	0.0	100.00%	4316079	CP053102.1
Bacillus subtilis subsp. subtilis str. 168 chromosome, complete genome	Bacillus subtilis subsp. subtilis str. 168	2338	23362	100%	0.0	100.00%	4398844	CP052842.1
Bacillus subtilis subsp. subtilis strain UCMB5021 chromosome, complete genome	Bacillus subtilis subsp. subtilis	2338	23371	100%	0.0	100.00%	4060035	CP051466.1
Bacillus subtilis subsp. subtilis strain UCMB5121 chromosome, complete genome	Bacillus subtilis subsp. subtilis	2338	23378	100%	0.0	100.00%	4059834	CP051465.1
Bacillus subtilis strain At3 chromosome, complete genome	Bacillus subtilis	2338	25710	100%	0.0	100.00%	4197841	CP051462.1
Bacillus subtilis subsp. inaquosorum strain T1 16S ribosomal RNA gene, partial sequence	Bacillus inaquosorum	2338	2338	100%	0.0	100.00%	1557	MT339257.1

B.

Descriptions | Graphic Summary | Alignments | Taxonomy

Sequences producing significant alignments

Download | Manage Columns | Show 10

select all *10 sequences selected*

GenBank | Graphics | Distance tree of results

Description	Max Score	Total Score	Query Cover	E value	Per. Ident	Accession
Bacillus clausii strain BRM043935 16S ribosomal RNA gene, partial sequence	2547	2547	100%	0.0	99.57%	MH305350.1
Bacillus clausii strain ENTPro, complete genome	2547	17759	100%	0.0	99.57%	CP012475.1
Bacillus clausii strain ANA38 16S ribosomal RNA gene, partial sequence	2542	2542	100%	0.0	99.50%	MT110681.1
Bacillus clausii strain ANA37 16S ribosomal RNA gene, partial sequence	2542	2542	100%	0.0	99.50%	MT110679.1
Bacillus clausii strain ANA36 16S ribosomal RNA gene, partial sequence	2542	2542	100%	0.0	99.50%	MT107136.1
Bacillus clausii strain ANA35 16S ribosomal RNA gene, partial sequence	2542	2542	100%	0.0	99.50%	MT107086.1
Bacillus clausii strain SL4-4 16S ribosomal RNA gene, partial sequence	2542	2542	100%	0.0	99.50%	MK312486.1
Bacillus rhizosphaerae strain WA12 16S ribosomal RNA gene, partial sequence	2542	2542	100%	0.0	99.50%	KT595230.1
Bacillus clausii strain E2 16S ribosomal RNA gene, partial sequence	2542	2542	100%	0.0	99.50%	EU117277.1
Bacillus clausii KSM-K16 DNA, complete genome	2542	17715	100%	0.0	99.50%	AP006627.1

Fig S4. BLAST analysis of *Bacillus subtilis* ANA4 (A) and *B. clausii* ANA39 (B).

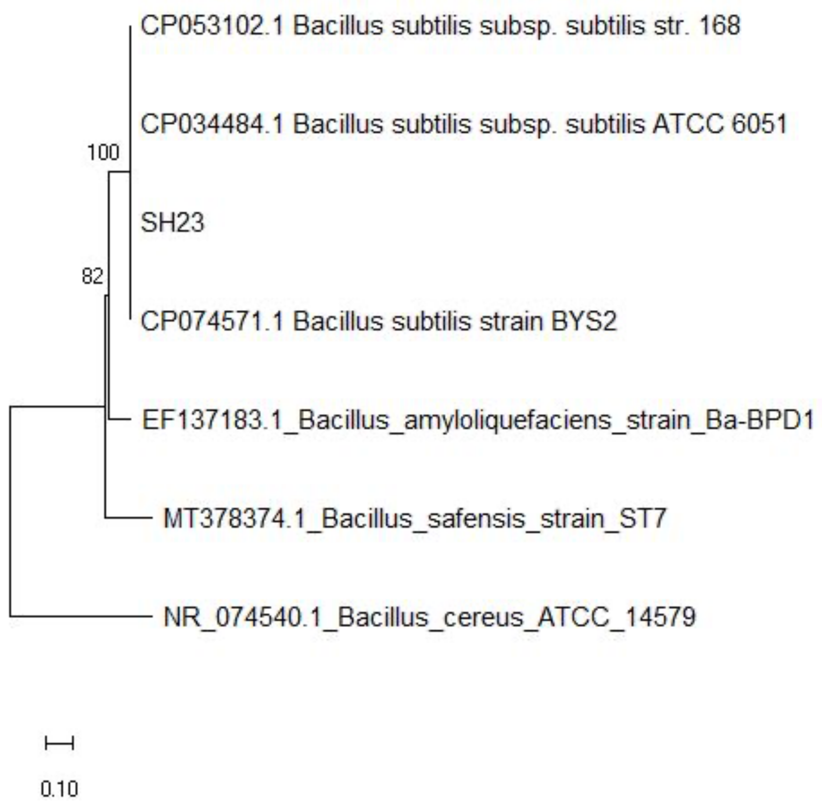

Fig. S5. Phylogenetic tree for *B. subtilis* ANA4

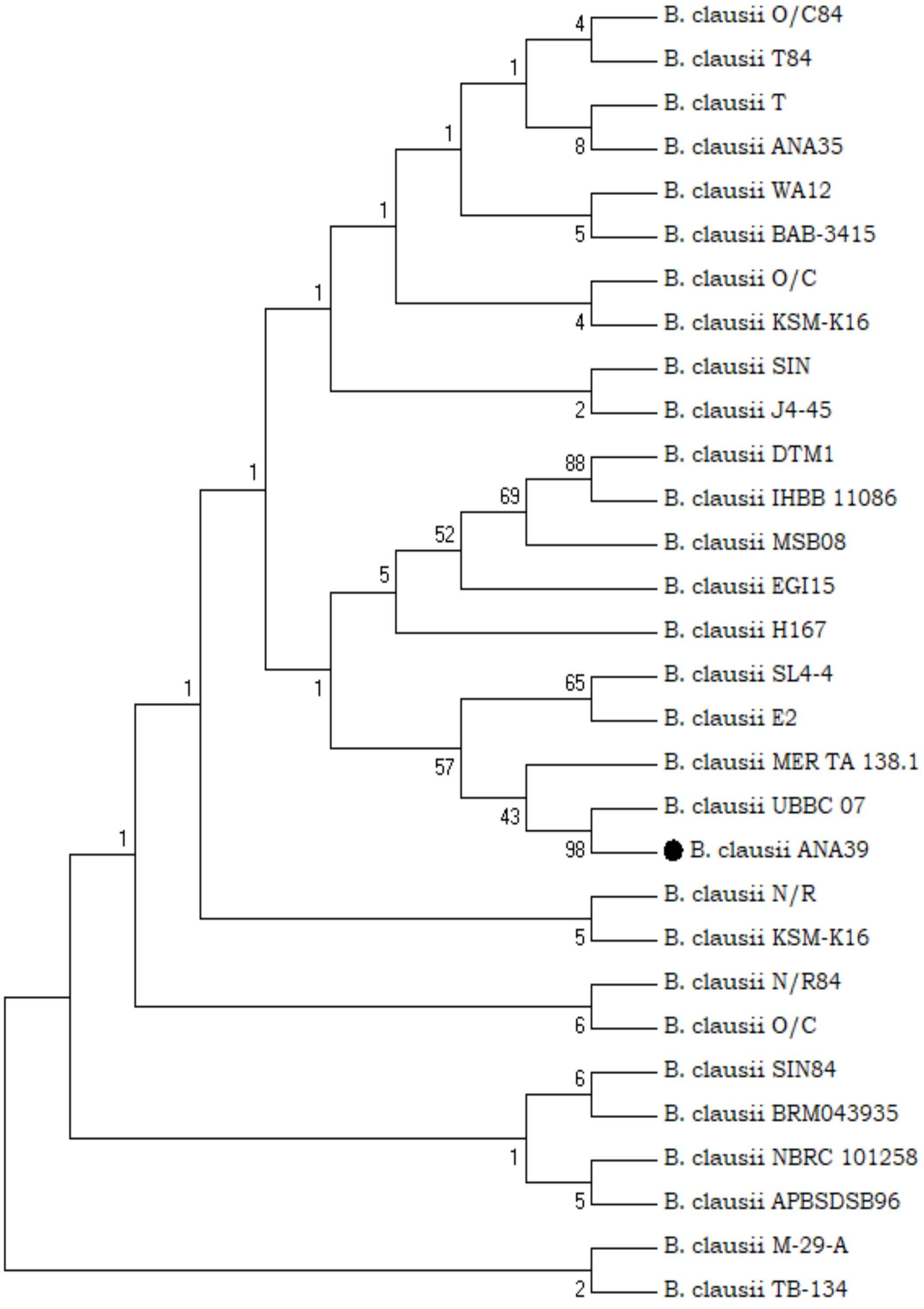

Fig. S6. Phylogenetic tree for *B. clausii* ANA39
