# Supplementary material for: Nasal-spraying Bacillus spores as an effective symptomatic treatment for children with acute respiratory syncytial virus infection
Source: Sci Rep. 2022 Jul 20;12:12402. doi: 10.1038/s41598-022-16136-z (PMC9297280; doi:10.1038/s41598-022-16136-z)
Supplement: Supplementary file 4 — Supplementary Information 4. [file 41598_2022_16136_MOESM4_ESM.pdf]

Supplemental Tables S1-S5

Table S1. Characterization of *B. subtilis* ANA4 and *B. clausii* ANA39

**Characteristics**	**ANA4**	**ANA39**
Sporulation efficiency (%)	100	90
Heat stability of spores (°C)	80	65
Width size of vegetative cell (μm)	< 1 μm	< 1 μm
Amylase	++++	++++
Caseinase	++++	+++
Lipase	+	++
Catalase	+	+
Gelatinase	++	+
Optimal temperature (°C)	37	37
Optimal pH	7.4	8.0
6.5% NaCl, 50°C	+++	-
8% NaCl, 37°C	-	+++
Aerobic	++++	+++
Anerobic	+	+++
Hemolysis	γ (No)	γ (No)
VP Test	+	-
Closest match*	*B. subtilis* (100%)	*B. clausii* (99.5%)

-, negative; +, weak or positive; ++, average; +++, good/high; ++++, very good/very high.

**Using 16S rDNA sequence analysis in this work. The similarity score is shown in brackets.*

Table S2. Antibiotic susceptibility of *Bacillus subtilis* ANA4 and *B. clausii* ANA39

**Antibiotic discs (µg)***	***B. subtilis* ANA4⁺**	***B. clausii* ANA39⁺**
Ampicillin (10)	24.34 ± 0.51 (S)	27.04 ± 1.3 (S)
Chloramphenicol (30)	26.41 ± 0.57 (S)	18.47 ± 0.20 (S)
Ciprofloxacin (5)	0 (R)	30.89 ± 0.61 (S)
Clindamycin (2)	17.05 ± 0.24 (S)	0 (R)
Cotrimoxazol (25)	28.43 ± 0.45 (S)	34.50 ± 0.95 (S)
Erythromycin (15)	22.33 ± 0.31 (S)	0 (R)
Gentamicin (10)	21.52 ± 0.10 (S)	28.22 ± 0.39 (S)
Kanamycin (30)	25.48 ± 0.24 (S)	24.84 ± 0.04 (S)
Neomycin (30)	20.29 ± 0.24 (S)	24.91 ± 0.13 (S)
Rifampicin (30)	19.68 ± 0.57 (S)	39.06 ± 0.68 (S)
Streptomycin (10)	15.17 ± 0.23 (S)	6.51 ± 0.46 (R)
Tetracycline (30)	0 (R)	27.99 ± 0.14 (S)
Trimethoprim (5)	21.13 ± 0.25 (S)	39.83 ± 0.72 (S)
Vancomycin (30)	17.67 ± 0.70 (S)	22.41 0.22 (S)

**Antibiotic-impregnated discs (6 mm) with amount in µg shown in brackets.*

⁺Diameter of inhibition zones from three individual experiments. S, sensitive; I, intermediate resistant; R, resistant.

Table S3. Sequence analysis of antibiotic resistance genes in *B. subtilis* ANA4 genome

Resistance gene	% Coverage	% Identity	Start	End	Predicted phenotype	Accession number
rifamycin-inactivating phosphotransferase *rphc*	99.58	82.45	139044	139044	Rifamycin resistance	ng_063825.1
aminoglycoside 6-adenylyltransferase *aadk*	100	98.83	624584	625438	Aminoglycoside resistance	ng_047379.1
streptothricin n-acetyltransferase *sata*	100	95.59	38226	38747	Streptothricin resistance	ng_064662.1
abc-f type ribosomal protection protein *vmlr*	100	98.54	190934	192580	Lincosamide, Streptogramin, Tiamulin resistance	ng_063831.1
macrolide 2'-phosphotransferase *mphk*	100	99.35	108040	108960	Macrolide resistance	ng_065846.1

Notes: The five genes including *rphc, aadk, sata, vmlr, mphk* classified in Ansamycin, Aminoglycoside, Lincosamide/Streptogramin/Tiamulin, and Macrolide antibiotic groups, respectively, were found. They may belong to acquired antibiotic resistant genes. Although those genes are available in the genome, their expression levels may be low so that the strain is still sensitive to tested antibiotics such as streptomycin and erythromycin… Interestingly, tetracyclin and fluoroquinolones resistance genes were not found in the genome of *B. subtilis* ANA4, suggesting that the strain is intrinsic resistance to tetracyclin and ciprofloxacin.

Table S4. Sequence analysis of antibiotic resistance genes in *B. clausii* ANA39 genome

Resistance gene	% Identity	Query / Template length	Contig	Position in contig	Predicted phenotype	Accession number
ant(4')- Ib	98.83	771 / 771	000000F |arrow	81510..82280	Aminoglycoside resistance	AJ506108
erm(34)	96.04	833 / 846	000000F |arrow	1033445..1034277	Macrolide resistance	AY234334
cat	96.79	685 / 687	000000F |arrow	2589949..2590625	Phenicol resistance	AY238971

Notes: The three genes including ant(4')-Ib classified in Aminoglycoside antibiotic group, erm(34) classified in Macrolide antibiotic group, and cat classified in Phenicol antibiotic group were found. They may belong to acquired antibiotic resistant genes. The presence of the two genes ant(4')-Ib and erm(34) are consistent with the streptomycin and erythromycin resistant phenotype of *B. clausii* ANA39 indicated by the diffusion dics assay. Although cat is available in the genome, its expression level may be low so that the strain is still sensitive to chloramphenicol. Interestingly, clindamycin resistance gene was not found in the genome of *B. clausii* ANA39, suggesting that the strain is intrinsic resistance to clindamycin.

Table S5. Sequence analysis of toxin genes in *B. subtilis* ANA4 and *B. clausii* ANA39 genomes

No	Genes	**Detection method**			
		Specific sequence amplified by PCR		***Number of gene detected by whole genome sequencing***	
		B. subtilis ANA4	*B. clausii* ANA39	*B. subtilis* ANA4	*B. clausii* ANA39
1	Hemolysin B (*hbl*B)	Not Detectable	Not Detectable	0	0
2	Non-hemolytic enterotoxin A (*nhe*A)	Not Detectable	Not Detectable	0	0
3	*nhe*B	Not Detectable	Not Detectable	0	0
4	*nhe*C	Not Detectable	Not Detectable	0	0
5	Cytotoxin K (*cyt*K)	Not Detectable	Not Detectable	0	0
